# Multimorbidity analysis and hospitalizations for diabetes before and after lockdown due to the COVID-19 pandemic in Peru

**DOI:** 10.1016/j.pmedr.2022.101884

**Published:** 2022-07-04

**Authors:** Akram Hernández-Vásquez, Antonio Barrenechea-Pulache, Andres Portocarrero-Bonifaz, Carlos Rojas-Roque, Jesús Eduardo Gamboa-Unsihuay

**Affiliations:** a Centro de Excelencia en Investigaciones Económicas y Sociales en Salud, Vicerrectorado de Investigación, Universidad San Ignacio de Loyola, Lima, Peru; b Universidad Científica del Sur, Lima, Peru; c Pontificia Universidad Católica del Perú, Lima, Peru; d Independent Researcher, León, Nicaragua; e Universidad Nacional Agraria La Molina, Lima, Peru

**Keywords:** COVID-19, Diabetes mellitus, Noncommunicable diseases, Multimorbidity, Hospitalization, Mortality, Peru

## Abstract

COVID-19 has disrupted the treatment of non-communicable diseases (NCDs). This study conducted a multimorbidity analysis and evaluated hospital admissions and death rates among diabetic patients before and after the implementation of lockdown due to the COVID-19 pandemic in Peru. Data from the Ministry of Health (MINSA) of Peru from January 2017 to December 2020 was used. Hospital death, discharge and the percentage of death/hospital admissions were defined as outcomes of interest. We performed an interrupted time series analysis to assess the aggregate change in the outcomes of interest before and after mandatory lockdown in response to the COVID-19 pandemic in Peru (n = 65,935). Additionally, a network analysis was performed to evaluate the frequency of occurrence of hospital admissions before and after the mandatory lockdown according to demographic characteristics. The average monthly hospital admissions among diabetic patients in Peru decreased by 29% after the implementation of the lockdown. The largest reduction was observed in women (−41%) and for patients 60 years or older (−35%). Furthermore, there was a 92% increase in the average number of monthly deaths. The largest percentage change occurred in men (+113%) and in the group of 40–59 years (+144%). After the implementation of lockdown in Peru, hospital admissions among diabetic patients significantly decreased while in-hospital mortality slightly increased. Our findings shed light on the limitations of the Peruvian health system and the importance of ensuring continued care of NCDs as part of the response strategy during times of crisis.

## 1 Introduction

Non-communicable diseases (NCDs) are responsible for 74.37% of global deaths (Vos et al., 2020). It is estimated that 25% of the population in the Americas lives with at least one NCD (World Health Organization, 2020). Diabetes is a leading NCD and caused 1.55 million deaths in 2019. The disability adjusted life years (DALYs) associated to diabetes is 70.9 million for people of all ages.

(Vos et al., 2020). Worldwide, implementation of restrictive measures attempting to curb the growing COVID-19 cases have interrupted essential health services in 90% of countries, with greater disruptions being reported in low- and middle-income countries (LMIC) (World Health Organization, 2020). This has led to the interruption of treatment for chronic diseases and a decrease in routine follow-ups for all “non-urgent” conditions (Imlach et al., 2021). For example, a systematic review estimated that health care utilization decreased by about a third, with a median reduction of 42% and 30% for visits and therapeutics, respectively (Moynihan et al., 2021). This situation negatively impacts the health of patients with NCDs like diabetes which requires regular patient–provider interactions for patient education, prescriptions and management of complications (Beran et al., 2021).

In the USA and Europe, up to 50% of patients with NCDs have reported a worsening of their medical condition, while 26% described a negative impact of the pandemic on their long-term treatment intake (Pécout et al., 2021). Hospitalizations for acute cardiovascular, chronic obstructive pulmonary disease and diabetes have decreased (Bhatt et al., 2020, Blecker et al., 2021, Lui et al., 2020). Current diabetes guidelines recommend that patients that have not met glycemic control objectives or have recently changed medication should be evaluated at least every three months (Association, 2021). Evaluation for microvascular complications of diabetes should be carried out at least once a year. Yet, up to 47% of diabetes nurses across Europe have reported that the pandemic has led to severe or extreme disruption to routine diabetes care, citing diabetes education, psychological and self-management support as the areas with the greatest disruption (Forde et al., 2021). As in India, it is likely that marginalized populations have faced the most difficulties in accessing health care during this time (Singh et al., 2021). In high income countries such as the UK increased deaths by all causes in private homes (owned by an individual) have already been reported, and this situation would only be more severe in LMIC which often have weaker health care systems (Higginson et al., 2021).

In spite of its increase in social and economic indicators, Peru suffers from longstanding structural deficiencies which have made it particularly vulnerable during the COVID-19 pandemic, contributing to its high death rate (Carrillo-Larco et al., 2022). In 2018, Peru only invested 5.24% of its GDP on health care and had a lack of personnel, medications, supplies and diagnostic tests (The World Bank, 2021, Soto, 2019). Care for diabetes was found to be deficient prior to the pandemic with low political commitment by the government being cited as the main impediment for the improvement of provision of care. This situation as well as other factors were proposed to contribute to one third of diabetic patients in Lima, the capital of Peru, being diagnosed in the emergency department after presenting diabetes-related complications (Cardenas et al., 2016). With the nation-wide lockdown implemented in March 2020, patients with chronic diseases were unattended (Herrera-Añazco et al., 2021). A case series conducted in Lima concluded that 7 out of every 10 non-COVID-19-related hospitalizations were due to not receiving timely treatment, with 1 of every 5 of these patients dying, suggesting that these measures have caused excess death among people with NCDs (Málaga, 2020). Nevertheless, a formal analysis of the impact that lockdown has had on hospitalization and death rates due to diabetes has yet to be conducted.

The objective of the current study is to conduct a multimorbidity analysis and evaluate hospitalization and death rates due to diabetes before and after the implementation of lockdown in Peru.

## 2 Methods

### 2.1 Study setting

As of 2017 approximately 75% of the Peruvian population reported having healthcare insurance, an increase of over 33 percentage points compared to what was found in 2007 (Instituto Nacional de Estadística e Informática, 2018). The healthcare system is fragmented into *Sistema integral de Salud* (SIS) for the poor and extremely poor [administered directly by the Ministry of Health of Peru (MINSA)], Social security (EsSalud) for dependent workers and their legal beneficiaries, police and armed forces, and the private sector (Lazo-Gonzales et al., 2016). Approximately 65% of the population is covered by SIS (Organisation for Economic Co-operation and Development, 2017). Despite SIS having a wide national distribution of primary health centers they often have limited equipment and resources, especially in rural and hard-to-reach areas (Carrillo-Larco et al., 2022).

### 2.2 Study design

The database on hospital admissions of the MINSA was used to conduct this cross-sectional study. It provides nationally representative data with information on hospital admissions due to any cause, in any public hospital and specialized institute of Peru belonging to the MINSA from January 2017 to December 2020. This database provides basic demographic characteristics of hospitalized individuals along with ICD-10 codes for the admission and discharge diagnosis as well as state of discharge (alive/dead).

### 2.3 Sample

The database included a total of 3,263,154 individuals who were hospitalized during the study period. We included data from individuals 18 years of age or older admitted to the hospital with diabetes (ICD-10 codes from E100 to E149) as one of the four admission diagnoses that the registry system allows to enter upon the arrival of the patient. Individuals with missing values in the outcomes of interest or with other admission codes were excluded.

### 2.4 Outcomes of interest

Among the diabetic patients hospitalized in public hospitals between January 2017 and December 2020 we defined the following outcomes of interest: in-hospital death, discharge, and the percentage of death/hospital admissions. Except for the death/hospital admissions, all the outcomes are a continuous variable measured monthly.

### 2.5 Variable stratification

We included two groups for variable stratification representing the demographic and clinical characteristics of the individuals. The demographic variables included sex (male or female) and age (18–39 years / 40–59 years / 60 years or more). We chose these cut-off points to: better characterize early-onset adult type 2 diabetic patients (Cardenas et al., 2016, Dickey and Fuller, 1979, Ding et al., 2020, Durbin and Watson, 1951, Fried and Gather, 2016, Fuller, 1996, Herrera-Añazco et al., 2021, Khow et al., 2021, Lazo-Gonzales et al., 2016, Málaga, 2020, Organisation for Economic Co-operation and Development, 2017, Phillips and Perron, 1988, Sargeant et al., 2020, Zimmet et al., 2014, Instituto Nacional de Estadística e Informática, 2018, Congreso de la República del Perú, 2016, World Health Organization, 2010, Lopez Bernal, 2016, Kontopantelis et al., 2015, Wickham et al., 2021, Wickham, 2021, Csardi and Nepusz, 2005), specify the largest group of working people with diabetes (Bastani et al., 2021, Ccorahua-Ríos et al., 2019, Chang et al., 2021, Erener, 2020, Flood et al., 2021, Flores-Flores et al., 2018, Gianella et al., 2021, Gregg et al., 2021, Instituto Nacional de Estadística e Informática, 2021, Kshanti et al., 2021, Pesantes et al., 2020, Poppe, 2020, Vázquez-Rowe and Gandolfi, 2020, Taylor, 2021, Dyer, 2021, Mostajo-Radji, 2021, Ceron, 2011, Jumpa-Armas, 2019, Seclén Santisteban, 2021, Custodio-Sánchez et al., 2020), and include the definition of an elderly person in Peru (≥60) (Khow et al., 2021, Sargeant et al., 2020, Zimmet et al., 2014, Congreso de la República del Perú, 2016). Clinical characteristics were the presence of other comorbidities of the individual. The comorbidities were defined using the ICD-10 codes, developed by the World Health Organization (World Health Organization, 2010).

### 2.6 Statistical analysis

The interrupted time series was conducted using Stata v14.2 (Stata Corporation, College Station, Texas, USA), and the R software (R Foundation for Statistical Computing, Vienna, Austria) was used to perform the network analysis. In all the analyses, statistical significance was evaluated with a *p* < 0.05.

First, we defined an intervention variable which takes the value of one after implementing the mandatory lockdown in response to the COVID-19 pandemic in Peru (from March 2020 to December 2020), and zero otherwise (from January 2017 to February 2020). This intervention variable was used to perform an interrupted time series analysis (ITS) to assess the aggregate change in the outcomes of interest before and after the mandatory lockdown in Peru. The ITS is a methodology used to evaluate changes in longitudinal series after a quasi-experimental intervention that occurs at a set point in time (Lopez Bernal, 2016, Kontopantelis et al., 2015). In the analysis, we used the following equation to run the statistical model:

(1) $$Y_{t}=\beta_{0}+\beta_{1}T_{t}+\beta_{2}I_{t}+\beta_{3}{\mathrm{TI}}_{t}+\mu_{t}$$

In equation (1), $Y_{t}$ is the aggregate outcome of interest measured on a monthly basis, $T_{t}$ is the time, measured every month that has passed since the start of the study (from month 1 to month 48), $I_{t}$ is the intervention variable and ${\mathrm{TI}}_{t}$ is an interaction term. Thus, $\beta_{0}$ is the mean value of the outcome of interest at the start of the study, $\beta_{1}$ is the value of the slope of the outcome of interest, $\beta_{2}$ is the change in the slope of the outcome of interest immediately after the intervention, $\beta_{3}$ is the difference of the slope after and before the intervention and $\mu_{t}$ represents the stochastic term of the statistical model. The coefficient $\beta_{3}$ is akin to a difference-in-difference (DID) slope.

For all the outcomes of interest, the stationarity of the series was assessed using the augmented Dickey-Fuller test for unit root (Fuller, 1996, Dickey and Fuller, 1979) and the Phillip-Perron test for unit root (Phillips and Perron, 1988). In addition, the autocorrelation for each outcome of interest was assessed by plotting the correlogram and by estimating the Durbin-Watson test (Durbin and Watson, 1951). When autocorrelation of first order appeared, we corrected it by estimating Prais-Winsten AR (1) regression (Fried and Gather, 2016).

We then perform a network analysis to evaluate the frequency of co-occurrence of diagnosis for hospital admissions according to demographic characteristics and before and after the mandatory lockdown in response to the COVID-19 pandemic. To do this, CIE-10 codes from E100 to E149 were grouped just as diabetes, and the dataset was split according to various criteria: whether the hospitalization was before (from January 2017 to March 2020) or after the pandemic lockdown (from March to December 2020), whether the patient lived or died after admission to a hospital, and by sex and age group (Cardenas et al., 2016, Dickey and Fuller, 1979, Ding et al., 2020, Durbin and Watson, 1951, Fried and Gather, 2016, Fuller, 1996, Herrera-Añazco et al., 2021, Khow et al., 2021, Lazo-Gonzales et al., 2016, Málaga, 2020, Organisation for Economic Co-operation and Development, 2017, Phillips and Perron, 1988, Sargeant et al., 2020, Zimmet et al., 2014, Instituto Nacional de Estadística e Informática, 2018, Congreso de la República del Perú, 2016, World Health Organization, 2010, Lopez Bernal, 2016, Kontopantelis et al., 2015, Wickham et al., 2021, Wickham, 2021, Csardi and Nepusz, 2005);(Bastani et al., 2021, Ccorahua-Ríos et al., 2019, Chang et al., 2021, Erener, 2020, Flood et al., 2021, Flores-Flores et al., 2018, Gianella et al., 2021, Gregg et al., 2021, Instituto Nacional de Estadística e Informática, 2021, Kshanti et al., 2021, Pesantes et al., 2020, Poppe, 2020, Vázquez-Rowe and Gandolfi, 2020, Taylor, 2021, Dyer, 2021, Mostajo-Radji, 2021, Ceron, 2011, Jumpa-Armas, 2019, Seclén Santisteban, 2021, Custodio-Sánchez et al., 2020) of the patient. In each study interest group a network analysis was carried out, in which pairs of diagnoses that were repeated in a frequency greater than 10% of the total records were selected; the most frequent admission codes were chosen in cases in which the number of admission code pairs was greater than 20. Each pair of diagnoses was linked by a line, where a greater width corresponds to a greater occurrence of the pair. The width of the line was standardized so that it was proportional to the ratio frequency of occurrence and number of instances. The graphs were built with R language, using the dplyr (Wickham et al., 2021); tidyr (Wickham, 2021) and igraph (Csardi and Nepusz, 2005) packages.

### 2.7 Ethics statement

The Ministry of Health of Peru provided the anonymized database on hospitalizations, after a request for access to public information (http://www.minsa.gob.pe/portada/transparencia/solicitud/frmformulario.asp). Ethical approval was not required for this research due to the public and anonymous nature of the data used.

## 3 Results

After applying inclusion and exclusion criteria, the final sample consisted of 65,935 observations. Overall, the average monthly hospital admissions due to diabetes decreased by 29% during the pandemic compared to before lockdown. The highest percentage change was among individuals aged 60 years and older, with a 35% reduction. Moreover, the highest reduction (45%) was found among the oldest women studied. Regarding deaths of hospitalized diabetic patients, overall, there was a 92% increase in the average number of monthly deaths during the pandemic compared to before lockdown. The highest percentage change was observed among individuals aged 40–59 years, with a 113% increase. The increase in deaths was most noticeable among men in all age categories. The average monthly number of deaths tripled among diabetic men aged 18–39 years and increased by 144% among those aged 40–59 years (Table 1).

**Table 1:** Mean monthly hospital admissions and in-hospital deaths among Peruvian patients with diabetes, 2017–2020.

	Hospital admissions			Deaths		
	Men	Women	Total	Men	Women	Total
**Age 18**–**39 years**						
During the COVID-19 pandemic	57 (9.1)	57 (7.7)	114 (14.5)	3 (2.2)	2 (1.5)	5 (2.6)
Mean in equivalent time periods before lockdown	58 (10.7)	82 (11.3)	140 (17.8)	1 (1.1)	1 (0.9)	3 (1.3)
Percentage change	−2%	−30%	−19%	200%	100%	67%
**Age 40**–**59 years**						
During the COVID-19 pandemic	234 (46.5)	204 (37.5)	437 (74.5)	22 (15.6)	12 (8.0)	34 (22.4)
Mean in equivalent time periods before lockdown	248 (24.8)	327 (29.9)	576 (47.1)	9 (3.7)	8 (2.6)	16 (4.7)
Percentage change	−6%	−38%	−24%	144%	50%	113%
**Age ≥60 years**						
During the COVID-19 pandemic	232 (44.0)	253 (49.2)	485 (86.7)	42 (23.8)	40 (17.1)	82 (40.0)
Mean in equivalent time periods before lockdown	290 (29.0)	457 (36.1)	748 (58.8)	19 (4.3)	26 (5.5)	44 (7.0)
Percentage change	−20%	−45%	−35%	121%	54%	86%
**Overall**						
During the COVID-19 pandemic	522 (95.3)	513 (91.0)	1035 (171.3)	67 (40.0)	54 (24.3)	121 (63.5)
Mean in equivalent time periods before lockdown	596 (53.6)	867 (64.8)	1463 (109.0)	29 (6.0)	35 (6.8)	63 (8.8)
Percentage change	−12%	−41%	−29%	131%	54%	92%

The table describes means and standard deviation (SD) unless otherwise stated.

Means have been rounded to whole numbers.

Period before lockdown: from January 2017 to February 2020. Period during the COVID-19 pandemic: from March to December 2020.

The lockdown started on March 16, 2020.

### 3.1 Hospital admissions of diabetic patients before and after lockdown

**Fig. 1:**
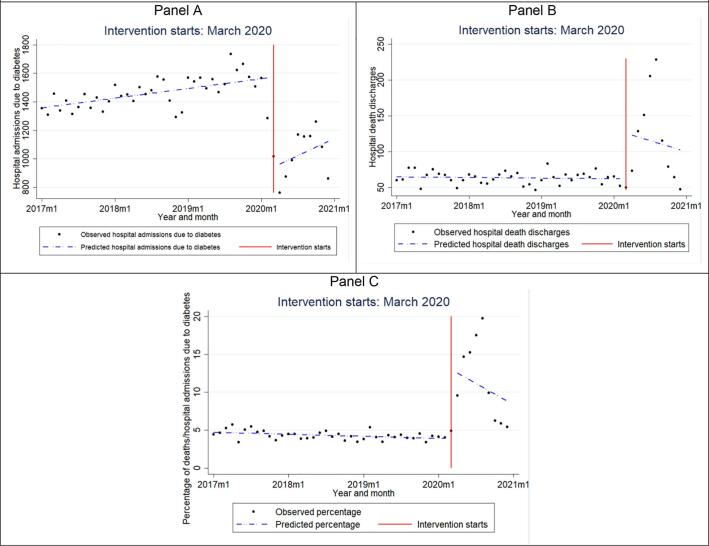
Interrupted Time Series: Hospitalizations and in-hospital deaths diabetic patients from 2017 to 2020. A. Hospital admissions of diabetic patients before and after lockdown; B. Discharge status of diabetic patients before and after lockdown. C. Percentage of deaths/hospital admissions of diabetic patients before and after lockdown.

Regarding hospital admissions of diabetic patients there was a positive linear slope from 2017 to March 2020. The difference of the slope after and before the intervention was −2.4 admissions per month (95% CI –32.56 to 27.70; p < 0.872) in the number of cases (Panel A of Fig. 1, Table 2).

**Table 2:** Difference-in-difference estimates.

**Variable**	**Coefficient**	**95% CI**	***p*-value**
**Outcome 1. Hospital admissions due to diabetes^a^**			
Time, in months	4.88	0.42 to 9.32	0.033
Intervention	−426.04	−1716.43 to 864.34	0.509
Interaction term	−2.43	–32.56 to 27.70	0.872
Constant	1362.78	1262.22 to 1463.33	<0.001
**Outcome 2. In-hospital deaths^a^**			
Time, in months	−0.037	−0.82 to 0.74	0.925
Intervention	256.785	18.33 to 495.23	0.035
Interaction term	−5.522	−11.10 to 0.05	0.052
Constant	22.161	−1.54 to 45.86	0.066
**Outcome 3. Percentage of in-hospital deaths/hospital admissions^a^**			
Time, in months	−0.008	−0.06 to 0.04	0.771
Intervention	30.477	14.07 to 46.87	0.001
Interaction term	−0.649	−1.03 to −0.26	0.001
Constant	1.441	−0.08 to 2.96	0.063

^a^Estimated using the Prais-Winsten AR (1) regression.

CI: confidence interval.

### 3.2 Discharge status of diabetic patients before and after lockdown

As with hospital admissions, a positive linear slope was found in relation to the death of hospitalized diabetic patients from 2017 to March 2020. Upon the implementation of total lockdown, there was a noticeable increase in deaths from April which peaked in August 2020 (Panel B and C of Figure 1), followed by a decline to normal pre-pandemic levels of death in December. The difference of the slope after and before the lockdown was −5.522, 95% CI: −11.10 to 0.05; p = 0.052 (Panel B of Fig. 1, Table 2).

### 3.3 General, Sex, and age divided multimorbidity network analysis

**Fig. 2:**
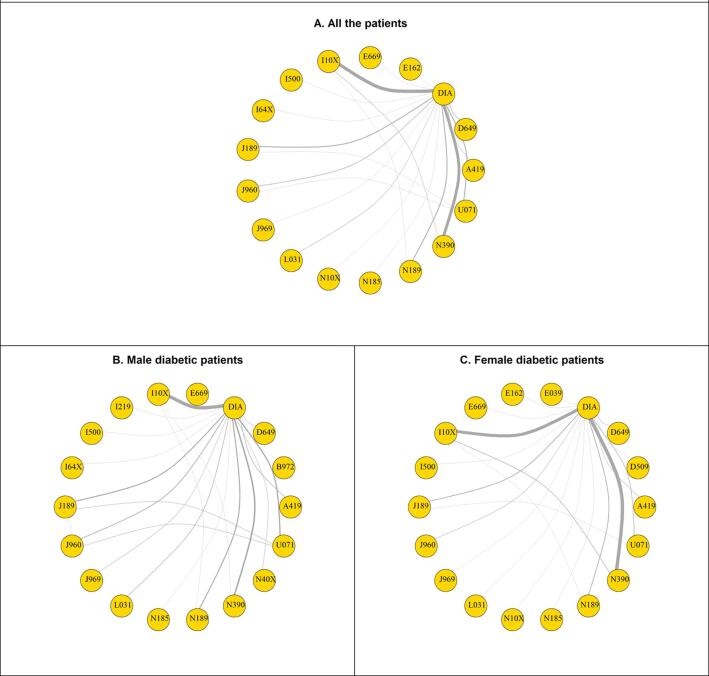

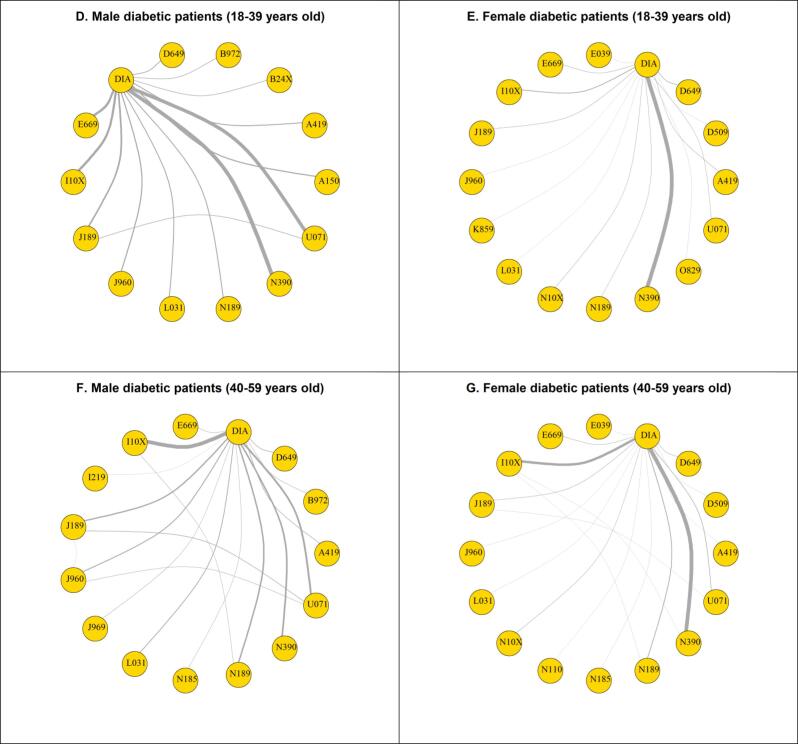

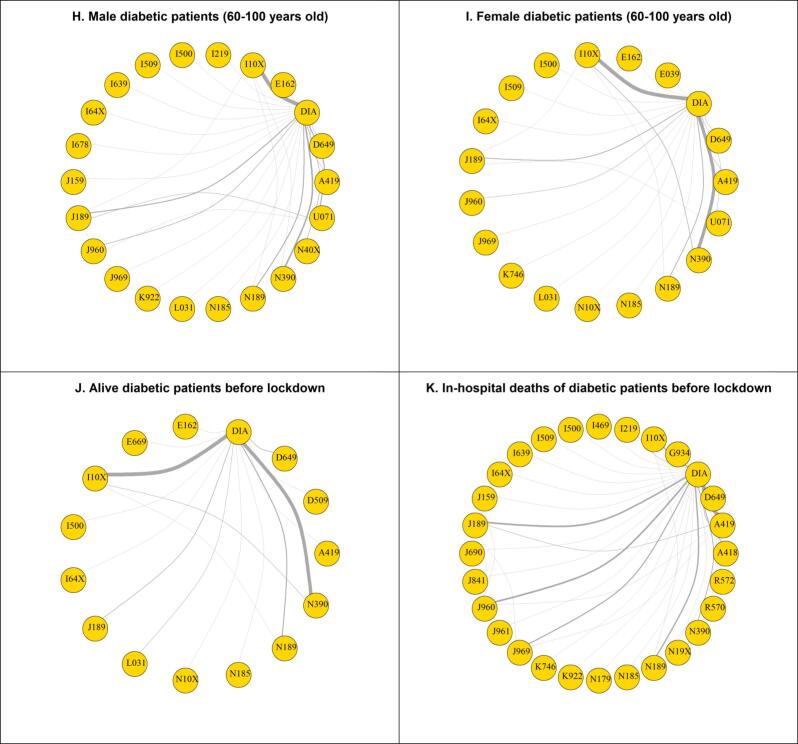

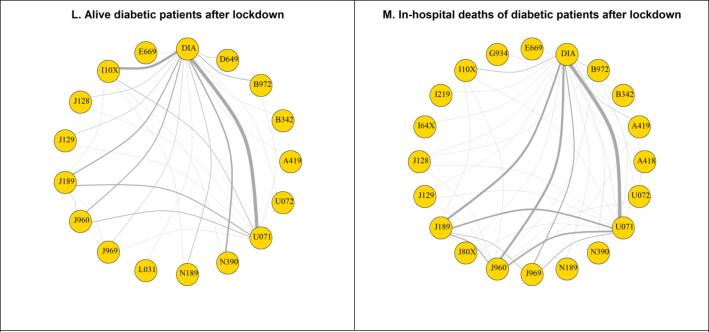
Multi-morbidity network analysis.

The multimorbidity network analysis from January 2017 to December 2020 shown in Panel A of Fig. 2 shows that among all patients admitted with diabetes ICD codes hypertension (I10.X), and urinary tract infection (N39.0) were the comorbidities with highest correlation. Even though the first COVID-19 (U07.1) case was detected around March 2020 a correlation between the two is observed. For women, I10.X and N39.0 have the highest relevance (Panel C of Fig. 2), while only I10X is strongly correlated for men (Panel B of Fig. 2). Lastly, when the analysis was performed for patients with diabetes in different age groups, the highest associations for the group of 18 to 39 years were with COVID-19 (U07.1) for males and with urinary tract infection (N39.0) for both sexes. For the group of 40 to 59 and also of 60 to 100 the greatest associations were with urinary tract infection (N39.0) for women and with hypertension (I10.X) for both sexes (Panel D, E, F, G, H, I of Fig. 2). All pairwise comorbidities are shown in Supplementary material.

### 3.4 Pre and post lockdown multimorbidity network analysis

Among patients discharged alive from hospitalization showed that, pre-lockdown (Panel J of Fig. 2), admission diagnosis of diabetes was correlated with concomitant diagnosis of hypertension (I10.X) and urinary tract infection (N39.0). Meanwhile, among diabetic patients that died before lockdown (Panel K of Fig. 2) the main diagnosis codes were sepsis (A41.9), followed by pneumonia (J18.9), respiratory failure (J96.9, J96.0), and chronic renal disease (N18.9). In contrast, after the implementation of the lockdown, COVID-19 and hypertension (U07.1 and I10.X) were the most important concomitant admission diagnosis for diabetic patients that were discharged from hospital alive (Panel L of Fig. 2). Whereas there was a stronger correlation between diagnosis of diabetes, COVID-19 virus identified (U07.1), respiratory failure (J96.0), and pneumonia (J18.9) among patients that died (Panel M of Fig. 2).

## 4 Discussion

We conducted a multimorbidity analysis and ITS among Peruvians with a hospital admission diagnosis of diabetes between January 2017 and December 2020. Before the start of the COVID-19 pandemic, deaths among hospitalized diabetic patients were correlated mainly with sepsis, followed by pneumonia, respiratory failure, and chronic kidney disease. After lockdown (intervention) the correlations shifted towards COVID-19 (U07.1), respiratory failure and pneumonia (J96.0, J18.9). The average monthly number of hospitalizations decreased after lockdown, with a subsequent increase in the number of deaths which peaked around August 2020.

### 4.1 Hospitalizations and deaths among diabetic patients

In Peru, the regular pre-pandemic trend for diabetes-related hospital admissions was around 1363 per month and decreased after the lockdown the trend reduced by 426.04 cases. The reduction is comparable to a systematic review that found that the utilization of health care services decreased by a third during the pandemic, with a median 28.4% reduction in admissions (Moynihan et al., 2021). Peru also focused most of its resources on containment of COVID-19, further neglecting chronic diseases (Herrera-Añazco et al., 2021). Many patients cited fear of being infected and the perception of their illness not being “severe enough” as barriers to attending the hospital (Blecker et al., 2021, Ding et al., 2020). In Peru, daily reports of rising case numbers, implementation of severe containment measures, images of lines of patients waiting to receive attention in overflowing hospitals and families desperately searching for oxygen for their loved ones likely dissuaded many patients from seeking health care (Taylor, 2021, Dyer, 2021, Vázquez-Rowe and Gandolfi, 2020). Likewise, across Latin America, the spread of fake news, politicization of health interventions and conflicting information on how to control the disease contributed to mistrust and a general sense of helplessness in the face of widespread disaster (Mostajo-Radji, 2021, Ceron, 2011). Hence, uncertainty lead to decreased hospitalizations after the implementation of lockdown.

The reduction in hospitalized diabetic patients was most noticeable among women and those aged 60 years and older. The elderly experience more barriers to access health care during the pandemic due to greater disability, low availability of routine care, limited access to home visits, lower digital literacy and economic dependence (Bastani et al., 2021). In Peru, adults over the age of 65 who live in poverty have limited access to health care irrespective of their physical capacities, and those who are insured seldom have access to preventive health services (Flores-Flores et al., 2018). Indeed, insurance does not guarantee access to care due to structural deficiencies of the healthcare system, which has failed to expand its supply of services despite the growing number of individuals with insurance (Instituto Nacional de Estadística e Informática, 2018, Jumpa-Armas, 2019). After lockdown, Peruvian outpatient services were closed for months (Pesantes et al., 2020). This probably caused many patients to modify their medication and experience lifestyle disruptions which would lead to an increase in mortality, both in hospitals and in the community (Pécout et al., 2021, Higginson et al., 2021, Gianella et al., 2021, Chang et al., 2021). In Indonesia, up to 70% of diabetic survey respondents faced difficulties managing their diabetes during the pandemic. Over half reported that they let their condition deteriorate, while a third reported that they discontinued their medication, leading 24% to develop diabetic complications (Kshanti et al., 2021). These deficiencies predominantly affect the most vulnerable populations. The elderly in Peru, particularly those with the greatest economic limitations who are most likely to access public hospitals, faced the greatest barriers to access health care during the pandemic.

There was a 92% increase in the average amount of deaths among hospitalized diabetic patients. A local study comparing deaths reported between 2019 and 2020 found that in July 2020 there was a 200% increase in the total deaths by any cause (Seclén Santisteban, 2021). Deaths associated with NCDs in non-COVID patients were significantly increased compared to 2019. This is consistent with our study, in which the months of July and August had the highest number of in-hospital deaths. The wide variations of in-hospital death from month to month is likely why the DID analysis shows an interaction term of −5.522 (95% CI-11.10 to 0.05; *p* = 0.052). Generalized quarantine did not guarantee a reduction in the number of deaths or contagion. In Peru and Ecuador, macro indicators such as low access to sanitation, drinking water and informal working status likely hindered compliance with lockdown and led to increased contagion compared to Colombia (Poppe, 2020). The lack of a comprehensive plan to follow-up of NCDs in Peru likely contributed to an increase in the amount of patients presenting with Myocardial Infarction 24 h after symptom onset during the first 45 days of lockdown (Pesantes et al., 2020, Custodio-Sánchez et al., 2020). Overall mortality rates in the general diabetic population have been estimated to be 50% higher than historical trends (Gregg et al., 2021). Thus, the lack of a structured and context-sensitive plan to care for NCD patients as part of the strategy to face the COVID-19 pandemic has contributed to excess mortality among diabetic patients in Peru.

### 4.2 Multimorbidity analysis

Between January 2017 and March 2020 deaths among hospitalized diabetic patients most frequently occurred among those with infectious diseases or respiratory failure at admission. Poorly controlled diabetic patients have been found to be at increased risk for serious infections, with significantly increased hospitalization and mortality rates (Erener, 2020). In Peru, only 67.9% of people who report having been diagnosed with diabetes reported having received or bought medication for the disease within the last 12 months. The proportion of individuals who obtained medication is lower among residents of the highlands (59.2%), historically the most underserved population versus the coast or jungle (71.8 and 71.9% respectively) (Instituto Nacional de Estadística e Informática, 2021). This suggests that at least a third of the diabetic population in Peru is not receiving any treatment for their disease. In LMIC only around 4.6% of diabetic patients meet the needs for all treatments recommended to them. Only 8.1% of patients are fully covered with the indicated pharmacological treatment, while non-pharmacological treatment coverage reaches 25.2% (Flood et al., 2021). In Lima, lack of resources at the primary care level forces many diabetic patients to be referred for treatment with specialists, where little time is dedicated to patient education. This leads some to believe that they can manage their disease by relying solely on medication (Cardenas et al., 2016). Furthermore, insurance does not provide patients with equipment for self-monitoring blood sugar levels, impeding adequate daily glycemic control. The dependence on tertiary prevention has likely contributed to the 10-fold increase in the diabetes prevalence in Peruvians younger than 30 years between 2005 and 2018 (from 2.1 to 22.1 per 100 000 inhabitants), rivaling that of first world countries (Ccorahua-Ríos et al., 2019).

After lockdown, an increase in the number of deaths was found among diabetic patients with a diagnosis of COVID-19 and other respiratory illnesses at admission. Preexisting diabetes increases the risk of severe or critical illness and in-hospital mortality two- to three-fold independently of age, sex and other confounders (Corona et al., 2021, Mantovani et al., 2020). Given the poor level of control most Peruvian diabetic patients can achieve, and the additional difficulties that lockdown placed on them, they were at an increased risk of contagion and death during the COVID-19 pandemic. Major initiatives should take place to improve the healthcare system, particularly in the primary care level, to ensure adequate control of chronic diseases irrespective of the state of emergency.

## 5 Limitations

The cross-sectional nature of the data set prevents us from establishing a causal relationship between the study variables. Secondly, the data we employed is subject to correct and complete filling of the diagnostic information of each hospitalized patient by the doctors performing the initial treatment. It is likely that sometimes the diagnosis of diabetes mellitus was not adequately recorded during hospital admission, which would lead to underreporting of the number of cases included in the study. We did not consider discharge diagnosis or cause of death, which may have differed from the initial cause of admission. Also, the database used includes only patients hospitalized in centers administered by the Ministry of Health (MINSA). Lastly the results may be affected by the higher frequency of deaths at home in times of pandemic, where people with diabetes did not go to a health facility for fear of being infected. Our study therefore excludes diabetic patients hospitalized in other centers. Nevertheless, the MINSA provides health care for individuals insured by the *Sistema Integral de Salud (SIS)* which covers approximately 65% of the population (Organisation for Economic Co-operation and Development, 2017).

## 6 Conclusion

In trying to buffer the impact of COVID-19 on the health system, the government ignored the needs of those most vulnerable patients with NCDs. Having identified these deficiencies can show stakeholders the true cost of their inaction toward changing the status quo. Worldwide, the COVID-19 pandemic has thwarted the achievement of the sustainable development goals and highlighted the shortcomings that exist in facing NCDs. They should now be considered an integral part of the response strategy for COVID-19 and other large-scale catastrophes.

## Author contributions

AHV had the research idea, designed the study, collected, and processed the data. AHV, CRR, JEGU analyzed the data. All authors participated in the interpretation of the data, drafting of the manuscript and approved the final version.

## 8 Sources of financing

This research did not receive any specific grant from funding agencies in the public, commercial, or not-for-profit sectors.

## Declaration of Competing Interest

The authors declare that they have no known competing financial interests or personal relationships that could have appeared to influence the work reported in this paper.
