# Supplementary material for: Multimorbidity analysis and hospitalizations for diabetes before and after lockdown due to the COVID-19 pandemic in Peru
Source: Prev Med Rep. 2022 Jul 4;28:101884. doi: 10.1016/j.pmedr.2022.101884 (PMC9251897; doi:10.1016/j.pmedr.2022.101884)
Supplement: Supplementary Data 1 [file mmc1.docx]

Supplementary Material

All pairwise for multimorbidity network analysis in hospital admissions due to diabetes

### More frequent diagnoses

## Selecting by n

## CIE10 Frequency
## 1 DIA 70388
## 2 I10X 11312
## 3 N390 9651
## 4 N189 3178
## 5 J189 2889
## 6 U071 2827
## 7 J960 1762
## 8 D649 1563
## 9 A419 1474
## 10 L031 1172
## 11 E669 885
## 12 I500 850
## 13 J969 833
## 14 N185 820
## 15 N10X 794
## 16 E162 706
## 17 I64X 688
## 18 D509 598
## 19 E039 529
## 20 K922 527
## 21 N40X 505
## 22 B972 498
## 23 I509 494
## 24 K746 477
## 25 J159 463

### More frequent co-occurrence of diagnoses

## General

## Diag1 Diag2 Frequency
## 1 I10X DIA 11577
## 2 N390 DIA 9934
## 3 N189 DIA 3292
## 4 J189 DIA 2938
## 5 U071 DIA 2858
## 6 J960 DIA 1784
## 7 D649 DIA 1630
## 8 A419 DIA 1535
## 9 L031 DIA 1228
## 10 E669 DIA 915
## 11 I500 DIA 869
## 12 N185 DIA 842
## 13 J969 DIA 837
## 14 N10X DIA 829
## 15 E162 DIA 729
## 16 I64X DIA 698
## 17 D509 DIA 615
## 18 E039 DIA 547
## 19 K922 DIA 538
## 20 N40X DIA 524
## 21 B972 DIA 500
## 22 I509 DIA 500
## 23 K746 DIA 485
## 24 J159 DIA 481
## 25 I219 DIA 462
## 26 I639 DIA 437
## 27 I678 DIA 420
## 28 R739 DIA 417
## 29 L039 DIA 413
## 30 A090 DIA 382
## 31 N110 DIA 380
## 32 A099 DIA 372
## 33 J129 DIA 366
## 34 A150 DIA 360
## 35 E86X DIA 330
## 36 R104 DIA 330
## 37 N12X DIA 326
## 38 N179 DIA 312
## 39 N039 DIA 292
## 40 J128 DIA 287
## 41 S913 DIA 286
## 42 A560 DIA 284
## 43 I679 DIA 276
## 44 J90X DIA 252
## 45 K859 DIA 247
## 46 K802 DIA 243
## 47 J849 DIA 241
## 48 K811 DIA 241
## 49 J841 DIA 237
## 50 N200 DIA 231
## 51 N19X DIA 224
## 52 K703 DIA 218
## 53 I739 DIA 208
## 54 G934 DIA 200
## 55 L97X DIA 199
## 56 L030 DIA 190
## 57 I119 DIA 188
## 58 J459 DIA 187
## 59 G409 DIA 184
## 60 A418 DIA 178
## 61 I619 DIA 174
## 62 I489 DIA 173
## 63 J80X DIA 167
## 64 D539 DIA 165
## 65 K810 DIA 162
## 66 D500 DIA 159
## 67 I159 DIA 158
## 68 I694 DIA 154
## 69 U072 DIA 153
## 70 K297 DIA 152
## 71 R509 DIA 151
## 72 L024 DIA 149
## 73 J961 DIA 147
## 74 L038 DIA 147
## 75 E660 DIA 145
## 76 R568 DIA 141
## 77 L023 DIA 137
## 78 M869 DIA 134
## 79 J209 DIA 130
## 80 K805 DIA 130
## 81 K801 DIA 129
## 82 N819 DIA 128
## 83 M069 DIA 126
## 84 S984 DIA 126
## 85 J690 DIA 125
## 86 R02X DIA 124
## 87 T136 DIA 124
## 88 J180 DIA 123
## 89 I480 DIA 122
## 90 A162 DIA 118
## 91 K850 DIA 118
## 92 R11X DIA 118
## 93 E668 DIA 116
## 94 N180 DIA 116
## 95 I872 DIA 113
## 96 L029 DIA 113
## 97 E160 DIA 110
## 98 E872 DIA 110
## 99 N181 DIA 108
## 100 G459 DIA 107
## 101 E46X DIA 106
## 102 L899 DIA 106
## 103 N184 DIA 105
## 104 E785 DIA 104
## 105 S889 DIA 103
## 106 K800 DIA 102
## 107 I110 DIA 101
## 108 L032 DIA 101
## 109 I259 DIA 99
## 110 A169 DIA 98
## 111 K610 DIA 97
## 112 S981 DIA 97
## 113 I200 DIA 95
## 114 N399 DIA 95
## 115 N151 DIA 93
## 116 I120 DIA 92
## 117 K295 DIA 92
## 118 E780 DIA 90
## 119 J188 DIA 89
## 120 J13X DIA 88
## 121 J22X DIA 88
## 122 K429 DIA 88
## 123 K85X DIA 88
## 124 H360 DIA 87
## 125 K729 DIA 87
## 126 R572 DIA 86
## 127 E782 DIA 83
## 128 L089 DIA 83
## 129 N111 DIA 83
## 130 J120 DIA 82
## 131 N183 DIA 82
## 132 K291 DIA 81
## 133 I48X DIA 80
## 134 D648 DIA 79
## 135 K590 DIA 78
## 136 R042 DIA 78
## 137 I633 DIA 76
## 138 R18X DIA 75
## 139 I802 DIA 74
## 140 K819 DIA 73
## 141 N832 DIA 72
## 142 N813 DIA 71
## 143 A09X DIA 70
## 144 G20X DIA 70
## 145 I499 DIA 70
## 146 I610 DIA 70
## 147 L984 DIA 70
## 148 J81X DIA 69
## 149 K409 DIA 69
## 150 A159 DIA 68
## 151 A409 DIA 68
## 152 I830 DIA 67
## 153 K439 DIA 67
## 154 K566 DIA 66
## 155 L022 DIA 66
## 156 L890 DIA 66
## 157 N049 DIA 66
## 158 F209 DIA 65
## 159 R51X DIA 64
## 160 E871 DIA 63
## 161 I209 DIA 63
## 162 N319 DIA 63
## 163 F200 DIA 61
## 164 I693 DIA 61
## 165 N119 DIA 61
## 166 S069 DIA 60
## 167 F329 DIA 59
## 168 J029 DIA 59
## 169 N289 DIA 59
## 170 T814 DIA 59
## 171 I829 DIA 58
## 172 I469 DIA 57
## 173 K750 DIA 57
## 174 J158 DIA 56
## 175 S982 DIA 56
## 176 E059 DIA 55
## 177 I150 DIA 55
## 178 L021 DIA 55
## 179 N760 DIA 55
## 180 F412 DIA 54
## 181 L028 DIA 54
## 182 O829 DIA 54
## 183 K830 DIA 53
## 184 D638 DIA 52
## 185 I609 DIA 52
## 186 I634 DIA 52
## 187 J852 DIA 52
## 188 K808 DIA 52
## 189 R100 DIA 52
## 190 S819 DIA 52
## 191 K30X DIA 51
## 192 M725 DIA 51
## 193 A010 DIA 50
## 194 I859 DIA 50
## 195 N498 DIA 50
## 196 L020 DIA 49
## 197 F419 DIA 48
## 198 G590 DIA 48
## 199 J157 DIA 48
## 200 S721 DIA 48
## 201 C539 DIA 47
## 202 D693 DIA 47
## 203 F03X DIA 47
## 204 I442 DIA 47
## 205 R17X DIA 47
## 206 R609 DIA 47
## 207 I698 DIA 46
## 208 N139 DIA 46
## 209 M726 DIA 45
## 210 I792 DIA 44
## 211 N209 DIA 44
## 212 S822 DIA 44
## 213 A153 DIA 43
## 214 I630 DIA 43
## 215 L409 DIA 43
## 216 N133 DIA 43
## 217 R101 DIA 43
## 218 B86X DIA 42
## 219 C169 DIA 42
## 220 J40X DIA 42
## 221 K259 DIA 42
## 222 E161 DIA 41
## 223 E876 DIA 41
## 224 G632 DIA 41
## 225 I255 DIA 41
## 226 R571 DIA 41
## 227 E43X DIA 40
## 228 J984 DIA 40
## 229 K769 DIA 40
## 230 O821 DIA 40
## 231 C189 DIA 39
## 232 C61X DIA 39
## 233 E878 DIA 39
## 234 G610 DIA 39
## 235 G819 DIA 39
## 236 I832 DIA 39
## 237 J150 DIA 39
## 238 J168 DIA 39
## 239 J181 DIA 39
## 240 S880 DIA 39
## 241 T009 DIA 39
## 242 E870 DIA 38
## 243 H269 DIA 38
## 244 J869 DIA 38
## 245 K047 DIA 38
## 246 L033 DIA 38
## 247 N61X DIA 38
## 248 T874 DIA 38
## 249 A971 DIA 37
## 250 E038 DIA 37
## 251 L89X DIA 37
## 252 N182 DIA 37
## 253 R31X DIA 37
## 254 E440 DIA 36
## 255 E880 DIA 36
## 256 I490 DIA 36
## 257 K37X DIA 36
## 258 K851 DIA 36
## 259 S729 DIA 36
## 260 A499 DIA 35
## 261 R33X DIA 35
## 262 D696 DIA 34
## 263 G629 DIA 34
## 264 I850 DIA 34
## 265 N814 DIA 34
## 266 S911 DIA 34
## 267 F102 DIA 33
## 268 I269 DIA 33
## 269 J152 DIA 33
## 270 M109 DIA 33
## 271 R570 DIA 33
## 272 A90X DIA 32
## 273 J100 DIA 32
## 274 K122 DIA 32
## 275 A020 DIA 31
## 276 G309 DIA 31
## 277 I743 DIA 31
## 278 K290 DIA 31
## 279 N23X DIA 31
## 280 O234 DIA 31
## 281 I210 DIA 30
## 282 I340 DIA 30
## 283 I519 DIA 30
## 284 I620 DIA 30
## 285 I635 DIA 30
## 286 M139 DIA 30
## 287 N083 DIA 30
## 288 N771 DIA 30
## 289 O990 DIA 30
## 290 H819 DIA 29
## 291 M009 DIA 29
## 292 S065 DIA 29
## 293 J448 DIA 28
## 294 R579 DIA 28
## 295 S681 DIA 28
## 296 T887 DIA 28
## 297 E781 DIA 27
## 298 G909 DIA 27
## 299 I612 DIA 27
## 300 I828 DIA 27
## 301 I959 DIA 27
## 302 J46X DIA 27
## 303 N459 DIA 27
## 304 A156 DIA 26
## 305 A410 DIA 26
## 306 C250 DIA 26
## 307 C56X DIA 26
## 308 E249 DIA 26
## 309 G510 DIA 26
## 310 G969 DIA 26
## 311 H050 DIA 26
## 312 I249 DIA 26
## 313 I771 DIA 26
## 314 K250 DIA 26
## 315 K920 DIA 26
## 316 N812 DIA 26
## 317 R418 DIA 26
## 318 T141 DIA 26
## 319 C229 DIA 25
## 320 D531 DIA 25
## 321 E889 DIA 25
## 322 G442 DIA 25
## 323 I151 DIA 25
## 324 I629 DIA 25
## 325 K279 DIA 25
## 326 K296 DIA 25
## 327 K591 DIA 25
## 328 M179 DIA 25
## 329 N492 DIA 25
## 330 O034 DIA 25
## 331 T793 DIA 25
## 332 C220 DIA 24
## 333 I158 DIA 24
## 334 J068 DIA 24
## 335 J440 DIA 24
## 336 J980 DIA 24
## 337 K564 DIA 24
## 338 N178 DIA 24
## 339 O342 DIA 24
## 340 S828 DIA 24
## 341 S912 DIA 24
## 342 S980 DIA 24
## 343 E835 DIA 23
## 344 F321 DIA 23
## 345 H813 DIA 23
## 346 J069 DIA 23
## 347 J156 DIA 23
## 348 J698 DIA 23
## 349 J91X DIA 23
## 350 K469 DIA 23
## 351 K650 DIA 23
## 352 N172 DIA 23
## 353 N312 DIA 23
## 354 O809 DIA 23
## 355 R32X DIA 23
## 356 B379 DIA 22
## 357 B829 DIA 22
## 358 D487 DIA 22
## 359 E441 DIA 22
## 360 G049 DIA 22
## 361 I809 DIA 22
## 362 K745 DIA 22
## 363 N300 DIA 22
## 364 B370 DIA 21
## 365 D410 DIA 21
## 366 H280 DIA 21
## 367 I702 DIA 21
## 368 M059 DIA 21
## 369 N310 DIA 21
## 370 O064 DIA 21
## 371 O149 DIA 21
## 372 A400 DIA 20
## 373 G408 DIA 20
## 374 H540 DIA 20
## 375 I839 DIA 20
## 376 L509 DIA 20
## 377 L88X DIA 20
## 378 N938 DIA 20
## 379 R688 DIA 20
## 380 S789 DIA 20
## 381 S818 DIA 20
## 382 T147 DIA 20
## 383 C259 DIA 19
## 384 C900 DIA 19
## 385 H409 DIA 19
## 386 I251 DIA 19
## 387 I618 DIA 19
## 388 J42X DIA 19
## 389 J851 DIA 19
## 390 K359 DIA 19
## 391 K760 DIA 19
## 392 L80X DIA 19
## 393 N170 DIA 19
## 394 N499 DIA 19
## 395 R001 DIA 19
## 396 R601 DIA 19
## 397 A170 DIA 18
## 398 H669 DIA 18
## 399 I443 DIA 18
## 400 I471 DIA 18
## 401 J939 DIA 18
## 402 K269 DIA 18
## 403 L088 DIA 18
## 404 L400 DIA 18
## 405 M544 DIA 18
## 406 N040 DIA 18
## 407 N220 DIA 18
## 408 O820 DIA 18
## 409 R770 DIA 18
## 410 S917 DIA 18
## 411 T252 DIA 18
## 412 Y835 DIA 18
## 413 B378 DIA 17
## 414 B465 DIA 17
## 415 E875 DIA 17
## 416 F011 DIA 17
## 417 F019 DIA 17
## 418 G255 DIA 17
## 419 H46X DIA 17
## 420 H811 DIA 17
## 421 H82X DIA 17
## 422 I252 DIA 17
## 423 I516 DIA 17
## 424 I611 DIA 17
## 425 I632 DIA 17
## 426 I749 DIA 17
## 427 J123 DIA 17
## 428 J219 DIA 17
## 429 J450 DIA 17
## 430 K420 DIA 17
## 431 K659 DIA 17
## 432 L309 DIA 17
## 433 L893 DIA 17
## 434 N188 DIA 17
## 435 S619 DIA 17
## 436 T131 DIA 17
## 437 B461 DIA 16
## 438 C910 DIA 16
## 439 E169 DIA 16
## 440 G990 DIA 16
## 441 I420 DIA 16
## 442 K20X DIA 16
## 443 K929 DIA 16
## 444 N130 DIA 16
## 445 N159 DIA 16
## 446 N309 DIA 16
## 447 O141 DIA 16
## 448 R072 DIA 16
## 449 T633 DIA 16
## 450 T813 DIA 16
## 451 B009 DIA 15
## 452 B354 DIA 15
## 453 D376 DIA 15
## 454 I129 DIA 15
## 455 I152 DIA 15
## 456 I350 DIA 15
## 457 J151 DIA 15
## 458 J631 DIA 15
## 459 S723 DIA 15
## 460 T054 DIA 15
## 461 B351 DIA 14
## 462 C221 DIA 14
## 463 E220 DIA 14
## 464 E271 DIA 14
## 465 G042 DIA 14
## 466 G400 DIA 14
## 467 G403 DIA 14
## 468 G919 DIA 14
## 469 I250 DIA 14
## 470 I803 DIA 14
## 471 J848 DIA 14
## 472 K318 DIA 14
## 473 K351 DIA 14
## 474 K352 DIA 14
## 475 K402 DIA 14
## 476 K660 DIA 14
## 477 K803 DIA 14
## 478 K839 DIA 14
## 479 N219 DIA 14
## 480 N47X DIA 14
## 481 N818 DIA 14
## 482 O200 DIA 14
## 483 R42X DIA 14
## 484 R53X DIA 14
## 485 R55X DIA 14
## 486 S820 DIA 14
## 487 S983 DIA 14
## 488 B028 DIA 13
## 489 B353 DIA 13
## 490 D391 DIA 13
## 491 G589 DIA 13
## 492 G932 DIA 13
## 493 H259 DIA 13
## 494 I258 DIA 13
## 495 I613 DIA 13
## 496 I674 DIA 13
## 497 J850 DIA 13
## 498 J948 DIA 13
## 499 K260 DIA 13
## 500 K632 DIA 13
## 501 K928 DIA 13
## 502 L080 DIA 13
## 503 M866 DIA 13
## 504 N450 DIA 13
## 505 O364 DIA 13
## 506 O800 DIA 13
## 507 R578 DIA 13
## 508 S682 DIA 13
## 509 T302 DIA 13
## 510 Z488 DIA 13
## 511 D609 DIA 12
## 512 E230 DIA 12
## 513 F410 DIA 12
## 514 H919 DIA 12
## 515 I130 DIA 12
## 516 J039 DIA 12
## 517 K046 DIA 12
## 518 K353 DIA 12
## 519 K719 DIA 12
## 520 K721 DIA 12
## 521 K759 DIA 12
## 522 M321 DIA 12
## 523 M600 DIA 12
## 524 N210 DIA 12
## 525 N281 DIA 12
## 526 N328 DIA 12
## 527 N398 DIA 12
## 528 N872 DIA 12
## 529 R64X DIA 12
## 530 S610 DIA 12
## 531 S781 DIA 12
## 532 T600 DIA 12
## 533 T888 DIA 12
## 534 A480 DIA 11
## 535 E041 DIA 11
## 536 E050 DIA 11
## 537 F009 DIA 11
## 538 F449 DIA 11
## 539 G401 DIA 11
## 540 I059 DIA 11
## 541 I48 DIA 11
## 542 I481 DIA 11
## 543 I600 DIA 11
## 544 K219 DIA 11
## 545 K294 DIA 11
## 546 K717 DIA 11
## 547 K768 DIA 11
## 548 K804 DIA 11
## 549 L892 DIA 11
## 550 M729 DIA 11
## 551 M868 DIA 11
## 552 N009 DIA 11
## 553 N202 DIA 11
## 554 N709 DIA 11
## 555 N840 DIA 11
## 556 R000 DIA 11
## 557 S223 DIA 11
## 558 S328 DIA 11
## 559 S724 DIA 11
## 560 T140 DIA 11
## 561 W199 DIA 11
## 562 43552 DIA 10
## 563 D069 DIA 10
## 564 E02X DIA 10
## 565 E240 DIA 10
## 566 F100 DIA 10
## 567 G060 DIA 10
## 568 G619 DIA 10
## 569 I211 DIA 10
## 570 I479 DIA 10
## 571 I64 DIA 10
## 572 I775 DIA 10
## 573 I800 DIA 10
## 574 I99X DIA 10
## 575 J36X DIA 10
## 576 J410 DIA 10
## 577 J949 DIA 10
## 578 K221 DIA 10
## 579 K270 DIA 10
## 580 K579 DIA 10
## 581 K740 DIA 10
## 582 L891 DIA 10
## 583 M100 DIA 10
## 584 M169 DIA 10
## 585 M431 DIA 10
## 586 M490 DIA 10
## 587 M512 DIA 10
## 588 M793 DIA 10
## 589 N394 DIA 10
## 590 N481 DIA 10
## 591 N830 DIA 10
## 592 R074 DIA 10
## 593 R560 DIA 10
## 594 S411 DIA 10
## 595 S422 DIA 10
## 596 S810 DIA 10
## 597 T824 DIA 10
## 598 43553 DIA 9
## 599 A279 DIA 9
## 600 E874 DIA 9
## 601 F050 DIA 9
## 602 F059 DIA 9
## 603 G009 DIA 9
## 604 G402 DIA 9
## 605 G936 DIA 9
## 606 H650 DIA 9
## 607 I060 DIA 9
## 608 I213 DIA 9
## 609 I270 DIA 9
## 610 I460 DIA 9
## 611 I631 DIA 9
## 612 I776 DIA 9
## 613 J019 DIA 9
## 614 K319 DIA 9
## 615 K350 DIA 9
## 616 K565 DIA 9
## 617 K612 DIA 9
## 618 K625 DIA 9
## 619 K630 DIA 9
## 620 K658 DIA 9
## 621 K720 DIA 9
## 622 K858 DIA 9
## 623 M255 DIA 9
## 624 M549 DIA 9
## 625 M819 DIA 9
## 626 M861 DIA 9
## 627 N000 DIA 9
## 628 N059 DIA 9
## 629 N971 DIA 9
## 630 O210 DIA 9
## 631 R392 DIA 9
## 632 R410 DIA 9
## 633 R54X DIA 9
## 634 R69X DIA 9
## 635 S062 DIA 9
## 636 S202 DIA 9
## 637 S525 DIA 9
## 638 S817 DIA 9
## 639 S920 DIA 9
## 640 T243 DIA 9
## 641 Z519 DIA 9
## 642 A064 DIA 8
## 643 D589 DIA 8
## 644 E242 DIA 8
## 645 E784 DIA 8
## 646 F130 DIA 8
## 647 F320 DIA 8
## 648 F328 DIA 8
## 649 F411 DIA 8
## 650 F432 DIA 8
## 651 G360 DIA 8
## 652 G410 DIA 8
## 653 G569 DIA 8
## 654 G92X DIA 8
## 655 G931 DIA 8
## 656 G939 DIA 8
## 657 G959 DIA 8
## 658 H660 DIA 8
## 659 I051 DIA 8
## 660 I429 DIA 8
## 661 I440 DIA 8
## 662 I459 DIA 8
## 663 I498 DIA 8
## 664 I770 DIA 8
## 665 I801 DIA 8
## 666 J040 DIA 8
## 667 J14X DIA 8
## 668 J938 DIA 8
## 669 J988 DIA 8
## 670 K102 DIA 8
## 671 K210 DIA 8
## 672 K648 DIA 8
## 673 K861 DIA 8
## 674 L500 DIA 8
## 675 L959 DIA 8
## 676 M331 DIA 8
## 677 M541 DIA 8
## 678 M809 DIA 8
## 679 N029 DIA 8
## 680 N158 DIA 8
## 681 N411 DIA 8
## 682 N433 DIA 8
## 683 N751 DIA 8
## 684 N810 DIA 8
## 685 N870 DIA 8
## 686 N879 DIA 8
## 687 N911 DIA 8
## 688 N952 DIA 8
## 689 O140 DIA 8
## 690 O230 DIA 8
## 691 O249 DIA 8
## 692 R092 DIA 8
## 693 S010 DIA 8
## 694 S099 DIA 8
## 695 S910 DIA 8
## 696 T242 DIA 8
## 697 T827 DIA 8
## 698 T857 DIA 8
## 699 43564 DIA 7
## 700 43566 DIA 7
## 701 C329 DIA 7
## 702 D590 DIA 7
## 703 E049 DIA 7
## 704 E538 DIA 7
## 705 E873 DIA 7
## 706 F064 DIA 7
## 707 F067 DIA 7
## 708 F09X DIA 7
## 709 F341 DIA 7
## 710 G039 DIA 7
## 711 G041 DIA 7
## 712 G373 DIA 7
## 713 G628 DIA 7
## 714 H350 DIA 7
## 715 I229 DIA 7
## 716 I441 DIA 7
## 717 I447 DIA 7
## 718 I671 DIA 7
## 719 I688 DIA 7
## 720 I709 DIA 7
## 721 J441 DIA 7
## 722 J680 DIA 7
## 723 J930 DIA 7
## 724 J942 DIA 7
## 725 K299 DIA 7
## 726 K449 DIA 7
## 727 K567 DIA 7
## 728 K766 DIA 7
## 729 K828 DIA 7
## 730 K829 DIA 7
## 731 L100 DIA 7
## 732 L719 DIA 7
## 733 L989 DIA 7
## 734 M053 DIA 7
## 735 M131 DIA 7
## 736 M150 DIA 7
## 737 M479 DIA 7
## 738 M609 DIA 7
## 739 N132 DIA 7
## 740 N288 DIA 7
## 741 N320 DIA 7
## 742 N359 DIA 7
## 743 N809 DIA 7
## 744 N816 DIA 7
## 745 O020 DIA 7
## 746 O689 DIA 7
## 747 Q667 DIA 7
## 748 R030 DIA 7
## 749 R160 DIA 7
## 750 R190 DIA 7
## 751 R402 DIA 7
## 752 R529 DIA 7
## 753 R58X DIA 7
## 754 R600 DIA 7
## 755 T253 DIA 7
## 756 43549 DIA 6
## 757 43556 DIA 6
## 758 43565 DIA 6
## 759 B022 DIA 6
## 760 D414 DIA 6
## 761 F069 DIA 6
## 762 F323 DIA 6
## 763 F339 DIA 6
## 764 F448 DIA 6
## 765 G219 DIA 6
## 766 G439 DIA 6
## 767 H601 DIA 6
## 768 H609 DIA 6
## 769 H659 DIA 6
## 770 I517 DIA 6
## 771 I601 DIA 6
## 772 I691 DIA 6
## 773 I729 DIA 6
## 774 I831 DIA 6
## 775 I891 DIA 6
## 776 J398 DIA 6
## 777 K113 DIA 6
## 778 K257 DIA 6
## 779 K605 DIA 6
## 780 K661 DIA 6
## 781 K700 DIA 6
## 782 K85 DIA 6
## 783 K863 DIA 6
## 784 M480 DIA 6
## 785 M860 DIA 6
## 786 M879 DIA 6
## 787 M939 DIA 6
## 788 N048 DIA 6
## 789 N131 DIA 6
## 790 N201 DIA 6
## 791 N322 DIA 6
## 792 N391 DIA 6
## 793 N738 DIA 6
## 794 N820 DIA 6
## 795 O021 DIA 6
## 796 O429 DIA 6
## 797 O730 DIA 6
## 798 O758 DIA 6
## 799 R520 DIA 6
## 800 S009 DIA 6
## 801 S311 DIA 6
## 802 S399 DIA 6
## 803 S701 DIA 6
## 804 S711 DIA 6
## 805 S821 DIA 6
## 806 S826 DIA 6
## 807 S829 DIA 6
## 808 T059 DIA 6
## 809 T07X DIA 6
## 810 T630 DIA 6
## 811 T856 DIA 6
## 812 43558 DIA 5
## 813 43571 DIA 5
## 814 E222 DIA 5
## 815 E272 DIA 5
## 816 E756 DIA 5
## 817 F058 DIA 5
## 818 F101 DIA 5
## 819 F103 DIA 5
## 820 F109 DIA 5
## 821 F445 DIA 5
## 822 G048 DIA 5
## 823 G311 DIA 5
## 824 G530 DIA 5
## 825 G700 DIA 5
## 826 G938 DIA 5
## 827 G952 DIA 5
## 828 G98X DIA 5
## 829 H043 DIA 5
## 830 H059 DIA 5
## 831 H109 DIA 5
## 832 H400 DIA 5
## 833 H588 DIA 5
## 834 H663 DIA 5
## 835 H810 DIA 5
## 836 H900 DIA 5
## 837 H903 DIA 5
## 838 I050 DIA 5
## 839 I131 DIA 5
## 840 I139 DIA 5
## 841 I260 DIA 5
## 842 I309 DIA 5
## 843 I330 DIA 5
## 844 I461 DIA 5
## 845 I482 DIA 5
## 846 I501 DIA 5
## 847 I614 DIA 5
## 848 I660 DIA 5
## 849 I700 DIA 5
## 850 I738 DIA 5
## 851 I742 DIA 5
## 852 I744 DIA 5
## 853 I779 DIA 5
## 854 I821 DIA 5
## 855 I822 DIA 5
## 856 I864 DIA 5
## 857 J160 DIA 5
## 858 J342 DIA 5
## 859 J679 DIA 5
## 860 J840 DIA 5
## 861 J853 DIA 5
## 862 K253 DIA 5
## 863 K273 DIA 5
## 864 K293 DIA 5
## 865 K298 DIA 5
## 866 K36X DIA 5
## 867 K421 DIA 5
## 868 K623 DIA 5
## 869 K649 DIA 5
## 870 K710 DIA 5
## 871 K739 DIA 5
## 872 K743 DIA 5
## 873 K818 DIA 5
## 874 K868 DIA 5
## 875 L259 DIA 5
## 876 L304 DIA 5
## 877 L539 DIA 5
## 878 L89 DIA 5
## 879 M146 DIA 5
## 880 M300 DIA 5
## 881 M430 DIA 5
## 882 M705 DIA 5
## 883 M842 DIA 5
## 884 N050 DIA 5
## 885 N118 DIA 5
## 886 N340 DIA 5
## 887 N512 DIA 5
## 888 N736 DIA 5
## 889 N800 DIA 5
## 890 O069 DIA 5
## 891 O600 DIA 5
## 892 R221 DIA 5
## 893 R99X DIA 5
## 894 S064 DIA 5
## 895 S066 DIA 5
## 896 S271 DIA 5
## 897 S310 DIA 5
## 898 S320 DIA 5
## 899 S324 DIA 5
## 900 S520 DIA 5
## 901 S611 DIA 5
## 902 S700 DIA 5
## 903 S718 DIA 5
## 904 S722 DIA 5
## 905 S823 DIA 5
## 906 T019 DIA 5
## 907 T10X DIA 5
## 908 T149 DIA 5
## 909 T634 DIA 5
## 910 T659 DIA 5
## 911 T702 DIA 5
## 912 Z896 DIA 5
## 913 43540 DIA 4
## 914 43547 DIA 4
## 915 43554 DIA 4
## 916 43559 DIA 4
## 917 43577 DIA 4
## 918 43580 DIA 4
## 919 A521 DIA 4
## 920 D329 DIA 4
## 921 E031 DIA 4
## 922 E559 DIA 4
## 923 E700 DIA 4
## 924 E739 DIA 4
## 925 E755 DIA 4
## 926 E831 DIA 4
## 927 F172 DIA 4
## 928 F192 DIA 4
## 929 F250 DIA 4
## 930 F29X DIA 4
## 931 F319 DIA 4
## 932 F609 DIA 4
## 933 G01X DIA 4
## 934 G050 DIA 4
## 935 G08X DIA 4
## 936 G35X DIA 4
## 937 G458 DIA 4
## 938 G464 DIA 4
## 939 G500 DIA 4
## 940 G579 DIA 4
## 941 G588 DIA 4
## 942 G618 DIA 4
## 943 G822 DIA 4
## 944 G911 DIA 4
## 945 H024 DIA 4
## 946 H268 DIA 4
## 947 H368 DIA 4
## 948 H490 DIA 4
## 949 H544 DIA 4
## 950 H654 DIA 4
## 951 H664 DIA 4
## 952 H701 DIA 4
## 953 I071 DIA 4
## 954 I212 DIA 4
## 955 I248 DIA 4
## 956 I278 DIA 4
## 957 I451 DIA 4
## 958 I495 DIA 4
## 959 I615 DIA 4
## 960 I670 DIA 4
## 961 I690 DIA 4
## 962 I778 DIA 4
## 963 I842 DIA 4
## 964 I868 DIA 4
## 965 J101 DIA 4
## 966 J139 DIA 4
## 967 J170 DIA 4
## 968 J178 DIA 4
## 969 J386 DIA 4
## 970 J390 DIA 4
## 971 J61X DIA 4
## 972 K029 DIA 4
## 973 k259 DIA 4
## 974 K602 DIA 4
## 975 K631 DIA 4
## 976 K704 DIA 4
## 977 K744 DIA 4
## 978 K823 DIA 4
## 979 K831 DIA 4
## 980 K860 DIA 4
## 981 L00X DIA 4
## 982 L043 DIA 4
## 983 L102 DIA 4
## 984 L209 DIA 4
## 985 L219 DIA 4
## 986 L281 DIA 4
## 987 L401 DIA 4
## 988 L405 DIA 4
## 989 L408 DIA 4
## 990 L511 DIA 4
## 991 L729 DIA 4
## 992 M029 DIA 4
## 993 M130 DIA 4
## 994 M542 DIA 4
## 995 M728 DIA 4
## 996 M779 DIA 4
## 997 M870 DIA 4
## 998 M878 DIA 4
## 999 M932 DIA 4
## 1000 N211 DIA 4
## 1001 N311 DIA 4
## 1002 N321 DIA 4
## 1003 N63X DIA 4
## 1004 N72X DIA 4
## 1005 N871 DIA 4
## 1006 O335 DIA 4
## 1007 O40X DIA 4
## 1008 O479 DIA 4
## 1009 O828 DIA 4
## 1010 O860 DIA 4
## 1011 Q249 DIA 4
## 1012 Q610 DIA 4
## 1013 Q909 DIA 4
## 1014 R060 DIA 4
## 1015 R13X DIA 4
## 1016 R162 DIA 4
## 1017 R229 DIA 4
## 1018 R960 DIA 4
## 1019 S008 DIA 4
## 1020 S019 DIA 4
## 1021 S024 DIA 4
## 1022 S424 DIA 4
## 1023 S430 DIA 4
## 1024 S618 DIA 4
## 1025 S626 DIA 4
## 1026 S628 DIA 4
## 1027 S683 DIA 4
## 1028 S728 DIA 4
## 1029 S824 DIA 4
## 1030 S925 DIA 4
## 1031 S929 DIA 4
## 1032 T303 DIA 4
## 1033 T424 DIA 4
## 1034 T477 DIA 4
## 1035 T519 DIA 4
## 1036 T825 DIA 4
## 1037 T905 DIA 4
## 1038 W019 DIA 4
## 1039 Y919 DIA 4
## 1040 Z359 DIA 4
## 1041 43519 DIA 3
## 1042 43550 DIA 3
## 1043 43560 DIA 3
## 1044 43561 DIA 3
## 1045 43563 DIA 3
## 1046 43570 DIA 3
## 1047 43572 DIA 3
## 1048 A083 DIA 3
## 1049 C166 DIA 3
## 1050 C959 DIA 3
## 1051 D151 DIA 3
## 1052 D591 DIA 3
## 1053 E030 DIA 3
## 1054 E034 DIA 3
## 1055 E042 DIA 3
## 1056 E052 DIA 3
## 1057 E069 DIA 3
## 1058 E15X DIA 3
## 1059 E209 DIA 3
## 1060 E210 DIA 3
## 1061 E229 DIA 3
## 1062 E232 DIA 3
## 1063 E236 DIA 3
## 1064 E270 DIA 3
## 1065 E274 DIA 3
## 1066 E512 DIA 3
## 1067 E722 DIA 3
## 1068 E788 DIA 3
## 1069 E888 DIA 3
## 1070 F010 DIA 3
## 1071 F018 DIA 3
## 1072 F062 DIA 3
## 1073 F078 DIA 3
## 1074 F142 DIA 3
## 1075 F238 DIA 3
## 1076 F413 DIA 3
## 1077 F450 DIA 3
## 1078 F799 DIA 3
## 1079 G020 DIA 3
## 1080 G040 DIA 3
## 1081 G062 DIA 3
## 1082 G09X DIA 3
## 1083 G10X DIA 3
## 1084 G122 DIA 3
## 1085 G310 DIA 3
## 1086 G419 DIA 3
## 1087 G430 DIA 3
## 1088 G448 DIA 3
## 1089 G450 DIA 3
## 1090 G531 DIA 3
## 1091 G578 DIA 3
## 1092 G609 DIA 3
## 1093 G633 DIA 3
## 1094 G729 DIA 3
## 1095 G825 DIA 3
## 1096 H010 DIA 3
## 1097 H103 DIA 3
## 1098 H110 DIA 3
## 1099 H352 DIA 3
## 1100 H358 DIA 3
## 1101 H440 DIA 3
## 1102 H527 DIA 3
## 1103 H549 DIA 3
## 1104 H602 DIA 3
## 1105 H651 DIA 3
## 1106 H653 DIA 3
## 1107 I061 DIA 3
## 1108 I069 DIA 3
## 1109 I089 DIA 3
## 1110 I132 DIA 3
## 1111 I241 DIA 3
## 1112 I339 DIA 3
## 1113 I358 DIA 3
## 1114 I378 DIA 3
## 1115 I38X DIA 3
## 1116 I390 DIA 3
## 1117 I421 DIA 3
## 1118 I446 DIA 3
## 1119 I510 DIA 3
## 1120 I528 DIA 3
## 1121 I602 DIA 3
## 1122 I606 DIA 3
## 1123 I728 DIA 3
## 1124 I748 DIA 3
## 1125 I871 DIA 3
## 1126 I879 DIA 3
## 1127 I890 DIA 3
## 1128 I899 DIA 3
## 1129 I982 DIA 3
## 1130 J014 DIA 3
## 1131 J15 E11 3
## 1132 J154 DIA 3
## 1133 J155 DIA 3
## 1134 J200 DIA 3
## 1135 J320 DIA 3
## 1136 J340 DIA 3
## 1137 J380 DIA 3
## 1138 J393 DIA 3
## 1139 J451 DIA 3
## 1140 J64X DIA 3
## 1141 J941 DIA 3
## 1142 J998 DIA 3
## 1143 K041 DIA 3
## 1144 K044 DIA 3
## 1145 K052 DIA 3
## 1146 K228 DIA 3
## 1147 K254 DIA 3
## 1148 K316 DIA 3
## 1149 K317 DIA 3
## 1150 K388 DIA 3
## 1151 K440 DIA 3
## 1152 K458 DIA 3
## 1153 K461 DIA 3
## 1154 K519 DIA 3
## 1155 K550 DIA 3
## 1156 K559 DIA 3
## 1157 K570 DIA 3
## 1158 K593 DIA 3
## 1159 K626 DIA 3
## 1160 K628 DIA 3
## 1161 K639 DIA 3
## 1162 K701 DIA 3
## 1163 K702 DIA 3
## 1164 K712 DIA 3
## 1165 K758 DIA 3
## 1166 K820 DIA 3
## 1167 K833 DIA 3
## 1168 K838 DIA 3
## 1169 K862 DIA 3
## 1170 K869 DIA 3
## 1171 K904 DIA 3
## 1172 K914 DIA 3
## 1173 K915 DIA 3
## 1174 K921 DIA 3
## 1175 L010 DIA 3
## 1176 L048 DIA 3
## 1177 L239 DIA 3
## 1178 L402 DIA 3
## 1179 L600 DIA 3
## 1180 L930 DIA 3
## 1181 L988 DIA 3
## 1182 M050 DIA 3
## 1183 M068 DIA 3
## 1184 M073 DIA 3
## 1185 M125 DIA 3
## 1186 M154 DIA 3
## 1187 M171 DIA 3
## 1188 M189 DIA 3
## 1189 M254 DIA 3
## 1190 M311 DIA 3
## 1191 M313 DIA 3
## 1192 M465 DIA 3
## 1193 M478 DIA 3
## 1194 M489 DIA 3
## 1195 M511 DIA 3
## 1196 M543 DIA 3
## 1197 M620 DIA 3
## 1198 M623 DIA 3
## 1199 M624 DIA 3
## 1200 M659 DIA 3
## 1201 M712 DIA 3
## 1202 M720 DIA 3
## 1203 M790 DIA 3
## 1204 M795 DIA 3
## 1205 M844 DIA 3
## 1206 M863 DIA 3
## 1207 M864 DIA 3
## 1208 M900 DIA 3
## 1209 N020 DIA 3
## 1210 N038 DIA 3
## 1211 N079 DIA 3
## 1212 N10 DIA 3
## 1213 N137 DIA 3
## 1214 N138 DIA 3
## 1215 N144 DIA 3
## 1216 N258 DIA 3
## 1217 N302 DIA 3
## 1218 N350 DIA 3
## 1219 N44X DIA 3
## 1220 N482 DIA 3
## 1221 N700 DIA 3
## 1222 N701 DIA 3
## 1223 N710 DIA 3
## 1224 N750 DIA 3
## 1225 N758 DIA 3
## 1226 N761 DIA 3
## 1227 N778 DIA 3
## 1228 N835 DIA 3
## 1229 N841 DIA 3
## 1230 N851 DIA 3
## 1231 N859 DIA 3
## 1232 N898 DIA 3
## 1233 N930 DIA 3
## 1234 N990 DIA 3
## 1235 N994 DIA 3
## 1236 O009 DIA 3
## 1237 O100 DIA 3
## 1238 O13X DIA 3
## 1239 O16X DIA 3
## 1240 O268 DIA 3
## 1241 O367 DIA 3
## 1242 O410 DIA 3
## 1243 O441 DIA 3
## 1244 O649 DIA 3
## 1245 O669 DIA 3
## 1246 O839 DIA 3
## 1247 O912 DIA 3
## 1248 O992 DIA 3
## 1249 Q250 DIA 3
## 1250 Q501 DIA 3
## 1251 R049 DIA 3
## 1252 R05X DIA 3
## 1253 R070 DIA 3
## 1254 R098 DIA 3
## 1255 R222 DIA 3
## 1256 R488 DIA 3
## 1257 R522 DIA 3
## 1258 R590 DIA 3
## 1259 R634 DIA 3
## 1260 R650 DIA 3
## 1261 R651 DIA 3
## 1262 R730 DIA 3
## 1263 R828 DIA 3
## 1264 S109 DIA 3
## 1265 S210 DIA 3
## 1266 S224 DIA 3
## 1267 S299 DIA 3
## 1268 S300 DIA 3
## 1269 S301 DIA 3
## 1270 S313 DIA 3
## 1271 S321 DIA 3
## 1272 S369 DIA 3
## 1273 S370 DIA 3
## 1274 S420 DIA 3
## 1275 S518 DIA 3
## 1276 S522 DIA 3
## 1277 S730 DIA 3
## 1278 S780 DIA 3
## 1279 T116 DIA 3
## 1280 T143 DIA 3
## 1281 T240 DIA 3
## 1282 T250 DIA 3
## 1283 T301 DIA 3
## 1284 T310 DIA 3
## 1285 T509 DIA 3
## 1286 T543 DIA 3
## 1287 T740 DIA 3
## 1288 T784 DIA 3
## 1289 T983 DIA 3
## 1290 U069 DIA 3
## 1291 W179 DIA 3
## 1292 Z100 DIA 3
## 1293 Z540 DIA 3
## 1294 Z608 DIA 3
## 1295 Z950 DIA 3
## 1296 43498 DIA 2
## 1297 43501 I10X 2
## 1298 43509 DIA 2
## 1299 43525 S889 2
## 1300 43526 I509 2
## 1301 43530 I10X 2
## 1302 43541 I10X 2
## 1303 43551 N390 2
## 1304 43555 DIA 2
## 1305 43562 I10X 2
## 1306 43567 DIA 2
## 1307 43568 E86X 2
## 1308 43569 N390 2
## 1309 43574 DIA 2
## 1310 43579 DIA 2
## 1311 A58X DIA 2
## 1312 A599 DIA 2
## 1313 A852 DIA 2
## 1314 B878 DIA 2
## 1315 C258 DIA 2
## 1316 C310 DIA 2
## 1317 C348 DIA 2
## 1318 C540 DIA 2
## 1319 C840 DIA 2
## 1320 D042 DIA 2
## 1321 D061 DIA 2
## 1322 D521 DIA 2
## 1323 D642 DIA 2
## 1324 E000 DIA 2
## 1325 E010 DIA 2
## 1326 E012 DIA 2
## 1327 E032 DIA 2
## 1328 E033 DIA 2
## 1329 E055 DIA 2
## 1330 E063 DIA 2
## 1331 E079 DIA 2
## 1332 E168 DIA 2
## 1333 E200 DIA 2
## 1334 E212 DIA 2
## 1335 E248 DIA 2
## 1336 E273 DIA 2
## 1337 E308 DIA 2
## 1338 E40X DIA 2
## 1339 E45X DIA 2
## 1340 E60X DIA 2
## 1341 E639 DIA 2
## 1342 E662 DIA 2
## 1343 E713 DIA 2
## 1344 E748 DIA 2
## 1345 E786 DIA 2
## 1346 E790 DIA 2
## 1347 E832 DIA 2
## 1348 E839 DIA 2
## 1349 E840 DIA 2
## 1350 E849 DIA 2
## 1351 E859 DIA 2
## 1352 E890 DIA 2
## 1353 F002 DIA 2
## 1354 F012 DIA 2
## 1355 F013 DIA 2
## 1356 F028 DIA 2
## 1357 F051 DIA 2
## 1358 F072 DIA 2
## 1359 F104 DIA 2
## 1360 F105 DIA 2
## 1361 F106 DIA 2
## 1362 F132 DIA 2
## 1363 F190 DIA 2
## 1364 F199 DIA 2
## 1365 F201 DIA 2
## 1366 F203 DIA 2
## 1367 F206 DIA 2
## 1368 F230 DIA 2
## 1369 F232 DIA 2
## 1370 F239 DIA 2
## 1371 F332 DIA 2
## 1372 F409 DIA 2
## 1373 F418 DIA 2
## 1374 F429 DIA 2
## 1375 F459 DIA 2
## 1376 F481 DIA 2
## 1377 F606 DIA 2
## 1378 F639 DIA 2
## 1379 F709 DIA 2
## 1380 F719 DIA 2
## 1381 F721 DIA 2
## 1382 F811 DIA 2
## 1383 F82X DIA 2
## 1384 F99X DIA 2
## 1385 G008 DIA 2
## 1386 G030 DIA 2
## 1387 G032 DIA 2
## 1388 G038 DIA 2
## 1389 G051 DIA 2
## 1390 G058 DIA 2
## 1391 G061 DIA 2
## 1392 G131 DIA 2
## 1393 G249 DIA 2
## 1394 G253 DIA 2
## 1395 G300 DIA 2
## 1396 G328 DIA 2
## 1397 G405 DIA 2
## 1398 G432 DIA 2
## 1399 G440 DIA 2
## 1400 G509 DIA 2
## 1401 G519 DIA 2
## 1402 G529 DIA 2
## 1403 G540 DIA 2
## 1404 G611 DIA 2
## 1405 G638 DIA 2
## 1406 G709 DIA 2
## 1407 G710 DIA 2
## 1408 G802 DIA 2
## 1409 G808 DIA 2
## 1410 G820 DIA 2
## 1411 G903 DIA 2
## 1412 G912 DIA 2
## 1413 G930 DIA 2
## 1414 G948 DIA 2
## 1415 G998 B690 2
## 1416 H060 DIA 2
## 1417 H062 DIA 2
## 1418 H160 DIA 2
## 1419 H162 DIA 2
## 1420 H193 DIA 2
## 1421 H359 DIA 2
## 1422 H431 DIA 2
## 1423 H600 DIA 2
## 1424 H603 DIA 2
## 1425 H620 DIA 2
## 1426 H622 DIA 2
## 1427 H652 DIA 2
## 1428 H700 DIA 2
## 1429 H708 DIA 2
## 1430 H709 DIA 2
## 1431 H730 DIA 2
## 1432 H812 DIA 2
## 1433 H902 DIA 2
## 1434 I052 DIA 2
## 1435 I080 DIA 2
## 1436 I201 DIA 2
## 1437 I208 DIA 2
## 1438 I240 DIA 2
## 1439 I288 DIA 2
## 1440 I289 DIA 2
## 1441 I311 DIA 2
## 1442 I313 DIA 2
## 1443 I319 DIA 2
## 1444 I351 DIA 2
## 1445 I352 DIA 2
## 1446 I379 DIA 2
## 1447 I400 DIA 2
## 1448 I438 DIA 2
## 1449 I452 DIA 2
## 1450 I454 DIA 2
## 1451 I472 DIA 2
## 1452 I604 DIA 2
## 1453 I607 DIA 2
## 1454 I616 DIA 2
## 1455 I652 DIA 2
## 1456 I659 R568 2
## 1457 I663 DIA 2
## 1458 I692 DIA 2
## 1459 I719 DIA 2
## 1460 I724 DIA 2
## 1461 I741 DIA 2
## 1462 I808 DIA 2
## 1463 I848 DIA 2
## 1464 I849 DIA 2
## 1465 I870 DIA 2
## 1466 I951 DIA 2
## 1467 I958 DIA 2
## 1468 I983 DIA 2
## 1469 I988 DIA 2
## 1470 J010 DIA 2
## 1471 J042 DIA 2
## 1472 J09X DIA 2
## 1473 J118 DIA 2
## 1474 J153 DIA 2
## 1475 J182 DIA 2
## 1476 J20 DIA 2
## 1477 J210 DIA 2
## 1478 J304 DIA 2
## 1479 J329 DIA 2
## 1480 J371 DIA 2
## 1481 J438 DIA 2
## 1482 J458 DIA 2
## 1483 J633 DIA 2
## 1484 J691 DIA 2
## 1485 J931 DIA 2
## 1486 J950 DIA 2
## 1487 J951 DIA 2
## 1488 J958 DIA 2
## 1489 J982 DIA 2
## 1490 J986 DIA 2
## 1491 J990 DIA 2
## 1492 K040 DIA 2
## 1493 K050 DIA 2
## 1494 K109 DIA 2
## 1495 K112 DIA 2
## 1496 K121 DIA 2
## 1497 K222 DIA 2
## 1498 K225 DIA 2
## 1499 K226 DIA 2
## 1500 K251 DIA 2
## 1501 K263 DIA 2
## 1502 K264 DIA 2
## 1503 K292 DIA 2
## 1504 K310 DIA 2
## 1505 K381 DIA 2
## 1506 K389 DIA 2
## 1507 K403 DIA 2
## 1508 K404 DIA 2
## 1509 K419 DIA 2
## 1510 K430 DIA 2
## 1511 K450 DIA 2
## 1512 K460 DIA 2
## 1513 K500 DIA 2
## 1514 K522 DIA 2
## 1515 K563 DIA 2
## 1516 K572 DIA 2
## 1517 K573 DIA 2
## 1518 K574 DIA 2
## 1519 K589 DIA 2
## 1520 K611 DIA 2
## 1521 K627 DIA 2
## 1522 K635 DIA 2
## 1523 K638 DIA 2
## 1524 K711 DIA 2
## 1525 K718 DIA 2
## 1526 K752 DIA 2
## 1527 K767 DIA 2
## 1528 K80 DIA 2
## 1529 K822 DIA 2
## 1530 K834 DIA 2
## 1531 K900 DIA 2
## 1532 K913 DIA 2
## 1533 K918 DIA 2
## 1534 L040 DIA 2
## 1535 L10 DIA 2
## 1536 L109 DIA 2
## 1537 L270 A150 2
## 1538 L301 DIA 2
## 1539 L308 DIA 2
## 1540 L510 DIA 2
## 1541 L512 DIA 2
## 1542 L739 DIA 2
## 1543 L921 DIA 2
## 1544 L932 DIA 2
## 1545 L958 DIA 2
## 1546 L982 DIA 2
## 1547 L983 DIA 2
## 1548 M000 DIA 2
## 1549 M013 DIA 2
## 1550 M058 DIA 2
## 1551 M060 DIA 2
## 1552 M104 DIA 2
## 1553 M142 DIA 2
## 1554 M148 DIA 2
## 1555 M160 DIA 2
## 1556 M190 DIA 2
## 1557 M318 DIA 2
## 1558 M319 DIA 2
## 1559 M320 DIA 2
## 1560 M328 DIA 2
## 1561 M330 DIA 2
## 1562 M340 DIA 2
## 1563 M348 DIA 2
## 1564 M349 DIA 2
## 1565 M353 DIA 2
## 1566 M354 DIA 2
## 1567 M359 DIA 2
## 1568 M360 DIA 2
## 1569 M411 DIA 2
## 1570 M45X DIA 2
## 1571 M494 DIA 2
## 1572 M531 DIA 2
## 1573 M625 DIA 2
## 1574 M626 DIA 2
## 1575 M704 DIA 2
## 1576 M715 DIA 2
## 1577 M719 DIA 2
## 1578 M722 DIA 2
## 1579 M723 DIA 2
## 1580 M724 DIA 2
## 1581 M752 DIA 2
## 1582 M755 DIA 2
## 1583 M841 DIA 2
## 1584 M899 DIA 2
## 1585 M930 DIA 2
## 1586 M940 DIA 2
## 1587 M952 DIA 2
## 1588 N030 DIA 2
## 1589 N042 DIA 2
## 1590 N069 DIA 2
## 1591 N071 DIA 2
## 1592 N078 DIA 2
## 1593 N080 DIA 2
## 1594 N136 DIA 2
## 1595 N150 DIA 2
## 1596 N20 DIA 2
## 1597 N228 DIA 2
## 1598 N251 DIA 2
## 1599 N290 DIA 2
## 1600 N301 DIA 2
## 1601 N318 DIA 2
## 1602 N341 DIA 2
## 1603 N368 DIA 2
## 1604 N392 DIA 2
## 1605 N40 DIA 2
## 1606 N434 DIA 2
## 1607 N485 DIA 2
## 1608 N508 DIA 2
## 1609 N62X DIA 2
## 1610 N649 DIA 2
## 1611 N711 DIA 2
## 1612 N719 DIA 2
## 1613 N748 DIA 2
## 1614 N763 DIA 2
## 1615 N766 DIA 2
## 1616 N770 DIA 2
## 1617 N829 DIA 2
## 1618 N842 DIA 2
## 1619 N857 DIA 2
## 1620 N888 DIA 2
## 1621 N900 DIA 2
## 1622 N910 DIA 2
## 1623 N912 DIA 2
## 1624 N949 DIA 2
## 1625 N979 DIA 2
## 1626 N993 DIA 2
## 1627 O039 DIA 2
## 1628 O211 DIA 2
## 1629 O235 DIA 2
## 1630 O269 DIA 2
## 1631 O321 DIA 2
## 1632 O366 DIA 2
## 1633 O601 DIA 2
## 1634 O60X DIA 2
## 1635 O639 DIA 2
## 1636 O731 DIA 2
## 1637 O911 DIA 2
## 1638 Q181 DIA 2
## 1639 Q211 DIA 2
## 1640 Q282 DIA 2
## 1641 Q283 DIA 2
## 1642 Q444 DIA 2
## 1643 Q505 DIA 2
## 1644 Q603 DIA 2
## 1645 Q803 DIA 2
## 1646 R063 DIA 2
## 1647 R066 DIA 2
## 1648 R071 DIA 2
## 1649 R073 DIA 2
## 1650 R091 DIA 2
## 1651 R14X DIA 2
## 1652 R34X DIA 2
## 1653 R35X DIA 2
## 1654 R36X DIA 2
## 1655 R398 DIA 2
## 1656 R450 DIA 2
## 1657 R451 DIA 2
## 1658 R458 DIA 2
## 1659 R490 DIA 2
## 1660 R740 DIA 2
## 1661 R820 DIA 2
## 1662 R827 DIA 2
## 1663 S001 DIA 2
## 1664 S027 DIA 2
## 1665 S029 DIA 2
## 1666 S051 DIA 2
## 1667 S059 DIA 2
## 1668 S089 DIA 2
## 1669 S219 DIA 2
## 1670 S220 DIA 2
## 1671 S270 DIA 2
## 1672 S272 DIA 2
## 1673 S279 DIA 2
## 1674 S309 DIA 2
## 1675 S318 DIA 2
## 1676 S322 DIA 2
## 1677 S323 DIA 2
## 1678 S364 DIA 2
## 1679 S400 DIA 2
## 1680 S421 DIA 2
## 1681 S480 DIA 2
## 1682 S489 DIA 2
## 1683 S526 DIA 2
## 1684 S589 DIA 2
## 1685 S609 DIA 2
## 1686 S627 DIA 2
## 1687 S668 DIA 2
## 1688 S670 DIA 2
## 1689 S699 DIA 2
## 1690 S749 DIA 2
## 1691 S770 DIA 2
## 1692 S801 DIA 2
## 1693 S808 DIA 2
## 1694 S825 DIA 2
## 1695 S827 DIA 2
## 1696 S842 DIA 2
## 1697 S903 DIA 2
## 1698 S921 DIA 2
## 1699 S922 DIA 2
## 1700 S930 DIA 2
## 1701 S934 DIA 2
## 1702 S971 DIA 2
## 1703 S998 DIA 2
## 1704 T008 DIA 2
## 1705 T013 DIA 2
## 1706 T055 DIA 2
## 1707 T093 DIA 2
## 1708 T111 DIA 2
## 1709 T130 DIA 2
## 1710 T202 DIA 2
## 1711 T212 DIA 2
## 1712 T222 DIA 2
## 1713 T245 DIA 2
## 1714 T246 DIA 2
## 1715 T292 DIA 2
## 1716 T348 DIA 2
## 1717 T451 DIA 2
## 1718 T639 DIA 2
## 1719 T671 DIA 2
## 1720 T68X DIA 2
## 1721 T709 DIA 2
## 1722 T782 DIA 2
## 1723 T802 DIA 2
## 1724 T809 DIA 2
## 1725 T818 DIA 2
## 1726 T829 DIA 2
## 1727 T861 DIA 2
## 1728 T868 DIA 2
## 1729 T871 DIA 2
## 1730 T886 DIA 2
## 1731 T889 DIA 2
## 1732 T930 DIA 2
## 1733 U202 DIA 2
## 1734 W010 DIA 2
## 1735 W170 DIA 2
## 1736 W180 DIA 2
## 1737 X259 DIA 2
## 1738 Y579 DIA 2
## 1739 Y841 DIA 2
## 1740 Y912 DIA 2
## 1741 Z433 DIA 2
## 1742 Z992 DIA 2
## 1743 B178 B029 1
## 1744 B358 B24X 1
## 1745 B427 A419 1
## 1746 B451 B009 1
## 1747 B779 A071 1
## 1748 C091 A419 1
## 1749 C460 B24X 1
## 1750 C919 C910 1
## 1751 D070 D060 1
## 1752 D199 B370 1
## 1753 D25 C64X 1
## 1754 D339 C719 1
## 1755 D373 A162 1
## 1756 D379 C786 1
## 1757 D382 A165 1
## 1758 D823 A419 1
## 1759 E011 DIA 1
## 1760 E040 DIA 1
## 1761 E060 DIA 1
## 1762 E11.6 DIA 1
## 1763 E111D D250 1
## 1764 E208 DIA 1
## 1765 E211 DIA 1
## 1766 E233 DIA 1
## 1767 E237 DIA 1
## 1768 E260 D509 1
## 1769 E279 DIA 1
## 1770 E282 D432 1
## 1771 E340 A150 1
## 1772 E344 DIA 1
## 1773 E348 DIA 1
## 1774 E350 DIA 1
## 1775 E43 C169 1
## 1776 E449 DIA 1
## 1777 E509 DIA 1
## 1778 E539 DIA 1
## 1779 E649 A419 1
## 1780 E6691 B972 1
## 1781 E673 DIA 1
## 1782 E709 DIA 1
## 1783 E720 DIA 1
## 1784 E728 DIA 1
## 1785 E729 DIA 1
## 1786 E738 D509 1
## 1787 E742 DIA 1
## 1788 E750 DIA 1
## 1789 E752 DIA 1
## 1790 E768 DIA 1
## 1791 E778 DIA 1
## 1792 E779 DIA 1
## 1793 E800 DIA 1
## 1794 E804 DIA 1
## 1795 E806 A090 1
## 1796 E807 DIA 1
## 1797 E850 DIA 1
## 1798 E882 DIA 1
## 1799 E893 DIA 1
## 1800 E90X DIA 1
## 1801 F000 DIA 1
## 1802 F020 DIA 1
## 1803 F023 DIA 1
## 1804 F068 DIA 1
## 1805 F070 DIA 1
## 1806 F079 DIA 1
## 1807 F116 DIA 1
## 1808 F129 DIA 1
## 1809 F135 D509 1
## 1810 F141 DIA 1
## 1811 F171 DIA 1
## 1812 F189 DIA 1
## 1813 F194 DIA 1
## 1814 F20X DIA 1
## 1815 F21X D500 1
## 1816 F229 DIA 1
## 1817 F259 DIA 1
## 1818 F31 DIA 1
## 1819 F310 DIA 1
## 1820 F311 DIA 1
## 1821 F312 DIA 1
## 1822 F313 DIA 1
## 1823 F316 DIA 1
## 1824 F330 DIA 1
## 1825 F41 DIA 1
## 1826 F440 DIA 1
## 1827 F444 DIA 1
## 1828 F500 DIA 1
## 1829 F504 DIA 1
## 1830 F509 DIA 1
## 1831 F510 DIA 1
## 1832 F514 DIA 1
## 1833 F519 DIA 1
## 1834 F602 DIA 1
## 1835 F603 DIA 1
## 1836 F604 DIA 1
## 1837 F608 DIA 1
## 1838 F61X DIA 1
## 1839 F669 DIA 1
## 1840 F708 DIA 1
## 1841 F729 DIA 1
## 1842 F780 DIA 1
## 1843 F790 DIA 1
## 1844 F800 DIA 1
## 1845 F808 DIA 1
## 1846 F844 DIA 1
## 1847 F88X DIA 1
## 1848 F900 DIA 1
## 1849 F909 DIA 1
## 1850 F919 DIA 1
## 1851 F980 DIA 1
## 1852 G000 DIA 1
## 1853 G112 DIA 1
## 1854 G114 DIA 1
## 1855 G129 DIA 1
## 1856 G20 DIA 1
## 1857 G211 DIA 1
## 1858 G218 DIA 1
## 1859 G22X DIA 1
## 1860 G231 DIA 1
## 1861 G250 DIA 1
## 1862 G258 DIA 1
## 1863 G301 DIA 1
## 1864 G368 DIA 1
## 1865 G369 DIA 1
## 1866 G370 DIA 1
## 1867 G371 DIA 1
## 1868 G372 DIA 1
## 1869 G379 DIA 1
## 1870 G412 DIA 1
## 1871 G418 DIA 1
## 1872 G441 DIA 1
## 1873 G443 DIA 1
## 1874 G444 DIA 1
## 1875 G45 DIA 1
## 1876 G452 DIA 1
## 1877 G460 DIA 1
## 1878 G462 DIA 1
## 1879 G465 D180 1
## 1880 G468 DIA 1
## 1881 G473 DIA 1
## 1882 G528 DIA 1
## 1883 G538 DIA 1
## 1884 G544 DIA 1
## 1885 G560 DIA 1
## 1886 G561 DIA 1
## 1887 G580 A010 1
## 1888 G603 DIA 1
## 1889 G64X DIA 1
## 1890 G713 DIA 1
## 1891 G732 A418 1
## 1892 G800 DIA 1
## 1893 G902 DIA 1
## 1894 G908 DIA 1
## 1895 G918 DIA 1
## 1896 G941 D432 1
## 1897 G951 DIA 1
## 1898 G958 DIA 1
## 1899 G961 DIA 1
## 1900 G968 DIA 1
## 1901 G970 DIA 1
## 1902 H000 DIA 1
## 1903 H027 DIA 1
## 1904 H031 B023 1
## 1905 H041 DIA 1
## 1906 H045 DIA 1
## 1907 H100 DIA 1
## 1908 H105 DIA 1
## 1909 H113 DIA 1
## 1910 H150 A539 1
## 1911 H151 DIA 1
## 1912 H169 DIA 1
## 1913 H189 C539 1
## 1914 H208 DIA 1
## 1915 H209 DIA 1
## 1916 H218 DIA 1
## 1917 H220 A514 1
## 1918 H250 DIA 1
## 1919 H262 DIA 1
## 1920 H282 DIA 1
## 1921 H309 DIA 1
## 1922 H335 A090 1
## 1923 H428 DIA 1
## 1924 H438 D649 1
## 1925 H441 DIA 1
## 1926 H448 DIA 1
## 1927 H449 DIA 1
## 1928 H451 DIA 1
## 1929 H472 DIA 1
## 1930 H492 DIA 1
## 1931 H493 DIA 1
## 1932 H494 DIA 1
## 1933 H521 DIA 1
## 1934 H523 DIA 1
## 1935 H524 DIA 1
## 1936 H530 DIA 1
## 1937 H533 DIA 1
## 1938 H542 DIA 1
## 1939 H578 DIA 1
## 1940 H598 DIA 1
## 1941 H604 DIA 1
## 1942 H605 DIA 1
## 1943 H60X DIA 1
## 1944 H611 DIA 1
## 1945 H678 DIA 1
## 1946 H818 DIA 1
## 1947 H830 DIA 1
## 1948 H901 A161 1
## 1949 H905 DIA 1
## 1950 H911 DIA 1
## 1951 H913 DIA 1
## 1952 H920 DIA 1
## 1953 H933 DIA 1
## 1954 I010 DIA 1
## 1955 I018 DIA 1
## 1956 I070 DIA 1
## 1957 I079 DIA 1
## 1958 I081 DIA 1
## 1959 I091 DIA 1
## 1960 I12 DIA 1
## 1961 I21 D50 1
## 1962 I233 DIA 1
## 1963 I236 DIA 1
## 1964 I238 DIA 1
## 1965 I253 DIA 1
## 1966 I254 DIA 1
## 1967 I256 B342 1
## 1968 I272 DIA 1
## 1969 I28 E11 1
## 1970 I280 DIA 1
## 1971 I300 C300 1
## 1972 I301 DIA 1
## 1973 I321 DIA 1
## 1974 I348 DIA 1
## 1975 I361 DIA 1
## 1976 I369 DIA 1
## 1977 I372 DIA 1
## 1978 I392 B342 1
## 1979 I409 DIA 1
## 1980 I411 DIA 1
## 1981 I422 DIA 1
## 1982 I424 DIA 1
## 1983 I432 DIA 1
## 1984 I453 DIA 1
## 1985 I456 DIA 1
## 1986 I470 DIA 1
## 1987 I493 DIA 1
## 1988 I513 DIA 1
## 1989 I603 DIA 1
## 1990 I636 DIA 1
## 1991 I650 DIA 1
## 1992 I651 DIA 1
## 1993 I661 DIA 1
## 1994 I664 DIA 1
## 1995 I669 DIA 1
## 1996 I676 DIA 1
## 1997 I69 DIA 1
## 1998 I708 DIA 1
## 1999 I718 DIA 1
## 2000 I720 DIA 1
## 2001 I721 DIA 1
## 2002 I730 DIA 1
## 2003 I745 DIA 1
## 2004 I772 DIA 1
## 2005 I774 DIA 1
## 2006 I790 DIA 1
## 2007 I798 DIA 1
## 2008 I81X DIA 1
## 2009 I840 DIA 1
## 2010 I861 DIA 1
## 2011 I863 DIA 1
## 2012 I898 DIA 1
## 2013 I950 DIA 1
## 2014 J020 DIA 1
## 2015 J028 DIA 1
## 2016 J030 DIA 1
## 2017 J038 DIA 1
## 2018 J041 DIA 1
## 2019 J06 DIA 1
## 2020 J060 DIA 1
## 2021 J110 D509 1
## 2022 J111 DIA 1
## 2023 J122 DIA 1
## 2024 J172 DIA 1
## 2025 J173 B59X 1
## 2026 J20X DIA 1
## 2027 J211 DIA 1
## 2028 J312 DIA 1
## 2029 J321 DIA 1
## 2030 J330 DIA 1
## 2031 J339 DIA 1
## 2032 J359 DIA 1
## 2033 J370 DIA 1
## 2034 J382 DIA 1
## 2035 J391 DIA 1
## 2036 J392 DIA 1
## 2037 J40 E11 1
## 2038 J42 D500 1
## 2039 J439 D649 1
## 2040 J45 E11 1
## 2041 J45X DIA 1
## 2042 J47 DIA 1
## 2043 J60X DIA 1
## 2044 J620 DIA 1
## 2045 J628 DIA 1
## 2046 J634 D649 1
## 2047 J638 DIA 1
## 2048 J65X DIA 1
## 2049 J671 DIA 1
## 2050 J677 DIA 1
## 2051 J684 DIA 1
## 2052 J929 DIA 1
## 2053 J940 DIA 1
## 2054 J953 DIA 1
## 2055 J955 B972 1
## 2056 J96 A90 1
## 2057 J98O DIA 1
## 2058 K021 DIA 1
## 2059 K039 B374 1
## 2060 K051 DIA 1
## 2061 K053 DIA 1
## 2062 K068 DIA 1
## 2063 K088 DIA 1
## 2064 K092 DIA 1
## 2065 K099 DIA 1
## 2066 K103 DIA 1
## 2067 K115 DIA 1
## 2068 K118 DIA 1
## 2069 K119 A09X 1
## 2070 K120 C859 1
## 2071 K123 A150 1
## 2072 K20 DIA 1
## 2073 K220 A150 1
## 2074 K25 D53 1
## 2075 k250 DIA 1
## 2076 K252 DIA 1
## 2077 K255 DIA 1
## 2078 K256 DIA 1
## 2079 K261 A418 1
## 2080 K267 DIA 1
## 2081 K274 DIA 1
## 2082 K275 A419 1
## 2083 K311 D649 1
## 2084 K314 DIA 1
## 2085 K315 DIA 1
## 2086 K383 DIA 1
## 2087 K40 C61 1
## 2088 K410 DIA 1
## 2089 K431 DIA 1
## 2090 K509 DIA 1
## 2091 K510 DIA 1
## 2092 K512 D500 1
## 2093 K515 DIA 1
## 2094 K520 DIA 1
## 2095 K521 DIA 1
## 2096 K528 DIA 1
## 2097 K552 DIA 1
## 2098 K578 DIA 1
## 2099 K580 DIA 1
## 2100 K59 DIA 1
## 2101 K592 DIA 1
## 2102 K599 DIA 1
## 2103 K601 DIA 1
## 2104 K604 DIA 1
## 2105 K614 DIA 1
## 2106 K620 DIA 1
## 2107 K624 DIA 1
## 2108 K633 D500 1
## 2109 K640 DIA 1
## 2110 K641 DIA 1
## 2111 K66 E14 1
## 2112 K713 A099 1
## 2113 K714 DIA 1
## 2114 K716 DIA 1
## 2115 K730 DIA 1
## 2116 K732 D134 1
## 2117 K738 DIA 1
## 2118 K753 DIA 1
## 2119 K754 DIA 1
## 2120 K76 E14 1
## 2121 K761 DIA 1
## 2122 K770 D509 1
## 2123 K778 DIA 1
## 2124 K80X DIA 1
## 2125 K81X B24X 1
## 2126 K821 DIA 1
## 2127 K824 DIA 1
## 2128 K832 DIA 1
## 2129 K86 DIA 1
## 2130 K90 E14 1
## 2131 K938 DIA 1
## 2132 L03X E11X 1
## 2133 L059 DIA 1
## 2134 L08 DIA 1
## 2135 L081 D508 1
## 2136 L108 DIA 1
## 2137 L120 DIA 1
## 2138 L121 D649 1
## 2139 L129 DIA 1
## 2140 L139 DIA 1
## 2141 L14X DIA 1
## 2142 L200 DIA 1
## 2143 L208 DIA 1
## 2144 L210 DIA 1
## 2145 L22X DIA 1
## 2146 L231 D509 1
## 2147 L238 DIA 1
## 2148 L249 DIA 1
## 2149 L26 A499 1
## 2150 L271 DIA 1
## 2151 L280 DIA 1
## 2152 L282 DIA 1
## 2153 L292 DIA 1
## 2154 L298 DIA 1
## 2155 L300 DIA 1
## 2156 L414 DIA 1
## 2157 L480 DIA 1
## 2158 L501 DIA 1
## 2159 L502 DIA 1
## 2160 L508 DIA 1
## 2161 L519 D649 1
## 2162 L538 D649 1
## 2163 L551 DIA 1
## 2164 L570 DIA 1
## 2165 L602 DIA 1
## 2166 L603 DIA 1
## 2167 L640 DIA 1
## 2168 L680 DIA 1
## 2169 L702 DIA 1
## 2170 L817 DIA 1
## 2171 L819 A099 1
## 2172 L82X DIA 1
## 2173 L84X DIA 1
## 2174 L853 DIA 1
## 2175 L871 DIA 1
## 2176 L903 DIA 1
## 2177 L905 DIA 1
## 2178 L929 DIA 1
## 2179 L931 DIA 1
## 2180 L940 DIA 1
## 2181 L97 DIA 1
## 2182 L980 B180 1
## 2183 L998 DIA 1
## 2184 M008 DIA 1
## 2185 M018 DIA 1
## 2186 M030 DIA 1
## 2187 M052 DIA 1
## 2188 M064 DIA 1
## 2189 M0699 DIA 1
## 2190 M082 DIA 1
## 2191 M103 DIA 1
## 2192 M112 DIA 1
## 2193 M120 DIA 1
## 2194 M138 DIA 1
## 2195 M145 DIA 1
## 2196 M153 DIA 1
## 2197 M159 A090 1
## 2198 M161 DIA 1
## 2199 M172 DIA 1
## 2200 M175 DIA 1
## 2201 M185 DIA 1
## 2202 M191 DIA 1
## 2203 M198 DIA 1
## 2204 M204 DIA 1
## 2205 M211 DIA 1
## 2206 M215 DIA 1
## 2207 M224 DIA 1
## 2208 M231 DIA 1
## 2209 M232 DIA 1
## 2210 M235 DIA 1
## 2211 M243 DIA 1
## 2212 M245 DIA 1
## 2213 M250 DIA 1
## 2214 M259 DIA 1
## 2215 M316 DIA 1
## 2216 M317 DIA 1
## 2217 M350 DIA 1
## 2218 M361 DIA 1
## 2219 M413 DIA 1
## 2220 M418 DIA 1
## 2221 M421 A498 1
## 2222 M429 DIA 1
## 2223 M462 DIA 1
## 2224 M47 E14 1
## 2225 M470 DIA 1
## 2226 M471 DIA 1
## 2227 M488 DIA 1
## 2228 M493 DIA 1
## 2229 M502 DIA 1
## 2230 M510 DIA 1
## 2231 M518 DIA 1
## 2232 M530 DIA 1
## 2233 M540 D530 1
## 2234 M546 DIA 1
## 2235 M548 DIA 1
## 2236 M621 DIA 1
## 2237 M650 DIA 1
## 2238 M651 DIA 1
## 2239 M653 DIA 1
## 2240 M658 DIA 1
## 2241 M662 DIA 1
## 2242 M688 DIA 1
## 2243 M701 DIA 1
## 2244 M703 DIA 1
## 2245 M706 DIA 1
## 2246 M711 DIA 1
## 2247 M751 D693 1
## 2248 M753 DIA 1
## 2249 M754 DIA 1
## 2250 M758 DIA 1
## 2251 M760 D500 1
## 2252 M761 DIA 1
## 2253 M762 DIA 1
## 2254 M763 DIA 1
## 2255 M766 DIA 1
## 2256 M770 DIA 1
## 2257 M773 DIA 1
## 2258 M792 DIA 1
## 2259 M796 DIA 1
## 2260 M797 DIA 1
## 2261 M800 DIA 1
## 2262 M839 DIA 1
## 2263 M843 DIA 1
## 2264 M848 DIA 1
## 2265 M850 DIA 1
## 2266 M854 C050 1
## 2267 M862 A419 1
## 2268 M865 DIA 1
## 2269 M898 DIA 1
## 2270 M901 DIA 1
## 2271 M902 DIA 1
## 2272 M903 DIA 1
## 2273 M906 DIA 1
## 2274 M922 D500 1
## 2275 M928 DIA 1
## 2276 M948 DIA 1
## 2277 M960 DIA 1
## 2278 M966 DIA 1
## 2279 M990 DIA 1
## 2280 M995 DIA 1
## 2281 N002 DIA 1
## 2282 N021 DIA 1
## 2283 N025 DIA 1
## 2284 N028 DIA 1
## 2285 N058 DIA 1
## 2286 N063 A09X 1
## 2287 N070 DIA 1
## 2288 N088 DIA 1
## 2289 N12 DIA 1
## 2290 N134 DIA 1
## 2291 N135 DIA 1
## 2292 N140 DIA 1
## 2293 N164 DIA 1
## 2294 N165 DIA 1
## 2295 N171 DIA 1
## 2296 N19 D599 1
## 2297 N21 E13 1
## 2298 N218 DIA 1
## 2299 N222 DIA 1
## 2300 N26X D649 1
## 2301 N298 DIA 1
## 2302 N303 DIA 1
## 2303 N304 D501 1
## 2304 N308 DIA 1
## 2305 N329 A049 1
## 2306 N342 DIA 1
## 2307 N358 DIA 1
## 2308 N360 DIA 1
## 2309 N362 DIA 1
## 2310 N369 A419 1
## 2311 N370 DIA 1
## 2312 N418 DIA 1
## 2313 N46 DIA 1
## 2314 N48 DIA 1
## 2315 N489 DIA 1
## 2316 N500 DIA 1
## 2317 N509 DIA 1
## 2318 N600 DIA 1
## 2319 N601 DIA 1
## 2320 N61 E14 1
## 2321 N641 A419 1
## 2322 N645 DIA 1
## 2323 N730 DIA 1
## 2324 N731 D589 1
## 2325 N732 D539 1
## 2326 N734 DIA 1
## 2327 N76 DIA 1
## 2328 N762 DIA 1
## 2329 N765 D049 1
## 2330 N808 DIA 1
## 2331 N81 B972 1
## 2332 N828 DIA 1
## 2333 N831 DIA 1
## 2334 N8324 DIA 1
## 2335 N838 DIA 1
## 2336 N839 DIA 1
## 2337 N849 DIA 1
## 2338 N855 DIA 1
## 2339 N890 DIA 1
## 2340 N891 DIA 1
## 2341 N908 DIA 1
## 2342 N909 DIA 1
## 2343 N950 D649 1
## 2344 N951 E10X 1
## 2345 N970 DIA 1
## 2346 N992 DIA 1
## 2347 N998 DIA 1
## 2348 O000 DIA 1
## 2349 O010 DIA 1
## 2350 O019 DIA 1
## 2351 O030 DIA 1
## 2352 O033 DIA 1
## 2353 O054 DIA 1
## 2354 O088 DIA 1
## 2355 O219 DIA 1
## 2356 O220 DIA 1
## 2357 O231 DIA 1
## 2358 O239 DIA 1
## 2359 O243 DIA 1
## 2360 O244 DIA 1
## 2361 O25X DIA 1
## 2362 O300 DIA 1
## 2363 O312 DIA 1
## 2364 O323 DIA 1
## 2365 O324 DIA 1
## 2366 O326 DIA 1
## 2367 O330 DIA 1
## 2368 O334 DIA 1
## 2369 O338 DIA 1
## 2370 O339 DIA 1
## 2371 O360 DIA 1
## 2372 O365 DIA 1
## 2373 O368 DIA 1
## 2374 O420 DIA 1
## 2375 O421 DIA 1
## 2376 O439 DIA 1
## 2377 O471 DIA 1
## 2378 O610 DIA 1
## 2379 O624 DIA 1
## 2380 O645 DIA 1
## 2381 O648 DIA 1
## 2382 O662 DIA 1
## 2383 O679 DIA 1
## 2384 O700 DIA 1
## 2385 O710 DIA 1
## 2386 O722 DIA 1
## 2387 O759 DIA 1
## 2388 O8001 DIA 1
## 2389 O808 DIA 1
## 2390 O8284 DIA 1
## 2391 O838 DIA 1
## 2392 O840 B972 1
## 2393 O85X DIA 1
## 2394 O862 DIA 1
## 2395 O863 DIA 1
## 2396 O868 DIA 1
## 2397 O900 DIA 1
## 2398 O908 DIA 1
## 2399 O994 DIA 1
## 2400 P369 DIA 1
## 2401 P95X DIA 1
## 2402 Q019 DIA 1
## 2403 Q046 DIA 1
## 2404 Q049 DIA 1
## 2405 Q219 DIA 1
## 2406 Q253 DIA 1
## 2407 Q259 DIA 1
## 2408 Q273 DIA 1
## 2409 Q279 DIA 1
## 2410 Q400 DIA 1
## 2411 Q602 DIA 1
## 2412 Q605 DIA 1
## 2413 Q612 C609 1
## 2414 Q620 DIA 1
## 2415 Q631 D410 1
## 2416 Q649 DIA 1
## 2417 Q660 DIA 1
## 2418 Q720 C300 1
## 2419 Q738 DIA 1
## 2420 Q780 DIA 1
## 2421 Q785 DIA 1
## 2422 Q810 DIA 1
## 2423 Q811 DIA 1
## 2424 Q828 DIA 1
## 2425 Q831 DIA 1
## 2426 Q850 DIA 1
## 2427 Q871 DIA 1
## 2428 Q874 DIA 1
## 2429 Q96 DIA 1
## 2430 R011 DIA 1
## 2431 R02 D509 1
## 2432 R031 DIA 1
## 2433 R068 DIA 1
## 2434 R090 DIA 1
## 2435 R12X DIA 1
## 2436 R17 C249 1
## 2437 R198 DIA 1
## 2438 R224 DIA 1
## 2439 R270 DIA 1
## 2440 R278 D649 1
## 2441 R301 D509 1
## 2442 R390 DIA 1
## 2443 R400 DIA 1
## 2444 R470 DIA 1
## 2445 R478 DIA 1
## 2446 R51 DIA 1
## 2447 R54 A415 1
## 2448 R599 DIA 1
## 2449 R632 DIA 1
## 2450 R652 DIA 1
## 2451 R680 A403 1
## 2452 R749 DIA 1
## 2453 R785 DIA 1
## 2454 R799 DIA 1
## 2455 R826 D508 1
## 2456 R829 B972 1
## 2457 R831 A152 1
## 2458 R900 DIA 1
## 2459 R931 DIA 1
## 2460 R98X DIA 1
## 2461 S007 DIA 1
## 2462 S014 DIA 1
## 2463 S015 DIA 1
## 2464 S017 DIA 1
## 2465 S018 DIA 1
## 2466 S025 DIA 1
## 2467 S026 DIA 1
## 2468 S031 DIA 1
## 2469 S045 DIA 1
## 2470 S055 B370 1
## 2471 S056 DIA 1
## 2472 S060 DIA 1
## 2473 S061 DIA 1
## 2474 S063 DIA 1
## 2475 S068 DIA 1
## 2476 S06X E10X 1
## 2477 S071 DIA 1
## 2478 S098 DIA 1
## 2479 S100 DIA 1
## 2480 S119 DIA 1
## 2481 S127 DIA 1
## 2482 S129 DIA 1
## 2483 S143 DIA 1
## 2484 S144 DIA 1
## 2485 S151 DIA 1
## 2486 S159 DIA 1
## 2487 S203 DIA 1
## 2488 S208 DIA 1
## 2489 S218 DIA 1
## 2490 S222 B001 1
## 2491 S228 DIA 1
## 2492 S230 DIA 1
## 2493 S298 DIA 1
## 2494 S302 DIA 1
## 2495 S308 DIA 1
## 2496 S312 DIA 1
## 2497 S315 DIA 1
## 2498 S325 D648 1
## 2499 S327 DIA 1
## 2500 S340 DIA 1
## 2501 S341 DIA 1
## 2502 S344 DIA 1
## 2503 S359 D62X 1
## 2504 S361 D649 1
## 2505 S372 DIA 1
## 2506 S373 A419 1
## 2507 S390 DIA 1
## 2508 S398 D62X 1
## 2509 S407 DIA 1
## 2510 S408 DIA 1
## 2511 S410 DIA 1
## 2512 S418 DIA 1
## 2513 S429 DIA 1
## 2514 S431 DIA 1
## 2515 S459 DIA 1
## 2516 S460 DIA 1
## 2517 S481 DIA 1
## 2518 S498 DIA 1
## 2519 S507 DIA 1
## 2520 S510 D649 1
## 2521 S521 DIA 1
## 2522 S523 DIA 1
## 2523 S531 D649 1
## 2524 S550 DIA 1
## 2525 S580 DIA 1
## 2526 S607 DIA 1
## 2527 S623 DIA 1
## 2528 S635 DIA 1
## 2529 S644 DIA 1
## 2530 S678 DIA 1
## 2531 S684 DIA 1
## 2532 S689 DIA 1
## 2533 S708 DIA 1
## 2534 S717 DIA 1
## 2535 S748 DIA 1
## 2536 S799 DIA 1
## 2537 S809 DIA 1
## 2538 S832 DIA 1
## 2539 S849 DIA 1
## 2540 S860 DIA 1
## 2541 S878 DIA 1
## 2542 S899 DIA 1
## 2543 S900 DIA 1
## 2544 S902 DIA 1
## 2545 S907 DIA 1
## 2546 S908 DIA 1
## 2547 S909 DIA 1
## 2548 S923 DIA 1
## 2549 S927 DIA 1
## 2550 S936 A480 1
## 2551 S948 DIA 1
## 2552 S960 DIA 1
## 2553 S978 DIA 1
## 2554 S999 DIA 1
## 2555 T000 DIA 1
## 2556 T018 DIA 1
## 2557 T050 DIA 1
## 2558 T058 DIA 1
## 2559 T068 DIA 1
## 2560 T08X B972 1
## 2561 T099 DIA 1
## 2562 T135 DIA 1
## 2563 T138 DIA 1
## 2564 T139 DIA 1
## 2565 T142 DIA 1
## 2566 T145 DIA 1
## 2567 T148 DIA 1
## 2568 T150 DIA 1
## 2569 T180 DIA 1
## 2570 T189 DIA 1
## 2571 T191 DIA 1
## 2572 T210 DIA 1
## 2573 T214 A419 1
## 2574 T230 DIA 1
## 2575 T231 DIA 1
## 2576 T241 DIA 1
## 2577 T291 DIA 1
## 2578 T293 DIA 1
## 2579 T300 DIA 1
## 2580 T304 DIA 1
## 2581 T315 DIA 1
## 2582 T365 DIA 1
## 2583 T368 DIA 1
## 2584 T409 DIA 1
## 2585 T441 DIA 1
## 2586 T443 DIA 1
## 2587 T479 DIA 1
## 2588 T528 DIA 1
## 2589 T541 DIA 1
## 2590 T542 DIA 1
## 2591 T58X DIA 1
## 2592 T601 DIA 1
## 2593 T604 DIA 1
## 2594 T635 DIA 1
## 2595 T652 DIA 1
## 2596 T670 DIA 1
## 2597 T674 DIA 1
## 2598 T698 DIA 1
## 2599 T741 DIA 1
## 2600 T751 DIA 1
## 2601 T78 DIA 1
## 2602 T783 DIA 1
## 2603 T789 DIA 1
## 2604 T794 DIA 1
## 2605 T801 DIA 1
## 2606 T803 D689 1
## 2607 T808 DIA 1
## 2608 T815 DIA 1
## 2609 T819 DIA 1
## 2610 T833 DIA 1
## 2611 T835 D649 1
## 2612 T848 DIA 1
## 2613 T859 DIA 1
## 2614 T860 DIA 1
## 2615 T869 DIA 1
## 2616 T935 DIA 1
## 2617 T936 DIA 1
## 2618 U205 DIA 1
## 2619 V099 DIA 1
## 2620 V385 DIA 1
## 2621 W018 DIA 1
## 2622 W060 DIA 1
## 2623 W079 DIA 1
## 2624 W089 DIA 1
## 2625 W159 DIA 1
## 2626 W184 DIA 1
## 2627 W204 DIA 1
## 2628 W229 DIA 1
## 2629 X100 DIA 1
## 2630 X109 DIA 1
## 2631 X689 A059 1
## 2632 X690 DIA 1
## 2633 X699 DIA 1
## 2634 X954 DIA 1
## 2635 Y279 DIA 1
## 2636 Y411 A153 1
## 2637 Y423 DIA 1
## 2638 Y822 DIA 1
## 2639 Y832 A419 1
## 2640 Y834 DIA 1
## 2641 Y846 DIA 1
## 2642 Y913 DIA 1
## 2643 Z038 DIA 1
## 2644 Z208 DIA 1
## 2645 Z226 B86X 1
## 2646 Z3593 DIA 1
## 2647 Z391 DIA 1
## 2648 Z392 DIA 1
## 2649 Z450 DIA 1
## 2650 Z549 DIA 1
## 2651 Z639 DIA 1
## 2652 Z721 DIA 1
## 2653 Z730 DIA 1
## 2654 Z749 DIA 1
## 2655 Z930 DIA 1
## 2656 Z932 DIA 1
## 2657 Z958 DIA 1
## 2658 Z988 DIA 1

## Before lockdown

## Diag1 Diag2 Frequency
## 1 I10X DIA 9931
## 2 N390 DIA 8874
## 3 N189 DIA 2866
## 4 J189 DIA 1736
## 5 D649 DIA 1408
## 6 A419 DIA 1336
## 7 L031 DIA 1113
## 8 I500 DIA 808
## 9 N185 DIA 789
## 10 N10X DIA 776
## 11 J960 DIA 714
## 12 E669 DIA 702
## 13 E162 DIA 660
## 14 I64X DIA 616
## 15 D509 DIA 554
## 16 N40X DIA 473
## 17 E039 DIA 471
## 18 K922 DIA 468
## 19 I509 DIA 451
## 20 K746 DIA 434
## 21 J159 DIA 426
## 22 J969 DIA 426
## 23 I219 DIA 425
## 24 I678 DIA 387
## 25 I639 DIA 381
## 26 L039 DIA 367
## 27 R739 DIA 364
## 28 A090 DIA 351
## 29 A099 DIA 351
## 30 N110 DIA 342
## 31 A150 DIA 309
## 32 R104 DIA 298
## 33 E86X DIA 296
## 34 N12X DIA 296
## 35 N179 DIA 278
## 36 A560 DIA 263
## 37 N039 DIA 257
## 38 S913 DIA 254
## 39 I679 DIA 245
## 40 J90X DIA 234
## 41 K811 DIA 233
## 42 J849 DIA 226
## 43 K802 DIA 224
## 44 J841 DIA 221
## 45 N200 DIA 217
## 46 K859 DIA 213
## 47 N19X DIA 202
## 48 K703 DIA 189
## 49 I739 DIA 182
## 50 I119 DIA 181
## 51 L97X DIA 168
## 52 L030 DIA 166
## 53 I489 DIA 164
## 54 J459 DIA 163
## 55 G409 DIA 162
## 56 G934 DIA 159
## 57 D259 DIA 156
## 58 K810 DIA 154
## 59 D539 DIA 149
## 60 A418 DIA 148
## 61 I619 DIA 146
## 62 I159 DIA 145
## 63 I694 DIA 145
## 64 L024 DIA 138
## 65 R509 DIA 137
## 66 D500 DIA 136
## 67 J961 DIA 132
## 68 K297 DIA 131
## 69 L038 DIA 124
## 70 N819 DIA 122
## 71 R568 DIA 122
## 72 K801 DIA 120
## 73 J209 DIA 119
## 74 M869 DIA 118
## 75 J180 DIA 114
## 76 K805 DIA 113
## 77 L023 DIA 113
## 78 R02X DIA 113
## 79 E660 DIA 112
## 80 J690 DIA 111
## 81 M069 DIA 111
## 82 N180 DIA 111
## 83 T136 DIA 111
## 84 I480 DIA 108
## 85 R11X DIA 105
## 86 I872 DIA 104
## 87 A162 DIA 101
## 88 E160 DIA 99
## 89 L899 DIA 98
## 90 G459 DIA 97
## 91 E872 DIA 96
## 92 L032 DIA 95
## 93 L029 DIA 94
## 94 N184 DIA 94
## 95 I259 DIA 93
## 96 E785 DIA 92
## 97 E668 DIA 91
## 98 I110 DIA 91
## 99 I200 DIA 90
## 100 K800 DIA 90
## 101 A169 DIA 88
## 102 N181 DIA 88
## 103 S981 DIA 88
## 104 S984 DIA 88
## 105 E46X DIA 87
## 106 K850 DIA 85
## 107 H360 DIA 83
## 108 K295 DIA 83
## 109 N151 DIA 83
## 110 K429 DIA 81
## 111 N399 DIA 81
## 112 E780 DIA 80
## 113 J188 DIA 80
## 114 K610 DIA 80
## 115 K729 DIA 80
## 116 K85X DIA 79
## 117 N183 DIA 79
## 118 S889 DIA 78
## 119 N111 DIA 77
## 120 E782 DIA 76
## 121 I48X DIA 76
## 122 I120 DIA 74
## 123 I633 DIA 74
## 124 K291 DIA 74
## 125 J13X DIA 73
## 126 R572 DIA 73
## 127 J120 DIA 71
## 128 I802 DIA 69
## 129 L089 DIA 69
## 130 R18X DIA 69
## 131 J80X DIA 68
## 132 N813 DIA 68
## 133 K409 DIA 67
## 134 D648 DIA 66
## 135 G20X DIA 66
## 136 N832 DIA 66
## 137 R042 DIA 66
## 138 A09X DIA 65
## 139 I499 DIA 65
## 140 K439 DIA 64
## 141 I610 DIA 63
## 142 K819 DIA 63
## 143 I830 DIA 62
## 144 K590 DIA 62
## 145 L022 DIA 62
## 146 N049 DIA 62
## 147 J81X DIA 61
## 148 L984 DIA 61
## 149 R51X DIA 60
## 150 N119 DIA 59
## 151 A409 DIA 58
## 152 K566 DIA 58
## 153 F200 DIA 56
## 154 I209 DIA 56
## 155 L890 DIA 56
## 156 T814 DIA 56
## 157 J029 DIA 55
## 158 E871 DIA 54
## 159 N319 DIA 54
## 160 I693 DIA 53
## 161 N760 DIA 53
## 162 D638 DIA 52
## 163 F329 DIA 52
## 164 S069 DIA 52
## 165 F209 DIA 51
## 166 I829 DIA 51
## 167 K808 DIA 51
## 168 N289 DIA 51
## 169 E059 DIA 50
## 170 F412 DIA 50
## 171 A159 DIA 49
## 172 I469 DIA 49
## 173 K30X DIA 49
## 174 I150 DIA 48
## 175 I609 DIA 48
## 176 J158 DIA 48
## 177 J129 DIA 47
## 178 K750 DIA 47
## 179 R100 DIA 47
## 180 S982 DIA 47
## 181 I442 DIA 46
## 182 I859 DIA 46
## 183 J852 DIA 46
## 184 L021 DIA 46
## 185 M725 DIA 46
## 186 J22X DIA 45
## 187 A010 DIA 44
## 188 F03X DIA 44
## 189 G590 DIA 44
## 190 I792 DIA 44
## 191 L020 DIA 44
## 192 K830 DIA 43
## 193 C539 DIA 42
## 194 N139 DIA 42
## 195 N498 DIA 42
## 196 R17X DIA 42
## 197 S721 DIA 42
## 198 A153 DIA 41
## 199 I634 DIA 41
## 200 I698 DIA 41
## 201 J40X DIA 41
## 202 O829 DIA 41
## 203 R609 DIA 41
## 204 S819 DIA 41
## 205 G632 DIA 40
## 206 L409 DIA 40
## 207 I832 DIA 39
## 208 K769 DIA 39
## 209 S822 DIA 39
## 210 D693 DIA 38
## 211 F419 DIA 38
## 212 J181 DIA 38
## 213 J984 DIA 38
## 214 N133 DIA 38
## 215 C169 DIA 37
## 216 E43X DIA 37
## 217 H269 DIA 37
## 218 I255 DIA 37
## 219 I630 DIA 37
## 220 K047 DIA 37
## 221 R571 DIA 37
## 222 C61X DIA 36
## 223 E876 DIA 36
## 224 J869 DIA 36
## 225 K259 DIA 36
## 226 N209 DIA 36
## 227 E161 DIA 35
## 228 N61X DIA 35
## 229 T009 DIA 35
## 230 T874 DIA 35
## 231 E038 DIA 34
## 232 G610 DIA 34
## 233 N814 DIA 34
## 234 R101 DIA 34
## 235 S880 DIA 34
## 236 B86X DIA 33
## 237 E440 DIA 33
## 238 E870 DIA 33
## 239 E880 DIA 33
## 240 L033 DIA 33
## 241 L89X DIA 33
## 242 M109 DIA 33
## 243 M726 DIA 33
## 244 S729 DIA 33
## 245 E878 DIA 32
## 246 R31X DIA 32
## 247 R33X DIA 32
## 248 A499 DIA 31
## 249 C189 DIA 31
## 250 F102 DIA 30
## 251 I340 DIA 30
## 252 I490 DIA 30
## 253 J152 DIA 30
## 254 N182 DIA 30
## 255 O821 DIA 30
## 256 G629 DIA 29
## 257 I519 DIA 29
## 258 I743 DIA 29
## 259 J150 DIA 29
## 260 K37X DIA 29
## 261 G309 DIA 28
## 262 G819 DIA 28
## 263 I850 DIA 28
## 264 J100 DIA 28
## 265 J448 DIA 28
## 266 M139 DIA 28
## 267 N771 DIA 28
## 268 R570 DIA 28
## 269 G909 DIA 27
## 270 I210 DIA 27
## 271 J46X DIA 27
## 272 K290 DIA 27
## 273 K851 DIA 27
## 274 N083 DIA 27
## 275 O990 DIA 27
## 276 T887 DIA 27
## 277 D696 DIA 26
## 278 E249 DIA 26
## 279 H819 DIA 26
## 280 I269 DIA 26
## 281 I620 DIA 26
## 282 K122 DIA 26
## 283 N812 DIA 26
## 284 A90X DIA 25
## 285 G510 DIA 25
## 286 I151 DIA 25
## 287 I828 DIA 25
## 288 M009 DIA 25
## 289 N23X DIA 25
## 290 S065 DIA 25
## 291 S911 DIA 25
## 292 C250 DIA 24
## 293 E889 DIA 24
## 294 I612 DIA 24
## 295 I959 DIA 24
## 296 K250 DIA 24
## 297 K920 DIA 24
## 298 N459 DIA 24
## 299 N492 DIA 24
## 300 A156 DIA 23
## 301 A410 DIA 23
## 302 C229 DIA 23
## 303 C56X DIA 23
## 304 H050 DIA 23
## 305 I158 DIA 23
## 306 I249 DIA 23
## 307 I635 DIA 23
## 308 I771 DIA 23
## 309 J156 DIA 23
## 310 J440 DIA 23
## 311 K296 DIA 23
## 312 M179 DIA 23
## 313 O234 DIA 23
## 314 T141 DIA 23
## 315 B829 DIA 22
## 316 G442 DIA 22
## 317 G969 DIA 22
## 318 I629 DIA 22
## 319 J698 DIA 22
## 320 J980 DIA 22
## 321 K469 DIA 22
## 322 K591 DIA 22
## 323 K650 DIA 22
## 324 N312 DIA 22
## 325 S681 DIA 22
## 326 A971 DIA 21
## 327 C220 DIA 21
## 328 E441 DIA 21
## 329 J068 DIA 21
## 330 J91X DIA 21
## 331 K279 DIA 21
## 332 K745 DIA 21
## 333 O034 DIA 21
## 334 O342 DIA 21
## 335 R32X DIA 21
## 336 R418 DIA 21
## 337 S828 DIA 21
## 338 S980 DIA 21
## 339 H280 DIA 20
## 340 H540 DIA 20
## 341 H813 DIA 20
## 342 I702 DIA 20
## 343 N300 DIA 20
## 344 F321 DIA 19
## 345 H409 DIA 19
## 346 I809 DIA 19
## 347 I839 DIA 19
## 348 K564 DIA 19
## 349 K760 DIA 19
## 350 M059 DIA 19
## 351 N170 DIA 19
## 352 N178 DIA 19
## 353 N310 DIA 19
## 354 N938 DIA 19
## 355 T793 DIA 19
## 356 B370 DIA 18
## 357 B379 DIA 18
## 358 C259 DIA 18
## 359 D410 DIA 18
## 360 E835 DIA 18
## 361 I471 DIA 18
## 362 J42X DIA 18
## 363 J851 DIA 18
## 364 L400 DIA 18
## 365 O820 DIA 18
## 366 R001 DIA 18
## 367 R579 DIA 18
## 368 S789 DIA 18
## 369 S818 DIA 18
## 370 T252 DIA 18
## 371 A400 DIA 17
## 372 D531 DIA 17
## 373 G049 DIA 17
## 374 H669 DIA 17
## 375 I251 DIA 17
## 376 I443 DIA 17
## 377 J219 DIA 17
## 378 K269 DIA 17
## 379 K359 DIA 17
## 380 L509 DIA 17
## 381 L80X DIA 17
## 382 L893 DIA 17
## 383 M544 DIA 17
## 384 N040 DIA 17
## 385 N172 DIA 17
## 386 N220 DIA 17
## 387 O809 DIA 17
## 388 S912 DIA 17
## 389 E875 DIA 16
## 390 G255 DIA 16
## 391 G408 DIA 16
## 392 I618 DIA 16
## 393 I632 DIA 16
## 394 I749 DIA 16
## 395 L088 DIA 16
## 396 N159 DIA 16
## 397 O064 DIA 16
## 398 R601 DIA 16
## 399 R688 DIA 16
## 400 R770 DIA 16
## 401 T131 DIA 16
## 402 T147 DIA 16
## 403 T633 DIA 16
## 404 T813 DIA 16
## 405 Y835 DIA 16
## 406 A170 DIA 15
## 407 C910 DIA 15
## 408 E169 DIA 15
## 409 F019 DIA 15
## 410 G990 DIA 15
## 411 H46X DIA 15
## 412 H811 DIA 15
## 413 H82X DIA 15
## 414 I129 DIA 15
## 415 I420 DIA 15
## 416 J069 DIA 15
## 417 J123 DIA 15
## 418 J151 DIA 15
## 419 K20X DIA 15
## 420 K420 DIA 15
## 421 K659 DIA 15
## 422 L88X DIA 15
## 423 N188 DIA 15
## 424 N309 DIA 15
## 425 N499 DIA 15
## 426 R072 DIA 15
## 427 S723 DIA 15
## 428 S917 DIA 15
## 429 B351 DIA 14
## 430 B378 DIA 14
## 431 C900 DIA 14
## 432 D376 DIA 14
## 433 G919 DIA 14
## 434 I152 DIA 14
## 435 J168 DIA 14
## 436 J631 DIA 14
## 437 K351 DIA 14
## 438 N130 DIA 14
## 439 N47X DIA 14
## 440 T054 DIA 14
## 441 B009 DIA 13
## 442 B354 DIA 13
## 443 B465 DIA 13
## 444 E220 DIA 13
## 445 E271 DIA 13
## 446 F011 DIA 13
## 447 G042 DIA 13
## 448 H259 DIA 13
## 449 I250 DIA 13
## 450 I350 DIA 13
## 451 I611 DIA 13
## 452 J450 DIA 13
## 453 J848 DIA 13
## 454 J939 DIA 13
## 455 K318 DIA 13
## 456 K402 DIA 13
## 457 K660 DIA 13
## 458 K929 DIA 13
## 459 L080 DIA 13
## 460 L309 DIA 13
## 461 N219 DIA 13
## 462 N450 DIA 13
## 463 O141 DIA 13
## 464 R42X DIA 13
## 465 R55X DIA 13
## 466 S619 DIA 13
## 467 S820 DIA 13
## 468 C221 DIA 12
## 469 D391 DIA 12
## 470 G400 DIA 12
## 471 G589 DIA 12
## 472 H919 DIA 12
## 473 I252 DIA 12
## 474 I516 DIA 12
## 475 I613 DIA 12
## 476 I674 DIA 12
## 477 J039 DIA 12
## 478 J948 DIA 12
## 479 K046 DIA 12
## 480 K352 DIA 12
## 481 K353 DIA 12
## 482 K632 DIA 12
## 483 K719 DIA 12
## 484 K759 DIA 12
## 485 K803 DIA 12
## 486 K839 DIA 12
## 487 M600 DIA 12
## 488 M866 DIA 12
## 489 N281 DIA 12
## 490 N328 DIA 12
## 491 N818 DIA 12
## 492 N872 DIA 12
## 493 O200 DIA 12
## 494 O364 DIA 12
## 495 S983 DIA 12
## 496 T302 DIA 12
## 497 A480 DIA 11
## 498 G401 DIA 11
## 499 G932 DIA 11
## 500 I059 DIA 11
## 501 I130 DIA 11
## 502 I258 DIA 11
## 503 I803 DIA 11
## 504 J157 DIA 11
## 505 J850 DIA 11
## 506 K260 DIA 11
## 507 L892 DIA 11
## 508 M321 DIA 11
## 509 M729 DIA 11
## 510 N840 DIA 11
## 511 R53X DIA 11
## 512 R578 DIA 11
## 513 S610 DIA 11
## 514 S682 DIA 11
## 515 S724 DIA 11
## 516 T888 DIA 11
## 517 43552 DIA 10
## 518 B461 DIA 10
## 519 E02X DIA 10
## 520 E041 DIA 10
## 521 E050 DIA 10
## 522 E240 DIA 10
## 523 F009 DIA 10
## 524 F410 DIA 10
## 525 I211 DIA 10
## 526 I48 DIA 10
## 527 I481 DIA 10
## 528 I99X DIA 10
## 529 J36X DIA 10
## 530 J410 DIA 10
## 531 J949 DIA 10
## 532 K221 DIA 10
## 533 K294 DIA 10
## 534 K721 DIA 10
## 535 K740 DIA 10
## 536 K804 DIA 10
## 537 M100 DIA 10
## 538 M431 DIA 10
## 539 M868 DIA 10
## 540 N202 DIA 10
## 541 N394 DIA 10
## 542 N709 DIA 10
## 543 N830 DIA 10
## 544 O149 DIA 10
## 545 O800 DIA 10
## 546 S223 DIA 10
## 547 S422 DIA 10
## 548 T600 DIA 10
## 549 43553 DIA 9
## 550 B353 DIA 9
## 551 D069 DIA 9
## 552 E230 DIA 9
## 553 F100 DIA 9
## 554 F449 DIA 9
## 555 G619 DIA 9
## 556 H650 DIA 9
## 557 I213 DIA 9
## 558 I270 DIA 9
## 559 I479 DIA 9
## 560 I600 DIA 9
## 561 I775 DIA 9
## 562 I776 DIA 9
## 563 I800 DIA 9
## 564 J860 DIA 9
## 565 K219 DIA 9
## 566 K270 DIA 9
## 567 K350 DIA 9
## 568 K579 DIA 9
## 569 K612 DIA 9
## 570 K625 DIA 9
## 571 K658 DIA 9
## 572 K768 DIA 9
## 573 L891 DIA 9
## 574 M793 DIA 9
## 575 M819 DIA 9
## 576 M861 DIA 9
## 577 N009 DIA 9
## 578 N398 DIA 9
## 579 R074 DIA 9
## 580 R392 DIA 9
## 581 R54X DIA 9
## 582 R64X DIA 9
## 583 R69X DIA 9
## 584 S062 DIA 9
## 585 S328 DIA 9
## 586 S411 DIA 9
## 587 S525 DIA 9
## 588 S920 DIA 9
## 589 T243 DIA 9
## 590 Z488 DIA 9
## 591 Z519 DIA 9
## 592 A064 DIA 8
## 593 A279 DIA 8
## 594 D589 DIA 8
## 595 F050 DIA 8
## 596 F130 DIA 8
## 597 F320 DIA 8
## 598 F328 DIA 8
## 599 G009 DIA 8
## 600 G060 DIA 8
## 601 G360 DIA 8
## 602 G402 DIA 8
## 603 G403 DIA 8
## 604 G410 DIA 8
## 605 G569 DIA 8
## 606 G92X DIA 8
## 607 G931 DIA 8
## 608 G936 DIA 8
## 609 H660 DIA 8
## 610 I051 DIA 8
## 611 I459 DIA 8
## 612 I460 DIA 8
## 613 I498 DIA 8
## 614 I631 DIA 8
## 615 I64 DIA 8
## 616 I770 DIA 8
## 617 I801 DIA 8
## 618 J128 DIA 8
## 619 J14X DIA 8
## 620 K210 DIA 8
## 621 K319 DIA 8
## 622 K648 DIA 8
## 623 K861 DIA 8
## 624 L500 DIA 8
## 625 M169 DIA 8
## 626 M255 DIA 8
## 627 M331 DIA 8
## 628 M490 DIA 8
## 629 M512 DIA 8
## 630 M541 DIA 8
## 631 M549 DIA 8
## 632 N000 DIA 8
## 633 N059 DIA 8
## 634 N210 DIA 8
## 635 N481 DIA 8
## 636 N870 DIA 8
## 637 N952 DIA 8
## 638 O210 DIA 8
## 639 O249 DIA 8
## 640 R000 DIA 8
## 641 S810 DIA 8
## 642 S817 DIA 8
## 643 S910 DIA 8
## 644 T140 DIA 8
## 645 T242 DIA 8
## 646 T857 DIA 8
## 647 43564 DIA 7
## 648 43566 DIA 7
## 649 D590 DIA 7
## 650 D609 DIA 7
## 651 E242 DIA 7
## 652 E873 DIA 7
## 653 E874 DIA 7
## 654 F09X DIA 7
## 655 F432 DIA 7
## 656 G939 DIA 7
## 657 H350 DIA 7
## 658 I229 DIA 7
## 659 I429 DIA 7
## 660 I441 DIA 7
## 661 J040 DIA 7
## 662 J441 DIA 7
## 663 J680 DIA 7
## 664 J930 DIA 7
## 665 J938 DIA 7
## 666 J942 DIA 7
## 667 J988 DIA 7
## 668 K299 DIA 7
## 669 K449 DIA 7
## 670 K567 DIA 7
## 671 K630 DIA 7
## 672 K766 DIA 7
## 673 K928 DIA 7
## 674 L100 DIA 7
## 675 L719 DIA 7
## 676 L959 DIA 7
## 677 L989 DIA 7
## 678 M053 DIA 7
## 679 M150 DIA 7
## 680 M479 DIA 7
## 681 M609 DIA 7
## 682 N029 DIA 7
## 683 N158 DIA 7
## 684 N288 DIA 7
## 685 N320 DIA 7
## 686 N411 DIA 7
## 687 N751 DIA 7
## 688 N809 DIA 7
## 689 N810 DIA 7
## 690 N879 DIA 7
## 691 N911 DIA 7
## 692 O689 DIA 7
## 693 Q667 DIA 7
## 694 R402 DIA 7
## 695 R410 DIA 7
## 696 R529 DIA 7
## 697 R560 DIA 7
## 698 R58X DIA 7
## 699 S202 DIA 7
## 700 T253 DIA 7
## 701 T827 DIA 7
## 702 W199 DIA 7
## 703 43549 DIA 6
## 704 43556 DIA 6
## 705 43565 DIA 6
## 706 B022 DIA 6
## 707 C329 DIA 6
## 708 D414 DIA 6
## 709 E049 DIA 6
## 710 E538 DIA 6
## 711 F059 DIA 6
## 712 F064 DIA 6
## 713 F067 DIA 6
## 714 F069 DIA 6
## 715 F323 DIA 6
## 716 G039 DIA 6
## 717 G219 DIA 6
## 718 G373 DIA 6
## 719 G439 DIA 6
## 720 G628 DIA 6
## 721 G959 DIA 6
## 722 H601 DIA 6
## 723 H609 DIA 6
## 724 I060 DIA 6
## 725 I440 DIA 6
## 726 I447 DIA 6
## 727 I601 DIA 6
## 728 I671 DIA 6
## 729 I688 DIA 6
## 730 I729 DIA 6
## 731 I831 DIA 6
## 732 I891 DIA 6
## 733 J019 DIA 6
## 734 K102 DIA 6
## 735 K257 DIA 6
## 736 K565 DIA 6
## 737 K605 DIA 6
## 738 K717 DIA 6
## 739 K720 DIA 6
## 740 K828 DIA 6
## 741 K829 DIA 6
## 742 M131 DIA 6
## 743 M809 DIA 6
## 744 N048 DIA 6
## 745 N132 DIA 6
## 746 N201 DIA 6
## 747 N359 DIA 6
## 748 N433 DIA 6
## 749 O020 DIA 6
## 750 O021 DIA 6
## 751 O140 DIA 6
## 752 O230 DIA 6
## 753 R092 DIA 6
## 754 R600 DIA 6
## 755 S009 DIA 6
## 756 S010 DIA 6
## 757 S099 DIA 6
## 758 S701 DIA 6
## 759 S821 DIA 6
## 760 S829 DIA 6
## 761 T630 DIA 6
## 762 T824 DIA 6
## 763 43558 DIA 5
## 764 43571 DIA 5
## 765 E222 DIA 5
## 766 E784 DIA 5
## 767 F058 DIA 5
## 768 F101 DIA 5
## 769 F103 DIA 5
## 770 F109 DIA 5
## 771 F339 DIA 5
## 772 F341 DIA 5
## 773 F448 DIA 5
## 774 G041 DIA 5
## 775 G700 DIA 5
## 776 G938 DIA 5
## 777 H059 DIA 5
## 778 H109 DIA 5
## 779 H659 DIA 5
## 780 H663 DIA 5
## 781 H810 DIA 5
## 782 H900 DIA 5
## 783 H903 DIA 5
## 784 I050 DIA 5
## 785 I131 DIA 5
## 786 I260 DIA 5
## 787 I517 DIA 5
## 788 I614 DIA 5
## 789 I660 DIA 5
## 790 I691 DIA 5
## 791 I700 DIA 5
## 792 I709 DIA 5
## 793 I738 DIA 5
## 794 I744 DIA 5
## 795 I864 DIA 5
## 796 J342 DIA 5
## 797 J679 DIA 5
## 798 J840 DIA 5
## 799 J853 DIA 5
## 800 K113 DIA 5
## 801 K253 DIA 5
## 802 K293 DIA 5
## 803 K298 DIA 5
## 804 K36X DIA 5
## 805 K421 DIA 5
## 806 K649 DIA 5
## 807 K85 DIA 5
## 808 K858 DIA 5
## 809 K863 DIA 5
## 810 L259 DIA 5
## 811 L304 DIA 5
## 812 L539 DIA 5
## 813 M300 DIA 5
## 814 M430 DIA 5
## 815 M480 DIA 5
## 816 M705 DIA 5
## 817 M842 DIA 5
## 818 M879 DIA 5
## 819 N050 DIA 5
## 820 N118 DIA 5
## 821 N322 DIA 5
## 822 N340 DIA 5
## 823 N391 DIA 5
## 824 N736 DIA 5
## 825 N738 DIA 5
## 826 N800 DIA 5
## 827 N816 DIA 5
## 828 N820 DIA 5
## 829 N971 DIA 5
## 830 O069 DIA 5
## 831 O600 DIA 5
## 832 R160 DIA 5
## 833 R520 DIA 5
## 834 R99X DIA 5
## 835 S271 DIA 5
## 836 S310 DIA 5
## 837 S311 DIA 5
## 838 S324 DIA 5
## 839 S399 DIA 5
## 840 S611 DIA 5
## 841 S700 DIA 5
## 842 S718 DIA 5
## 843 S722 DIA 5
## 844 S823 DIA 5
## 845 T059 DIA 5
## 846 T07X DIA 5
## 847 T10X DIA 5
## 848 T634 DIA 5
## 849 T659 DIA 5
## 850 T702 DIA 5
## 851 43540 DIA 4
## 852 43547 DIA 4
## 853 43554 DIA 4
## 854 43559 DIA 4
## 855 43577 DIA 4
## 856 43580 DIA 4
## 857 A521 DIA 4
## 858 E031 DIA 4
## 859 E272 DIA 4
## 860 E559 DIA 4
## 861 E700 DIA 4
## 862 E739 DIA 4
## 863 E755 DIA 4
## 864 E756 DIA 4
## 865 E831 DIA 4
## 866 F319 DIA 4
## 867 F411 DIA 4
## 868 F609 DIA 4
## 869 G01X DIA 4
## 870 G048 DIA 4
## 871 G050 DIA 4
## 872 G35X DIA 4
## 873 G464 DIA 4
## 874 G500 DIA 4
## 875 G530 DIA 4
## 876 G579 DIA 4
## 877 G588 DIA 4
## 878 G618 DIA 4
## 879 G822 DIA 4
## 880 G98X DIA 4
## 881 H024 DIA 4
## 882 H043 DIA 4
## 883 H268 DIA 4
## 884 H368 DIA 4
## 885 H490 DIA 4
## 886 H588 DIA 4
## 887 H654 DIA 4
## 888 H664 DIA 4
## 889 H701 DIA 4
## 890 I071 DIA 4
## 891 I139 DIA 4
## 892 I278 DIA 4
## 893 I451 DIA 4
## 894 I461 DIA 4
## 895 I482 DIA 4
## 896 I501 DIA 4
## 897 I778 DIA 4
## 898 I779 DIA 4
## 899 I822 DIA 4
## 900 J101 DIA 4
## 901 J139 DIA 4
## 902 J170 DIA 4
## 903 J386 DIA 4
## 904 J390 DIA 4
## 905 J61X DIA 4
## 906 k259 DIA 4
## 907 K273 DIA 4
## 908 K602 DIA 4
## 909 K623 DIA 4
## 910 K631 DIA 4
## 911 K661 DIA 4
## 912 K700 DIA 4
## 913 K704 DIA 4
## 914 K739 DIA 4
## 915 K743 DIA 4
## 916 K818 DIA 4
## 917 K823 DIA 4
## 918 K831 DIA 4
## 919 K860 DIA 4
## 920 K868 DIA 4
## 921 L102 DIA 4
## 922 L219 DIA 4
## 923 L281 DIA 4
## 924 L401 DIA 4
## 925 L405 DIA 4
## 926 L408 DIA 4
## 927 L729 DIA 4
## 928 M029 DIA 4
## 929 M130 DIA 4
## 930 M146 DIA 4
## 931 M542 DIA 4
## 932 M779 DIA 4
## 933 M860 DIA 4
## 934 M932 DIA 4
## 935 N131 DIA 4
## 936 N211 DIA 4
## 937 N311 DIA 4
## 938 N72X DIA 4
## 939 N871 DIA 4
## 940 O429 DIA 4
## 941 O479 DIA 4
## 942 O758 DIA 4
## 943 O828 DIA 4
## 944 Q249 DIA 4
## 945 Q610 DIA 4
## 946 Q909 DIA 4
## 947 R13X DIA 4
## 948 R190 DIA 4
## 949 R221 DIA 4
## 950 R960 DIA 4
## 951 S008 DIA 4
## 952 S019 DIA 4
## 953 S024 DIA 4
## 954 S066 DIA 4
## 955 S320 DIA 4
## 956 S424 DIA 4
## 957 S520 DIA 4
## 958 S626 DIA 4
## 959 S628 DIA 4
## 960 S711 DIA 4
## 961 S826 DIA 4
## 962 S925 DIA 4
## 963 S929 DIA 4
## 964 T019 DIA 4
## 965 T149 DIA 4
## 966 T424 DIA 4
## 967 T519 DIA 4
## 968 T825 DIA 4
## 969 T856 DIA 4
## 970 T905 DIA 4
## 971 W019 DIA 4
## 972 43519 DIA 3
## 973 43550 DIA 3
## 974 43560 DIA 3
## 975 43561 DIA 3
## 976 43563 DIA 3
## 977 43570 DIA 3
## 978 43572 DIA 3
## 979 A083 DIA 3
## 980 C166 DIA 3
## 981 C959 DIA 3
## 982 D151 DIA 3
## 983 D591 DIA 3
## 984 E042 DIA 3
## 985 E052 DIA 3
## 986 E069 DIA 3
## 987 E15X DIA 3
## 988 E209 DIA 3
## 989 E210 DIA 3
## 990 E229 DIA 3
## 991 E232 DIA 3
## 992 E236 DIA 3
## 993 E270 DIA 3
## 994 E66 DIA 3
## 995 E788 DIA 3
## 996 E888 DIA 3
## 997 F018 DIA 3
## 998 F062 DIA 3
## 999 F078 DIA 3
## 1000 F142 DIA 3
## 1001 F172 DIA 3
## 1002 F192 DIA 3
## 1003 F238 DIA 3
## 1004 F29X DIA 3
## 1005 F445 DIA 3
## 1006 F450 DIA 3
## 1007 F799 DIA 3
## 1008 G020 DIA 3
## 1009 G062 DIA 3
## 1010 G08X DIA 3
## 1011 G09X DIA 3
## 1012 G122 DIA 3
## 1013 G311 DIA 3
## 1014 G419 DIA 3
## 1015 G430 DIA 3
## 1016 G448 DIA 3
## 1017 G450 DIA 3
## 1018 G458 DIA 3
## 1019 G531 DIA 3
## 1020 G578 DIA 3
## 1021 G609 DIA 3
## 1022 G729 DIA 3
## 1023 G825 DIA 3
## 1024 G952 DIA 3
## 1025 H010 DIA 3
## 1026 H103 DIA 3
## 1027 H110 DIA 3
## 1028 H352 DIA 3
## 1029 H358 DIA 3
## 1030 H400 DIA 3
## 1031 H440 DIA 3
## 1032 H527 DIA 3
## 1033 H544 DIA 3
## 1034 H549 DIA 3
## 1035 H602 DIA 3
## 1036 H651 DIA 3
## 1037 H653 DIA 3
## 1038 I069 DIA 3
## 1039 I089 DIA 3
## 1040 I132 DIA 3
## 1041 I212 DIA 3
## 1042 I241 DIA 3
## 1043 I248 DIA 3
## 1044 I309 DIA 3
## 1045 I330 DIA 3
## 1046 I339 DIA 3
## 1047 I358 DIA 3
## 1048 I378 DIA 3
## 1049 I38X DIA 3
## 1050 I390 DIA 3
## 1051 I421 DIA 3
## 1052 I446 DIA 3
## 1053 I495 DIA 3
## 1054 I510 DIA 3
## 1055 I528 DIA 3
## 1056 I602 DIA 3
## 1057 I606 DIA 3
## 1058 I615 DIA 3
## 1059 I670 DIA 3
## 1060 I690 DIA 3
## 1061 I728 DIA 3
## 1062 I742 DIA 3
## 1063 I821 DIA 3
## 1064 I842 DIA 3
## 1065 I890 DIA 3
## 1066 I899 DIA 3
## 1067 I982 DIA 3
## 1068 J014 DIA 3
## 1069 J155 DIA 3
## 1070 J200 DIA 3
## 1071 J340 DIA 3
## 1072 J380 DIA 3
## 1073 J393 DIA 3
## 1074 J451 DIA 3
## 1075 J64X DIA 3
## 1076 J941 DIA 3
## 1077 J998 DIA 3
## 1078 K029 DIA 3
## 1079 K041 DIA 3
## 1080 K044 DIA 3
## 1081 K052 DIA 3
## 1082 K228 DIA 3
## 1083 K316 DIA 3
## 1084 K388 DIA 3
## 1085 K458 DIA 3
## 1086 K461 DIA 3
## 1087 K519 DIA 3
## 1088 K550 DIA 3
## 1089 K559 DIA 3
## 1090 K570 DIA 3
## 1091 K626 DIA 3
## 1092 K628 DIA 3
## 1093 K639 DIA 3
## 1094 K701 DIA 3
## 1095 K702 DIA 3
## 1096 K710 DIA 3
## 1097 K712 DIA 3
## 1098 K744 DIA 3
## 1099 K758 DIA 3
## 1100 K820 DIA 3
## 1101 K833 DIA 3
## 1102 K838 DIA 3
## 1103 K862 DIA 3
## 1104 K869 DIA 3
## 1105 K904 DIA 3
## 1106 K914 DIA 3
## 1107 K915 DIA 3
## 1108 K921 DIA 3
## 1109 L00X DIA 3
## 1110 L010 DIA 3
## 1111 L043 DIA 3
## 1112 L239 DIA 3
## 1113 L402 DIA 3
## 1114 L930 DIA 3
## 1115 L988 DIA 3
## 1116 M050 DIA 3
## 1117 M068 DIA 3
## 1118 M073 DIA 3
## 1119 M125 DIA 3
## 1120 M154 DIA 3
## 1121 M171 DIA 3
## 1122 M189 DIA 3
## 1123 M254 DIA 3
## 1124 M311 DIA 3
## 1125 M313 DIA 3
## 1126 M465 DIA 3
## 1127 M489 DIA 3
## 1128 M543 DIA 3
## 1129 M620 DIA 3
## 1130 M623 DIA 3
## 1131 M624 DIA 3
## 1132 M659 DIA 3
## 1133 M712 DIA 3
## 1134 M728 DIA 3
## 1135 M790 DIA 3
## 1136 M844 DIA 3
## 1137 M863 DIA 3
## 1138 M864 DIA 3
## 1139 M870 DIA 3
## 1140 M878 DIA 3
## 1141 M939 DIA 3
## 1142 N020 DIA 3
## 1143 N038 DIA 3
## 1144 N079 DIA 3
## 1145 N137 DIA 3
## 1146 N138 DIA 3
## 1147 N144 DIA 3
## 1148 N258 DIA 3
## 1149 N350 DIA 3
## 1150 N44X DIA 3
## 1151 N512 DIA 3
## 1152 N700 DIA 3
## 1153 N701 DIA 3
## 1154 N710 DIA 3
## 1155 N750 DIA 3
## 1156 N758 DIA 3
## 1157 N761 DIA 3
## 1158 N778 DIA 3
## 1159 N835 DIA 3
## 1160 N851 DIA 3
## 1161 N859 DIA 3
## 1162 N898 DIA 3
## 1163 N990 DIA 3
## 1164 O009 DIA 3
## 1165 O100 DIA 3
## 1166 O16X DIA 3
## 1167 O268 DIA 3
## 1168 O335 DIA 3
## 1169 O367 DIA 3
## 1170 O441 DIA 3
## 1171 O649 DIA 3
## 1172 O669 DIA 3
## 1173 O860 DIA 3
## 1174 O992 DIA 3
## 1175 Q250 DIA 3
## 1176 Q501 DIA 3
## 1177 R030 DIA 3
## 1178 R049 DIA 3
## 1179 R05X DIA 3
## 1180 R060 DIA 3
## 1181 R070 DIA 3
## 1182 R098 DIA 3
## 1183 R162 DIA 3
## 1184 R488 DIA 3
## 1185 R522 DIA 3
## 1186 R590 DIA 3
## 1187 R650 DIA 3
## 1188 R730 DIA 3
## 1189 R828 DIA 3
## 1190 S064 DIA 3
## 1191 S109 DIA 3
## 1192 S210 DIA 3
## 1193 S224 DIA 3
## 1194 S299 DIA 3
## 1195 S300 DIA 3
## 1196 S313 DIA 3
## 1197 S321 DIA 3
## 1198 S420 DIA 3
## 1199 S430 DIA 3
## 1200 S518 DIA 3
## 1201 S522 DIA 3
## 1202 S618 DIA 3
## 1203 S683 DIA 3
## 1204 S728 DIA 3
## 1205 S730 DIA 3
## 1206 S780 DIA 3
## 1207 S824 DIA 3
## 1208 T116 DIA 3
## 1209 T143 DIA 3
## 1210 T240 DIA 3
## 1211 T301 DIA 3
## 1212 T303 DIA 3
## 1213 T310 DIA 3
## 1214 T477 DIA 3
## 1215 T509 DIA 3
## 1216 T543 DIA 3
## 1217 T740 DIA 3
## 1218 T784 DIA 3
## 1219 U069 DIA 3
## 1220 W179 DIA 3
## 1221 Y919 DIA 3
## 1222 Z540 DIA 3
## 1223 Z608 DIA 3
## 1224 Z950 DIA 3
## 1225 43498 DIA 2
## 1226 43501 I10X 2
## 1227 43509 DIA 2
## 1228 43525 S889 2
## 1229 43526 I509 2
## 1230 43530 I10X 2
## 1231 43541 I10X 2
## 1232 43551 N390 2
## 1233 43555 DIA 2
## 1234 43562 I10X 2
## 1235 43567 DIA 2
## 1236 43568 E86X 2
## 1237 43569 N390 2
## 1238 43574 DIA 2
## 1239 43579 DIA 2
## 1240 A58X DIA 2
## 1241 A852 DIA 2
## 1242 B878 DIA 2
## 1243 C258 DIA 2
## 1244 C310 DIA 2
## 1245 C348 DIA 2
## 1246 C540 DIA 2
## 1247 C840 DIA 2
## 1248 D042 DIA 2
## 1249 D061 DIA 2
## 1250 D521 DIA 2
## 1251 D642 DIA 2
## 1252 E000 DIA 2
## 1253 E010 DIA 2
## 1254 E012 DIA 2
## 1255 E032 DIA 2
## 1256 E033 DIA 2
## 1257 E034 DIA 2
## 1258 E055 DIA 2
## 1259 E168 DIA 2
## 1260 E200 DIA 2
## 1261 E248 DIA 2
## 1262 E273 DIA 2
## 1263 E274 DIA 2
## 1264 E308 DIA 2
## 1265 E40X DIA 2
## 1266 E45X DIA 2
## 1267 E512 DIA 2
## 1268 E639 DIA 2
## 1269 E662 DIA 2
## 1270 E713 DIA 2
## 1271 E722 DIA 2
## 1272 E748 DIA 2
## 1273 E786 DIA 2
## 1274 E790 DIA 2
## 1275 E832 DIA 2
## 1276 E839 DIA 2
## 1277 E840 DIA 2
## 1278 E849 DIA 2
## 1279 E859 DIA 2
## 1280 E890 DIA 2
## 1281 F002 DIA 2
## 1282 F010 DIA 2
## 1283 F012 DIA 2
## 1284 F013 DIA 2
## 1285 F028 DIA 2
## 1286 F072 DIA 2
## 1287 F104 DIA 2
## 1288 F105 DIA 2
## 1289 F132 DIA 2
## 1290 F190 DIA 2
## 1291 F199 DIA 2
## 1292 F201 DIA 2
## 1293 F203 DIA 2
## 1294 F206 DIA 2
## 1295 F230 DIA 2
## 1296 F232 DIA 2
## 1297 F250 DIA 2
## 1298 F332 DIA 2
## 1299 F409 DIA 2
## 1300 F429 DIA 2
## 1301 F606 DIA 2
## 1302 F639 DIA 2
## 1303 F709 DIA 2
## 1304 F721 DIA 2
## 1305 F811 DIA 2
## 1306 F99X DIA 2
## 1307 G008 DIA 2
## 1308 G030 DIA 2
## 1309 G032 DIA 2
## 1310 G040 DIA 2
## 1311 G051 DIA 2
## 1312 G058 DIA 2
## 1313 G061 DIA 2
## 1314 G10X DIA 2
## 1315 G131 DIA 2
## 1316 G249 DIA 2
## 1317 G253 DIA 2
## 1318 G300 DIA 2
## 1319 G310 DIA 2
## 1320 G328 DIA 2
## 1321 G405 DIA 2
## 1322 G432 DIA 2
## 1323 G509 DIA 2
## 1324 G519 DIA 2
## 1325 G529 DIA 2
## 1326 G540 DIA 2
## 1327 G611 DIA 2
## 1328 G633 DIA 2
## 1329 G638 DIA 2
## 1330 G709 DIA 2
## 1331 G710 DIA 2
## 1332 G802 DIA 2
## 1333 G808 DIA 2
## 1334 G820 DIA 2
## 1335 G903 DIA 2
## 1336 G911 DIA 2
## 1337 G912 DIA 2
## 1338 G930 DIA 2
## 1339 G948 DIA 2
## 1340 G998 B690 2
## 1341 H062 DIA 2
## 1342 H160 DIA 2
## 1343 H162 DIA 2
## 1344 H193 DIA 2
## 1345 H359 DIA 2
## 1346 H431 DIA 2
## 1347 H600 DIA 2
## 1348 H603 DIA 2
## 1349 H620 DIA 2
## 1350 H622 DIA 2
## 1351 H652 DIA 2
## 1352 H700 DIA 2
## 1353 H708 DIA 2
## 1354 H709 DIA 2
## 1355 H730 DIA 2
## 1356 H812 DIA 2
## 1357 H902 DIA 2
## 1358 I052 DIA 2
## 1359 I061 DIA 2
## 1360 I080 DIA 2
## 1361 I201 DIA 2
## 1362 I208 DIA 2
## 1363 I240 DIA 2
## 1364 I288 DIA 2
## 1365 I289 DIA 2
## 1366 I311 DIA 2
## 1367 I313 DIA 2
## 1368 I319 DIA 2
## 1369 I351 DIA 2
## 1370 I352 DIA 2
## 1371 I379 DIA 2
## 1372 I438 DIA 2
## 1373 I452 DIA 2
## 1374 I454 DIA 2
## 1375 I472 DIA 2
## 1376 I604 DIA 2
## 1377 I659 R568 2
## 1378 I663 DIA 2
## 1379 I692 DIA 2
## 1380 I719 DIA 2
## 1381 I724 DIA 2
## 1382 I741 DIA 2
## 1383 I748 DIA 2
## 1384 I808 DIA 2
## 1385 I848 DIA 2
## 1386 I849 DIA 2
## 1387 I868 DIA 2
## 1388 I871 DIA 2
## 1389 I879 DIA 2
## 1390 I951 DIA 2
## 1391 I983 DIA 2
## 1392 J010 DIA 2
## 1393 J042 DIA 2
## 1394 J118 DIA 2
## 1395 J15 E11 2
## 1396 J153 DIA 2
## 1397 J154 DIA 2
## 1398 J182 DIA 2
## 1399 J20 DIA 2
## 1400 J210 DIA 2
## 1401 J304 DIA 2
## 1402 J329 DIA 2
## 1403 J371 DIA 2
## 1404 J398 DIA 2
## 1405 J438 DIA 2
## 1406 J458 DIA 2
## 1407 J633 DIA 2
## 1408 J691 DIA 2
## 1409 J931 DIA 2
## 1410 J951 DIA 2
## 1411 J958 DIA 2
## 1412 K040 DIA 2
## 1413 K050 DIA 2
## 1414 K112 DIA 2
## 1415 K222 DIA 2
## 1416 K225 DIA 2
## 1417 K251 DIA 2
## 1418 K254 DIA 2
## 1419 K264 DIA 2
## 1420 K292 DIA 2
## 1421 K310 DIA 2
## 1422 K317 DIA 2
## 1423 K381 DIA 2
## 1424 K389 DIA 2
## 1425 K403 DIA 2
## 1426 K404 DIA 2
## 1427 K419 DIA 2
## 1428 K440 DIA 2
## 1429 K450 DIA 2
## 1430 K460 DIA 2
## 1431 K500 DIA 2
## 1432 K522 DIA 2
## 1433 K563 DIA 2
## 1434 K572 DIA 2
## 1435 K573 DIA 2
## 1436 K574 DIA 2
## 1437 K589 DIA 2
## 1438 K627 DIA 2
## 1439 K635 DIA 2
## 1440 K638 DIA 2
## 1441 K711 DIA 2
## 1442 K718 DIA 2
## 1443 K767 DIA 2
## 1444 K80 DIA 2
## 1445 K834 DIA 2
## 1446 K900 DIA 2
## 1447 K913 DIA 2
## 1448 K918 DIA 2
## 1449 L040 DIA 2
## 1450 L048 DIA 2
## 1451 L10 DIA 2
## 1452 L209 DIA 2
## 1453 L270 A150 2
## 1454 L301 DIA 2
## 1455 L308 DIA 2
## 1456 L510 DIA 2
## 1457 L511 DIA 2
## 1458 L512 DIA 2
## 1459 L739 DIA 2
## 1460 L921 DIA 2
## 1461 L958 DIA 2
## 1462 L982 DIA 2
## 1463 L983 DIA 2
## 1464 M000 DIA 2
## 1465 M013 DIA 2
## 1466 M058 DIA 2
## 1467 M060 DIA 2
## 1468 M104 DIA 2
## 1469 M148 DIA 2
## 1470 M160 DIA 2
## 1471 M190 DIA 2
## 1472 M318 DIA 2
## 1473 M319 DIA 2
## 1474 M328 DIA 2
## 1475 M330 DIA 2
## 1476 M340 DIA 2
## 1477 M348 DIA 2
## 1478 M349 DIA 2
## 1479 M353 DIA 2
## 1480 M354 DIA 2
## 1481 M359 DIA 2
## 1482 M360 DIA 2
## 1483 M411 DIA 2
## 1484 M45X DIA 2
## 1485 M478 DIA 2
## 1486 M494 DIA 2
## 1487 M511 DIA 2
## 1488 M531 DIA 2
## 1489 M625 DIA 2
## 1490 M626 DIA 2
## 1491 M704 DIA 2
## 1492 M715 DIA 2
## 1493 M720 DIA 2
## 1494 M722 DIA 2
## 1495 M723 DIA 2
## 1496 M724 DIA 2
## 1497 M755 DIA 2
## 1498 M795 DIA 2
## 1499 M899 DIA 2
## 1500 M940 DIA 2
## 1501 N030 DIA 2
## 1502 N069 DIA 2
## 1503 N078 DIA 2
## 1504 N080 DIA 2
## 1505 N136 DIA 2
## 1506 N20 DIA 2
## 1507 N228 DIA 2
## 1508 N251 DIA 2
## 1509 N290 DIA 2
## 1510 N301 DIA 2
## 1511 N318 DIA 2
## 1512 N321 DIA 2
## 1513 N368 DIA 2
## 1514 N392 DIA 2
## 1515 N40 DIA 2
## 1516 N434 DIA 2
## 1517 N482 DIA 2
## 1518 N485 DIA 2
## 1519 N508 DIA 2
## 1520 N62X DIA 2
## 1521 N63X DIA 2
## 1522 N649 DIA 2
## 1523 N711 DIA 2
## 1524 N719 DIA 2
## 1525 N748 DIA 2
## 1526 N763 DIA 2
## 1527 N766 DIA 2
## 1528 N770 DIA 2
## 1529 N829 DIA 2
## 1530 N841 DIA 2
## 1531 N842 DIA 2
## 1532 N857 DIA 2
## 1533 N888 DIA 2
## 1534 N900 DIA 2
## 1535 N910 DIA 2
## 1536 N912 DIA 2
## 1537 N979 DIA 2
## 1538 N993 DIA 2
## 1539 N994 DIA 2
## 1540 O13X DIA 2
## 1541 O235 DIA 2
## 1542 O269 DIA 2
## 1543 O321 DIA 2
## 1544 O366 DIA 2
## 1545 O40X DIA 2
## 1546 O410 DIA 2
## 1547 O601 DIA 2
## 1548 O60X DIA 2
## 1549 O639 DIA 2
## 1550 O730 DIA 2
## 1551 O731 DIA 2
## 1552 O839 DIA 2
## 1553 O911 DIA 2
## 1554 O912 DIA 2
## 1555 Q211 DIA 2
## 1556 Q282 DIA 2
## 1557 Q283 DIA 2
## 1558 Q444 DIA 2
## 1559 Q505 DIA 2
## 1560 Q803 DIA 2
## 1561 R066 DIA 2
## 1562 R073 DIA 2
## 1563 R091 DIA 2
## 1564 R14X DIA 2
## 1565 R229 DIA 2
## 1566 R34X DIA 2
## 1567 R35X DIA 2
## 1568 R36X DIA 2
## 1569 R450 DIA 2
## 1570 R451 DIA 2
## 1571 R458 DIA 2
## 1572 R490 DIA 2
## 1573 R634 DIA 2
## 1574 R740 DIA 2
## 1575 R820 DIA 2
## 1576 R827 DIA 2
## 1577 S001 DIA 2
## 1578 S027 DIA 2
## 1579 S029 DIA 2
## 1580 S051 DIA 2
## 1581 S059 DIA 2
## 1582 S089 DIA 2
## 1583 S219 DIA 2
## 1584 S220 DIA 2
## 1585 S272 DIA 2
## 1586 S279 DIA 2
## 1587 S301 DIA 2
## 1588 S309 DIA 2
## 1589 S322 DIA 2
## 1590 S323 DIA 2
## 1591 S364 DIA 2
## 1592 S369 DIA 2
## 1593 S370 DIA 2
## 1594 S421 DIA 2
## 1595 S480 DIA 2
## 1596 S489 DIA 2
## 1597 S526 DIA 2
## 1598 S609 DIA 2
## 1599 S627 DIA 2
## 1600 S670 DIA 2
## 1601 S699 DIA 2
## 1602 S749 DIA 2
## 1603 S770 DIA 2
## 1604 S808 DIA 2
## 1605 S827 DIA 2
## 1606 S842 DIA 2
## 1607 S903 DIA 2
## 1608 S921 DIA 2
## 1609 S930 DIA 2
## 1610 S971 DIA 2
## 1611 S998 DIA 2
## 1612 T008 DIA 2
## 1613 T055 DIA 2
## 1614 T093 DIA 2
## 1615 T111 DIA 2
## 1616 T130 DIA 2
## 1617 T202 DIA 2
## 1618 T222 DIA 2
## 1619 T245 DIA 2
## 1620 T246 DIA 2
## 1621 T250 DIA 2
## 1622 T292 DIA 2
## 1623 T348 DIA 2
## 1624 T451 DIA 2
## 1625 T671 DIA 2
## 1626 T68X DIA 2
## 1627 T709 DIA 2
## 1628 T782 DIA 2
## 1629 T809 DIA 2
## 1630 T829 DIA 2
## 1631 T861 DIA 2
## 1632 T868 DIA 2
## 1633 T871 DIA 2
## 1634 T886 DIA 2
## 1635 T930 DIA 2
## 1636 T983 DIA 2
## 1637 U202 DIA 2
## 1638 W010 DIA 2
## 1639 X259 DIA 2
## 1640 Y579 DIA 2
## 1641 Y841 DIA 2
## 1642 Y912 DIA 2
## 1643 Z100 DIA 2
## 1644 Z359 DIA 2
## 1645 Z433 DIA 2
## 1646 Z896 DIA 2
## 1647 Z992 DIA 2
## 1648 B178 B029 1
## 1649 B358 B24X 1
## 1650 B427 A419 1
## 1651 B450 B24X 1
## 1652 B451 B009 1
## 1653 B779 A071 1
## 1654 C091 A419 1
## 1655 C460 B24X 1
## 1656 C919 C910 1
## 1657 D070 D060 1
## 1658 D199 B370 1
## 1659 D25 C64X 1
## 1660 D339 C719 1
## 1661 D373 A162 1
## 1662 D382 A165 1
## 1663 D823 A419 1
## 1664 E011 DIA 1
## 1665 E030 DIA 1
## 1666 E040 DIA 1
## 1667 E060 DIA 1
## 1668 E063 DIA 1
## 1669 E079 DIA 1
## 1670 E11.6 DIA 1
## 1671 E111D D250 1
## 1672 E208 DIA 1
## 1673 E211 DIA 1
## 1674 E233 DIA 1
## 1675 E237 DIA 1
## 1676 E260 D509 1
## 1677 E282 D432 1
## 1678 E344 DIA 1
## 1679 E348 DIA 1
## 1680 E350 DIA 1
## 1681 E43 C169 1
## 1682 E449 DIA 1
## 1683 E509 DIA 1
## 1684 E539 DIA 1
## 1685 E60X DIA 1
## 1686 E649 A419 1
## 1687 E709 DIA 1
## 1688 E720 DIA 1
## 1689 E728 DIA 1
## 1690 E729 DIA 1
## 1691 E738 D509 1
## 1692 E742 DIA 1
## 1693 E752 DIA 1
## 1694 E768 DIA 1
## 1695 E779 DIA 1
## 1696 E800 DIA 1
## 1697 E804 DIA 1
## 1698 E806 A090 1
## 1699 E807 DIA 1
## 1700 E850 DIA 1
## 1701 E882 DIA 1
## 1702 E893 DIA 1
## 1703 E90X DIA 1
## 1704 F000 DIA 1
## 1705 F020 DIA 1
## 1706 F023 DIA 1
## 1707 F051 DIA 1
## 1708 F068 DIA 1
## 1709 F070 DIA 1
## 1710 F079 DIA 1
## 1711 F106 DIA 1
## 1712 F116 DIA 1
## 1713 F129 DIA 1
## 1714 F135 D509 1
## 1715 F141 DIA 1
## 1716 F171 DIA 1
## 1717 F189 DIA 1
## 1718 F194 DIA 1
## 1719 F20X DIA 1
## 1720 F21X D500 1
## 1721 F229 DIA 1
## 1722 F239 DIA 1
## 1723 F310 DIA 1
## 1724 F311 DIA 1
## 1725 F313 DIA 1
## 1726 F316 DIA 1
## 1727 F330 DIA 1
## 1728 F41 DIA 1
## 1729 F413 DIA 1
## 1730 F418 DIA 1
## 1731 F440 DIA 1
## 1732 F444 DIA 1
## 1733 F459 DIA 1
## 1734 F481 DIA 1
## 1735 F500 DIA 1
## 1736 F504 DIA 1
## 1737 F509 DIA 1
## 1738 F510 DIA 1
## 1739 F514 DIA 1
## 1740 F519 DIA 1
## 1741 F603 DIA 1
## 1742 F604 DIA 1
## 1743 F608 DIA 1
## 1744 F61X DIA 1
## 1745 F669 DIA 1
## 1746 F708 DIA 1
## 1747 F719 DIA 1
## 1748 F729 DIA 1
## 1749 F780 DIA 1
## 1750 F790 DIA 1
## 1751 F800 DIA 1
## 1752 F808 DIA 1
## 1753 F82X DIA 1
## 1754 F844 DIA 1
## 1755 F88X DIA 1
## 1756 F900 DIA 1
## 1757 F980 DIA 1
## 1758 G000 DIA 1
## 1759 G038 B461 1
## 1760 G112 DIA 1
## 1761 G114 DIA 1
## 1762 G129 DIA 1
## 1763 G20 DIA 1
## 1764 G211 DIA 1
## 1765 G218 DIA 1
## 1766 G22X DIA 1
## 1767 G231 DIA 1
## 1768 G250 DIA 1
## 1769 G258 DIA 1
## 1770 G368 DIA 1
## 1771 G369 DIA 1
## 1772 G370 DIA 1
## 1773 G371 DIA 1
## 1774 G372 DIA 1
## 1775 G379 DIA 1
## 1776 G412 DIA 1
## 1777 G418 DIA 1
## 1778 G440 DIA 1
## 1779 G441 DIA 1
## 1780 G444 DIA 1
## 1781 G45 DIA 1
## 1782 G452 DIA 1
## 1783 G460 DIA 1
## 1784 G462 DIA 1
## 1785 G465 D180 1
## 1786 G468 DIA 1
## 1787 G473 DIA 1
## 1788 G528 DIA 1
## 1789 G538 DIA 1
## 1790 G544 DIA 1
## 1791 G560 DIA 1
## 1792 G561 DIA 1
## 1793 G580 A010 1
## 1794 G603 DIA 1
## 1795 G64X DIA 1
## 1796 G713 DIA 1
## 1797 G732 A418 1
## 1798 G800 DIA 1
## 1799 G902 DIA 1
## 1800 G908 DIA 1
## 1801 G918 DIA 1
## 1802 G941 D432 1
## 1803 G951 DIA 1
## 1804 G961 DIA 1
## 1805 G968 DIA 1
## 1806 H000 DIA 1
## 1807 H027 DIA 1
## 1808 H031 B023 1
## 1809 H045 DIA 1
## 1810 H100 DIA 1
## 1811 H105 DIA 1
## 1812 H150 A539 1
## 1813 H151 DIA 1
## 1814 H169 DIA 1
## 1815 H189 C539 1
## 1816 H209 DIA 1
## 1817 H220 A514 1
## 1818 H250 DIA 1
## 1819 H262 DIA 1
## 1820 H282 DIA 1
## 1821 H309 DIA 1
## 1822 H335 A090 1
## 1823 H428 DIA 1
## 1824 H438 D649 1
## 1825 H448 DIA 1
## 1826 H449 DIA 1
## 1827 H451 DIA 1
## 1828 H472 DIA 1
## 1829 H492 DIA 1
## 1830 H493 DIA 1
## 1831 H494 DIA 1
## 1832 H521 DIA 1
## 1833 H523 DIA 1
## 1834 H524 DIA 1
## 1835 H530 DIA 1
## 1836 H533 DIA 1
## 1837 H542 DIA 1
## 1838 H578 DIA 1
## 1839 H598 DIA 1
## 1840 H604 DIA 1
## 1841 H605 DIA 1
## 1842 H60X DIA 1
## 1843 H611 DIA 1
## 1844 H678 DIA 1
## 1845 H818 DIA 1
## 1846 H830 DIA 1
## 1847 H901 A161 1
## 1848 H905 DIA 1
## 1849 H911 DIA 1
## 1850 H913 DIA 1
## 1851 H933 DIA 1
## 1852 I010 DIA 1
## 1853 I018 DIA 1
## 1854 I070 DIA 1
## 1855 I081 DIA 1
## 1856 I091 DIA 1
## 1857 I12 DIA 1
## 1858 I21 D50 1
## 1859 I233 DIA 1
## 1860 I236 DIA 1
## 1861 I253 DIA 1
## 1862 I254 DIA 1
## 1863 I272 DIA 1
## 1864 I28 E11 1
## 1865 I280 DIA 1
## 1866 I300 C300 1
## 1867 I301 DIA 1
## 1868 I321 DIA 1
## 1869 I348 DIA 1
## 1870 I361 DIA 1
## 1871 I369 DIA 1
## 1872 I372 DIA 1
## 1873 I400 DIA 1
## 1874 I409 DIA 1
## 1875 I411 DIA 1
## 1876 I422 DIA 1
## 1877 I424 DIA 1
## 1878 I432 DIA 1
## 1879 I453 DIA 1
## 1880 I456 DIA 1
## 1881 I470 DIA 1
## 1882 I493 DIA 1
## 1883 I513 DIA 1
## 1884 I607 DIA 1
## 1885 I616 DIA 1
## 1886 I636 DIA 1
## 1887 I650 DIA 1
## 1888 I651 DIA 1
## 1889 I652 DIA 1
## 1890 I661 DIA 1
## 1891 I664 DIA 1
## 1892 I669 DIA 1
## 1893 I676 DIA 1
## 1894 I69 DIA 1
## 1895 I708 DIA 1
## 1896 I718 DIA 1
## 1897 I720 DIA 1
## 1898 I721 DIA 1
## 1899 I730 DIA 1
## 1900 I745 DIA 1
## 1901 I772 DIA 1
## 1902 I774 DIA 1
## 1903 I790 DIA 1
## 1904 I81X DIA 1
## 1905 I840 DIA 1
## 1906 I863 DIA 1
## 1907 I870 DIA 1
## 1908 I898 DIA 1
## 1909 I950 DIA 1
## 1910 I988 DIA 1
## 1911 J020 DIA 1
## 1912 J028 DIA 1
## 1913 J030 DIA 1
## 1914 J038 DIA 1
## 1915 J041 DIA 1
## 1916 J06 DIA 1
## 1917 J060 DIA 1
## 1918 J09X DIA 1
## 1919 J110 D509 1
## 1920 J111 DIA 1
## 1921 J122 DIA 1
## 1922 J160 DIA 1
## 1923 J172 DIA 1
## 1924 J173 B59X 1
## 1925 J178 DIA 1
## 1926 J18 E11 1
## 1927 J20X DIA 1
## 1928 J211 DIA 1
## 1929 J22 E11 1
## 1930 J312 DIA 1
## 1931 J320 DIA 1
## 1932 J321 DIA 1
## 1933 J330 DIA 1
## 1934 J339 DIA 1
## 1935 J359 DIA 1
## 1936 J370 DIA 1
## 1937 J382 DIA 1
## 1938 J391 DIA 1
## 1939 J392 DIA 1
## 1940 J40 E11 1
## 1941 J439 D649 1
## 1942 J45 E11 1
## 1943 J45X DIA 1
## 1944 J47 DIA 1
## 1945 J60X DIA 1
## 1946 J620 DIA 1
## 1947 J628 DIA 1
## 1948 J65X DIA 1
## 1949 J671 DIA 1
## 1950 J677 DIA 1
## 1951 J684 DIA 1
## 1952 J950 DIA 1
## 1953 J953 DIA 1
## 1954 J982 DIA 1
## 1955 J986 DIA 1
## 1956 J98O DIA 1
## 1957 J990 DIA 1
## 1958 K021 DIA 1
## 1959 K039 B374 1
## 1960 K051 DIA 1
## 1961 K053 DIA 1
## 1962 K068 DIA 1
## 1963 K088 DIA 1
## 1964 K092 DIA 1
## 1965 K099 DIA 1
## 1966 K103 DIA 1
## 1967 K109 DIA 1
## 1968 K118 DIA 1
## 1969 K119 A09X 1
## 1970 K120 C859 1
## 1971 K121 DIA 1
## 1972 K123 A150 1
## 1973 K20 DIA 1
## 1974 K220 A150 1
## 1975 K226 DIA 1
## 1976 K25 D53 1
## 1977 k250 DIA 1
## 1978 K252 DIA 1
## 1979 K255 DIA 1
## 1980 K256 DIA 1
## 1981 K261 A418 1
## 1982 K263 DIA 1
## 1983 K267 DIA 1
## 1984 K274 DIA 1
## 1985 K275 A419 1
## 1986 K311 D649 1
## 1987 K314 DIA 1
## 1988 K383 DIA 1
## 1989 K40 C61 1
## 1990 K410 DIA 1
## 1991 K430 DIA 1
## 1992 K509 DIA 1
## 1993 K510 DIA 1
## 1994 K515 DIA 1
## 1995 K520 DIA 1
## 1996 K521 DIA 1
## 1997 K528 DIA 1
## 1998 K552 DIA 1
## 1999 K578 DIA 1
## 2000 K580 DIA 1
## 2001 K59 DIA 1
## 2002 K592 DIA 1
## 2003 K593 DIA 1
## 2004 K599 DIA 1
## 2005 K601 DIA 1
## 2006 K604 DIA 1
## 2007 K611 DIA 1
## 2008 K620 DIA 1
## 2009 K624 DIA 1
## 2010 K633 D500 1
## 2011 K640 DIA 1
## 2012 K641 DIA 1
## 2013 K66 E14 1
## 2014 K713 A099 1
## 2015 K714 DIA 1
## 2016 K716 DIA 1
## 2017 K730 DIA 1
## 2018 K738 DIA 1
## 2019 K752 A090 1
## 2020 K753 DIA 1
## 2021 K754 DIA 1
## 2022 K76 E14 1
## 2023 K761 DIA 1
## 2024 K770 D509 1
## 2025 K778 DIA 1
## 2026 K80X DIA 1
## 2027 K81X B24X 1
## 2028 K821 DIA 1
## 2029 K822 D619 1
## 2030 K824 DIA 1
## 2031 K832 DIA 1
## 2032 K86 DIA 1
## 2033 K90 E14 1
## 2034 K938 DIA 1
## 2035 L059 DIA 1
## 2036 L08 DIA 1
## 2037 L081 D508 1
## 2038 L108 DIA 1
## 2039 L121 D649 1
## 2040 L129 DIA 1
## 2041 L139 DIA 1
## 2042 L14X DIA 1
## 2043 L200 DIA 1
## 2044 L208 DIA 1
## 2045 L210 DIA 1
## 2046 L22X DIA 1
## 2047 L231 D509 1
## 2048 L238 DIA 1
## 2049 L249 DIA 1
## 2050 L26 A499 1
## 2051 L271 DIA 1
## 2052 L280 DIA 1
## 2053 L282 DIA 1
## 2054 L292 DIA 1
## 2055 L298 DIA 1
## 2056 L300 DIA 1
## 2057 L414 DIA 1
## 2058 L480 DIA 1
## 2059 L501 DIA 1
## 2060 L502 DIA 1
## 2061 L508 DIA 1
## 2062 L519 D649 1
## 2063 L538 D649 1
## 2064 L551 DIA 1
## 2065 L570 DIA 1
## 2066 L600 DIA 1
## 2067 L602 DIA 1
## 2068 L603 DIA 1
## 2069 L640 DIA 1
## 2070 L680 DIA 1
## 2071 L702 DIA 1
## 2072 L817 DIA 1
## 2073 L819 A099 1
## 2074 L82X DIA 1
## 2075 L84X DIA 1
## 2076 L853 DIA 1
## 2077 L871 DIA 1
## 2078 L903 DIA 1
## 2079 L905 DIA 1
## 2080 L929 DIA 1
## 2081 L931 DIA 1
## 2082 L932 DIA 1
## 2083 L940 DIA 1
## 2084 L980 B180 1
## 2085 L998 DIA 1
## 2086 M008 DIA 1
## 2087 M018 DIA 1
## 2088 M030 DIA 1
## 2089 M052 DIA 1
## 2090 M064 DIA 1
## 2091 M082 DIA 1
## 2092 M103 DIA 1
## 2093 M112 DIA 1
## 2094 M120 DIA 1
## 2095 M138 DIA 1
## 2096 M142 DIA 1
## 2097 M145 DIA 1
## 2098 M153 DIA 1
## 2099 M159 A090 1
## 2100 M161 DIA 1
## 2101 M172 DIA 1
## 2102 M175 DIA 1
## 2103 M198 DIA 1
## 2104 M204 DIA 1
## 2105 M215 DIA 1
## 2106 M224 DIA 1
## 2107 M231 DIA 1
## 2108 M232 DIA 1
## 2109 M243 DIA 1
## 2110 M245 DIA 1
## 2111 M250 DIA 1
## 2112 M259 DIA 1
## 2113 M316 DIA 1
## 2114 M317 DIA 1
## 2115 M350 DIA 1
## 2116 M361 DIA 1
## 2117 M413 DIA 1
## 2118 M418 DIA 1
## 2119 M421 A498 1
## 2120 M429 DIA 1
## 2121 M462 DIA 1
## 2122 M47 E14 1
## 2123 M470 DIA 1
## 2124 M471 DIA 1
## 2125 M488 DIA 1
## 2126 M502 DIA 1
## 2127 M510 DIA 1
## 2128 M518 DIA 1
## 2129 M530 DIA 1
## 2130 M540 D530 1
## 2131 M546 DIA 1
## 2132 M548 DIA 1
## 2133 M621 DIA 1
## 2134 M650 DIA 1
## 2135 M651 DIA 1
## 2136 M653 DIA 1
## 2137 M658 DIA 1
## 2138 M662 DIA 1
## 2139 M688 DIA 1
## 2140 M701 DIA 1
## 2141 M703 DIA 1
## 2142 M711 DIA 1
## 2143 M719 DIA 1
## 2144 M751 D693 1
## 2145 M752 DIA 1
## 2146 M754 DIA 1
## 2147 M758 DIA 1
## 2148 M760 D500 1
## 2149 M761 DIA 1
## 2150 M762 DIA 1
## 2151 M763 DIA 1
## 2152 M766 DIA 1
## 2153 M770 DIA 1
## 2154 M773 DIA 1
## 2155 M792 DIA 1
## 2156 M796 DIA 1
## 2157 M797 DIA 1
## 2158 M800 DIA 1
## 2159 M841 DIA 1
## 2160 M848 DIA 1
## 2161 M850 DIA 1
## 2162 M854 C050 1
## 2163 M862 A419 1
## 2164 M865 DIA 1
## 2165 M898 DIA 1
## 2166 M900 DIA 1
## 2167 M901 DIA 1
## 2168 M902 DIA 1
## 2169 M903 DIA 1
## 2170 M906 DIA 1
## 2171 M922 D500 1
## 2172 M928 DIA 1
## 2173 M948 DIA 1
## 2174 M952 DIA 1
## 2175 M960 DIA 1
## 2176 M966 DIA 1
## 2177 M990 DIA 1
## 2178 M995 DIA 1
## 2179 N002 DIA 1
## 2180 N021 DIA 1
## 2181 N025 DIA 1
## 2182 N028 DIA 1
## 2183 N042 DIA 1
## 2184 N058 DIA 1
## 2185 N063 A09X 1
## 2186 N070 DIA 1
## 2187 N088 DIA 1
## 2188 N10 DIA 1
## 2189 N12 DIA 1
## 2190 N134 DIA 1
## 2191 N135 DIA 1
## 2192 N140 DIA 1
## 2193 N150 DIA 1
## 2194 N164 DIA 1
## 2195 N171 DIA 1
## 2196 N21 E13 1
## 2197 N222 DIA 1
## 2198 N26X D649 1
## 2199 N298 DIA 1
## 2200 N302 DIA 1
## 2201 N303 DIA 1
## 2202 N304 D501 1
## 2203 N308 DIA 1
## 2204 N329 A049 1
## 2205 N341 DIA 1
## 2206 N342 DIA 1
## 2207 N358 DIA 1
## 2208 N360 DIA 1
## 2209 N362 DIA 1
## 2210 N369 A419 1
## 2211 N370 DIA 1
## 2212 N418 DIA 1
## 2213 N46 DIA 1
## 2214 N48 DIA 1
## 2215 N500 DIA 1
## 2216 N509 DIA 1
## 2217 N600 DIA 1
## 2218 N601 DIA 1
## 2219 N61 E14 1
## 2220 N641 A419 1
## 2221 N645 DIA 1
## 2222 N730 DIA 1
## 2223 N731 D589 1
## 2224 N732 D539 1
## 2225 N734 DIA 1
## 2226 N76 DIA 1
## 2227 N765 D049 1
## 2228 N808 DIA 1
## 2229 N828 DIA 1
## 2230 N831 DIA 1
## 2231 N8324 DIA 1
## 2232 N838 DIA 1
## 2233 N839 DIA 1
## 2234 N849 DIA 1
## 2235 N855 DIA 1
## 2236 N890 DIA 1
## 2237 N891 DIA 1
## 2238 N908 DIA 1
## 2239 N909 DIA 1
## 2240 N949 DIA 1
## 2241 N950 D649 1
## 2242 N951 E10X 1
## 2243 N970 DIA 1
## 2244 N992 DIA 1
## 2245 N998 DIA 1
## 2246 O000 DIA 1
## 2247 O019 DIA 1
## 2248 O030 DIA 1
## 2249 O033 DIA 1
## 2250 O039 DIA 1
## 2251 O054 DIA 1
## 2252 O088 DIA 1
## 2253 O219 DIA 1
## 2254 O220 DIA 1
## 2255 O231 DIA 1
## 2256 O239 DIA 1
## 2257 O243 DIA 1
## 2258 O244 DIA 1
## 2259 O25X DIA 1
## 2260 O300 DIA 1
## 2261 O323 DIA 1
## 2262 O324 DIA 1
## 2263 O326 DIA 1
## 2264 O330 DIA 1
## 2265 O334 DIA 1
## 2266 O338 DIA 1
## 2267 O339 DIA 1
## 2268 O365 DIA 1
## 2269 O368 DIA 1
## 2270 O420 DIA 1
## 2271 O421 DIA 1
## 2272 O439 DIA 1
## 2273 O471 DIA 1
## 2274 O610 DIA 1
## 2275 O624 DIA 1
## 2276 O645 DIA 1
## 2277 O648 DIA 1
## 2278 O662 DIA 1
## 2279 O679 DIA 1
## 2280 O700 DIA 1
## 2281 O722 DIA 1
## 2282 O759 DIA 1
## 2283 O8001 DIA 1
## 2284 O808 DIA 1
## 2285 O8284 DIA 1
## 2286 O838 DIA 1
## 2287 O85X DIA 1
## 2288 O862 DIA 1
## 2289 O863 DIA 1
## 2290 O868 DIA 1
## 2291 O900 DIA 1
## 2292 O994 DIA 1
## 2293 P369 DIA 1
## 2294 P95X DIA 1
## 2295 Q019 DIA 1
## 2296 Q046 DIA 1
## 2297 Q049 DIA 1
## 2298 Q219 DIA 1
## 2299 Q253 DIA 1
## 2300 Q259 DIA 1
## 2301 Q273 DIA 1
## 2302 Q279 DIA 1
## 2303 Q400 DIA 1
## 2304 Q603 DIA 1
## 2305 Q605 DIA 1
## 2306 Q612 C609 1
## 2307 Q620 DIA 1
## 2308 Q631 D410 1
## 2309 Q660 DIA 1
## 2310 Q720 C300 1
## 2311 Q738 DIA 1
## 2312 Q780 DIA 1
## 2313 Q785 DIA 1
## 2314 Q810 DIA 1
## 2315 Q828 DIA 1
## 2316 Q831 DIA 1
## 2317 Q850 DIA 1
## 2318 Q871 DIA 1
## 2319 Q874 DIA 1
## 2320 Q96 DIA 1
## 2321 R011 DIA 1
## 2322 R02 D509 1
## 2323 R031 DIA 1
## 2324 R068 DIA 1
## 2325 R071 DIA 1
## 2326 R090 DIA 1
## 2327 R12X DIA 1
## 2328 R198 DIA 1
## 2329 R222 DIA 1
## 2330 R224 DIA 1
## 2331 R270 DIA 1
## 2332 R278 D649 1
## 2333 R301 D509 1
## 2334 R390 DIA 1
## 2335 R398 DIA 1
## 2336 R400 DIA 1
## 2337 R470 DIA 1
## 2338 R478 DIA 1
## 2339 R51 DIA 1
## 2340 R54 A415 1
## 2341 R599 DIA 1
## 2342 R651 A419 1
## 2343 R652 DIA 1
## 2344 R680 A403 1
## 2345 R749 DIA 1
## 2346 R785 DIA 1
## 2347 R799 DIA 1
## 2348 R831 A152 1
## 2349 R900 DIA 1
## 2350 R98X DIA 1
## 2351 S007 DIA 1
## 2352 S014 DIA 1
## 2353 S015 DIA 1
## 2354 S017 DIA 1
## 2355 S018 DIA 1
## 2356 S025 DIA 1
## 2357 S026 DIA 1
## 2358 S031 DIA 1
## 2359 S045 DIA 1
## 2360 S055 B370 1
## 2361 S060 DIA 1
## 2362 S061 DIA 1
## 2363 S063 DIA 1
## 2364 S068 DIA 1
## 2365 S098 DIA 1
## 2366 S100 DIA 1
## 2367 S127 DIA 1
## 2368 S129 DIA 1
## 2369 S143 DIA 1
## 2370 S144 DIA 1
## 2371 S151 DIA 1
## 2372 S159 DIA 1
## 2373 S203 DIA 1
## 2374 S218 DIA 1
## 2375 S228 DIA 1
## 2376 S230 DIA 1
## 2377 S270 DIA 1
## 2378 S298 DIA 1
## 2379 S302 DIA 1
## 2380 S308 DIA 1
## 2381 S312 DIA 1
## 2382 S315 DIA 1
## 2383 S318 DIA 1
## 2384 S325 D648 1
## 2385 S327 DIA 1
## 2386 S340 DIA 1
## 2387 S341 DIA 1
## 2388 S344 DIA 1
## 2389 S359 D62X 1
## 2390 S361 D649 1
## 2391 S372 DIA 1
## 2392 S390 DIA 1
## 2393 S398 D62X 1
## 2394 S400 DIA 1
## 2395 S407 DIA 1
## 2396 S408 DIA 1
## 2397 S410 DIA 1
## 2398 S418 DIA 1
## 2399 S429 DIA 1
## 2400 S431 DIA 1
## 2401 S459 DIA 1
## 2402 S460 DIA 1
## 2403 S481 DIA 1
## 2404 S507 DIA 1
## 2405 S521 DIA 1
## 2406 S523 DIA 1
## 2407 S531 D649 1
## 2408 S550 DIA 1
## 2409 S589 DIA 1
## 2410 S607 DIA 1
## 2411 S623 DIA 1
## 2412 S635 DIA 1
## 2413 S644 DIA 1
## 2414 S668 DIA 1
## 2415 S678 DIA 1
## 2416 S684 DIA 1
## 2417 S689 DIA 1
## 2418 S708 DIA 1
## 2419 S717 DIA 1
## 2420 S748 DIA 1
## 2421 S799 DIA 1
## 2422 S801 DIA 1
## 2423 S809 DIA 1
## 2424 S832 DIA 1
## 2425 S849 DIA 1
## 2426 S860 DIA 1
## 2427 S899 DIA 1
## 2428 S900 DIA 1
## 2429 S902 DIA 1
## 2430 S907 DIA 1
## 2431 S922 DIA 1
## 2432 S923 DIA 1
## 2433 S927 DIA 1
## 2434 S934 DIA 1
## 2435 S936 A480 1
## 2436 S948 DIA 1
## 2437 S978 DIA 1
## 2438 S999 DIA 1
## 2439 T000 DIA 1
## 2440 T013 DIA 1
## 2441 T018 DIA 1
## 2442 T050 DIA 1
## 2443 T058 DIA 1
## 2444 T068 DIA 1
## 2445 T099 DIA 1
## 2446 T135 DIA 1
## 2447 T138 DIA 1
## 2448 T145 DIA 1
## 2449 T148 DIA 1
## 2450 T150 DIA 1
## 2451 T180 DIA 1
## 2452 T189 DIA 1
## 2453 T191 DIA 1
## 2454 T210 DIA 1
## 2455 T212 DIA 1
## 2456 T214 A419 1
## 2457 T230 DIA 1
## 2458 T231 DIA 1
## 2459 T291 DIA 1
## 2460 T293 DIA 1
## 2461 T300 DIA 1
## 2462 T304 DIA 1
## 2463 T315 DIA 1
## 2464 T365 DIA 1
## 2465 T368 DIA 1
## 2466 T441 DIA 1
## 2467 T443 DIA 1
## 2468 T479 DIA 1
## 2469 T528 DIA 1
## 2470 T541 DIA 1
## 2471 T542 DIA 1
## 2472 T58X DIA 1
## 2473 T601 DIA 1
## 2474 T604 DIA 1
## 2475 T635 DIA 1
## 2476 T670 DIA 1
## 2477 T674 DIA 1
## 2478 T698 DIA 1
## 2479 T741 DIA 1
## 2480 T751 DIA 1
## 2481 T78 DIA 1
## 2482 T783 DIA 1
## 2483 T789 DIA 1
## 2484 T794 DIA 1
## 2485 T801 DIA 1
## 2486 T802 D649 1
## 2487 T803 D689 1
## 2488 T808 DIA 1
## 2489 T815 DIA 1
## 2490 T818 DIA 1
## 2491 T819 DIA 1
## 2492 T833 DIA 1
## 2493 T835 D649 1
## 2494 T848 DIA 1
## 2495 T859 DIA 1
## 2496 T869 DIA 1
## 2497 T889 DIA 1
## 2498 T935 DIA 1
## 2499 T936 DIA 1
## 2500 U205 DIA 1
## 2501 V099 DIA 1
## 2502 V385 DIA 1
## 2503 W018 DIA 1
## 2504 W060 DIA 1
## 2505 W079 DIA 1
## 2506 W089 DIA 1
## 2507 W159 DIA 1
## 2508 W170 DIA 1
## 2509 W180 DIA 1
## 2510 W184 DIA 1
## 2511 W204 DIA 1
## 2512 W229 DIA 1
## 2513 X100 DIA 1
## 2514 X109 DIA 1
## 2515 X689 A059 1
## 2516 X690 DIA 1
## 2517 X699 DIA 1
## 2518 X954 DIA 1
## 2519 Y279 DIA 1
## 2520 Y411 A153 1
## 2521 Y423 DIA 1
## 2522 Y822 DIA 1
## 2523 Y834 DIA 1
## 2524 Y846 DIA 1
## 2525 Y913 DIA 1
## 2526 Z226 B86X 1
## 2527 Z3593 DIA 1
## 2528 Z391 DIA 1
## 2529 Z450 DIA 1
## 2530 Z730 DIA 1
## 2531 Z749 DIA 1
## 2532 Z930 DIA 1
## 2533 Z932 DIA 1
## 2534 Z958 DIA 1

### Alive patients, before lockdown

## Diag1 Diag2 Frequency
## 1 I10X DIA 9734
## 2 N390 DIA 8736
## 3 N189 DIA 2640
## 4 J189 DIA 1458
## 5 D649 DIA 1374
## 6 L031 DIA 1105
## 7 N10X DIA 767
## 8 I500 DIA 749
## 9 N185 DIA 734
## 10 A419 DIA 720
## 11 E669 DIA 693
## 12 E162 DIA 643
## 13 I64X DIA 569
## 14 D509 DIA 541
## 15 N40X DIA 472
## 16 E039 DIA 456
## 17 K922 DIA 441
## 18 J960 DIA 437
## 19 I509 DIA 425
## 20 K746 DIA 395
## 21 J159 DIA 369
## 22 I678 DIA 367
## 23 L039 DIA 364
## 24 I219 DIA 360
## 25 R739 DIA 359
## 26 I639 DIA 354
## 27 A099 DIA 347
## 28 A090 DIA 345
## 29 N110 DIA 337
## 30 R104 DIA 297
## 31 N12X DIA 292
## 32 A150 DIA 286
## 33 E86X DIA 282
## 34 A560 DIA 260
## 35 S913 DIA 253
## 36 N179 DIA 240
## 37 J969 DIA 239
## 38 N039 DIA 238
## 39 K811 DIA 232
## 40 I679 DIA 225
## 41 K802 DIA 223
## 42 J90X DIA 220
## 43 N200 DIA 217
## 44 J849 DIA 213
## 45 K859 DIA 211
## 46 J841 DIA 197
## 47 I739 DIA 179
## 48 K703 DIA 177
## 49 N19X DIA 172
## 50 I119 DIA 171
## 51 L97X DIA 167
## 52 L030 DIA 164
## 53 J459 DIA 161
## 54 G409 DIA 159
## 55 D259 DIA 156
## 56 I489 DIA 153
## 57 K810 DIA 153
## 58 I159 DIA 143
## 59 D539 DIA 140
## 60 L024 DIA 137
## 61 R509 DIA 136
## 62 D500 DIA 135
## 63 I694 DIA 134
## 64 K297 DIA 131
## 65 G934 DIA 128
## 66 I619 DIA 127
## 67 L038 DIA 122
## 68 N819 DIA 122
## 69 K801 DIA 120
## 70 R568 DIA 120
## 71 J209 DIA 118
## 72 M869 DIA 116
## 73 E660 DIA 112
## 74 K805 DIA 112
## 75 L023 DIA 112
## 76 M069 DIA 109
## 77 N180 DIA 107
## 78 T136 DIA 106
## 79 R11X DIA 105
## 80 I872 DIA 104
## 81 J180 DIA 103
## 82 R02X DIA 100
## 83 E160 DIA 98
## 84 I480 DIA 98
## 85 J961 DIA 95
## 86 L029 DIA 94
## 87 L032 DIA 94
## 88 A162 DIA 92
## 89 E785 DIA 91
## 90 G459 DIA 91
## 91 E668 DIA 90
## 92 I259 DIA 90
## 93 K800 DIA 90
## 94 L899 DIA 90
## 95 I110 DIA 89
## 96 I200 DIA 88
## 97 N184 DIA 88
## 98 S981 DIA 88
## 99 N181 DIA 87
## 100 S984 DIA 86
## 101 K850 DIA 84
## 102 H360 DIA 83
## 103 K295 DIA 83
## 104 N151 DIA 82
## 105 A169 DIA 81
## 106 K429 DIA 81
## 107 N399 DIA 81
## 108 E780 DIA 80
## 109 K610 DIA 79
## 110 K85X DIA 78
## 111 N183 DIA 78
## 112 E782 DIA 76
## 113 E872 DIA 76
## 114 N111 DIA 76
## 115 E46X DIA 75
## 116 S889 DIA 75
## 117 J690 DIA 74
## 118 K291 DIA 74
## 119 I48X DIA 71
## 120 I633 DIA 71
## 121 J188 DIA 71
## 122 J120 DIA 70
## 123 K729 DIA 69
## 124 N813 DIA 68
## 125 I120 DIA 67
## 126 I802 DIA 67
## 127 K409 DIA 66
## 128 N832 DIA 66
## 129 R042 DIA 66
## 130 R18X DIA 66
## 131 A09X DIA 65
## 132 G20X DIA 65
## 133 L089 DIA 65
## 134 D648 DIA 64
## 135 K439 DIA 64
## 136 I610 DIA 63
## 137 J13X DIA 63
## 138 K819 DIA 63
## 139 K590 DIA 62
## 140 I830 DIA 61
## 141 N049 DIA 60
## 142 L022 DIA 59
## 143 R51X DIA 59
## 144 J80X DIA 58
## 145 N119 DIA 58
## 146 L984 DIA 57
## 147 F200 DIA 56
## 148 I209 DIA 56
## 149 J029 DIA 55
## 150 T814 DIA 55
## 151 N319 DIA 54
## 152 I499 DIA 53
## 153 K566 DIA 53
## 154 N760 DIA 53
## 155 D638 DIA 52
## 156 F329 DIA 52
## 157 A418 DIA 51
## 158 E871 DIA 51
## 159 K808 DIA 51
## 160 N289 DIA 51
## 161 E059 DIA 50
## 162 F209 DIA 50
## 163 F412 DIA 50
## 164 I693 DIA 50
## 165 I829 DIA 50
## 166 L890 DIA 50
## 167 K30X DIA 49
## 168 I150 DIA 48
## 169 I442 DIA 46
## 170 J852 DIA 46
## 171 L021 DIA 46
## 172 A159 DIA 45
## 173 I859 DIA 45
## 174 J129 DIA 45
## 175 J158 DIA 45
## 176 J22X DIA 45
## 177 K750 DIA 45
## 178 S069 DIA 45
## 179 S982 DIA 45
## 180 A010 DIA 44
## 181 A409 DIA 44
## 182 F03X DIA 44
## 183 I792 DIA 44
## 184 L020 DIA 44
## 185 R100 DIA 44
## 186 G590 DIA 43
## 187 I609 DIA 42
## 188 N498 DIA 42
## 189 I698 DIA 41
## 190 J40X DIA 41
## 191 N139 DIA 41
## 192 O829 DIA 41
## 193 S721 DIA 41
## 194 S819 DIA 41
## 195 G632 DIA 40
## 196 I634 DIA 40
## 197 M725 DIA 40
## 198 I832 DIA 39
## 199 R17X DIA 39
## 200 R609 DIA 39
## 201 S822 DIA 39
## 202 A153 DIA 38
## 203 C539 DIA 38
## 204 F419 DIA 38
## 205 L409 DIA 38
## 206 N133 DIA 38
## 207 D693 DIA 37
## 208 H269 DIA 37
## 209 J181 DIA 37
## 210 J984 DIA 37
## 211 K830 DIA 37
## 212 K047 DIA 36
## 213 K769 DIA 36
## 214 I255 DIA 35
## 215 K259 DIA 35
## 216 N209 DIA 35
## 217 N61X DIA 35
## 218 T009 DIA 35
## 219 C169 DIA 34
## 220 E038 DIA 34
## 221 E876 DIA 34
## 222 G610 DIA 34
## 223 I630 DIA 34
## 224 N814 DIA 34
## 225 S880 DIA 34
## 226 B86X DIA 33
## 227 C61X DIA 33
## 228 E161 DIA 33
## 229 E880 DIA 33
## 230 J869 DIA 33
## 231 M109 DIA 33
## 232 R101 DIA 33
## 233 E43X DIA 32
## 234 E870 DIA 32
## 235 R31X DIA 32
## 236 R33X DIA 32
## 237 S729 DIA 32
## 238 T874 DIA 32
## 239 L89X DIA 31
## 240 E878 DIA 30
## 241 L033 DIA 30
## 242 O821 DIA 30
## 243 J152 DIA 29
## 244 K37X DIA 29
## 245 E440 DIA 28
## 246 F102 DIA 28
## 247 G629 DIA 28
## 248 I519 DIA 28
## 249 J100 DIA 28
## 250 M139 DIA 28
## 251 N182 DIA 28
## 252 N771 DIA 28
## 253 C189 DIA 27
## 254 G309 DIA 27
## 255 G819 DIA 27
## 256 G909 DIA 27
## 257 I340 DIA 27
## 258 I743 DIA 27
## 259 I850 DIA 27
## 260 J448 DIA 27
## 261 J46X DIA 27
## 262 K290 DIA 27
## 263 K851 DIA 27
## 264 N083 DIA 27
## 265 O990 DIA 27
## 266 T887 DIA 27
## 267 A499 DIA 26
## 268 E249 DIA 26
## 269 H819 DIA 26
## 270 K122 DIA 26
## 271 N812 DIA 26
## 272 A90X DIA 25
## 273 G510 DIA 25
## 274 I151 DIA 25
## 275 I620 DIA 25
## 276 J150 DIA 25
## 277 D696 DIA 24
## 278 I490 DIA 24
## 279 I828 DIA 24
## 280 M726 DIA 24
## 281 N23X DIA 24
## 282 N459 DIA 24
## 283 N492 DIA 24
## 284 S065 DIA 24
## 285 S911 DIA 24
## 286 A156 DIA 23
## 287 H050 DIA 23
## 288 I771 DIA 23
## 289 K250 DIA 23
## 290 K296 DIA 23
## 291 K920 DIA 23
## 292 M009 DIA 23
## 293 M179 DIA 23
## 294 O234 DIA 23
## 295 R571 DIA 23
## 296 T141 DIA 23
## 297 B829 DIA 22
## 298 G442 DIA 22
## 299 I158 DIA 22
## 300 I249 DIA 22
## 301 I635 DIA 22
## 302 I959 DIA 22
## 303 J156 DIA 22
## 304 J980 DIA 22
## 305 K469 DIA 22
## 306 K591 DIA 22
## 307 N312 DIA 22
## 308 R572 DIA 22
## 309 S681 DIA 22
## 310 A971 DIA 21
## 311 E889 DIA 21
## 312 I210 DIA 21
## 313 I629 DIA 21
## 314 J068 DIA 21
## 315 J440 DIA 21
## 316 K279 DIA 21
## 317 O034 DIA 21
## 318 O342 DIA 21
## 319 R32X DIA 21
## 320 S828 DIA 21
## 321 S980 DIA 21
## 322 C56X DIA 20
## 323 E441 DIA 20
## 324 H813 DIA 20
## 325 I702 DIA 20
## 326 K603 DIA 20
## 327 K745 DIA 20
## 328 N300 DIA 20
## 329 R418 DIA 20
## 330 F321 DIA 19
## 331 H280 DIA 19
## 332 H409 DIA 19
## 333 H540 DIA 19
## 334 I612 DIA 19
## 335 I809 DIA 19
## 336 I839 DIA 19
## 337 J698 DIA 19
## 338 K760 DIA 19
## 339 M059 DIA 19
## 340 N310 DIA 19
## 341 N938 DIA 19
## 342 T793 DIA 19
## 343 B370 DIA 18
## 344 B379 DIA 18
## 345 C220 DIA 18
## 346 C229 DIA 18
## 347 D410 DIA 18
## 348 I471 DIA 18
## 349 K564 DIA 18
## 350 L400 DIA 18
## 351 N170 DIA 18
## 352 O820 DIA 18
## 353 S789 DIA 18
## 354 S818 DIA 18
## 355 T252 DIA 18
## 356 A400 DIA 17
## 357 A410 DIA 17
## 358 D531 DIA 17
## 359 G049 DIA 17
## 360 H669 DIA 17
## 361 I251 DIA 17
## 362 K269 DIA 17
## 363 K359 DIA 17
## 364 K650 DIA 17
## 365 L509 DIA 17
## 366 L80X DIA 17
## 367 M544 DIA 17
## 368 N040 DIA 17
## 369 O809 DIA 17
## 370 S912 DIA 17
## 371 C259 DIA 16
## 372 G255 DIA 16
## 373 G408 DIA 16
## 374 G969 DIA 16
## 375 I443 DIA 16
## 376 J219 DIA 16
## 377 J42X DIA 16
## 378 J851 DIA 16
## 379 N172 DIA 16
## 380 N220 DIA 16
## 381 O064 DIA 16
## 382 R001 DIA 16
## 383 T131 DIA 16
## 384 T147 DIA 16
## 385 T633 DIA 16
## 386 T813 DIA 16
## 387 F019 DIA 15
## 388 G990 DIA 15
## 389 H46X DIA 15
## 390 H811 DIA 15
## 391 H82X DIA 15
## 392 I618 DIA 15
## 393 J069 DIA 15
## 394 J91X DIA 15
## 395 K20X DIA 15
## 396 K420 DIA 15
## 397 L088 DIA 15
## 398 L88X DIA 15
## 399 N178 DIA 15
## 400 N188 DIA 15
## 401 N309 DIA 15
## 402 R601 DIA 15
## 403 R770 DIA 15
## 404 S723 DIA 15
## 405 S917 DIA 15
## 406 Y835 DIA 15
## 407 A170 DIA 14
## 408 B351 DIA 14
## 409 B378 DIA 14
## 410 C910 DIA 14
## 411 E169 DIA 14
## 412 E835 DIA 14
## 413 G919 DIA 14
## 414 I129 DIA 14
## 415 I152 DIA 14
## 416 I420 DIA 14
## 417 I632 DIA 14
## 418 K351 DIA 14
## 419 N130 DIA 14
## 420 N159 DIA 14
## 421 N499 DIA 14
## 422 R072 DIA 14
## 423 B009 DIA 13
## 424 B354 DIA 13
## 425 D376 DIA 13
## 426 E220 DIA 13
## 427 E271 DIA 13
## 428 F011 DIA 13
## 429 H259 DIA 13
## 430 I250 DIA 13
## 431 I350 DIA 13
## 432 I611 DIA 13
## 433 J450 DIA 13
## 434 J848 DIA 13
## 435 K318 DIA 13
## 436 K402 DIA 13
## 437 K660 DIA 13
## 438 L309 DIA 13
## 439 N219 DIA 13
## 440 N450 DIA 13
## 441 N47X DIA 13
## 442 O141 DIA 13
## 443 R42X DIA 13
## 444 R55X DIA 13
## 445 S619 DIA 13
## 446 S820 DIA 13
## 447 B465 DIA 12
## 448 D391 DIA 12
## 449 E875 DIA 12
## 450 G400 DIA 12
## 451 H919 DIA 12
## 452 I269 DIA 12
## 453 I516 DIA 12
## 454 I613 DIA 12
## 455 I674 DIA 12
## 456 I749 DIA 12
## 457 J039 DIA 12
## 458 J123 DIA 12
## 459 J151 DIA 12
## 460 J631 DIA 12
## 461 K046 DIA 12
## 462 K352 DIA 12
## 463 K353 DIA 12
## 464 K632 DIA 12
## 465 K659 DIA 12
## 466 K719 DIA 12
## 467 K759 DIA 12
## 468 L080 DIA 12
## 469 M866 DIA 12
## 470 N281 DIA 12
## 471 N328 DIA 12
## 472 N818 DIA 12
## 473 N872 DIA 12
## 474 O200 DIA 12
## 475 O364 DIA 12
## 476 S983 DIA 12
## 477 A480 DIA 11
## 478 G401 DIA 11
## 479 G589 DIA 11
## 480 I252 DIA 11
## 481 I258 DIA 11
## 482 I803 DIA 11
## 483 J168 DIA 11
## 484 J850 DIA 11
## 485 J948 DIA 11
## 486 K260 DIA 11
## 487 K803 DIA 11
## 488 K839 DIA 11
## 489 K929 DIA 11
## 490 L892 DIA 11
## 491 L893 DIA 11
## 492 M321 DIA 11
## 493 M600 DIA 11
## 494 N840 DIA 11
## 495 R53X DIA 11
## 496 S610 DIA 11
## 497 S682 DIA 11
## 498 T054 DIA 11
## 499 T302 DIA 11
## 500 T888 DIA 11
## 501 43552 DIA 10
## 502 C221 DIA 10
## 503 E02X DIA 10
## 504 E041 DIA 10
## 505 E050 DIA 10
## 506 E240 DIA 10
## 507 G042 DIA 10
## 508 I130 DIA 10
## 509 I48 DIA 10
## 510 I481 DIA 10
## 511 J157 DIA 10
## 512 J36X DIA 10
## 513 J410 DIA 10
## 514 J939 DIA 10
## 515 K294 DIA 10
## 516 K721 DIA 10
## 517 K740 DIA 10
## 518 K804 DIA 10
## 519 M100 DIA 10
## 520 M431 DIA 10
## 521 M868 DIA 10
## 522 N202 DIA 10
## 523 N394 DIA 10
## 524 N709 DIA 10
## 525 N830 DIA 10
## 526 O149 DIA 10
## 527 O800 DIA 10
## 528 S223 DIA 10
## 529 S422 DIA 10
## 530 S724 DIA 10
## 531 T600 DIA 10
## 532 43553 DIA 9
## 533 B353 DIA 9
## 534 D069 DIA 9
## 535 E230 DIA 9
## 536 F009 DIA 9
## 537 F100 DIA 9
## 538 F410 DIA 9
## 539 F449 DIA 9
## 540 G619 DIA 9
## 541 H650 DIA 9
## 542 I059 DIA 9
## 543 I213 DIA 9
## 544 I270 DIA 9
## 545 I479 DIA 9
## 546 I776 DIA 9
## 547 I800 DIA 9
## 548 I99X DIA 9
## 549 J860 DIA 9
## 550 J949 DIA 9
## 551 K219 DIA 9
## 552 K221 DIA 9
## 553 K270 DIA 9
## 554 K350 DIA 9
## 555 K579 DIA 9
## 556 K612 DIA 9
## 557 K625 DIA 9
## 558 K768 DIA 9
## 559 L891 DIA 9
## 560 M793 DIA 9
## 561 M819 DIA 9
## 562 M861 DIA 9
## 563 N009 DIA 9
## 564 N398 DIA 9
## 565 R074 DIA 9
## 566 R54X DIA 9
## 567 S062 DIA 9
## 568 S411 DIA 9
## 569 S525 DIA 9
## 570 S920 DIA 9
## 571 T243 DIA 9
## 572 Z488 DIA 9
## 573 Z519 DIA 9
## 574 A279 DIA 8
## 575 F320 DIA 8
## 576 F328 DIA 8
## 577 G060 DIA 8
## 578 G402 DIA 8
## 579 G403 DIA 8
## 580 G410 DIA 8
## 581 G569 DIA 8
## 582 H660 DIA 8
## 583 I211 DIA 8
## 584 I459 DIA 8
## 585 I498 DIA 8
## 586 I600 DIA 8
## 587 I631 DIA 8
## 588 I64 DIA 8
## 589 I770 DIA 8
## 590 I775 DIA 8
## 591 I801 DIA 8
## 592 J128 DIA 8
## 593 J14X DIA 8
## 594 K210 DIA 8
## 595 K319 DIA 8
## 596 K648 DIA 8
## 597 K658 DIA 8
## 598 K861 DIA 8
## 599 L500 DIA 8
## 600 M169 DIA 8
## 601 M255 DIA 8
## 602 M331 DIA 8
## 603 M490 DIA 8
## 604 M512 DIA 8
## 605 M541 DIA 8
## 606 M549 DIA 8
## 607 M729 DIA 8
## 608 N000 DIA 8
## 609 N210 DIA 8
## 610 N481 DIA 8
## 611 N870 DIA 8
## 612 N952 DIA 8
## 613 O210 DIA 8
## 614 O249 DIA 8
## 615 R000 DIA 8
## 616 S328 DIA 8
## 617 S810 DIA 8
## 618 S817 DIA 8
## 619 S910 DIA 8
## 620 T140 DIA 8
## 621 T242 DIA 8
## 622 T857 DIA 8
## 623 43564 DIA 7
## 624 43566 DIA 7
## 625 A064 DIA 7
## 626 D589 DIA 7
## 627 D590 DIA 7
## 628 E242 DIA 7
## 629 E874 DIA 7
## 630 F432 DIA 7
## 631 G360 DIA 7
## 632 G939 DIA 7
## 633 H350 DIA 7
## 634 I441 DIA 7
## 635 J040 DIA 7
## 636 J441 DIA 7
## 637 J942 DIA 7
## 638 J988 DIA 7
## 639 K299 DIA 7
## 640 K449 DIA 7
## 641 K567 DIA 7
## 642 K630 DIA 7
## 643 K766 DIA 7
## 644 L100 DIA 7
## 645 L719 DIA 7
## 646 L989 DIA 7
## 647 M053 DIA 7
## 648 M150 DIA 7
## 649 M479 DIA 7
## 650 M609 DIA 7
## 651 N029 DIA 7
## 652 N059 DIA 7
## 653 N158 DIA 7
## 654 N288 DIA 7
## 655 N320 DIA 7
## 656 N411 DIA 7
## 657 N751 DIA 7
## 658 N809 DIA 7
## 659 N810 DIA 7
## 660 N879 DIA 7
## 661 N911 DIA 7
## 662 O689 DIA 7
## 663 Q667 DIA 7
## 664 R410 DIA 7
## 665 R529 DIA 7
## 666 R560 DIA 7
## 667 R58X DIA 7
## 668 R64X DIA 7
## 669 S202 DIA 7
## 670 T253 DIA 7
## 671 T827 DIA 7
## 672 W199 DIA 7
## 673 43549 DIA 6
## 674 43556 DIA 6
## 675 43565 DIA 6
## 676 B022 DIA 6
## 677 C329 DIA 6
## 678 D414 DIA 6
## 679 D609 DIA 6
## 680 E049 DIA 6
## 681 E538 DIA 6
## 682 E873 DIA 6
## 683 F050 DIA 6
## 684 F059 DIA 6
## 685 F064 DIA 6
## 686 F130 DIA 6
## 687 F323 DIA 6
## 688 G009 DIA 6
## 689 G039 DIA 6
## 690 G219 DIA 6
## 691 G373 DIA 6
## 692 G439 DIA 6
## 693 G628 DIA 6
## 694 G92X DIA 6
## 695 G936 DIA 6
## 696 G959 DIA 6
## 697 H601 DIA 6
## 698 H609 DIA 6
## 699 I051 DIA 6
## 700 I060 DIA 6
## 701 I229 DIA 6
## 702 I429 DIA 6
## 703 I440 DIA 6
## 704 I447 DIA 6
## 705 I460 DIA 6
## 706 I469 DIA 6
## 707 I671 DIA 6
## 708 I688 DIA 6
## 709 I729 DIA 6
## 710 I831 DIA 6
## 711 I891 DIA 6
## 712 J019 DIA 6
## 713 J930 DIA 6
## 714 J938 DIA 6
## 715 K102 DIA 6
## 716 K257 DIA 6
## 717 K565 DIA 6
## 718 K605 DIA 6
## 719 K720 DIA 6
## 720 K828 DIA 6
## 721 K829 DIA 6
## 722 K928 DIA 6
## 723 L959 DIA 6
## 724 M131 DIA 6
## 725 M809 DIA 6
## 726 N048 DIA 6
## 727 N132 DIA 6
## 728 N201 DIA 6
## 729 N359 DIA 6
## 730 N433 DIA 6
## 731 O020 DIA 6
## 732 O021 DIA 6
## 733 O140 DIA 6
## 734 O230 DIA 6
## 735 R392 DIA 6
## 736 R600 DIA 6
## 737 S009 DIA 6
## 738 S010 DIA 6
## 739 S099 DIA 6
## 740 S701 DIA 6
## 741 S821 DIA 6
## 742 S829 DIA 6
## 743 T630 DIA 6
## 744 T824 DIA 6
## 745 43558 DIA 5
## 746 43571 DIA 5
## 747 B461 DIA 5
## 748 E222 DIA 5
## 749 E784 DIA 5
## 750 F058 DIA 5
## 751 F067 DIA 5
## 752 F09X DIA 5
## 753 F101 DIA 5
## 754 F103 DIA 5
## 755 F109 DIA 5
## 756 F339 DIA 5
## 757 F341 DIA 5
## 758 F448 DIA 5
## 759 G041 DIA 5
## 760 G700 DIA 5
## 761 G938 DIA 5
## 762 H059 DIA 5
## 763 H109 DIA 5
## 764 H659 DIA 5
## 765 H663 DIA 5
## 766 H810 DIA 5
## 767 H903 DIA 5
## 768 I050 DIA 5
## 769 I131 DIA 5
## 770 I517 DIA 5
## 771 I601 DIA 5
## 772 I614 DIA 5
## 773 I709 DIA 5
## 774 I738 DIA 5
## 775 I744 DIA 5
## 776 I864 DIA 5
## 777 J342 DIA 5
## 778 J680 DIA 5
## 779 K253 DIA 5
## 780 K293 DIA 5
## 781 K298 DIA 5
## 782 K421 DIA 5
## 783 K649 DIA 5
## 784 K717 DIA 5
## 785 K85 DIA 5
## 786 K863 DIA 5
## 787 L259 DIA 5
## 788 L304 DIA 5
## 789 L539 DIA 5
## 790 M300 DIA 5
## 791 M430 DIA 5
## 792 M480 DIA 5
## 793 M705 DIA 5
## 794 M842 DIA 5
## 795 M879 DIA 5
## 796 N118 DIA 5
## 797 N322 DIA 5
## 798 N340 DIA 5
## 799 N391 DIA 5
## 800 N736 DIA 5
## 801 N738 DIA 5
## 802 N800 DIA 5
## 803 N816 DIA 5
## 804 N820 DIA 5
## 805 N971 DIA 5
## 806 O069 DIA 5
## 807 O600 DIA 5
## 808 R160 DIA 5
## 809 R402 DIA 5
## 810 R520 DIA 5
## 811 S271 DIA 5
## 812 S310 DIA 5
## 813 S311 DIA 5
## 814 S399 DIA 5
## 815 S611 DIA 5
## 816 S700 DIA 5
## 817 S718 DIA 5
## 818 S722 DIA 5
## 819 S823 DIA 5
## 820 T059 DIA 5
## 821 T07X DIA 5
## 822 T10X DIA 5
## 823 T634 DIA 5
## 824 T659 DIA 5
## 825 T702 DIA 5
## 826 43540 DIA 4
## 827 43547 DIA 4
## 828 43554 DIA 4
## 829 43559 DIA 4
## 830 43577 DIA 4
## 831 43580 DIA 4
## 832 A521 DIA 4
## 833 E031 DIA 4
## 834 E272 DIA 4
## 835 E559 DIA 4
## 836 E700 DIA 4
## 837 E739 DIA 4
## 838 E755 DIA 4
## 839 E756 DIA 4
## 840 E831 DIA 4
## 841 F319 DIA 4
## 842 F411 DIA 4
## 843 F609 DIA 4
## 844 G048 DIA 4
## 845 G050 DIA 4
## 846 G35X DIA 4
## 847 G464 DIA 4
## 848 G500 DIA 4
## 849 G530 DIA 4
## 850 G579 DIA 4
## 851 G618 DIA 4
## 852 G822 DIA 4
## 853 G931 DIA 4
## 854 G932 DIA 4
## 855 G98X DIA 4
## 856 H024 DIA 4
## 857 H043 DIA 4
## 858 H268 DIA 4
## 859 H368 DIA 4
## 860 H490 DIA 4
## 861 H588 DIA 4
## 862 H654 DIA 4
## 863 H664 DIA 4
## 864 H701 DIA 4
## 865 H900 DIA 4
## 866 I071 DIA 4
## 867 I139 DIA 4
## 868 I451 DIA 4
## 869 I482 DIA 4
## 870 I501 DIA 4
## 871 I660 DIA 4
## 872 I691 DIA 4
## 873 I700 DIA 4
## 874 I778 DIA 4
## 875 I779 DIA 4
## 876 I822 DIA 4
## 877 J101 DIA 4
## 878 J139 DIA 4
## 879 J390 DIA 4
## 880 J61X DIA 4
## 881 J853 DIA 4
## 882 K113 DIA 4
## 883 k259 DIA 4
## 884 K273 DIA 4
## 885 K36X DIA 4
## 886 K602 DIA 4
## 887 K623 DIA 4
## 888 K661 DIA 4
## 889 K700 DIA 4
## 890 K739 DIA 4
## 891 K743 DIA 4
## 892 K818 DIA 4
## 893 K858 DIA 4
## 894 K860 DIA 4
## 895 K868 DIA 4
## 896 L102 DIA 4
## 897 L219 DIA 4
## 898 L281 DIA 4
## 899 L401 DIA 4
## 900 L405 DIA 4
## 901 L408 DIA 4
## 902 L729 DIA 4
## 903 M029 DIA 4
## 904 M130 DIA 4
## 905 M146 DIA 4
## 906 M542 DIA 4
## 907 M779 DIA 4
## 908 M860 DIA 4
## 909 M932 DIA 4
## 910 N050 DIA 4
## 911 N131 DIA 4
## 912 N211 DIA 4
## 913 N311 DIA 4
## 914 N72X DIA 4
## 915 N871 DIA 4
## 916 O429 DIA 4
## 917 O479 DIA 4
## 918 O758 DIA 4
## 919 O828 DIA 4
## 920 Q610 DIA 4
## 921 R190 DIA 4
## 922 R221 DIA 4
## 923 R570 DIA 4
## 924 S008 DIA 4
## 925 S019 DIA 4
## 926 S024 DIA 4
## 927 S320 DIA 4
## 928 S324 DIA 4
## 929 S424 DIA 4
## 930 S520 DIA 4
## 931 S626 DIA 4
## 932 S628 DIA 4
## 933 S711 DIA 4
## 934 S826 DIA 4
## 935 S925 DIA 4
## 936 S929 DIA 4
## 937 T019 DIA 4
## 938 T149 DIA 4
## 939 T424 DIA 4
## 940 T519 DIA 4
## 941 T856 DIA 4
## 942 T905 DIA 4
## 943 W019 DIA 4
## 944 43519 DIA 3
## 945 43550 DIA 3
## 946 43560 DIA 3
## 947 43561 DIA 3
## 948 43563 DIA 3
## 949 43570 DIA 3
## 950 43572 DIA 3
## 951 A083 DIA 3
## 952 C166 DIA 3
## 953 C959 DIA 3
## 954 D151 DIA 3
## 955 D591 DIA 3
## 956 E042 DIA 3
## 957 E052 DIA 3
## 958 E069 DIA 3
## 959 E15X DIA 3
## 960 E209 DIA 3
## 961 E210 DIA 3
## 962 E229 DIA 3
## 963 E232 DIA 3
## 964 E236 DIA 3
## 965 E270 DIA 3
## 966 E66 DIA 3
## 967 E788 DIA 3
## 968 F018 DIA 3
## 969 F062 DIA 3
## 970 F069 DIA 3
## 971 F142 DIA 3
## 972 F172 DIA 3
## 973 F192 DIA 3
## 974 F238 DIA 3
## 975 F29X DIA 3
## 976 F445 DIA 3
## 977 F450 DIA 3
## 978 F799 DIA 3
## 979 G01X DIA 3
## 980 G09X DIA 3
## 981 G311 DIA 3
## 982 G430 DIA 3
## 983 G448 DIA 3
## 984 G450 DIA 3
## 985 G458 DIA 3
## 986 G531 DIA 3
## 987 G578 DIA 3
## 988 G588 DIA 3
## 989 G609 DIA 3
## 990 G729 DIA 3
## 991 G825 DIA 3
## 992 G952 DIA 3
## 993 H010 DIA 3
## 994 H103 DIA 3
## 995 H110 DIA 3
## 996 H352 DIA 3
## 997 H358 DIA 3
## 998 H400 DIA 3
## 999 H440 DIA 3
## 1000 H527 DIA 3
## 1001 H544 DIA 3
## 1002 H549 DIA 3
## 1003 H602 DIA 3
## 1004 H651 DIA 3
## 1005 H653 DIA 3
## 1006 I069 DIA 3
## 1007 I089 DIA 3
## 1008 I132 DIA 3
## 1009 I212 DIA 3
## 1010 I241 DIA 3
## 1011 I260 DIA 3
## 1012 I278 DIA 3
## 1013 I309 DIA 3
## 1014 I330 DIA 3
## 1015 I358 DIA 3
## 1016 I378 DIA 3
## 1017 I38X DIA 3
## 1018 I390 DIA 3
## 1019 I421 DIA 3
## 1020 I446 DIA 3
## 1021 I495 DIA 3
## 1022 I510 DIA 3
## 1023 I528 DIA 3
## 1024 I602 DIA 3
## 1025 I728 DIA 3
## 1026 I742 DIA 3
## 1027 I821 DIA 3
## 1028 I842 DIA 3
## 1029 I890 DIA 3
## 1030 I899 DIA 3
## 1031 I982 DIA 3
## 1032 J014 DIA 3
## 1033 J155 DIA 3
## 1034 J170 DIA 3
## 1035 J200 DIA 3
## 1036 J340 DIA 3
## 1037 J380 DIA 3
## 1038 J386 DIA 3
## 1039 J393 DIA 3
## 1040 J451 DIA 3
## 1041 J64X DIA 3
## 1042 J840 DIA 3
## 1043 J941 DIA 3
## 1044 J998 DIA 3
## 1045 K029 DIA 3
## 1046 K041 DIA 3
## 1047 K044 DIA 3
## 1048 K052 DIA 3
## 1049 K228 DIA 3
## 1050 K316 DIA 3
## 1051 K388 DIA 3
## 1052 K458 DIA 3
## 1053 K461 DIA 3
## 1054 K519 DIA 3
## 1055 K570 DIA 3
## 1056 K626 DIA 3
## 1057 K628 DIA 3
## 1058 K639 DIA 3
## 1059 K702 DIA 3
## 1060 K704 DIA 3
## 1061 K710 DIA 3
## 1062 K712 DIA 3
## 1063 K744 DIA 3
## 1064 K758 DIA 3
## 1065 K820 DIA 3
## 1066 K823 DIA 3
## 1067 K831 DIA 3
## 1068 K833 DIA 3
## 1069 K838 DIA 3
## 1070 K862 DIA 3
## 1071 K869 DIA 3
## 1072 K904 DIA 3
## 1073 K914 DIA 3
## 1074 K921 DIA 3
## 1075 L00X DIA 3
## 1076 L010 DIA 3
## 1077 L043 DIA 3
## 1078 L239 DIA 3
## 1079 L402 DIA 3
## 1080 L930 DIA 3
## 1081 L988 DIA 3
## 1082 M050 DIA 3
## 1083 M068 DIA 3
## 1084 M073 DIA 3
## 1085 M125 DIA 3
## 1086 M154 DIA 3
## 1087 M171 DIA 3
## 1088 M189 DIA 3
## 1089 M254 DIA 3
## 1090 M311 DIA 3
## 1091 M313 DIA 3
## 1092 M465 DIA 3
## 1093 M489 DIA 3
## 1094 M543 DIA 3
## 1095 M620 DIA 3
## 1096 M623 DIA 3
## 1097 M624 DIA 3
## 1098 M659 DIA 3
## 1099 M712 DIA 3
## 1100 M728 DIA 3
## 1101 M790 DIA 3
## 1102 M863 DIA 3
## 1103 M864 DIA 3
## 1104 M878 DIA 3
## 1105 M939 DIA 3
## 1106 N020 DIA 3
## 1107 N038 DIA 3
## 1108 N079 DIA 3
## 1109 N137 DIA 3
## 1110 N138 DIA 3
## 1111 N144 DIA 3
## 1112 N258 DIA 3
## 1113 N350 DIA 3
## 1114 N44X DIA 3
## 1115 N512 DIA 3
## 1116 N700 DIA 3
## 1117 N701 DIA 3
## 1118 N710 DIA 3
## 1119 N750 DIA 3
## 1120 N758 DIA 3
## 1121 N761 DIA 3
## 1122 N778 DIA 3
## 1123 N835 DIA 3
## 1124 N851 DIA 3
## 1125 N859 DIA 3
## 1126 N898 DIA 3
## 1127 N990 DIA 3
## 1128 O100 DIA 3
## 1129 O16X DIA 3
## 1130 O268 DIA 3
## 1131 O335 DIA 3
## 1132 O367 DIA 3
## 1133 O441 DIA 3
## 1134 O649 DIA 3
## 1135 O669 DIA 3
## 1136 O860 DIA 3
## 1137 O992 DIA 3
## 1138 Q249 DIA 3
## 1139 Q250 DIA 3
## 1140 Q501 DIA 3
## 1141 Q909 DIA 3
## 1142 R030 DIA 3
## 1143 R049 DIA 3
## 1144 R05X DIA 3
## 1145 R060 DIA 3
## 1146 R070 DIA 3
## 1147 R13X DIA 3
## 1148 R162 DIA 3
## 1149 R522 DIA 3
## 1150 R590 DIA 3
## 1151 R730 DIA 3
## 1152 R828 DIA 3
## 1153 S064 DIA 3
## 1154 S066 DIA 3
## 1155 S109 DIA 3
## 1156 S210 DIA 3
## 1157 S224 DIA 3
## 1158 S299 DIA 3
## 1159 S300 DIA 3
## 1160 S313 DIA 3
## 1161 S321 DIA 3
## 1162 S420 DIA 3
## 1163 S430 DIA 3
## 1164 S518 DIA 3
## 1165 S522 DIA 3
## 1166 S618 DIA 3
## 1167 S683 DIA 3
## 1168 S728 DIA 3
## 1169 S730 DIA 3
## 1170 S780 DIA 3
## 1171 S824 DIA 3
## 1172 T116 DIA 3
## 1173 T143 DIA 3
## 1174 T240 DIA 3
## 1175 T301 DIA 3
## 1176 T303 DIA 3
## 1177 T477 DIA 3
## 1178 T509 DIA 3
## 1179 T543 DIA 3
## 1180 T740 DIA 3
## 1181 T784 DIA 3
## 1182 T825 DIA 3
## 1183 W179 DIA 3
## 1184 Y919 DIA 3
## 1185 Z540 DIA 3
## 1186 Z608 DIA 3
## 1187 Z950 DIA 3
## 1188 43498 DIA 2
## 1189 43501 I10X 2
## 1190 43509 DIA 2
## 1191 43525 S889 2
## 1192 43526 I509 2
## 1193 43530 I10X 2
## 1194 43541 I10X 2
## 1195 43551 N390 2
## 1196 43555 DIA 2
## 1197 43562 I10X 2
## 1198 43567 DIA 2
## 1199 43568 E86X 2
## 1200 43569 N390 2
## 1201 43574 DIA 2
## 1202 43579 DIA 2
## 1203 A58X DIA 2
## 1204 A852 DIA 2
## 1205 B878 DIA 2
## 1206 C258 DIA 2
## 1207 C310 DIA 2
## 1208 C348 DIA 2
## 1209 C540 DIA 2
## 1210 C840 DIA 2
## 1211 D042 DIA 2
## 1212 D061 DIA 2
## 1213 D521 DIA 2
## 1214 D642 DIA 2
## 1215 E000 DIA 2
## 1216 E010 DIA 2
## 1217 E012 DIA 2
## 1218 E032 DIA 2
## 1219 E033 DIA 2
## 1220 E034 DIA 2
## 1221 E055 DIA 2
## 1222 E168 DIA 2
## 1223 E200 DIA 2
## 1224 E248 DIA 2
## 1225 E273 DIA 2
## 1226 E274 DIA 2
## 1227 E308 DIA 2
## 1228 E40X DIA 2
## 1229 E45X DIA 2
## 1230 E512 DIA 2
## 1231 E639 DIA 2
## 1232 E662 DIA 2
## 1233 E713 DIA 2
## 1234 E722 DIA 2
## 1235 E748 DIA 2
## 1236 E786 DIA 2
## 1237 E790 DIA 2
## 1238 E832 DIA 2
## 1239 E839 DIA 2
## 1240 E840 DIA 2
## 1241 E849 DIA 2
## 1242 E859 DIA 2
## 1243 E888 DIA 2
## 1244 E890 DIA 2
## 1245 F002 DIA 2
## 1246 F010 DIA 2
## 1247 F012 DIA 2
## 1248 F013 DIA 2
## 1249 F028 DIA 2
## 1250 F072 DIA 2
## 1251 F104 DIA 2
## 1252 F105 DIA 2
## 1253 F132 DIA 2
## 1254 F199 DIA 2
## 1255 F201 DIA 2
## 1256 F206 DIA 2
## 1257 F230 DIA 2
## 1258 F232 DIA 2
## 1259 F250 DIA 2
## 1260 F332 DIA 2
## 1261 F409 DIA 2
## 1262 F429 DIA 2
## 1263 F606 DIA 2
## 1264 F639 DIA 2
## 1265 F709 DIA 2
## 1266 F721 DIA 2
## 1267 F811 DIA 2
## 1268 F99X DIA 2
## 1269 G008 DIA 2
## 1270 G020 DIA 2
## 1271 G030 DIA 2
## 1272 G032 DIA 2
## 1273 G040 DIA 2
## 1274 G051 DIA 2
## 1275 G058 DIA 2
## 1276 G061 DIA 2
## 1277 G062 DIA 2
## 1278 G08X DIA 2
## 1279 G10X DIA 2
## 1280 G122 DIA 2
## 1281 G131 DIA 2
## 1282 G249 DIA 2
## 1283 G253 DIA 2
## 1284 G300 DIA 2
## 1285 G310 DIA 2
## 1286 G328 DIA 2
## 1287 G405 DIA 2
## 1288 G419 DIA 2
## 1289 G432 DIA 2
## 1290 G509 DIA 2
## 1291 G519 DIA 2
## 1292 G529 DIA 2
## 1293 G611 DIA 2
## 1294 G633 DIA 2
## 1295 G638 DIA 2
## 1296 G709 DIA 2
## 1297 G710 DIA 2
## 1298 G802 DIA 2
## 1299 G808 DIA 2
## 1300 G820 DIA 2
## 1301 G903 DIA 2
## 1302 G911 DIA 2
## 1303 G912 DIA 2
## 1304 G930 DIA 2
## 1305 G948 DIA 2
## 1306 G998 B690 2
## 1307 H062 DIA 2
## 1308 H160 DIA 2
## 1309 H162 DIA 2
## 1310 H193 DIA 2
## 1311 H359 DIA 2
## 1312 H431 DIA 2
## 1313 H600 DIA 2
## 1314 H603 DIA 2
## 1315 H620 DIA 2
## 1316 H622 DIA 2
## 1317 H652 DIA 2
## 1318 H700 DIA 2
## 1319 H708 DIA 2
## 1320 H709 DIA 2
## 1321 H730 DIA 2
## 1322 H812 DIA 2
## 1323 H902 DIA 2
## 1324 I052 DIA 2
## 1325 I061 DIA 2
## 1326 I080 DIA 2
## 1327 I201 DIA 2
## 1328 I208 DIA 2
## 1329 I240 DIA 2
## 1330 I248 DIA 2
## 1331 I288 DIA 2
## 1332 I289 DIA 2
## 1333 I311 DIA 2
## 1334 I339 DIA 2
## 1335 I351 DIA 2
## 1336 I352 DIA 2
## 1337 I379 DIA 2
## 1338 I438 DIA 2
## 1339 I452 DIA 2
## 1340 I454 DIA 2
## 1341 I604 DIA 2
## 1342 I606 DIA 2
## 1343 I615 DIA 2
## 1344 I659 R568 2
## 1345 I663 DIA 2
## 1346 I670 DIA 2
## 1347 I690 DIA 2
## 1348 I692 DIA 2
## 1349 I719 DIA 2
## 1350 I724 DIA 2
## 1351 I741 DIA 2
## 1352 I808 DIA 2
## 1353 I849 DIA 2
## 1354 I868 DIA 2
## 1355 I871 DIA 2
## 1356 I879 DIA 2
## 1357 I951 DIA 2
## 1358 I983 DIA 2
## 1359 J010 DIA 2
## 1360 J042 DIA 2
## 1361 J118 DIA 2
## 1362 J15 E11 2
## 1363 J153 DIA 2
## 1364 J154 DIA 2
## 1365 J182 DIA 2
## 1366 J20 DIA 2
## 1367 J210 DIA 2
## 1368 J304 DIA 2
## 1369 J329 DIA 2
## 1370 J371 DIA 2
## 1371 J398 DIA 2
## 1372 J438 DIA 2
## 1373 J458 DIA 2
## 1374 J633 DIA 2
## 1375 J679 DIA 2
## 1376 J691 DIA 2
## 1377 J931 DIA 2
## 1378 J951 DIA 2
## 1379 K040 DIA 2
## 1380 K050 DIA 2
## 1381 K112 DIA 2
## 1382 K222 DIA 2
## 1383 K225 DIA 2
## 1384 K254 DIA 2
## 1385 K264 DIA 2
## 1386 K292 DIA 2
## 1387 K310 DIA 2
## 1388 K317 DIA 2
## 1389 K381 DIA 2
## 1390 K389 DIA 2
## 1391 K403 DIA 2
## 1392 K440 DIA 2
## 1393 K450 DIA 2
## 1394 K460 DIA 2
## 1395 K500 DIA 2
## 1396 K522 DIA 2
## 1397 K550 DIA 2
## 1398 K559 DIA 2
## 1399 K563 DIA 2
## 1400 K572 DIA 2
## 1401 K573 DIA 2
## 1402 K574 DIA 2
## 1403 K589 DIA 2
## 1404 K627 DIA 2
## 1405 K631 DIA 2
## 1406 K635 DIA 2
## 1407 K638 DIA 2
## 1408 K701 DIA 2
## 1409 K718 DIA 2
## 1410 K80 DIA 2
## 1411 K834 DIA 2
## 1412 K900 DIA 2
## 1413 K913 DIA 2
## 1414 K915 DIA 2
## 1415 K918 DIA 2
## 1416 L040 DIA 2
## 1417 L048 DIA 2
## 1418 L10 DIA 2
## 1419 L209 DIA 2
## 1420 L270 A150 2
## 1421 L301 DIA 2
## 1422 L308 DIA 2
## 1423 L510 DIA 2
## 1424 L511 DIA 2
## 1425 L512 DIA 2
## 1426 L739 DIA 2
## 1427 L921 DIA 2
## 1428 L958 DIA 2
## 1429 L982 DIA 2
## 1430 L983 DIA 2
## 1431 M000 DIA 2
## 1432 M013 DIA 2
## 1433 M058 DIA 2
## 1434 M060 DIA 2
## 1435 M104 DIA 2
## 1436 M148 DIA 2
## 1437 M160 DIA 2
## 1438 M190 DIA 2
## 1439 M318 DIA 2
## 1440 M319 DIA 2
## 1441 M328 DIA 2
## 1442 M330 DIA 2
## 1443 M348 DIA 2
## 1444 M349 DIA 2
## 1445 M353 DIA 2
## 1446 M354 DIA 2
## 1447 M359 DIA 2
## 1448 M360 DIA 2
## 1449 M411 DIA 2
## 1450 M45X DIA 2
## 1451 M478 DIA 2
## 1452 M494 DIA 2
## 1453 M511 DIA 2
## 1454 M531 DIA 2
## 1455 M625 DIA 2
## 1456 M626 DIA 2
## 1457 M704 DIA 2
## 1458 M715 DIA 2
## 1459 M720 DIA 2
## 1460 M722 DIA 2
## 1461 M723 DIA 2
## 1462 M724 DIA 2
## 1463 M755 DIA 2
## 1464 M795 DIA 2
## 1465 M844 DIA 2
## 1466 M870 DIA 2
## 1467 M899 DIA 2
## 1468 M940 DIA 2
## 1469 N030 DIA 2
## 1470 N069 DIA 2
## 1471 N078 DIA 2
## 1472 N080 DIA 2
## 1473 N136 DIA 2
## 1474 N20 DIA 2
## 1475 N228 DIA 2
## 1476 N251 DIA 2
## 1477 N290 DIA 2
## 1478 N301 DIA 2
## 1479 N318 DIA 2
## 1480 N321 DIA 2
## 1481 N368 DIA 2
## 1482 N392 DIA 2
## 1483 N40 DIA 2
## 1484 N434 DIA 2
## 1485 N482 DIA 2
## 1486 N485 DIA 2
## 1487 N508 DIA 2
## 1488 N62X DIA 2
## 1489 N63X DIA 2
## 1490 N649 DIA 2
## 1491 N711 DIA 2
## 1492 N719 DIA 2
## 1493 N748 DIA 2
## 1494 N763 DIA 2
## 1495 N766 DIA 2
## 1496 N770 DIA 2
## 1497 N829 DIA 2
## 1498 N841 DIA 2
## 1499 N842 DIA 2
## 1500 N857 DIA 2
## 1501 N888 DIA 2
## 1502 N900 DIA 2
## 1503 N910 DIA 2
## 1504 N912 DIA 2
## 1505 N979 DIA 2
## 1506 N993 DIA 2
## 1507 N994 DIA 2
## 1508 O009 DIA 2
## 1509 O13X DIA 2
## 1510 O235 DIA 2
## 1511 O269 DIA 2
## 1512 O321 DIA 2
## 1513 O366 DIA 2
## 1514 O40X DIA 2
## 1515 O410 DIA 2
## 1516 O601 DIA 2
## 1517 O60X DIA 2
## 1518 O639 DIA 2
## 1519 O730 DIA 2
## 1520 O731 DIA 2
## 1521 O839 DIA 2
## 1522 O911 DIA 2
## 1523 O912 DIA 2
## 1524 Q211 DIA 2
## 1525 Q282 DIA 2
## 1526 Q283 DIA 2
## 1527 Q444 DIA 2
## 1528 Q505 DIA 2
## 1529 Q803 DIA 2
## 1530 R066 DIA 2
## 1531 R073 DIA 2
## 1532 R091 DIA 2
## 1533 R14X DIA 2
## 1534 R229 DIA 2
## 1535 R35X DIA 2
## 1536 R36X DIA 2
## 1537 R451 DIA 2
## 1538 R458 DIA 2
## 1539 R490 DIA 2
## 1540 R578 DIA 2
## 1541 R579 DIA 2
## 1542 R688 DIA 2
## 1543 R740 DIA 2
## 1544 R820 DIA 2
## 1545 R827 DIA 2
## 1546 S001 DIA 2
## 1547 S027 DIA 2
## 1548 S051 DIA 2
## 1549 S059 DIA 2
## 1550 S089 DIA 2
## 1551 S219 DIA 2
## 1552 S220 DIA 2
## 1553 S272 DIA 2
## 1554 S279 DIA 2
## 1555 S301 DIA 2
## 1556 S309 DIA 2
## 1557 S322 DIA 2
## 1558 S364 DIA 2
## 1559 S369 DIA 2
## 1560 S370 DIA 2
## 1561 S421 DIA 2
## 1562 S480 DIA 2
## 1563 S526 DIA 2
## 1564 S609 DIA 2
## 1565 S627 DIA 2
## 1566 S670 DIA 2
## 1567 S699 DIA 2
## 1568 S749 DIA 2
## 1569 S770 DIA 2
## 1570 S808 DIA 2
## 1571 S827 DIA 2
## 1572 S842 DIA 2
## 1573 S903 DIA 2
## 1574 S921 DIA 2
## 1575 S930 DIA 2
## 1576 S971 DIA 2
## 1577 S998 DIA 2
## 1578 T008 DIA 2
## 1579 T055 DIA 2
## 1580 T093 DIA 2
## 1581 T111 DIA 2
## 1582 T130 DIA 2
## 1583 T202 DIA 2
## 1584 T222 DIA 2
## 1585 T245 DIA 2
## 1586 T246 DIA 2
## 1587 T250 DIA 2
## 1588 T292 DIA 2
## 1589 T310 DIA 2
## 1590 T348 DIA 2
## 1591 T451 DIA 2
## 1592 T671 DIA 2
## 1593 T68X DIA 2
## 1594 T709 DIA 2
## 1595 T782 DIA 2
## 1596 T809 DIA 2
## 1597 T829 DIA 2
## 1598 T861 DIA 2
## 1599 T868 DIA 2
## 1600 T871 DIA 2
## 1601 T886 DIA 2
## 1602 T930 DIA 2
## 1603 T983 DIA 2
## 1604 U069 DIA 2
## 1605 W010 DIA 2
## 1606 X259 DIA 2
## 1607 Y579 DIA 2
## 1608 Y841 DIA 2
## 1609 Y912 DIA 2
## 1610 Z100 DIA 2
## 1611 Z359 DIA 2
## 1612 Z433 DIA 2
## 1613 Z896 DIA 2
## 1614 Z992 DIA 2
## 1615 B178 B029 1
## 1616 B207 A090 1
## 1617 B358 B24X 1
## 1618 B450 B24X 1
## 1619 B451 B009 1
## 1620 B779 A071 1
## 1621 C091 A419 1
## 1622 C460 B24X 1
## 1623 C787 C189 1
## 1624 C919 C910 1
## 1625 D070 D060 1
## 1626 D199 B370 1
## 1627 D25 C64X 1
## 1628 D339 C719 1
## 1629 D373 A162 1
## 1630 D382 A165 1
## 1631 E011 DIA 1
## 1632 E030 DIA 1
## 1633 E040 DIA 1
## 1634 E060 DIA 1
## 1635 E063 DIA 1
## 1636 E079 DIA 1
## 1637 E11.6 DIA 1
## 1638 E111D D250 1
## 1639 E208 DIA 1
## 1640 E211 DIA 1
## 1641 E233 DIA 1
## 1642 E237 DIA 1
## 1643 E260 D509 1
## 1644 E282 D432 1
## 1645 E344 DIA 1
## 1646 E348 DIA 1
## 1647 E350 DIA 1
## 1648 E43 C169 1
## 1649 E449 DIA 1
## 1650 E539 DIA 1
## 1651 E60X DIA 1
## 1652 E649 A419 1
## 1653 E709 DIA 1
## 1654 E720 DIA 1
## 1655 E728 DIA 1
## 1656 E729 DIA 1
## 1657 E738 D509 1
## 1658 E742 DIA 1
## 1659 E768 DIA 1
## 1660 E800 DIA 1
## 1661 E804 DIA 1
## 1662 E806 A090 1
## 1663 E807 DIA 1
## 1664 E850 DIA 1
## 1665 E882 DIA 1
## 1666 E893 DIA 1
## 1667 E90X DIA 1
## 1668 F000 DIA 1
## 1669 F020 DIA 1
## 1670 F023 DIA 1
## 1671 F051 DIA 1
## 1672 F068 DIA 1
## 1673 F070 DIA 1
## 1674 F078 DIA 1
## 1675 F079 DIA 1
## 1676 F106 DIA 1
## 1677 F116 DIA 1
## 1678 F129 DIA 1
## 1679 F135 D509 1
## 1680 F141 DIA 1
## 1681 F171 DIA 1
## 1682 F189 DIA 1
## 1683 F190 DIA 1
## 1684 F194 DIA 1
## 1685 F203 DIA 1
## 1686 F20X DIA 1
## 1687 F21X D500 1
## 1688 F229 DIA 1
## 1689 F239 DIA 1
## 1690 F310 DIA 1
## 1691 F311 DIA 1
## 1692 F313 DIA 1
## 1693 F316 DIA 1
## 1694 F330 DIA 1
## 1695 F41 DIA 1
## 1696 F413 DIA 1
## 1697 F418 DIA 1
## 1698 F440 DIA 1
## 1699 F444 DIA 1
## 1700 F459 DIA 1
## 1701 F481 DIA 1
## 1702 F500 DIA 1
## 1703 F504 DIA 1
## 1704 F509 DIA 1
## 1705 F510 DIA 1
## 1706 F514 DIA 1
## 1707 F519 DIA 1
## 1708 F603 DIA 1
## 1709 F604 DIA 1
## 1710 F608 DIA 1
## 1711 F61X DIA 1
## 1712 F669 DIA 1
## 1713 F708 DIA 1
## 1714 F719 DIA 1
## 1715 F729 DIA 1
## 1716 F780 DIA 1
## 1717 F790 DIA 1
## 1718 F800 DIA 1
## 1719 F808 DIA 1
## 1720 F82X DIA 1
## 1721 F844 DIA 1
## 1722 F900 DIA 1
## 1723 F980 DIA 1
## 1724 G000 DIA 1
## 1725 G112 DIA 1
## 1726 G114 DIA 1
## 1727 G129 DIA 1
## 1728 G20 DIA 1
## 1729 G211 DIA 1
## 1730 G218 DIA 1
## 1731 G22X DIA 1
## 1732 G231 DIA 1
## 1733 G250 DIA 1
## 1734 G258 DIA 1
## 1735 G368 DIA 1
## 1736 G369 DIA 1
## 1737 G370 DIA 1
## 1738 G372 DIA 1
## 1739 G379 DIA 1
## 1740 G412 DIA 1
## 1741 G418 DIA 1
## 1742 G440 DIA 1
## 1743 G441 DIA 1
## 1744 G444 DIA 1
## 1745 G45 DIA 1
## 1746 G452 DIA 1
## 1747 G460 DIA 1
## 1748 G462 DIA 1
## 1749 G465 D180 1
## 1750 G468 DIA 1
## 1751 G473 DIA 1
## 1752 G528 DIA 1
## 1753 G538 DIA 1
## 1754 G540 DIA 1
## 1755 G544 DIA 1
## 1756 G560 DIA 1
## 1757 G561 DIA 1
## 1758 G580 A010 1
## 1759 G603 DIA 1
## 1760 G64X DIA 1
## 1761 G713 DIA 1
## 1762 G800 DIA 1
## 1763 G902 DIA 1
## 1764 G908 DIA 1
## 1765 G918 DIA 1
## 1766 G941 D432 1
## 1767 G951 DIA 1
## 1768 G961 DIA 1
## 1769 G968 DIA 1
## 1770 H000 DIA 1
## 1771 H027 DIA 1
## 1772 H031 B023 1
## 1773 H045 DIA 1
## 1774 H100 DIA 1
## 1775 H105 DIA 1
## 1776 H150 A539 1
## 1777 H151 DIA 1
## 1778 H169 DIA 1
## 1779 H189 C539 1
## 1780 H209 DIA 1
## 1781 H220 A514 1
## 1782 H250 DIA 1
## 1783 H262 DIA 1
## 1784 H282 DIA 1
## 1785 H309 DIA 1
## 1786 H335 A090 1
## 1787 H428 DIA 1
## 1788 H438 D649 1
## 1789 H448 DIA 1
## 1790 H449 DIA 1
## 1791 H451 DIA 1
## 1792 H472 DIA 1
## 1793 H492 DIA 1
## 1794 H493 DIA 1
## 1795 H494 DIA 1
## 1796 H521 DIA 1
## 1797 H523 DIA 1
## 1798 H524 DIA 1
## 1799 H530 DIA 1
## 1800 H533 DIA 1
## 1801 H542 DIA 1
## 1802 H578 DIA 1
## 1803 H598 DIA 1
## 1804 H604 DIA 1
## 1805 H605 DIA 1
## 1806 H60X DIA 1
## 1807 H611 DIA 1
## 1808 H678 DIA 1
## 1809 H818 DIA 1
## 1810 H830 DIA 1
## 1811 H901 A161 1
## 1812 H905 DIA 1
## 1813 H911 DIA 1
## 1814 H913 DIA 1
## 1815 H933 DIA 1
## 1816 I010 DIA 1
## 1817 I018 DIA 1
## 1818 I070 DIA 1
## 1819 I081 DIA 1
## 1820 I091 DIA 1
## 1821 I12 DIA 1
## 1822 I21 D50 1
## 1823 I236 DIA 1
## 1824 I253 DIA 1
## 1825 I254 DIA 1
## 1826 I272 DIA 1
## 1827 I28 E11 1
## 1828 I280 DIA 1
## 1829 I300 C300 1
## 1830 I301 DIA 1
## 1831 I313 A159 1
## 1832 I319 D649 1
## 1833 I321 DIA 1
## 1834 I348 DIA 1
## 1835 I361 DIA 1
## 1836 I369 DIA 1
## 1837 I372 DIA 1
## 1838 I400 DIA 1
## 1839 I409 DIA 1
## 1840 I411 DIA 1
## 1841 I422 DIA 1
## 1842 I424 DIA 1
## 1843 I432 DIA 1
## 1844 I453 DIA 1
## 1845 I456 DIA 1
## 1846 I461 DIA 1
## 1847 I470 DIA 1
## 1848 I472 DIA 1
## 1849 I493 DIA 1
## 1850 I513 DIA 1
## 1851 I616 DIA 1
## 1852 I636 DIA 1
## 1853 I650 DIA 1
## 1854 I651 DIA 1
## 1855 I652 DIA 1
## 1856 I661 DIA 1
## 1857 I664 DIA 1
## 1858 I669 DIA 1
## 1859 I676 DIA 1
## 1860 I69 DIA 1
## 1861 I708 DIA 1
## 1862 I718 DIA 1
## 1863 I720 DIA 1
## 1864 I721 DIA 1
## 1865 I730 DIA 1
## 1866 I745 DIA 1
## 1867 I748 DIA 1
## 1868 I772 DIA 1
## 1869 I790 DIA 1
## 1870 I81X DIA 1
## 1871 I840 DIA 1
## 1872 I848 D649 1
## 1873 I863 DIA 1
## 1874 I870 DIA 1
## 1875 I898 DIA 1
## 1876 I950 DIA 1
## 1877 I988 DIA 1
## 1878 J020 DIA 1
## 1879 J028 DIA 1
## 1880 J030 DIA 1
## 1881 J038 DIA 1
## 1882 J041 DIA 1
## 1883 J06 DIA 1
## 1884 J060 DIA 1
## 1885 J09X DIA 1
## 1886 J110 D509 1
## 1887 J111 DIA 1
## 1888 J122 DIA 1
## 1889 J172 DIA 1
## 1890 J173 B59X 1
## 1891 J18 E11 1
## 1892 J20X DIA 1
## 1893 J211 DIA 1
## 1894 J22 E11 1
## 1895 J312 DIA 1
## 1896 J320 DIA 1
## 1897 J321 DIA 1
## 1898 J330 DIA 1
## 1899 J339 DIA 1
## 1900 J359 DIA 1
## 1901 J370 DIA 1
## 1902 J382 DIA 1
## 1903 J391 DIA 1
## 1904 J392 DIA 1
## 1905 J40 E11 1
## 1906 J439 D649 1
## 1907 J45 E11 1
## 1908 J45X DIA 1
## 1909 J47 DIA 1
## 1910 J60X DIA 1
## 1911 J620 DIA 1
## 1912 J628 DIA 1
## 1913 J65X DIA 1
## 1914 J671 DIA 1
## 1915 J677 DIA 1
## 1916 J684 DIA 1
## 1917 J953 DIA 1
## 1918 J958 DIA 1
## 1919 J982 DIA 1
## 1920 J986 DIA 1
## 1921 J98O DIA 1
## 1922 J990 DIA 1
## 1923 K021 DIA 1
## 1924 K039 B374 1
## 1925 K051 DIA 1
## 1926 K053 DIA 1
## 1927 K068 DIA 1
## 1928 K088 DIA 1
## 1929 K092 DIA 1
## 1930 K099 DIA 1
## 1931 K103 DIA 1
## 1932 K109 DIA 1
## 1933 K118 DIA 1
## 1934 K119 A09X 1
## 1935 K120 C859 1
## 1936 K121 DIA 1
## 1937 K123 A150 1
## 1938 K20 DIA 1
## 1939 K220 A150 1
## 1940 K226 DIA 1
## 1941 K25 D53 1
## 1942 k250 DIA 1
## 1943 K251 DIA 1
## 1944 K252 DIA 1
## 1945 K255 DIA 1
## 1946 K256 DIA 1
## 1947 K263 DIA 1
## 1948 K267 DIA 1
## 1949 K274 DIA 1
## 1950 K311 D649 1
## 1951 K314 DIA 1
## 1952 K383 DIA 1
## 1953 K40 C61 1
## 1954 K404 DIA 1
## 1955 K410 DIA 1
## 1956 K419 DIA 1
## 1957 K430 DIA 1
## 1958 K509 DIA 1
## 1959 K510 DIA 1
## 1960 K515 DIA 1
## 1961 K520 DIA 1
## 1962 K521 DIA 1
## 1963 K528 DIA 1
## 1964 K552 DIA 1
## 1965 K578 DIA 1
## 1966 K580 DIA 1
## 1967 K59 DIA 1
## 1968 K592 DIA 1
## 1969 K593 DIA 1
## 1970 K599 DIA 1
## 1971 K601 DIA 1
## 1972 K604 DIA 1
## 1973 K611 DIA 1
## 1974 K620 DIA 1
## 1975 K624 DIA 1
## 1976 K633 D500 1
## 1977 K640 DIA 1
## 1978 K641 DIA 1
## 1979 K66 E14 1
## 1980 K711 DIA 1
## 1981 K713 A099 1
## 1982 K714 DIA 1
## 1983 K716 DIA 1
## 1984 K730 DIA 1
## 1985 K738 DIA 1
## 1986 K752 A090 1
## 1987 K753 DIA 1
## 1988 K754 DIA 1
## 1989 K76 E14 1
## 1990 K761 DIA 1
## 1991 K770 D509 1
## 1992 K80X DIA 1
## 1993 K81X B24X 1
## 1994 K822 D619 1
## 1995 K824 DIA 1
## 1996 K832 DIA 1
## 1997 K86 DIA 1
## 1998 K90 E14 1
## 1999 K938 DIA 1
## 2000 L059 DIA 1
## 2001 L08 DIA 1
## 2002 L081 D508 1
## 2003 L108 DIA 1
## 2004 L121 D649 1
## 2005 L129 DIA 1
## 2006 L139 DIA 1
## 2007 L14X DIA 1
## 2008 L200 DIA 1
## 2009 L208 DIA 1
## 2010 L210 DIA 1
## 2011 L22X DIA 1
## 2012 L231 D509 1
## 2013 L238 DIA 1
## 2014 L249 DIA 1
## 2015 L26 A499 1
## 2016 L271 DIA 1
## 2017 L280 DIA 1
## 2018 L282 DIA 1
## 2019 L292 DIA 1
## 2020 L298 DIA 1
## 2021 L300 DIA 1
## 2022 L414 DIA 1
## 2023 L480 DIA 1
## 2024 L501 DIA 1
## 2025 L502 DIA 1
## 2026 L508 DIA 1
## 2027 L519 D649 1
## 2028 L538 D649 1
## 2029 L551 DIA 1
## 2030 L570 DIA 1
## 2031 L600 DIA 1
## 2032 L602 DIA 1
## 2033 L603 DIA 1
## 2034 L640 DIA 1
## 2035 L680 DIA 1
## 2036 L702 DIA 1
## 2037 L817 DIA 1
## 2038 L819 A099 1
## 2039 L82X DIA 1
## 2040 L84X DIA 1
## 2041 L853 DIA 1
## 2042 L871 DIA 1
## 2043 L903 DIA 1
## 2044 L905 DIA 1
## 2045 L929 DIA 1
## 2046 L931 DIA 1
## 2047 L932 DIA 1
## 2048 L940 DIA 1
## 2049 L980 B180 1
## 2050 L998 DIA 1
## 2051 M008 DIA 1
## 2052 M018 DIA 1
## 2053 M030 DIA 1
## 2054 M052 DIA 1
## 2055 M064 DIA 1
## 2056 M082 DIA 1
## 2057 M103 DIA 1
## 2058 M112 DIA 1
## 2059 M120 DIA 1
## 2060 M138 DIA 1
## 2061 M142 DIA 1
## 2062 M145 DIA 1
## 2063 M153 DIA 1
## 2064 M159 A090 1
## 2065 M161 DIA 1
## 2066 M172 DIA 1
## 2067 M175 DIA 1
## 2068 M198 DIA 1
## 2069 M204 DIA 1
## 2070 M215 DIA 1
## 2071 M224 DIA 1
## 2072 M231 DIA 1
## 2073 M232 DIA 1
## 2074 M243 DIA 1
## 2075 M245 DIA 1
## 2076 M250 DIA 1
## 2077 M259 DIA 1
## 2078 M316 DIA 1
## 2079 M317 DIA 1
## 2080 M340 DIA 1
## 2081 M350 DIA 1
## 2082 M361 DIA 1
## 2083 M413 DIA 1
## 2084 M418 DIA 1
## 2085 M421 A498 1
## 2086 M429 DIA 1
## 2087 M462 DIA 1
## 2088 M47 E14 1
## 2089 M470 DIA 1
## 2090 M471 DIA 1
## 2091 M488 DIA 1
## 2092 M502 DIA 1
## 2093 M510 DIA 1
## 2094 M518 DIA 1
## 2095 M530 DIA 1
## 2096 M540 D530 1
## 2097 M546 DIA 1
## 2098 M548 DIA 1
## 2099 M621 DIA 1
## 2100 M650 DIA 1
## 2101 M651 DIA 1
## 2102 M653 DIA 1
## 2103 M658 DIA 1
## 2104 M662 DIA 1
## 2105 M688 DIA 1
## 2106 M701 DIA 1
## 2107 M703 DIA 1
## 2108 M711 DIA 1
## 2109 M719 DIA 1
## 2110 M751 D693 1
## 2111 M752 DIA 1
## 2112 M754 DIA 1
## 2113 M758 DIA 1
## 2114 M760 D500 1
## 2115 M761 DIA 1
## 2116 M762 DIA 1
## 2117 M763 DIA 1
## 2118 M766 DIA 1
## 2119 M773 DIA 1
## 2120 M792 DIA 1
## 2121 M796 DIA 1
## 2122 M797 DIA 1
## 2123 M800 DIA 1
## 2124 M841 DIA 1
## 2125 M848 DIA 1
## 2126 M850 DIA 1
## 2127 M854 C050 1
## 2128 M862 A419 1
## 2129 M865 DIA 1
## 2130 M898 DIA 1
## 2131 M900 DIA 1
## 2132 M901 DIA 1
## 2133 M902 DIA 1
## 2134 M903 DIA 1
## 2135 M906 DIA 1
## 2136 M922 D500 1
## 2137 M928 DIA 1
## 2138 M948 DIA 1
## 2139 M952 DIA 1
## 2140 M960 DIA 1
## 2141 M966 DIA 1
## 2142 M990 DIA 1
## 2143 M995 DIA 1
## 2144 N002 DIA 1
## 2145 N021 DIA 1
## 2146 N025 DIA 1
## 2147 N028 DIA 1
## 2148 N042 DIA 1
## 2149 N058 DIA 1
## 2150 N063 A09X 1
## 2151 N088 DIA 1
## 2152 N10 DIA 1
## 2153 N12 DIA 1
## 2154 N134 DIA 1
## 2155 N135 DIA 1
## 2156 N140 DIA 1
## 2157 N150 DIA 1
## 2158 N164 DIA 1
## 2159 N171 DIA 1
## 2160 N21 E13 1
## 2161 N222 DIA 1
## 2162 N26X D649 1
## 2163 N298 DIA 1
## 2164 N302 DIA 1
## 2165 N303 DIA 1
## 2166 N304 D501 1
## 2167 N308 DIA 1
## 2168 N329 A049 1
## 2169 N341 DIA 1
## 2170 N342 DIA 1
## 2171 N358 DIA 1
## 2172 N360 DIA 1
## 2173 N362 DIA 1
## 2174 N369 A419 1
## 2175 N370 DIA 1
## 2176 N418 DIA 1
## 2177 N46 DIA 1
## 2178 N48 DIA 1
## 2179 N500 DIA 1
## 2180 N509 DIA 1
## 2181 N600 DIA 1
## 2182 N601 DIA 1
## 2183 N61 E14 1
## 2184 N645 DIA 1
## 2185 N730 DIA 1
## 2186 N731 D589 1
## 2187 N732 D539 1
## 2188 N734 DIA 1
## 2189 N76 DIA 1
## 2190 N765 D049 1
## 2191 N808 DIA 1
## 2192 N828 DIA 1
## 2193 N831 DIA 1
## 2194 N8324 DIA 1
## 2195 N838 DIA 1
## 2196 N839 DIA 1
## 2197 N849 DIA 1
## 2198 N855 DIA 1
## 2199 N890 DIA 1
## 2200 N891 DIA 1
## 2201 N908 DIA 1
## 2202 N909 DIA 1
## 2203 N949 DIA 1
## 2204 N950 D649 1
## 2205 N951 E10X 1
## 2206 N970 DIA 1
## 2207 N992 DIA 1
## 2208 N998 DIA 1
## 2209 O000 DIA 1
## 2210 O019 DIA 1
## 2211 O030 DIA 1
## 2212 O033 DIA 1
## 2213 O039 DIA 1
## 2214 O054 DIA 1
## 2215 O088 DIA 1
## 2216 O219 DIA 1
## 2217 O220 DIA 1
## 2218 O231 DIA 1
## 2219 O239 DIA 1
## 2220 O243 DIA 1
## 2221 O244 DIA 1
## 2222 O25X DIA 1
## 2223 O300 DIA 1
## 2224 O323 DIA 1
## 2225 O324 DIA 1
## 2226 O326 DIA 1
## 2227 O330 DIA 1
## 2228 O334 DIA 1
## 2229 O338 DIA 1
## 2230 O339 DIA 1
## 2231 O365 DIA 1
## 2232 O368 DIA 1
## 2233 O420 DIA 1
## 2234 O421 DIA 1
## 2235 O439 DIA 1
## 2236 O471 DIA 1
## 2237 O610 DIA 1
## 2238 O624 DIA 1
## 2239 O645 DIA 1
## 2240 O648 DIA 1
## 2241 O662 DIA 1
## 2242 O679 DIA 1
## 2243 O700 DIA 1
## 2244 O722 DIA 1
## 2245 O759 DIA 1
## 2246 O8001 DIA 1
## 2247 O808 DIA 1
## 2248 O8284 DIA 1
## 2249 O838 DIA 1
## 2250 O85X DIA 1
## 2251 O862 DIA 1
## 2252 O863 DIA 1
## 2253 O868 DIA 1
## 2254 O900 DIA 1
## 2255 O994 DIA 1
## 2256 P95X DIA 1
## 2257 Q019 DIA 1
## 2258 Q046 DIA 1
## 2259 Q049 DIA 1
## 2260 Q219 DIA 1
## 2261 Q253 DIA 1
## 2262 Q259 DIA 1
## 2263 Q273 DIA 1
## 2264 Q279 DIA 1
## 2265 Q400 DIA 1
## 2266 Q603 DIA 1
## 2267 Q605 DIA 1
## 2268 Q612 C609 1
## 2269 Q620 DIA 1
## 2270 Q631 D410 1
## 2271 Q660 DIA 1
## 2272 Q720 C300 1
## 2273 Q738 DIA 1
## 2274 Q780 DIA 1
## 2275 Q785 DIA 1
## 2276 Q810 DIA 1
## 2277 Q828 DIA 1
## 2278 Q831 DIA 1
## 2279 Q850 DIA 1
## 2280 Q871 DIA 1
## 2281 Q874 DIA 1
## 2282 Q96 DIA 1
## 2283 R011 DIA 1
## 2284 R02 D509 1
## 2285 R031 DIA 1
## 2286 R068 DIA 1
## 2287 R071 DIA 1
## 2288 R090 DIA 1
## 2289 R098 DIA 1
## 2290 R12X DIA 1
## 2291 R198 DIA 1
## 2292 R222 DIA 1
## 2293 R224 DIA 1
## 2294 R270 DIA 1
## 2295 R301 D509 1
## 2296 R34X DIA 1
## 2297 R390 DIA 1
## 2298 R398 DIA 1
## 2299 R400 DIA 1
## 2300 R470 DIA 1
## 2301 R478 DIA 1
## 2302 R488 DIA 1
## 2303 R51 DIA 1
## 2304 R54 A415 1
## 2305 R599 DIA 1
## 2306 R634 DIA 1
## 2307 R749 DIA 1
## 2308 R785 DIA 1
## 2309 R799 DIA 1
## 2310 R831 A152 1
## 2311 S007 DIA 1
## 2312 S014 DIA 1
## 2313 S015 DIA 1
## 2314 S017 DIA 1
## 2315 S018 DIA 1
## 2316 S025 DIA 1
## 2317 S026 DIA 1
## 2318 S029 DIA 1
## 2319 S031 DIA 1
## 2320 S045 DIA 1
## 2321 S055 B370 1
## 2322 S060 DIA 1
## 2323 S061 DIA 1
## 2324 S063 DIA 1
## 2325 S068 DIA 1
## 2326 S100 DIA 1
## 2327 S127 DIA 1
## 2328 S129 DIA 1
## 2329 S143 DIA 1
## 2330 S151 DIA 1
## 2331 S159 DIA 1
## 2332 S203 DIA 1
## 2333 S218 DIA 1
## 2334 S228 DIA 1
## 2335 S230 DIA 1
## 2336 S270 DIA 1
## 2337 S298 DIA 1
## 2338 S302 DIA 1
## 2339 S308 DIA 1
## 2340 S312 DIA 1
## 2341 S315 DIA 1
## 2342 S318 DIA 1
## 2343 S323 DIA 1
## 2344 S325 D648 1
## 2345 S327 DIA 1
## 2346 S340 DIA 1
## 2347 S341 DIA 1
## 2348 S344 DIA 1
## 2349 S359 D62X 1
## 2350 S361 D649 1
## 2351 S372 DIA 1
## 2352 S390 DIA 1
## 2353 S398 D62X 1
## 2354 S400 DIA 1
## 2355 S407 DIA 1
## 2356 S408 DIA 1
## 2357 S410 DIA 1
## 2358 S418 DIA 1
## 2359 S429 DIA 1
## 2360 S431 DIA 1
## 2361 S459 DIA 1
## 2362 S460 DIA 1
## 2363 S481 DIA 1
## 2364 S489 DIA 1
## 2365 S507 DIA 1
## 2366 S521 DIA 1
## 2367 S523 DIA 1
## 2368 S531 D649 1
## 2369 S550 DIA 1
## 2370 S589 DIA 1
## 2371 S607 DIA 1
## 2372 S623 DIA 1
## 2373 S635 DIA 1
## 2374 S644 DIA 1
## 2375 S668 DIA 1
## 2376 S678 DIA 1
## 2377 S684 DIA 1
## 2378 S689 DIA 1
## 2379 S708 DIA 1
## 2380 S717 DIA 1
## 2381 S748 DIA 1
## 2382 S799 DIA 1
## 2383 S801 DIA 1
## 2384 S809 DIA 1
## 2385 S832 DIA 1
## 2386 S849 DIA 1
## 2387 S860 DIA 1
## 2388 S899 DIA 1
## 2389 S900 DIA 1
## 2390 S902 DIA 1
## 2391 S907 DIA 1
## 2392 S922 DIA 1
## 2393 S923 DIA 1
## 2394 S927 DIA 1
## 2395 S934 DIA 1
## 2396 S936 A480 1
## 2397 S948 DIA 1
## 2398 S978 DIA 1
## 2399 S999 DIA 1
## 2400 T000 DIA 1
## 2401 T013 DIA 1
## 2402 T018 DIA 1
## 2403 T050 DIA 1
## 2404 T058 DIA 1
## 2405 T068 DIA 1
## 2406 T099 DIA 1
## 2407 T135 DIA 1
## 2408 T138 DIA 1
## 2409 T145 DIA 1
## 2410 T148 DIA 1
## 2411 T150 DIA 1
## 2412 T180 DIA 1
## 2413 T189 DIA 1
## 2414 T191 DIA 1
## 2415 T210 DIA 1
## 2416 T212 DIA 1
## 2417 T230 DIA 1
## 2418 T231 DIA 1
## 2419 T291 DIA 1
## 2420 T293 DIA 1
## 2421 T300 DIA 1
## 2422 T304 DIA 1
## 2423 T315 DIA 1
## 2424 T365 DIA 1
## 2425 T368 DIA 1
## 2426 T441 DIA 1
## 2427 T443 DIA 1
## 2428 T479 DIA 1
## 2429 T528 DIA 1
## 2430 T541 DIA 1
## 2431 T542 DIA 1
## 2432 T58X DIA 1
## 2433 T601 DIA 1
## 2434 T604 DIA 1
## 2435 T635 DIA 1
## 2436 T670 DIA 1
## 2437 T674 DIA 1
## 2438 T698 DIA 1
## 2439 T741 DIA 1
## 2440 T751 DIA 1
## 2441 T78 DIA 1
## 2442 T783 DIA 1
## 2443 T789 DIA 1
## 2444 T794 DIA 1
## 2445 T801 DIA 1
## 2446 T802 D649 1
## 2447 T803 D689 1
## 2448 T808 DIA 1
## 2449 T815 DIA 1
## 2450 T818 DIA 1
## 2451 T819 DIA 1
## 2452 T833 DIA 1
## 2453 T835 D649 1
## 2454 T848 DIA 1
## 2455 T859 DIA 1
## 2456 T869 DIA 1
## 2457 T889 DIA 1
## 2458 T935 DIA 1
## 2459 T936 DIA 1
## 2460 U202 DIA 1
## 2461 U205 DIA 1
## 2462 V099 DIA 1
## 2463 V385 DIA 1
## 2464 W018 DIA 1
## 2465 W060 DIA 1
## 2466 W079 DIA 1
## 2467 W089 DIA 1
## 2468 W159 DIA 1
## 2469 W170 DIA 1
## 2470 W180 DIA 1
## 2471 W184 DIA 1
## 2472 W204 DIA 1
## 2473 W229 DIA 1
## 2474 X100 DIA 1
## 2475 X109 DIA 1
## 2476 X689 A059 1
## 2477 X690 DIA 1
## 2478 X699 DIA 1
## 2479 X954 DIA 1
## 2480 Y279 DIA 1
## 2481 Y411 A153 1
## 2482 Y423 DIA 1
## 2483 Y822 DIA 1
## 2484 Y834 DIA 1
## 2485 Y846 DIA 1
## 2486 Y913 DIA 1
## 2487 Z226 B86X 1
## 2488 Z3593 DIA 1
## 2489 Z391 DIA 1
## 2490 Z450 DIA 1
## 2491 Z730 DIA 1
## 2492 Z749 DIA 1
## 2493 Z930 DIA 1
## 2494 Z932 DIA 1
## 2495 Z958 DIA 1

### Dead patients, before lockdown

## Diag1 Diag2 Frequency
## 1 A419 DIA 616
## 2 J189 DIA 278
## 3 J960 DIA 277
## 4 N189 DIA 226
## 5 I10X DIA 197
## 6 J969 DIA 187
## 7 N390 DIA 138
## 8 A418 DIA 97
## 9 I219 DIA 65
## 10 I500 DIA 59
## 11 J159 DIA 57
## 12 N185 DIA 55
## 13 R572 DIA 51
## 14 I64X DIA 47
## 15 I469 DIA 43
## 16 K746 DIA 39
## 17 N179 DIA 38
## 18 J690 DIA 37
## 19 J961 DIA 37
## 20 G934 DIA 31
## 21 N19X DIA 30
## 22 I639 DIA 27
## 23 K922 DIA 27
## 24 I509 DIA 26
## 25 J841 DIA 24
## 26 R570 DIA 24
## 27 E872 DIA 20
## 28 I678 DIA 20
## 29 I679 DIA 20
## 30 I619 DIA 19
## 31 N039 DIA 19
## 32 E162 DIA 17
## 33 R579 DIA 16
## 34 E039 DIA 15
## 35 J81X DIA 15
## 36 A409 DIA 14
## 37 E86X DIA 14
## 38 I269 DIA 14
## 39 J90X DIA 14
## 40 R571 DIA 14
## 41 R688 DIA 14
## 42 D509 DIA 13
## 43 J849 DIA 13
## 44 R02X DIA 13
## 45 E46X DIA 12
## 46 I499 DIA 12
## 47 K703 DIA 12
## 48 I489 DIA 11
## 49 I694 DIA 11
## 50 J180 DIA 11
## 51 K729 DIA 11
## 52 I119 DIA 10
## 53 I480 DIA 10
## 54 J13X DIA 10
## 55 J80X DIA 10
## 56 D539 DIA 9
## 57 E669 DIA 9
## 58 J188 DIA 9
## 59 M726 DIA 9
## 60 N10X DIA 9
## 61 R578 DIA 9
## 62 R69X DIA 9
## 63 L031 DIA 8
## 64 L899 DIA 8
## 65 G932 DIA 7
## 66 I120 DIA 7
## 67 S069 DIA 7
## 68 G459 DIA 6
## 69 G969 DIA 6
## 70 I210 DIA 6
## 71 I490 DIA 6
## 72 I609 DIA 6
## 73 J91X DIA 6
## 74 K830 DIA 6
## 75 L890 DIA 6
## 76 L893 DIA 6
## 77 M725 DIA 6
## 78 N184 DIA 6
## 79 R092 DIA 6
## 80 C250 DIA 5
## 81 E43X DIA 5
## 82 E440 DIA 5
## 83 I48X DIA 5
## 84 I612 DIA 5
## 85 J449 DIA 5
## 86 J47X DIA 5
## 87 K566 DIA 5
## 88 K650 DIA 5
## 89 N110 DIA 5
## 90 R739 DIA 5
## 91 R99X DIA 5
## 92 T136 DIA 5
## 93 E835 DIA 4
## 94 E875 DIA 4
## 95 G931 DIA 4
## 96 I749 DIA 4
## 97 J150 DIA 4
## 98 L089 DIA 4
## 99 L984 DIA 4
## 100 N12X DIA 4
## 101 N178 DIA 4
## 102 N180 DIA 4
## 103 R960 DIA 4
## 104 E871 DIA 3
## 105 E889 DIA 3
## 106 F069 DIA 3
## 107 G042 DIA 3
## 108 G409 DIA 3
## 109 I259 DIA 3
## 110 I340 DIA 3
## 111 I461 DIA 3
## 112 I630 DIA 3
## 113 I633 DIA 3
## 114 I693 DIA 3
## 115 I739 DIA 3
## 116 J123 DIA 3
## 117 J151 DIA 3
## 118 J158 DIA 3
## 119 J168 DIA 3
## 120 J679 DIA 3
## 121 J698 DIA 3
## 122 J869 DIA 3
## 123 J939 DIA 3
## 124 K659 DIA 3
## 125 K769 DIA 3
## 126 L022 DIA 3
## 127 L033 DIA 3
## 128 L039 DIA 3
## 129 M729 DIA 3
## 130 R100 DIA 3
## 131 R17X DIA 3
## 132 R18X DIA 3
## 133 R392 DIA 3
## 134 R650 DIA 3
## 135 S720 DIA 3
## 136 S889 DIA 3
## 137 T054 DIA 3
## 138 T874 DIA 3
## 139 C900 DIA 2
## 140 E161 DIA 2
## 141 E876 A419 2
## 142 E878 DIA 2
## 143 F050 DIA 2
## 144 F078 DIA 2
## 145 F09X DIA 2
## 146 F102 DIA 2
## 147 F130 DIA 2
## 148 G009 DIA 2
## 149 G92X DIA 2
## 150 G936 DIA 2
## 151 I051 DIA 2
## 152 I059 DIA 2
## 153 I110 DIA 2
## 154 I159 DIA 2
## 155 I200 DIA 2
## 156 I211 DIA 2
## 157 I255 DIA 2
## 158 I260 DIA 2
## 159 I460 DIA 2
## 160 I632 DIA 2
## 161 I743 DIA 2
## 162 I802 DIA 2
## 163 I959 DIA 2
## 164 J129 DIA 2
## 165 J42X DIA 2
## 166 J440 DIA 2
## 167 J459 DIA 2
## 168 J631 DIA 2
## 169 J680 A419 2
## 170 J840 DIA 2
## 171 J851 DIA 2
## 172 J989 DIA 2
## 173 K631 DIA 2
## 174 K709 DIA 2
## 175 K750 DIA 2
## 176 K767 DIA 2
## 177 K859 DIA 2
## 178 K929 DIA 2
## 179 L030 DIA 2
## 180 L038 DIA 2
## 181 L409 DIA 2
## 182 L89X A419 2
## 183 M009 DIA 2
## 184 M069 DIA 2
## 185 M869 DIA 2
## 186 N049 DIA 2
## 187 N159 DIA 2
## 188 N182 DIA 2
## 189 R001 DIA 2
## 190 R098 DIA 2
## 191 R402 DIA 2
## 192 R450 DIA 2
## 193 R488 DIA 2
## 194 R568 DIA 2
## 195 R609 DIA 2
## 196 R64X DIA 2
## 197 S982 DIA 2
## 198 S984 DIA 2
## 199 A498 A419 1
## 200 A812 A419 1
## 201 B427 A419 1
## 202 B465 A419 1
## 203 B59X B210 1
## 204 C249 A419 1
## 205 C709 A419 1
## 206 C787 C629 1
## 207 C819 C80X 1
## 208 C857 A419 1
## 209 D376 A419 1
## 210 D609 A419 1
## 211 D65X A419 1
## 212 D693 A419 1
## 213 D823 A419 1
## 214 E160 DIA 1
## 215 E169 D649 1
## 216 E441 DIA 1
## 217 E509 DIA 1
## 218 E668 DIA 1
## 219 E752 DIA 1
## 220 E779 DIA 1
## 221 E785 DIA 1
## 222 E870 DIA 1
## 223 E873 A419 1
## 224 E888 DIA 1
## 225 F009 DIA 1
## 226 F067 DIA 1
## 227 F190 DIA 1
## 228 F203 DIA 1
## 229 F209 A419 1
## 230 F410 DIA 1
## 231 F88X DIA 1
## 232 G01X A398 1
## 233 G020 B24X 1
## 234 G038 B461 1
## 235 G062 DIA 1
## 236 G08X B461 1
## 237 G122 DIA 1
## 238 G20X DIA 1
## 239 G309 A86X 1
## 240 G360 DIA 1
## 241 G371 DIA 1
## 242 G419 DIA 1
## 243 G540 DIA 1
## 244 G588 DIA 1
## 245 G589 A419 1
## 246 G590 A419 1
## 247 G629 DIA 1
## 248 G732 A418 1
## 249 G819 A419 1
## 250 H280 DIA 1
## 251 H540 D539 1
## 252 H900 DIA 1
## 253 I10 DIA 1
## 254 I129 DIA 1
## 255 I130 DIA 1
## 256 I158 DIA 1
## 257 I229 DIA 1
## 258 I233 DIA 1
## 259 I248 DIA 1
## 260 I249 DIA 1
## 261 I252 DIA 1
## 262 I278 DIA 1
## 263 I313 A419 1
## 264 I319 DIA 1
## 265 I339 DIA 1
## 266 I420 DIA 1
## 267 I429 DIA 1
## 268 I443 DIA 1
## 269 I472 DIA 1
## 270 I519 DIA 1
## 271 I600 DIA 1
## 272 I601 DIA 1
## 273 I606 DIA 1
## 274 I607 DIA 1
## 275 I608 DIA 1
## 276 I615 DIA 1
## 277 I618 A419 1
## 278 I620 DIA 1
## 279 I629 DIA 1
## 280 I634 DIA 1
## 281 I635 DIA 1
## 282 I660 DIA 1
## 283 I670 DIA 1
## 284 I690 DIA 1
## 285 I691 DIA 1
## 286 I700 DIA 1
## 287 I748 DIA 1
## 288 I774 DIA 1
## 289 I775 A419 1
## 290 I828 C182 1
## 291 I829 D539 1
## 292 I830 DIA 1
## 293 I848 DIA 1
## 294 I850 D464 1
## 295 I859 DIA 1
## 296 I889 DIA 1
## 297 I99X DIA 1
## 298 J120 DIA 1
## 299 J152 DIA 1
## 300 J156 DIA 1
## 301 J157 A419 1
## 302 J160 DIA 1
## 303 J170 DIA 1
## 304 J178 DIA 1
## 305 J181 DIA 1
## 306 J208 D649 1
## 307 J209 DIA 1
## 308 J219 DIA 1
## 309 J386 DIA 1
## 310 J448 DIA 1
## 311 J853 A418 1
## 312 J930 DIA 1
## 313 J938 A150 1
## 314 J948 A419 1
## 315 J949 DIA 1
## 316 J950 DIA 1
## 317 J958 A415 1
## 318 J984 A560 1
## 319 K047 DIA 1
## 320 K113 DIA 1
## 321 K221 A86X 1
## 322 K250 DIA 1
## 323 K251 DIA 1
## 324 K259 DIA 1
## 325 K261 A418 1
## 326 K275 A419 1
## 327 K36X DIA 1
## 328 K404 DIA 1
## 329 K409 DIA 1
## 330 K419 A419 1
## 331 K529 DIA 1
## 332 K550 DIA 1
## 333 K559 A419 1
## 334 K562 A419 1
## 335 K564 DIA 1
## 336 K610 DIA 1
## 337 K658 A419 1
## 338 K701 DIA 1
## 339 K704 DIA 1
## 340 K711 DIA 1
## 341 K717 DIA 1
## 342 K745 DIA 1
## 343 K778 DIA 1
## 344 K802 A161 1
## 345 K803 A419 1
## 346 K805 DIA 1
## 347 K810 A419 1
## 348 K811 A419 1
## 349 K821 DIA 1
## 350 K823 DIA 1
## 351 K831 C259 1
## 352 K839 A419 1
## 353 K850 DIA 1
## 354 K858 DIA 1
## 355 K915 DIA 1
## 356 K920 B150 1
## 357 K928 DIA 1
## 358 L023 DIA 1
## 359 L024 A418 1
## 360 L032 B028 1
## 361 L080 A410 1
## 362 L088 DIA 1
## 363 L959 A419 1
## 364 L97X DIA 1
## 365 M329 DIA 1
## 366 M340 DIA 1
## 367 M600 DIA 1
## 368 M770 DIA 1
## 369 M844 DIA 1
## 370 M870 DIA 1
## 371 N050 A419 1
## 372 N059 A415 1
## 373 N070 DIA 1
## 374 N111 DIA 1
## 375 N119 A415 1
## 376 N139 DIA 1
## 377 N151 A419 1
## 378 N170 C541 1
## 379 N172 A419 1
## 380 N181 D649 1
## 381 N183 D649 1
## 382 N209 A419 1
## 383 N220 DIA 1
## 384 N23X A419 1
## 385 N40X DIA 1
## 386 N499 DIA 1
## 387 N641 A419 1
## 388 O009 DIA 1
## 389 P369 DIA 1
## 390 Q249 DIA 1
## 391 Q909 DIA 1
## 392 R072 DIA 1
## 393 R101 A419 1
## 394 R104 DIA 1
## 395 R13X C220 1
## 396 R278 D649 1
## 397 R34X DIA 1
## 398 R418 DIA 1
## 399 R509 DIA 1
## 400 R51X A419 1
## 401 R601 DIA 1
## 402 R634 A419 1
## 403 R651 A419 1
## 404 R652 DIA 1
## 405 R680 A403 1
## 406 R770 DIA 1
## 407 R900 DIA 1
## 408 R98X DIA 1
## 409 S029 DIA 1
## 410 S065 DIA 1
## 411 S066 DIA 1
## 412 S098 DIA 1
## 413 S144 DIA 1
## 414 S323 DIA 1
## 415 S324 DIA 1
## 416 S328 DIA 1
## 417 S423 DIA 1
## 418 S489 DIA 1
## 419 S721 DIA 1
## 420 S724 DIA 1
## 421 S729 A419 1
## 422 S911 A169 1
## 423 S913 D649 1
## 424 T214 A419 1
## 425 T302 DIA 1
## 426 T310 DIA 1
## 427 T814 DIA 1
## 428 T825 DIA 1
## 429 T876 DIA 1
## 430 U069 DIA 1
## 431 U202 A150 1
## 432 Y835 DIA 1

## After lockdown

## Diag1 Diag2 Frequency
## 1 U071 DIA 2858
## 2 I10X DIA 1646
## 3 J189 DIA 1202
## 4 J960 DIA 1070
## 5 N390 DIA 1060
## 6 B972 DIA 500
## 7 N189 DIA 426
## 8 J969 DIA 411
## 9 J128 DIA 279
## 10 D649 DIA 222
## 11 E669 DIA 213
## 12 A419 DIA 199
## 13 U072 DIA 153
## 14 L031 DIA 115
## 15 J80X DIA 99
## 16 I64X DIA 82
## 17 E039 DIA 76
## 18 K922 DIA 70
## 19 E162 DIA 69
## 20 D509 DIA 61
## 21 I500 DIA 61
## 22 I639 DIA 56
## 23 J159 DIA 55
## 24 N10X DIA 53
## 25 N185 DIA 53
## 26 R739 DIA 53
## 27 A150 DIA 51
## 28 K746 DIA 51
## 29 N40X DIA 51
## 30 I509 DIA 49
## 31 L039 DIA 46
## 32 J22X DIA 43
## 33 G934 DIA 41
## 34 N110 DIA 38
## 35 S984 DIA 38
## 36 I219 DIA 37
## 37 J157 DIA 37
## 38 N039 DIA 35
## 39 E86X DIA 34
## 40 K859 DIA 34
## 41 N179 DIA 34
## 42 E660 DIA 33
## 43 I678 DIA 33
## 44 K850 DIA 33
## 45 R104 DIA 32
## 46 S913 DIA 32
## 47 A090 DIA 31
## 48 I679 DIA 31
## 49 L97X DIA 31
## 50 A418 DIA 30
## 51 N12X DIA 30
## 52 K703 DIA 29
## 53 I619 DIA 28
## 54 I739 DIA 26
## 55 E668 DIA 25
## 56 J168 DIA 25
## 57 S889 DIA 25
## 58 J459 DIA 24
## 59 L023 DIA 24
## 60 L030 DIA 24
## 61 D500 DIA 23
## 62 L038 DIA 23
## 63 G409 DIA 22
## 64 K358 DIA 22
## 65 N19X DIA 22
## 66 K297 DIA 21
## 67 N181 DIA 20
## 68 E46X DIA 19
## 69 K802 DIA 19
## 70 L029 DIA 19
## 71 R568 DIA 19
## 72 I120 DIA 18
## 73 J90X DIA 18
## 74 A162 DIA 17
## 75 K610 DIA 17
## 76 K805 DIA 17
## 77 D539 DIA 16
## 78 J841 DIA 16
## 79 K590 DIA 16
## 80 M869 DIA 16
## 81 J13X DIA 15
## 82 J849 DIA 15
## 83 J961 DIA 15
## 84 M069 DIA 15
## 85 E872 DIA 14
## 86 F209 DIA 14
## 87 I480 DIA 14
## 88 J690 DIA 14
## 89 L089 DIA 14
## 90 N200 DIA 14
## 91 R509 DIA 14
## 92 D648 DIA 13
## 93 I159 DIA 13
## 94 O829 DIA 13
## 95 R11X DIA 13
## 96 R572 DIA 13
## 97 T136 DIA 13
## 98 E785 DIA 12
## 99 K800 DIA 12
## 100 M726 DIA 12
## 101 R042 DIA 12
## 102 E160 DIA 11
## 103 G819 DIA 11
## 104 I634 DIA 11
## 105 J120 DIA 11
## 106 J209 DIA 11
## 107 J47X DIA 11
## 108 L024 DIA 11
## 109 L028 DIA 11
## 110 N184 DIA 11
## 111 O149 DIA 11
## 112 R02X DIA 11
## 113 S819 DIA 11
## 114 E780 DIA 10
## 115 F419 DIA 10
## 116 G459 DIA 10
## 117 J150 DIA 10
## 118 J449 DIA 10
## 119 K750 DIA 10
## 120 K819 DIA 10
## 121 K830 DIA 10
## 122 L890 DIA 10
## 123 N151 DIA 10
## 124 O821 DIA 10
## 125 R579 DIA 10
## 126 E871 DIA 9
## 127 I489 DIA 9
## 128 I694 DIA 9
## 129 I872 DIA 9
## 130 J180 DIA 9
## 131 J188 DIA 9
## 132 K295 DIA 9
## 133 K801 DIA 9
## 134 K851 DIA 9
## 135 K85X DIA 9
## 136 L021 DIA 9
## 137 L984 DIA 9
## 138 N319 DIA 9
## 139 R101 DIA 9
## 140 S720 DIA 9
## 141 S911 DIA 9
## 142 S981 DIA 9
## 143 S982 DIA 9
## 144 I469 DIA 8
## 145 I693 DIA 8
## 146 J069 DIA 8
## 147 J158 DIA 8
## 148 J81X DIA 8
## 149 K566 DIA 8
## 150 K810 DIA 8
## 151 K811 DIA 8
## 152 L899 DIA 8
## 153 N209 DIA 8
## 154 N289 DIA 8
## 155 N498 DIA 8
## 156 O234 DIA 8
## 157 S069 DIA 8
## 158 S881 DIA 8
## 159 E782 DIA 7
## 160 E878 DIA 7
## 161 F329 DIA 7
## 162 I119 DIA 7
## 163 I150 DIA 7
## 164 I209 DIA 7
## 165 I269 DIA 7
## 166 I610 DIA 7
## 167 I635 DIA 7
## 168 I829 DIA 7
## 169 K291 DIA 7
## 170 K37X DIA 7
## 171 K429 DIA 7
## 172 K729 DIA 7
## 173 N182 DIA 7
## 174 O470 DIA 7
## 175 S912 DIA 7
## 176 E161 DIA 6
## 177 E781 DIA 6
## 178 G403 DIA 6
## 179 I259 DIA 6
## 180 I490 DIA 6
## 181 I630 DIA 6
## 182 I850 DIA 6
## 183 J852 DIA 6
## 184 K122 DIA 6
## 185 K259 DIA 6
## 186 K928 DIA 6
## 187 L032 DIA 6
## 188 N111 DIA 6
## 189 N172 DIA 6
## 190 N23X DIA 6
## 191 N739 DIA 6
## 192 N819 DIA 6
## 193 N939 DIA 6
## 194 O809 DIA 6
## 195 R18X DIA 6
## 196 R609 DIA 6
## 197 S681 DIA 6
## 198 S721 DIA 6
## 199 T793 DIA 6
## 200 D609 DIA 5
## 201 E059 DIA 5
## 202 E835 DIA 5
## 203 E870 DIA 5
## 204 E876 DIA 5
## 205 F200 DIA 5
## 206 G049 DIA 5
## 207 G610 DIA 5
## 208 G629 DIA 5
## 209 I200 DIA 5
## 210 I252 DIA 5
## 211 I499 DIA 5
## 212 I516 DIA 5
## 213 I698 DIA 5
## 214 I802 DIA 5
## 215 I830 DIA 5
## 216 J939 DIA 5
## 217 K564 DIA 5
## 218 K709 DIA 5
## 219 K717 DIA 5
## 220 L020 DIA 5
## 221 L033 DIA 5
## 222 L88X DIA 5
## 223 M725 DIA 5
## 224 N133 DIA 5
## 225 N178 DIA 5
## 226 N180 DIA 5
## 227 N811 DIA 5
## 228 O064 DIA 5
## 229 R100 DIA 5
## 230 R17X DIA 5
## 231 R31X DIA 5
## 232 R418 DIA 5
## 233 R570 DIA 5
## 234 S822 DIA 5
## 235 S880 DIA 5
## 236 A020 DIA 4
## 237 B353 DIA 4
## 238 E789 DIA 4
## 239 F011 DIA 4
## 240 F321 DIA 4
## 241 F411 DIA 4
## 242 F412 DIA 4
## 243 G20X DIA 4
## 244 G408 DIA 4
## 245 G590 DIA 4
## 246 G969 DIA 4
## 247 H360 DIA 4
## 248 I255 DIA 4
## 249 I48X DIA 4
## 250 I609 DIA 4
## 251 I611 DIA 4
## 252 I620 DIA 4
## 253 I859 DIA 4
## 254 J029 DIA 4
## 255 J100 DIA 4
## 256 J160 DIA 4
## 257 J398 DIA 4
## 258 J450 DIA 4
## 259 K279 DIA 4
## 260 K290 DIA 4
## 261 K858 DIA 4
## 262 L022 DIA 4
## 263 L309 DIA 4
## 264 L89 DIA 4
## 265 L89X DIA 4
## 266 M009 DIA 4
## 267 M329 DIA 4
## 268 N049 DIA 4
## 269 N139 DIA 4
## 270 N210 DIA 4
## 271 N499 DIA 4
## 272 N971 DIA 4
## 273 O034 DIA 4
## 274 O730 DIA 4
## 275 R030 DIA 4
## 276 R51X DIA 4
## 277 R571 DIA 4
## 278 R688 DIA 4
## 279 S065 DIA 4
## 280 S619 DIA 4
## 281 T009 DIA 4
## 282 T147 DIA 4
## 283 T824 DIA 4
## 284 W199 DIA 4
## 285 Z488 DIA 4
## 286 D410 DIA 3
## 287 D487 DIA 3
## 288 E038 DIA 3
## 289 E230 DIA 3
## 290 E43X DIA 3
## 291 E440 DIA 3
## 292 E784 DIA 3
## 293 E880 DIA 3
## 294 F03X DIA 3
## 295 F059 DIA 3
## 296 F102 DIA 3
## 297 G309 DIA 3
## 298 G442 DIA 3
## 299 H050 DIA 3
## 300 H813 DIA 3
## 301 H819 DIA 3
## 302 I060 DIA 3
## 303 I210 DIA 3
## 304 I249 DIA 3
## 305 I612 DIA 3
## 306 I618 DIA 3
## 307 I629 DIA 3
## 308 I771 DIA 3
## 309 I803 DIA 3
## 310 I809 DIA 3
## 311 I959 DIA 3
## 312 J019 DIA 3
## 313 J068 DIA 3
## 314 J152 DIA 3
## 315 J178 DIA 3
## 316 K439 DIA 3
## 317 K565 DIA 3
## 318 K591 DIA 3
## 319 K720 DIA 3
## 320 K929 DIA 3
## 321 L409 DIA 3
## 322 L509 DIA 3
## 323 M939 DIA 3
## 324 N083 DIA 3
## 325 N183 DIA 3
## 326 N398 DIA 3
## 327 N459 DIA 3
## 328 N61X DIA 3
## 329 N813 DIA 3
## 330 N930 DIA 3
## 331 O141 DIA 3
## 332 O342 DIA 3
## 333 O800 DIA 3
## 334 O990 DIA 3
## 335 R000 DIA 3
## 336 R190 DIA 3
## 337 R33X DIA 3
## 338 R53X DIA 3
## 339 R560 DIA 3
## 340 R601 DIA 3
## 341 R64X DIA 3
## 342 S729 DIA 3
## 343 S781 DIA 3
## 344 S828 DIA 3
## 345 S917 DIA 3
## 346 S980 DIA 3
## 347 T140 DIA 3
## 348 T141 DIA 3
## 349 T814 DIA 3
## 350 T874 DIA 3
## 351 Z896 DIA 3
## 352 A599 DIA 2
## 353 B028 DIA 2
## 354 D329 DIA 2
## 355 E030 DIA 2
## 356 E212 DIA 2
## 357 E874 DIA 2
## 358 F019 DIA 2
## 359 F250 DIA 2
## 360 F341 DIA 2
## 361 F410 DIA 2
## 362 F413 DIA 2
## 363 F445 DIA 2
## 364 F449 DIA 2
## 365 G041 DIA 2
## 366 G060 DIA 2
## 367 G311 DIA 2
## 368 G400 DIA 2
## 369 G404 DIA 2
## 370 G911 DIA 2
## 371 G932 DIA 2
## 372 G952 DIA 2
## 373 G959 DIA 2
## 374 H060 DIA 2
## 375 H400 DIA 2
## 376 H46X DIA 2
## 377 H811 DIA 2
## 378 H82X DIA 2
## 379 I251 DIA 2
## 380 I258 DIA 2
## 381 I309 DIA 2
## 382 I330 DIA 2
## 383 I350 DIA 2
## 384 I440 DIA 2
## 385 I600 DIA 2
## 386 I633 DIA 2
## 387 I638 DIA 2
## 388 I709 DIA 2
## 389 I742 DIA 2
## 390 I743 DIA 2
## 391 I821 DIA 2
## 392 I828 DIA 2
## 393 I868 DIA 2
## 394 I958 DIA 2
## 395 J123 DIA 2
## 396 J320 DIA 2
## 397 J850 DIA 2
## 398 J869 DIA 2
## 399 J91X DIA 2
## 400 J980 DIA 2
## 401 J981 DIA 2
## 402 J984 DIA 2
## 403 J989 DIA 2
## 404 K102 DIA 2
## 405 K219 DIA 2
## 406 K250 DIA 2
## 407 K260 DIA 2
## 408 K296 DIA 2
## 409 K30X DIA 2
## 410 K352 DIA 2
## 411 K359 DIA 2
## 412 K409 DIA 2
## 413 K420 DIA 2
## 414 K593 DIA 2
## 415 K630 DIA 2
## 416 K659 DIA 2
## 417 K661 DIA 2
## 418 K700 DIA 2
## 419 K710 DIA 2
## 420 K721 DIA 2
## 421 K768 DIA 2
## 422 K803 DIA 2
## 423 K839 DIA 2
## 424 K920 DIA 2
## 425 L088 DIA 2
## 426 L109 DIA 2
## 427 L209 DIA 2
## 428 L511 DIA 2
## 429 L600 DIA 2
## 430 L80X DIA 2
## 431 M059 DIA 2
## 432 M139 DIA 2
## 433 M169 DIA 2
## 434 M179 DIA 2
## 435 M199 DIA 2
## 436 M320 DIA 2
## 437 M490 DIA 2
## 438 M512 DIA 2
## 439 M545 DIA 2
## 440 M809 DIA 2
## 441 M860 DIA 2
## 442 M900 DIA 2
## 443 M930 DIA 2
## 444 N009 DIA 2
## 445 N071 DIA 2
## 446 N10 DIA 2
## 447 N119 DIA 2
## 448 N130 DIA 2
## 449 N131 DIA 2
## 450 N188 DIA 2
## 451 N300 DIA 2
## 452 N302 DIA 2
## 453 N310 DIA 2
## 454 N321 DIA 2
## 455 N393 DIA 2
## 456 N433 DIA 2
## 457 N481 DIA 2
## 458 N512 DIA 2
## 459 N63X DIA 2
## 460 N760 DIA 2
## 461 N764 DIA 2
## 462 N768 DIA 2
## 463 N771 DIA 2
## 464 N816 DIA 2
## 465 N818 DIA 2
## 466 N850 DIA 2
## 467 O140 DIA 2
## 468 O200 DIA 2
## 469 O211 DIA 2
## 470 O230 DIA 2
## 471 O40X DIA 2
## 472 O429 DIA 2
## 473 O758 DIA 2
## 474 Q181 DIA 2
## 475 R040 DIA 2
## 476 R063 DIA 2
## 477 R092 DIA 2
## 478 R160 DIA 2
## 479 R222 DIA 2
## 480 R229 DIA 2
## 481 R32X DIA 2
## 482 R410 DIA 2
## 483 R578 DIA 2
## 484 R651 DIA 2
## 485 R770 DIA 2
## 486 S010 DIA 2
## 487 S064 DIA 2
## 488 S099 DIA 2
## 489 S202 DIA 2
## 490 S328 DIA 2
## 491 S423 DIA 2
## 492 S682 DIA 2
## 493 S711 DIA 2
## 494 S789 DIA 2
## 495 S810 DIA 2
## 496 S818 DIA 2
## 497 S825 DIA 2
## 498 S826 DIA 2
## 499 S983 DIA 2
## 500 T600 DIA 2
## 501 T639 DIA 2
## 502 T856 DIA 2
## 503 Y835 DIA 2
## 504 Z359 DIA 2
## 505 A179 A159 1
## 506 A972 A270 1
## 507 B24 A154 1
## 508 B91X A180 1
## 509 C795 C349 1
## 510 C859 B24X 1
## 511 D057 B972 1
## 512 D379 C786 1
## 513 D692 A090 1
## 514 D70X B370 1
## 515 D728 D649 1
## 516 E034 DIA 1
## 517 E041 DIA 1
## 518 E049 DIA 1
## 519 E050 DIA 1
## 520 E063 DIA 1
## 521 E079 DIA 1
## 522 E169 DIA 1
## 523 E220 DIA 1
## 524 E242 DIA 1
## 525 E271 DIA 1
## 526 E272 DIA 1
## 527 E274 DIA 1
## 528 E279 DIA 1
## 529 E340 A150 1
## 530 E441 DIA 1
## 531 E512 DIA 1
## 532 E538 DIA 1
## 533 E60X DIA 1
## 534 E66 E11 1
## 535 E6691 B972 1
## 536 E673 DIA 1
## 537 E722 DIA 1
## 538 E750 DIA 1
## 539 E756 DIA 1
## 540 E778 DIA 1
## 541 E875 DIA 1
## 542 E889 DIA 1
## 543 F009 DIA 1
## 544 F010 DIA 1
## 545 F050 DIA 1
## 546 F051 DIA 1
## 547 F064 DIA 1
## 548 F067 D649 1
## 549 F100 DIA 1
## 550 F106 DIA 1
## 551 F172 DIA 1
## 552 F192 DIA 1
## 553 F239 DIA 1
## 554 F259 DIA 1
## 555 F29X DIA 1
## 556 F31 DIA 1
## 557 F312 DIA 1
## 558 F339 DIA 1
## 559 F418 DIA 1
## 560 F432 B972 1
## 561 F448 DIA 1
## 562 F459 A162 1
## 563 F481 DIA 1
## 564 F602 DIA 1
## 565 F719 DIA 1
## 566 F82X DIA 1
## 567 F909 DIA 1
## 568 F919 DIA 1
## 569 G009 DIA 1
## 570 G038 DIA 1
## 571 G039 DIA 1
## 572 G040 DIA 1
## 573 G042 DIA 1
## 574 G048 DIA 1
## 575 G08X DIA 1
## 576 G10X DIA 1
## 577 G255 DIA 1
## 578 G301 DIA 1
## 579 G310 DIA 1
## 580 G373 DIA 1
## 581 G402 DIA 1
## 582 G440 DIA 1
## 583 G443 DIA 1
## 584 G458 DIA 1
## 585 G510 DIA 1
## 586 G530 DIA 1
## 587 G589 A419 1
## 588 G619 DIA 1
## 589 G628 DIA 1
## 590 G632 DIA 1
## 591 G633 DIA 1
## 592 G936 DIA 1
## 593 G939 DIA 1
## 594 G958 DIA 1
## 595 G970 DIA 1
## 596 G98X DIA 1
## 597 G990 DIA 1
## 598 H041 DIA 1
## 599 H043 DIA 1
## 600 H113 DIA 1
## 601 H208 DIA 1
## 602 H218 DIA 1
## 603 H269 A090 1
## 604 H280 DIA 1
## 605 H441 DIA 1
## 606 H544 DIA 1
## 607 H588 B580 1
## 608 H659 DIA 1
## 609 H669 DIA 1
## 610 H920 DIA 1
## 611 I061 D509 1
## 612 I079 DIA 1
## 613 I130 DIA 1
## 614 I139 DIA 1
## 615 I152 DIA 1
## 616 I158 DIA 1
## 617 I212 DIA 1
## 618 I238 DIA 1
## 619 I248 DIA 1
## 620 I250 DIA 1
## 621 I256 B342 1
## 622 I392 B342 1
## 623 I400 DIA 1
## 624 I420 DIA 1
## 625 I429 D599 1
## 626 I442 DIA 1
## 627 I443 DIA 1
## 628 I447 DIA 1
## 629 I460 DIA 1
## 630 I461 A419 1
## 631 I479 DIA 1
## 632 I48 DIA 1
## 633 I481 DIA 1
## 634 I482 DIA 1
## 635 I495 DIA 1
## 636 I501 DIA 1
## 637 I517 DIA 1
## 638 I519 D649 1
## 639 I603 DIA 1
## 640 I607 DIA 1
## 641 I613 DIA 1
## 642 I615 DIA 1
## 643 I616 DIA 1
## 644 I631 DIA 1
## 645 I632 DIA 1
## 646 I652 DIA 1
## 647 I670 DIA 1
## 648 I671 DIA 1
## 649 I674 DIA 1
## 650 I688 DIA 1
## 651 I690 DIA 1
## 652 I691 DIA 1
## 653 I702 DIA 1
## 654 I748 B671 1
## 655 I749 DIA 1
## 656 I775 DIA 1
## 657 I779 DIA 1
## 658 I798 DIA 1
## 659 I800 DIA 1
## 660 I822 DIA 1
## 661 I839 DIA 1
## 662 I842 DIA 1
## 663 I861 DIA 1
## 664 I870 DIA 1
## 665 I871 C349 1
## 666 I879 DIA 1
## 667 I988 DIA 1
## 668 J040 DIA 1
## 669 J09X DIA 1
## 670 J15 A90 1
## 671 J154 DIA 1
## 672 J18 B972 1
## 673 J181 DIA 1
## 674 J22 B972 1
## 675 J40X DIA 1
## 676 J42 D500 1
## 677 J42X DIA 1
## 678 J440 DIA 1
## 679 J631 DIA 1
## 680 J634 D649 1
## 681 J638 DIA 1
## 682 J698 DIA 1
## 683 J80 B972 1
## 684 J848 D649 1
## 685 J851 DIA 1
## 686 J90 DIA 1
## 687 J929 DIA 1
## 688 J938 DIA 1
## 689 J940 DIA 1
## 690 J948 DIA 1
## 691 J950 C329 1
## 692 J955 B972 1
## 693 J96 A90 1
## 694 J982 DIA 1
## 695 J986 DIA 1
## 696 J988 DIA 1
## 697 J990 DIA 1
## 698 K029 DIA 1
## 699 K047 DIA 1
## 700 K109 DIA 1
## 701 K113 DIA 1
## 702 K115 DIA 1
## 703 K121 DIA 1
## 704 K20X DIA 1
## 705 K226 DIA 1
## 706 K254 DIA 1
## 707 K263 C248 1
## 708 K269 A419 1
## 709 K270 DIA 1
## 710 K273 DIA 1
## 711 K294 D648 1
## 712 K315 DIA 1
## 713 K317 D649 1
## 714 K318 DIA 1
## 715 K319 DIA 1
## 716 K400 DIA 1
## 717 K402 DIA 1
## 718 K430 DIA 1
## 719 K431 DIA 1
## 720 K440 C159 1
## 721 K469 DIA 1
## 722 K512 D500 1
## 723 K529 C780 1
## 724 K560 DIA 1
## 725 K562 DIA 1
## 726 K579 DIA 1
## 727 K611 DIA 1
## 728 K614 DIA 1
## 729 K623 DIA 1
## 730 K632 DIA 1
## 731 K650 DIA 1
## 732 K660 DIA 1
## 733 K732 D134 1
## 734 K739 DIA 1
## 735 K743 DIA 1
## 736 K744 DIA 1
## 737 K745 DIA 1
## 738 K752 DIA 1
## 739 K769 DIA 1
## 740 K804 DIA 1
## 741 K818 DIA 1
## 742 K822 DIA 1
## 743 K828 DIA 1
## 744 K829 DIA 1
## 745 K85 DIA 1
## 746 K863 DIA 1
## 747 K868 DIA 1
## 748 L00X DIA 1
## 749 L03 E11 1
## 750 L03X E11X 1
## 751 L043 DIA 1
## 752 L048 DIA 1
## 753 L120 DIA 1
## 754 L891 DIA 1
## 755 L932 DIA 1
## 756 L959 DIA 1
## 757 L97 DIA 1
## 758 M0699 DIA 1
## 759 M131 DIA 1
## 760 M142 DIA 1
## 761 M146 DIA 1
## 762 M170 DIA 1
## 763 M185 DIA 1
## 764 M191 DIA 1
## 765 M211 DIA 1
## 766 M235 DIA 1
## 767 M255 DIA 1
## 768 M321 DIA 1
## 769 M464 DIA 1
## 770 M478 A059 1
## 771 M480 DIA 1
## 772 M493 DIA 1
## 773 M511 DIA 1
## 774 M544 DIA 1
## 775 M549 DIA 1
## 776 M706 DIA 1
## 777 M719 DIA 1
## 778 M720 DIA 1
## 779 M728 DIA 1
## 780 M752 DIA 1
## 781 M753 DIA 1
## 782 M793 DIA 1
## 783 M795 DIA 1
## 784 M839 DIA 1
## 785 M841 DIA 1
## 786 M843 DIA 1
## 787 M866 DIA 1
## 788 M868 DIA 1
## 789 M870 B972 1
## 790 M878 DIA 1
## 791 M879 DIA 1
## 792 M952 DIA 1
## 793 N000 D509 1
## 794 N029 DIA 1
## 795 N040 DIA 1
## 796 N042 DIA 1
## 797 N059 DIA 1
## 798 N132 DIA 1
## 799 N150 D509 1
## 800 N158 D303 1
## 801 N165 DIA 1
## 802 N18 DIA 1
## 803 N18X E039 1
## 804 N19 D599 1
## 805 N202 DIA 1
## 806 N218 DIA 1
## 807 N219 DIA 1
## 808 N220 DIA 1
## 809 N309 DIA 1
## 810 N312 DIA 1
## 811 N322 DIA 1
## 812 N341 A010 1
## 813 N359 DIA 1
## 814 N391 DIA 1
## 815 N411 DIA 1
## 816 N482 C630 1
## 817 N489 DIA 1
## 818 N492 DIA 1
## 819 N709 DIA 1
## 820 N738 DIA 1
## 821 N751 DIA 1
## 822 N762 DIA 1
## 823 N81 B972 1
## 824 N810 DIA 1
## 825 N820 DIA 1
## 826 N841 DIA 1
## 827 N879 DIA 1
## 828 N911 B86X 1
## 829 N938 B342 1
## 830 N949 DIA 1
## 831 N994 DIA 1
## 832 O010 DIA 1
## 833 O020 DIA 1
## 834 O039 B342 1
## 835 O13X DIA 1
## 836 O210 DIA 1
## 837 O312 DIA 1
## 838 O335 DIA 1
## 839 O360 DIA 1
## 840 O364 DIA 1
## 841 O410 DIA 1
## 842 O710 DIA 1
## 843 O839 DIA 1
## 844 O840 B972 1
## 845 O860 DIA 1
## 846 O908 DIA 1
## 847 O912 DIA 1
## 848 Q602 DIA 1
## 849 Q603 DIA 1
## 850 Q649 DIA 1
## 851 Q811 DIA 1
## 852 R001 B972 1
## 853 R060 DIA 1
## 854 R071 DIA 1
## 855 R072 DIA 1
## 856 R074 DIA 1
## 857 R103 DIA 1
## 858 R162 DIA 1
## 859 R17 C249 1
## 860 R221 DIA 1
## 861 R398 DIA 1
## 862 R42X DIA 1
## 863 R520 DIA 1
## 864 R55X DIA 1
## 865 R600 DIA 1
## 866 R632 DIA 1
## 867 R634 DIA 1
## 868 R798 DIA 1
## 869 R826 D508 1
## 870 R829 B972 1
## 871 R931 DIA 1
## 872 S056 DIA 1
## 873 S066 DIA 1
## 874 S06X E10X 1
## 875 S071 DIA 1
## 876 S119 DIA 1
## 877 S208 DIA 1
## 878 S222 B001 1
## 879 S223 DIA 1
## 880 S270 DIA 1
## 881 S301 DIA 1
## 882 S311 DIA 1
## 883 S318 DIA 1
## 884 S320 DIA 1
## 885 S369 DIA 1
## 886 S370 DIA 1
## 887 S373 A419 1
## 888 S399 DIA 1
## 889 S400 DIA 1
## 890 S411 DIA 1
## 891 S430 DIA 1
## 892 S498 DIA 1
## 893 S510 D649 1
## 894 S520 DIA 1
## 895 S580 DIA 1
## 896 S589 DIA 1
## 897 S610 DIA 1
## 898 S618 DIA 1
## 899 S668 DIA 1
## 900 S683 DIA 1
## 901 S728 DIA 1
## 902 S800 DIA 1
## 903 S801 DIA 1
## 904 S817 DIA 1
## 905 S820 DIA 1
## 906 S824 DIA 1
## 907 S878 DIA 1
## 908 S908 DIA 1
## 909 S909 DIA 1
## 910 S922 DIA 1
## 911 S934 DIA 1
## 912 S960 DIA 1
## 913 T013 DIA 1
## 914 T019 DIA 1
## 915 T054 DIA 1
## 916 T059 DIA 1
## 917 T07X DIA 1
## 918 T08X B972 1
## 919 T131 DIA 1
## 920 T139 DIA 1
## 921 T142 DIA 1
## 922 T149 DIA 1
## 923 T212 DIA 1
## 924 T241 DIA 1
## 925 T250 D649 1
## 926 T302 DIA 1
## 927 T303 DIA 1
## 928 T409 DIA 1
## 929 T477 DIA 1
## 930 T652 DIA 1
## 931 T802 DIA 1
## 932 T818 DIA 1
## 933 T827 DIA 1
## 934 T860 DIA 1
## 935 T876 DIA 1
## 936 T887 DIA 1
## 937 T888 A150 1
## 938 T889 DIA 1
## 939 T983 DIA 1
## 940 W170 DIA 1
## 941 W180 DIA 1
## 942 Y832 A419 1
## 943 Y919 DIA 1
## 944 Z038 DIA 1
## 945 Z100 DIA 1
## 946 Z208 DIA 1
## 947 Z392 DIA 1
## 948 Z549 DIA 1
## 949 Z639 DIA 1
## 950 Z721 DIA 1
## 951 Z988 DIA 1

### Alive patients, after lockdown

## Diag1 Diag2 Frequency
## 1 U071 DIA 2226
## 2 I10X DIA 1551
## 3 N390 DIA 1036
## 4 J189 DIA 832
## 5 J960 DIA 662
## 6 B972 DIA 433
## 7 N189 DIA 390
## 8 J128 DIA 227
## 9 J969 DIA 221
## 10 D649 DIA 212
## 11 E669 DIA 196
## 12 A419 DIA 117
## 13 L031 DIA 115
## 14 U072 DIA 110
## 15 E039 DIA 70
## 16 I64X DIA 69
## 17 E162 DIA 67
## 18 K922 DIA 60
## 19 D509 DIA 58
## 20 I500 DIA 54
## 21 R739 DIA 53
## 22 I639 DIA 51
## 23 N10X DIA 51
## 24 N40X DIA 51
## 25 I509 DIA 48
## 26 K746 DIA 46
## 27 N185 DIA 46
## 28 J159 DIA 45
## 29 L039 DIA 45
## 30 A150 DIA 43
## 31 J80X DIA 40
## 32 N110 DIA 38
## 33 S984 DIA 38
## 34 J22X DIA 35
## 35 N039 DIA 33
## 36 E660 DIA 32
## 37 K850 DIA 32
## 38 K859 DIA 32
## 39 R104 DIA 32
## 40 A090 DIA 31
## 41 L97X DIA 31
## 42 S913 DIA 31
## 43 I678 DIA 30
## 44 N12X DIA 30
## 45 E86X DIA 28
## 46 G934 DIA 28
## 47 J157 DIA 28
## 48 K703 DIA 28
## 49 I679 DIA 26
## 50 E668 DIA 25
## 51 I739 DIA 25
## 52 N179 DIA 25
## 53 S889 DIA 25
## 54 I219 DIA 24
## 55 I619 DIA 24
## 56 L023 DIA 24
## 57 L030 DIA 24
## 58 L038 DIA 23
## 59 D500 DIA 22
## 60 G409 DIA 22
## 61 J459 DIA 22
## 62 K358 DIA 22
## 63 J168 DIA 21
## 64 K297 DIA 21
## 65 N181 DIA 20
## 66 N19X DIA 20
## 67 E46X DIA 19
## 68 K802 DIA 19
## 69 L029 DIA 19
## 70 J90X DIA 17
## 71 K805 DIA 17
## 72 R568 DIA 17
## 73 A162 DIA 16
## 74 K590 DIA 16
## 75 K610 DIA 16
## 76 M869 DIA 16
## 77 D539 DIA 14
## 78 J849 DIA 14
## 79 N200 DIA 14
## 80 R509 DIA 14
## 81 F209 DIA 13
## 82 I480 DIA 13
## 83 J13X DIA 13
## 84 O829 DIA 13
## 85 R11X DIA 13
## 86 A418 DIA 12
## 87 D648 DIA 12
## 88 E785 DIA 12
## 89 I120 DIA 12
## 90 I159 DIA 12
## 91 J841 DIA 12
## 92 J961 DIA 12
## 93 K800 DIA 12
## 94 L089 DIA 12
## 95 M069 DIA 12
## 96 E160 DIA 11
## 97 G819 DIA 11
## 98 J120 DIA 11
## 99 J209 DIA 11
## 100 J47X DIA 11
## 101 L024 DIA 11
## 102 L028 DIA 11
## 103 O149 DIA 11
## 104 R042 DIA 11
## 105 S819 DIA 11
## 106 E780 DIA 10
## 107 F419 DIA 10
## 108 G459 DIA 10
## 109 J449 DIA 10
## 110 K819 DIA 10
## 111 K830 DIA 10
## 112 L890 DIA 10
## 113 M726 DIA 10
## 114 O821 DIA 10
## 115 R02X DIA 10
## 116 E872 DIA 9
## 117 I634 DIA 9
## 118 I872 DIA 9
## 119 J188 DIA 9
## 120 J690 DIA 9
## 121 K295 DIA 9
## 122 K750 DIA 9
## 123 K801 DIA 9
## 124 K85X DIA 9
## 125 L021 DIA 9
## 126 L984 DIA 9
## 127 N151 DIA 9
## 128 N184 DIA 9
## 129 N319 DIA 9
## 130 R101 DIA 9
## 131 S720 DIA 9
## 132 S911 DIA 9
## 133 S981 DIA 9
## 134 S982 DIA 9
## 135 T136 DIA 9
## 136 I693 DIA 8
## 137 I694 DIA 8
## 138 J069 DIA 8
## 139 J150 DIA 8
## 140 K810 DIA 8
## 141 K811 DIA 8
## 142 K851 DIA 8
## 143 N209 DIA 8
## 144 O234 DIA 8
## 145 S069 DIA 8
## 146 S881 DIA 8
## 147 E782 DIA 7
## 148 E871 DIA 7
## 149 F329 DIA 7
## 150 I150 DIA 7
## 151 I209 DIA 7
## 152 I489 DIA 7
## 153 I610 DIA 7
## 154 I829 DIA 7
## 155 J180 DIA 7
## 156 K291 DIA 7
## 157 K37X DIA 7
## 158 K429 DIA 7
## 159 L899 DIA 7
## 160 N182 DIA 7
## 161 N289 DIA 7
## 162 N498 DIA 7
## 163 O470 DIA 7
## 164 S912 DIA 7
## 165 E161 DIA 6
## 166 E781 DIA 6
## 167 E878 DIA 6
## 168 G403 DIA 6
## 169 I119 DIA 6
## 170 I630 DIA 6
## 171 I635 DIA 6
## 172 I850 DIA 6
## 173 J158 DIA 6
## 174 J852 DIA 6
## 175 K259 DIA 6
## 176 K566 DIA 6
## 177 K729 DIA 6
## 178 K928 DIA 6
## 179 L032 DIA 6
## 180 N111 DIA 6
## 181 N23X DIA 6
## 182 N739 DIA 6
## 183 N819 DIA 6
## 184 N939 DIA 6
## 185 O809 DIA 6
## 186 R18X DIA 6
## 187 R609 DIA 6
## 188 S681 DIA 6
## 189 S721 DIA 6
## 190 T793 DIA 6
## 191 D609 DIA 5
## 192 E059 DIA 5
## 193 E835 DIA 5
## 194 E870 DIA 5
## 195 E876 DIA 5
## 196 F200 DIA 5
## 197 G049 DIA 5
## 198 G610 DIA 5
## 199 G629 DIA 5
## 200 I200 DIA 5
## 201 I499 DIA 5
## 202 I516 DIA 5
## 203 I698 DIA 5
## 204 I802 DIA 5
## 205 J81X DIA 5
## 206 K122 DIA 5
## 207 K564 DIA 5
## 208 K709 DIA 5
## 209 K717 DIA 5
## 210 L020 DIA 5
## 211 L033 DIA 5
## 212 L88X DIA 5
## 213 M725 DIA 5
## 214 N133 DIA 5
## 215 N180 DIA 5
## 216 N811 DIA 5
## 217 O064 DIA 5
## 218 R17X DIA 5
## 219 R31X DIA 5
## 220 R418 DIA 5
## 221 S822 DIA 5
## 222 A020 DIA 4
## 223 B353 DIA 4
## 224 E789 DIA 4
## 225 F011 DIA 4
## 226 F321 DIA 4
## 227 F411 DIA 4
## 228 F412 DIA 4
## 229 G20X DIA 4
## 230 G408 DIA 4
## 231 G590 DIA 4
## 232 H360 DIA 4
## 233 I259 DIA 4
## 234 I490 DIA 4
## 235 I611 DIA 4
## 236 I620 DIA 4
## 237 I830 DIA 4
## 238 I859 DIA 4
## 239 J029 DIA 4
## 240 J100 DIA 4
## 241 J398 DIA 4
## 242 J450 DIA 4
## 243 K279 DIA 4
## 244 K290 DIA 4
## 245 K858 DIA 4
## 246 L022 DIA 4
## 247 L309 DIA 4
## 248 L89 DIA 4
## 249 M009 DIA 4
## 250 M329 DIA 4
## 251 N049 DIA 4
## 252 N139 DIA 4
## 253 N172 DIA 4
## 254 N210 DIA 4
## 255 N971 DIA 4
## 256 O034 DIA 4
## 257 O730 DIA 4
## 258 R030 DIA 4
## 259 R100 DIA 4
## 260 R51X DIA 4
## 261 S619 DIA 4
## 262 T009 DIA 4
## 263 T147 DIA 4
## 264 T824 DIA 4
## 265 W199 DIA 4
## 266 Z488 DIA 4
## 267 D410 DIA 3
## 268 D487 DIA 3
## 269 E038 DIA 3
## 270 E230 DIA 3
## 271 E43X DIA 3
## 272 E784 DIA 3
## 273 E880 DIA 3
## 274 F03X DIA 3
## 275 F059 DIA 3
## 276 F102 DIA 3
## 277 G309 DIA 3
## 278 G442 DIA 3
## 279 G969 DIA 3
## 280 H050 DIA 3
## 281 H813 DIA 3
## 282 H819 DIA 3
## 283 I060 DIA 3
## 284 I255 DIA 3
## 285 I48X DIA 3
## 286 I609 DIA 3
## 287 I618 DIA 3
## 288 I629 DIA 3
## 289 I771 DIA 3
## 290 I809 DIA 3
## 291 J019 DIA 3
## 292 J152 DIA 3
## 293 J160 DIA 3
## 294 J178 DIA 3
## 295 J939 DIA 3
## 296 K439 DIA 3
## 297 K591 DIA 3
## 298 K720 DIA 3
## 299 K929 DIA 3
## 300 L409 DIA 3
## 301 L509 DIA 3
## 302 L89X DIA 3
## 303 M939 DIA 3
## 304 N083 DIA 3
## 305 N178 DIA 3
## 306 N183 DIA 3
## 307 N398 DIA 3
## 308 N459 DIA 3
## 309 N499 DIA 3
## 310 N61X DIA 3
## 311 N813 DIA 3
## 312 N930 DIA 3
## 313 O141 DIA 3
## 314 O342 DIA 3
## 315 O800 DIA 3
## 316 O990 DIA 3
## 317 R000 DIA 3
## 318 R190 DIA 3
## 319 R33X DIA 3
## 320 R53X DIA 3
## 321 R560 DIA 3
## 322 R601 DIA 3
## 323 R64X DIA 3
## 324 S065 DIA 3
## 325 S729 DIA 3
## 326 S781 DIA 3
## 327 S828 DIA 3
## 328 S880 DIA 3
## 329 S917 DIA 3
## 330 S980 DIA 3
## 331 T140 DIA 3
## 332 T141 DIA 3
## 333 T814 DIA 3
## 334 Z896 DIA 3
## 335 A599 DIA 2
## 336 B028 DIA 2
## 337 D329 DIA 2
## 338 E030 DIA 2
## 339 E212 DIA 2
## 340 E440 DIA 2
## 341 F019 DIA 2
## 342 F250 DIA 2
## 343 F341 DIA 2
## 344 F410 DIA 2
## 345 F445 DIA 2
## 346 F449 DIA 2
## 347 G041 DIA 2
## 348 G060 DIA 2
## 349 G311 DIA 2
## 350 G400 DIA 2
## 351 G404 DIA 2
## 352 G911 DIA 2
## 353 G932 DIA 2
## 354 G959 DIA 2
## 355 H060 DIA 2
## 356 H400 DIA 2
## 357 H46X DIA 2
## 358 H811 DIA 2
## 359 H82X DIA 2
## 360 I210 DIA 2
## 361 I249 DIA 2
## 362 I258 DIA 2
## 363 I269 DIA 2
## 364 I309 DIA 2
## 365 I350 DIA 2
## 366 I440 DIA 2
## 367 I600 DIA 2
## 368 I612 DIA 2
## 369 I633 DIA 2
## 370 I638 DIA 2
## 371 I709 DIA 2
## 372 I742 DIA 2
## 373 I743 DIA 2
## 374 I803 DIA 2
## 375 I821 DIA 2
## 376 I828 DIA 2
## 377 I868 DIA 2
## 378 J068 B342 2
## 379 J123 DIA 2
## 380 J91X DIA 2
## 381 J980 DIA 2
## 382 J981 DIA 2
## 383 J984 DIA 2
## 384 K102 DIA 2
## 385 K219 DIA 2
## 386 K250 DIA 2
## 387 K260 DIA 2
## 388 K296 DIA 2
## 389 K30X DIA 2
## 390 K352 DIA 2
## 391 K359 DIA 2
## 392 K409 DIA 2
## 393 K420 DIA 2
## 394 K565 DIA 2
## 395 K593 DIA 2
## 396 K630 DIA 2
## 397 K659 DIA 2
## 398 K661 DIA 2
## 399 K700 DIA 2
## 400 K710 DIA 2
## 401 K721 DIA 2
## 402 K768 DIA 2
## 403 K803 DIA 2
## 404 K839 DIA 2
## 405 L109 DIA 2
## 406 L209 DIA 2
## 407 L511 DIA 2
## 408 L600 DIA 2
## 409 L80X DIA 2
## 410 M059 DIA 2
## 411 M139 DIA 2
## 412 M169 DIA 2
## 413 M179 DIA 2
## 414 M199 DIA 2
## 415 M320 DIA 2
## 416 M490 DIA 2
## 417 M512 DIA 2
## 418 M545 DIA 2
## 419 M809 DIA 2
## 420 M860 DIA 2
## 421 M900 DIA 2
## 422 M930 DIA 2
## 423 N009 DIA 2
## 424 N071 DIA 2
## 425 N10 DIA 2
## 426 N119 DIA 2
## 427 N130 DIA 2
## 428 N131 DIA 2
## 429 N188 DIA 2
## 430 N300 DIA 2
## 431 N302 DIA 2
## 432 N310 DIA 2
## 433 N321 DIA 2
## 434 N393 DIA 2
## 435 N433 DIA 2
## 436 N481 DIA 2
## 437 N512 DIA 2
## 438 N63X DIA 2
## 439 N760 DIA 2
## 440 N768 DIA 2
## 441 N771 DIA 2
## 442 N816 DIA 2
## 443 N818 DIA 2
## 444 N850 DIA 2
## 445 O140 DIA 2
## 446 O200 DIA 2
## 447 O211 DIA 2
## 448 O230 DIA 2
## 449 O40X DIA 2
## 450 O429 DIA 2
## 451 O758 DIA 2
## 452 Q181 DIA 2
## 453 R040 DIA 2
## 454 R160 DIA 2
## 455 R222 DIA 2
## 456 R229 DIA 2
## 457 R32X DIA 2
## 458 R571 DIA 2
## 459 R572 DIA 2
## 460 R770 DIA 2
## 461 S010 DIA 2
## 462 S064 DIA 2
## 463 S099 DIA 2
## 464 S202 DIA 2
## 465 S328 DIA 2
## 466 S423 DIA 2
## 467 S682 DIA 2
## 468 S711 DIA 2
## 469 S789 DIA 2
## 470 S810 DIA 2
## 471 S818 DIA 2
## 472 S825 DIA 2
## 473 S826 DIA 2
## 474 S983 DIA 2
## 475 T600 DIA 2
## 476 T639 DIA 2
## 477 T856 DIA 2
## 478 T874 DIA 2
## 479 Y835 DIA 2
## 480 Z359 DIA 2
## 481 A179 A159 1
## 482 A972 A270 1
## 483 B24 A154 1
## 484 B91X A180 1
## 485 C795 C349 1
## 486 C859 B24X 1
## 487 D057 B972 1
## 488 D692 A090 1
## 489 D70X B370 1
## 490 D728 D649 1
## 491 E034 DIA 1
## 492 E041 DIA 1
## 493 E049 DIA 1
## 494 E050 DIA 1
## 495 E063 DIA 1
## 496 E079 DIA 1
## 497 E169 DIA 1
## 498 E220 DIA 1
## 499 E242 DIA 1
## 500 E271 DIA 1
## 501 E272 DIA 1
## 502 E274 DIA 1
## 503 E279 DIA 1
## 504 E340 A150 1
## 505 E441 DIA 1
## 506 E512 DIA 1
## 507 E538 DIA 1
## 508 E60X DIA 1
## 509 E66 E11 1
## 510 E6691 B972 1
## 511 E673 DIA 1
## 512 E722 DIA 1
## 513 E750 DIA 1
## 514 E756 DIA 1
## 515 E778 DIA 1
## 516 E889 DIA 1
## 517 F009 DIA 1
## 518 F010 DIA 1
## 519 F050 DIA 1
## 520 F051 DIA 1
## 521 F064 DIA 1
## 522 F067 D649 1
## 523 F106 DIA 1
## 524 F172 DIA 1
## 525 F192 DIA 1
## 526 F239 DIA 1
## 527 F259 DIA 1
## 528 F29X DIA 1
## 529 F31 DIA 1
## 530 F312 DIA 1
## 531 F339 DIA 1
## 532 F413 DIA 1
## 533 F418 DIA 1
## 534 F432 B972 1
## 535 F448 DIA 1
## 536 F459 A162 1
## 537 F481 DIA 1
## 538 F602 DIA 1
## 539 F719 DIA 1
## 540 F82X DIA 1
## 541 F909 DIA 1
## 542 F919 DIA 1
## 543 G009 DIA 1
## 544 G038 DIA 1
## 545 G039 DIA 1
## 546 G040 DIA 1
## 547 G042 DIA 1
## 548 G048 DIA 1
## 549 G08X DIA 1
## 550 G10X DIA 1
## 551 G255 DIA 1
## 552 G301 DIA 1
## 553 G310 DIA 1
## 554 G373 DIA 1
## 555 G402 DIA 1
## 556 G440 DIA 1
## 557 G443 DIA 1
## 558 G458 DIA 1
## 559 G510 DIA 1
## 560 G530 DIA 1
## 561 G589 A419 1
## 562 G619 DIA 1
## 563 G628 DIA 1
## 564 G632 DIA 1
## 565 G633 DIA 1
## 566 G936 DIA 1
## 567 G939 DIA 1
## 568 G952 C720 1
## 569 G958 DIA 1
## 570 G970 DIA 1
## 571 G98X DIA 1
## 572 G990 DIA 1
## 573 H041 DIA 1
## 574 H043 DIA 1
## 575 H113 DIA 1
## 576 H208 DIA 1
## 577 H218 DIA 1
## 578 H269 A090 1
## 579 H280 DIA 1
## 580 H441 DIA 1
## 581 H544 DIA 1
## 582 H588 B580 1
## 583 H659 DIA 1
## 584 H669 DIA 1
## 585 H920 DIA 1
## 586 I061 D509 1
## 587 I079 DIA 1
## 588 I139 DIA 1
## 589 I152 DIA 1
## 590 I158 DIA 1
## 591 I212 DIA 1
## 592 I248 DIA 1
## 593 I250 DIA 1
## 594 I251 DIA 1
## 595 I252 DIA 1
## 596 I256 B342 1
## 597 I330 DIA 1
## 598 I392 B342 1
## 599 I400 DIA 1
## 600 I420 DIA 1
## 601 I442 DIA 1
## 602 I443 DIA 1
## 603 I447 DIA 1
## 604 I460 DIA 1
## 605 I469 DIA 1
## 606 I479 DIA 1
## 607 I48 DIA 1
## 608 I481 DIA 1
## 609 I482 DIA 1
## 610 I495 DIA 1
## 611 I501 DIA 1
## 612 I517 DIA 1
## 613 I519 D649 1
## 614 I603 DIA 1
## 615 I613 DIA 1
## 616 I615 DIA 1
## 617 I616 DIA 1
## 618 I631 DIA 1
## 619 I632 DIA 1
## 620 I652 DIA 1
## 621 I671 DIA 1
## 622 I674 DIA 1
## 623 I688 DIA 1
## 624 I690 DIA 1
## 625 I691 DIA 1
## 626 I702 DIA 1
## 627 I748 B671 1
## 628 I749 DIA 1
## 629 I775 DIA 1
## 630 I779 DIA 1
## 631 I798 DIA 1
## 632 I800 DIA 1
## 633 I839 DIA 1
## 634 I842 DIA 1
## 635 I861 DIA 1
## 636 I870 DIA 1
## 637 I871 C349 1
## 638 I879 DIA 1
## 639 I988 DIA 1
## 640 J040 DIA 1
## 641 J15 A90 1
## 642 J154 DIA 1
## 643 J18 B972 1
## 644 J181 DIA 1
## 645 J22 B972 1
## 646 J40X DIA 1
## 647 J42 D500 1
## 648 J42X DIA 1
## 649 J440 DIA 1
## 650 J631 DIA 1
## 651 J634 D649 1
## 652 J698 DIA 1
## 653 J80 B972 1
## 654 J848 D649 1
## 655 J850 A160 1
## 656 J851 DIA 1
## 657 J869 DIA 1
## 658 J90 DIA 1
## 659 J938 DIA 1
## 660 J940 DIA 1
## 661 J948 DIA 1
## 662 J950 C329 1
## 663 J955 B972 1
## 664 J96 A90 1
## 665 J982 DIA 1
## 666 J988 DIA 1
## 667 J989 B972 1
## 668 K029 DIA 1
## 669 K047 DIA 1
## 670 K109 DIA 1
## 671 K113 DIA 1
## 672 K115 DIA 1
## 673 K121 DIA 1
## 674 K20X DIA 1
## 675 K226 DIA 1
## 676 K254 DIA 1
## 677 K263 C248 1
## 678 K270 DIA 1
## 679 K273 DIA 1
## 680 K294 D648 1
## 681 K315 DIA 1
## 682 K317 D649 1
## 683 K318 DIA 1
## 684 K319 DIA 1
## 685 K400 DIA 1
## 686 K402 DIA 1
## 687 K430 DIA 1
## 688 K440 C159 1
## 689 K469 DIA 1
## 690 K512 D500 1
## 691 K529 C780 1
## 692 K560 DIA 1
## 693 K562 DIA 1
## 694 K579 DIA 1
## 695 K611 DIA 1
## 696 K614 DIA 1
## 697 K623 DIA 1
## 698 K632 DIA 1
## 699 K650 DIA 1
## 700 K660 DIA 1
## 701 K732 D134 1
## 702 K739 DIA 1
## 703 K743 DIA 1
## 704 K744 DIA 1
## 705 K745 DIA 1
## 706 K752 DIA 1
## 707 K769 DIA 1
## 708 K804 DIA 1
## 709 K818 DIA 1
## 710 K822 DIA 1
## 711 K828 DIA 1
## 712 K829 DIA 1
## 713 K85 DIA 1
## 714 K863 DIA 1
## 715 K868 DIA 1
## 716 K920 DIA 1
## 717 L00X DIA 1
## 718 L03 E11 1
## 719 L03X E11X 1
## 720 L043 DIA 1
## 721 L048 DIA 1
## 722 L088 D538 1
## 723 L120 DIA 1
## 724 L891 DIA 1
## 725 L932 DIA 1
## 726 L97 DIA 1
## 727 M0699 DIA 1
## 728 M131 DIA 1
## 729 M142 DIA 1
## 730 M146 DIA 1
## 731 M170 DIA 1
## 732 M185 DIA 1
## 733 M191 DIA 1
## 734 M211 DIA 1
## 735 M235 DIA 1
## 736 M255 DIA 1
## 737 M321 DIA 1
## 738 M464 DIA 1
## 739 M478 A059 1
## 740 M480 DIA 1
## 741 M493 DIA 1
## 742 M511 DIA 1
## 743 M544 DIA 1
## 744 M549 DIA 1
## 745 M706 DIA 1
## 746 M719 DIA 1
## 747 M720 DIA 1
## 748 M728 DIA 1
## 749 M752 DIA 1
## 750 M753 DIA 1
## 751 M793 DIA 1
## 752 M795 DIA 1
## 753 M839 DIA 1
## 754 M841 DIA 1
## 755 M843 DIA 1
## 756 M866 DIA 1
## 757 M868 DIA 1
## 758 M870 B972 1
## 759 M878 DIA 1
## 760 M879 DIA 1
## 761 M952 DIA 1
## 762 N000 D509 1
## 763 N029 DIA 1
## 764 N040 DIA 1
## 765 N042 DIA 1
## 766 N059 DIA 1
## 767 N132 DIA 1
## 768 N150 D509 1
## 769 N158 D303 1
## 770 N18 DIA 1
## 771 N18X E039 1
## 772 N19 D599 1
## 773 N202 DIA 1
## 774 N218 DIA 1
## 775 N219 DIA 1
## 776 N220 DIA 1
## 777 N309 DIA 1
## 778 N312 DIA 1
## 779 N322 DIA 1
## 780 N341 A010 1
## 781 N359 DIA 1
## 782 N391 DIA 1
## 783 N411 DIA 1
## 784 N482 C630 1
## 785 N489 DIA 1
## 786 N492 DIA 1
## 787 N709 DIA 1
## 788 N738 DIA 1
## 789 N751 DIA 1
## 790 N762 DIA 1
## 791 N764 DIA 1
## 792 N81 B972 1
## 793 N810 DIA 1
## 794 N820 DIA 1
## 795 N841 DIA 1
## 796 N879 DIA 1
## 797 N911 B86X 1
## 798 N938 B342 1
## 799 N949 DIA 1
## 800 N994 DIA 1
## 801 O010 DIA 1
## 802 O020 DIA 1
## 803 O039 B342 1
## 804 O13X DIA 1
## 805 O210 DIA 1
## 806 O312 DIA 1
## 807 O335 DIA 1
## 808 O360 DIA 1
## 809 O364 DIA 1
## 810 O410 DIA 1
## 811 O710 DIA 1
## 812 O839 DIA 1
## 813 O840 B972 1
## 814 O860 DIA 1
## 815 O908 DIA 1
## 816 O912 DIA 1
## 817 Q602 DIA 1
## 818 Q603 DIA 1
## 819 Q649 DIA 1
## 820 Q811 DIA 1
## 821 R001 B972 1
## 822 R060 DIA 1
## 823 R063 DIA 1
## 824 R071 DIA 1
## 825 R072 DIA 1
## 826 R074 DIA 1
## 827 R092 DIA 1
## 828 R103 DIA 1
## 829 R162 DIA 1
## 830 R17 C249 1
## 831 R221 DIA 1
## 832 R42X DIA 1
## 833 R520 DIA 1
## 834 R55X DIA 1
## 835 R570 DIA 1
## 836 R578 DIA 1
## 837 R579 A499 1
## 838 R600 DIA 1
## 839 R632 DIA 1
## 840 R634 DIA 1
## 841 R798 DIA 1
## 842 R826 D508 1
## 843 R829 B972 1
## 844 S056 DIA 1
## 845 S066 DIA 1
## 846 S06X E10X 1
## 847 S071 DIA 1
## 848 S119 DIA 1
## 849 S208 DIA 1
## 850 S222 B001 1
## 851 S223 DIA 1
## 852 S270 DIA 1
## 853 S301 DIA 1
## 854 S311 DIA 1
## 855 S318 DIA 1
## 856 S320 DIA 1
## 857 S369 DIA 1
## 858 S370 DIA 1
## 859 S373 A419 1
## 860 S399 DIA 1
## 861 S400 DIA 1
## 862 S411 DIA 1
## 863 S430 DIA 1
## 864 S498 DIA 1
## 865 S510 D649 1
## 866 S520 DIA 1
## 867 S580 DIA 1
## 868 S589 DIA 1
## 869 S610 DIA 1
## 870 S618 DIA 1
## 871 S668 DIA 1
## 872 S683 DIA 1
## 873 S728 DIA 1
## 874 S800 DIA 1
## 875 S801 DIA 1
## 876 S817 DIA 1
## 877 S820 DIA 1
## 878 S824 DIA 1
## 879 S878 DIA 1
## 880 S908 DIA 1
## 881 S909 DIA 1
## 882 S922 DIA 1
## 883 S934 DIA 1
## 884 S960 DIA 1
## 885 T013 DIA 1
## 886 T019 DIA 1
## 887 T054 DIA 1
## 888 T059 DIA 1
## 889 T07X DIA 1
## 890 T08X B972 1
## 891 T131 DIA 1
## 892 T139 DIA 1
## 893 T142 DIA 1
## 894 T149 DIA 1
## 895 T212 DIA 1
## 896 T241 DIA 1
## 897 T250 D649 1
## 898 T302 DIA 1
## 899 T303 DIA 1
## 900 T409 DIA 1
## 901 T477 DIA 1
## 902 T652 DIA 1
## 903 T802 DIA 1
## 904 T818 DIA 1
## 905 T827 DIA 1
## 906 T860 DIA 1
## 907 T876 DIA 1
## 908 T887 DIA 1
## 909 T888 A150 1
## 910 T889 DIA 1
## 911 T983 DIA 1
## 912 W170 DIA 1
## 913 W180 DIA 1
## 914 Y832 A419 1
## 915 Z038 DIA 1
## 916 Z100 DIA 1
## 917 Z208 DIA 1
## 918 Z392 DIA 1
## 919 Z549 DIA 1
## 920 Z639 DIA 1
## 921 Z721 DIA 1
## 922 Z988 DIA 1

### Dead patients, after lockdown

## Diag1 Diag2 Frequency
## 1 U071 DIA 632
## 2 J960 DIA 408
## 3 J189 DIA 370
## 4 J969 DIA 190
## 5 I10X DIA 95
## 6 J80X DIA 59
## 7 J128 DIA 52
## 8 U072 DIA 43
## 9 J129 DIA 41
## 10 N189 DIA 36
## 11 N390 DIA 24
## 12 A418 DIA 18
## 13 E669 DIA 17
## 14 G934 DIA 13
## 15 I219 DIA 13
## 16 I64X DIA 13
## 17 R572 DIA 11
## 18 J159 DIA 10
## 19 K922 DIA 10
## 20 J157 DIA 9
## 21 N179 DIA 9
## 22 R579 DIA 9
## 23 J22X DIA 8
## 24 I469 DIA 7
## 25 I500 DIA 7
## 26 N185 DIA 7
## 27 E039 DIA 6
## 28 E86X DIA 6
## 29 I120 DIA 6
## 30 E872 DIA 5
## 31 I269 DIA 5
## 32 I639 DIA 5
## 33 I679 DIA 5
## 34 J690 DIA 5
## 35 K746 DIA 5
## 36 I252 DIA 4
## 37 I619 DIA 4
## 38 J168 DIA 4
## 39 J841 DIA 4
## 40 R570 DIA 4
## 41 R688 DIA 4
## 42 T136 DIA 4
## 43 I678 DIA 3
## 44 I959 DIA 3
## 45 J81X DIA 3
## 46 J961 DIA 3
## 47 M069 DIA 3
## 48 E162 DIA 2
## 49 E871 DIA 2
## 50 E874 DIA 2
## 51 I259 DIA 2
## 52 I489 DIA 2
## 53 I490 DIA 2
## 54 I634 DIA 2
## 55 I958 DIA 2
## 56 J13X DIA 2
## 57 J150 DIA 2
## 58 J158 DIA 2
## 59 J180 DIA 2
## 60 J320 DIA 2
## 61 J459 DIA 2
## 62 J939 DIA 2
## 63 K566 DIA 2
## 64 K859 DIA 2
## 65 L089 DIA 2
## 66 M726 DIA 2
## 67 N039 DIA 2
## 68 N10X DIA 2
## 69 N172 DIA 2
## 70 N178 DIA 2
## 71 N184 DIA 2
## 72 N19X DIA 2
## 73 R410 DIA 2
## 74 R568 DIA 2
## 75 R571 DIA 2
## 76 R651 DIA 2
## 77 S880 DIA 2
## 78 D379 C786 1
## 79 D500 A419 1
## 80 E440 A419 1
## 81 E660 DIA 1
## 82 E875 DIA 1
## 83 E878 DIA 1
## 84 F100 DIA 1
## 85 F209 DIA 1
## 86 F413 DIA 1
## 87 G952 A419 1
## 88 G969 A419 1
## 89 I119 B972 1
## 90 I130 DIA 1
## 91 I159 DIA 1
## 92 I210 A419 1
## 93 I238 DIA 1
## 94 I249 A419 1
## 95 I251 DIA 1
## 96 I255 DIA 1
## 97 I330 DIA 1
## 98 I429 D599 1
## 99 I461 A419 1
## 100 I480 A419 1
## 101 I48X DIA 1
## 102 I509 DIA 1
## 103 I607 DIA 1
## 104 I609 DIA 1
## 105 I612 DIA 1
## 106 I635 DIA 1
## 107 I670 DIA 1
## 108 I694 DIA 1
## 109 I739 A419 1
## 110 I803 DIA 1
## 111 I822 DIA 1
## 112 I830 DIA 1
## 113 J068 DIA 1
## 114 J09X DIA 1
## 115 J160 DIA 1
## 116 J638 DIA 1
## 117 J849 DIA 1
## 118 J850 DIA 1
## 119 J860 DIA 1
## 120 J869 A419 1
## 121 J90X DIA 1
## 122 J929 DIA 1
## 123 J986 DIA 1
## 124 J989 DIA 1
## 125 J990 DIA 1
## 126 K122 DIA 1
## 127 K269 A419 1
## 128 K431 DIA 1
## 129 K565 DIA 1
## 130 K610 DIA 1
## 131 K703 DIA 1
## 132 K729 DIA 1
## 133 K750 DIA 1
## 134 K850 DIA 1
## 135 K851 A419 1
## 136 K920 DIA 1
## 137 L039 A419 1
## 138 L088 DIA 1
## 139 L899 DIA 1
## 140 L89X A418 1
## 141 L959 DIA 1
## 142 N151 A419 1
## 143 N165 DIA 1
## 144 N289 A419 1
## 145 N498 A419 1
## 146 N499 A419 1
## 147 N764 DIA 1
## 148 R02X DIA 1
## 149 R042 B430 1
## 150 R063 DIA 1
## 151 R092 A150 1
## 152 R100 DIA 1
## 153 R398 DIA 1
## 154 R578 DIA 1
## 155 R931 DIA 1
## 156 S065 A150 1
## 157 S913 DIA 1
## 158 T874 DIA 1
## 159 Y919 DIA 1

## Male

## Diag1 Diag2 Frequency
## 1 I10X DIA 4511
## 2 N390 DIA 2038
## 3 U071 DIA 1674
## 4 N189 DIA 1435
## 5 J189 DIA 1394
## 6 J960 DIA 919
## 7 L031 DIA 677
## 8 A419 DIA 641
## 9 D649 DIA 603
## 10 N40X DIA 524
## 11 J969 DIA 428
## 12 N185 DIA 385
## 13 I500 DIA 355
## 14 E669 DIA 349
## 15 I64X DIA 344
## 16 I219 DIA 294
## 17 B972 DIA 281
## 18 K922 DIA 264
## 19 E162 DIA 259
## 20 J159 DIA 243
## 21 D509 DIA 232
## 22 I639 DIA 221
## 23 I509 DIA 216
## 24 A150 DIA 208
## 25 I678 DIA 205
## 26 J129 DIA 197
## 27 L039 DIA 187
## 28 K746 DIA 185
## 29 S913 DIA 174
## 30 J128 DIA 166
## 31 R739 DIA 162
## 32 A090 DIA 145
## 33 A099 DIA 144
## 34 N039 DIA 144
## 35 I739 DIA 131
## 36 N179 DIA 129
## 37 E86X DIA 127
## 38 I679 DIA 124
## 39 K703 DIA 117
## 40 B24X DIA 115
## 41 N10X DIA 110
## 42 J90X DIA 109
## 43 M869 DIA 107
## 44 L030 DIA 106
## 45 L97X DIA 105
## 46 E039 DIA 101
## 47 K859 DIA 101
## 48 N19X DIA 96
## 49 G409 DIA 95
## 50 J841 DIA 93
## 51 I619 DIA 90
## 52 L024 DIA 88
## 53 S984 DIA 88
## 54 A418 DIA 85
## 55 G934 DIA 84
## 56 R568 DIA 83
## 57 U072 DIA 83
## 58 I489 DIA 80
## 59 J80X DIA 80
## 60 R02X DIA 79
## 61 L038 DIA 76
## 62 R104 DIA 76
## 63 I119 DIA 73
## 64 J849 DIA 73
## 65 I694 DIA 72
## 66 T136 DIA 72
## 67 J47X DIA 69
## 68 S981 DIA 67
## 69 I159 DIA 66
## 70 J459 DIA 66
## 71 J961 DIA 66
## 72 K610 DIA 66
## 73 L029 DIA 66
## 74 D539 DIA 65
## 75 K810 DIA 65
## 76 R509 DIA 64
## 77 D500 DIA 63
## 78 I259 DIA 62
## 79 S889 DIA 62
## 80 A169 DIA 61
## 81 L023 DIA 60
## 82 N180 DIA 60
## 83 I200 DIA 59
## 84 I872 DIA 57
## 85 K802 DIA 57
## 86 K811 DIA 54
## 87 E660 DIA 53
## 88 E668 DIA 53
## 89 E872 DIA 53
## 90 I480 DIA 53
## 91 K805 DIA 53
## 92 G459 DIA 52
## 93 H360 DIA 51
## 94 K358 DIA 51
## 95 J180 DIA 49
## 96 L089 DIA 49
## 97 N498 DIA 49
## 98 L899 DIA 48
## 99 L032 DIA 47
## 100 N181 DIA 47
## 101 E46X DIA 46
## 102 N200 DIA 46
## 103 K409 DIA 45
## 104 J209 DIA 43
## 105 N183 DIA 43
## 106 I120 DIA 42
## 107 N110 DIA 42
## 108 I48X DIA 41
## 109 J188 DIA 41
## 110 N12X DIA 41
## 111 J22X DIA 40
## 112 N139 DIA 40
## 113 C61X DIA 39
## 114 E780 DIA 39
## 115 E785 DIA 39
## 116 N184 DIA 39
## 117 R042 DIA 38
## 118 I110 DIA 37
## 119 L984 DIA 37
## 120 F200 DIA 36
## 121 J852 DIA 36
## 122 L409 DIA 36
## 123 R11X DIA 36
## 124 S982 DIA 36
## 125 I633 DIA 35
## 126 I802 DIA 35
## 127 K85X DIA 34
## 128 E160 DIA 33
## 129 F209 DIA 33
## 130 I499 DIA 33
## 131 I610 DIA 33
## 132 I830 DIA 33
## 133 K729 DIA 33
## 134 N049 DIA 33
## 135 R572 DIA 33
## 136 S069 DIA 33
## 137 D648 DIA 32
## 138 J120 DIA 32
## 139 M109 DIA 32
## 140 S819 DIA 32
## 141 E782 DIA 31
## 142 F102 DIA 31
## 143 J869 DIA 31
## 144 I209 DIA 30
## 145 J157 DIA 30
## 146 K429 DIA 30
## 147 K566 DIA 30
## 148 K750 DIA 30
## 149 K801 DIA 30
## 150 L022 DIA 30
## 151 L890 DIA 30
## 152 J13X DIA 29
## 153 K590 DIA 29
## 154 A409 DIA 28
## 155 E871 DIA 28
## 156 I829 DIA 28
## 157 K259 DIA 28
## 158 N289 DIA 28
## 159 C169 DIA 27
## 160 J81X DIA 27
## 161 K295 DIA 27
## 162 K529 DIA 27
## 163 M725 DIA 27
## 164 N459 DIA 27
## 165 A09X DIA 26
## 166 A153 DIA 26
## 167 I634 DIA 26
## 168 I255 DIA 25
## 169 I698 DIA 25
## 170 K291 DIA 25
## 171 K769 DIA 25
## 172 N492 DIA 25
## 173 T874 DIA 25
## 174 K800 DIA 24
## 175 L021 DIA 24
## 176 R18X DIA 24
## 177 I609 DIA 23
## 178 J029 DIA 23
## 179 J158 DIA 23
## 180 J168 DIA 23
## 181 S681 DIA 23
## 182 S880 DIA 23
## 183 T009 DIA 23
## 184 G590 DIA 22
## 185 H269 DIA 22
## 186 I630 DIA 22
## 187 I693 DIA 22
## 188 I792 DIA 22
## 189 I832 DIA 22
## 190 L020 DIA 22
## 191 L028 DIA 22
## 192 N151 DIA 22
## 193 R33X DIA 22
## 194 E870 DIA 21
## 195 I442 DIA 21
## 196 I859 DIA 21
## 197 J150 DIA 21
## 198 J181 DIA 21
## 199 K830 DIA 21
## 200 S065 DIA 21
## 201 S911 DIA 21
## 202 D638 DIA 20
## 203 E876 DIA 20
## 204 G20X DIA 20
## 205 G629 DIA 20
## 206 K819 DIA 20
## 207 N319 DIA 20
## 208 R571 DIA 20
## 209 R609 DIA 20
## 210 E161 DIA 19
## 211 G632 DIA 19
## 212 I210 DIA 19
## 213 I469 DIA 19
## 214 I620 DIA 19
## 215 K30X DIA 19
## 216 M009 DIA 19
## 217 N399 DIA 19
## 218 N499 DIA 19
## 219 R17X DIA 19
## 220 R579 DIA 19
## 221 S822 DIA 19
## 222 T814 DIA 19
## 223 B86X DIA 18
## 224 D696 DIA 18
## 225 E889 DIA 18
## 226 I340 DIA 18
## 227 J152 DIA 18
## 228 M726 DIA 18
## 229 R100 DIA 18
## 230 R31X DIA 18
## 231 A499 DIA 17
## 232 E059 DIA 17
## 233 E789 DIA 17
## 234 E880 DIA 17
## 235 G819 DIA 17
## 236 J984 DIA 17
## 237 N111 DIA 17
## 238 N182 DIA 17
## 239 R51X DIA 17
## 240 R570 DIA 17
## 241 S912 DIA 17
## 242 E43X DIA 16
## 243 F329 DIA 16
## 244 G610 DIA 16
## 245 I635 DIA 16
## 246 K047 DIA 16
## 247 K122 DIA 16
## 248 S881 DIA 16
## 249 E878 DIA 15
## 250 G510 DIA 15
## 251 H540 DIA 15
## 252 I251 DIA 15
## 253 I490 DIA 15
## 254 I519 DIA 15
## 255 I743 DIA 15
## 256 K37X DIA 15
## 257 K439 DIA 15
## 258 K591 DIA 15
## 259 L80X DIA 15
## 260 T141 DIA 15
## 261 E249 DIA 14
## 262 E440 DIA 14
## 263 I151 DIA 14
## 264 I269 DIA 14
## 265 I629 DIA 14
## 266 I702 DIA 14
## 267 I959 DIA 14
## 268 J40X DIA 14
## 269 K603 DIA 14
## 270 S789 DIA 14
## 271 T793 DIA 14
## 272 C900 DIA 13
## 273 F03X DIA 13
## 274 F419 DIA 13
## 275 H813 DIA 13
## 276 I612 DIA 13
## 277 I771 DIA 13
## 278 I850 DIA 13
## 279 J851 DIA 13
## 280 L033 DIA 13
## 281 L400 DIA 13
## 282 M139 DIA 13
## 283 N450 DIA 13
## 284 N47X DIA 13
## 285 R418 DIA 13
## 286 S721 DIA 13
## 287 B370 DIA 12
## 288 C229 DIA 12
## 289 E781 DIA 12
## 290 E835 DIA 12
## 291 F321 DIA 12
## 292 G049 DIA 12
## 293 G969 DIA 12
## 294 H819 DIA 12
## 295 I249 DIA 12
## 296 I618 DIA 12
## 297 I638 DIA 12
## 298 J069 DIA 12
## 299 J698 DIA 12
## 300 J939 DIA 12
## 301 K564 DIA 12
## 302 K920 DIA 12
## 303 M069 DIA 12
## 304 N119 DIA 12
## 305 N172 DIA 12
## 306 S818 DIA 12
## 307 T876 DIA 12
## 308 Y835 DIA 12
## 309 A971 DIA 11
## 310 G042 DIA 11
## 311 G403 DIA 11
## 312 G909 DIA 11
## 313 H280 DIA 11
## 314 I252 DIA 11
## 315 I420 DIA 11
## 316 K250 DIA 11
## 317 K279 DIA 11
## 318 K709 DIA 11
## 319 K851 DIA 11
## 320 L088 DIA 11
## 321 L89X DIA 11
## 322 N083 DIA 11
## 323 N188 DIA 11
## 324 T887 DIA 11
## 325 B351 DIA 10
## 326 C259 DIA 10
## 327 E271 DIA 10
## 328 E441 DIA 10
## 329 E875 DIA 10
## 330 H409 DIA 10
## 331 I150 DIA 10
## 332 I258 DIA 10
## 333 I443 DIA 10
## 334 I632 DIA 10
## 335 I828 DIA 10
## 336 I839 DIA 10
## 337 J068 DIA 10
## 338 J440 DIA 10
## 339 K402 DIA 10
## 340 M100 DIA 10
## 341 N133 DIA 10
## 342 N170 DIA 10
## 343 N481 DIA 10
## 344 R688 DIA 10
## 345 S682 DIA 10
## 346 S917 DIA 10
## 347 A400 DIA 9
## 348 B354 DIA 9
## 349 B378 DIA 9
## 350 C220 DIA 9
## 351 D410 DIA 9
## 352 G408 DIA 9
## 353 G932 DIA 9
## 354 K269 DIA 9
## 355 K650 DIA 9
## 356 K745 DIA 9
## 357 K808 DIA 9
## 358 L080 DIA 9
## 359 L88X DIA 9
## 360 N040 DIA 9
## 361 N178 DIA 9
## 362 N23X DIA 9
## 363 R072 DIA 9
## 364 R101 DIA 9
## 365 S610 DIA 9
## 366 S729 DIA 9
## 367 S983 DIA 9
## 368 T147 DIA 9
## 369 B829 DIA 8
## 370 C910 DIA 8
## 371 D609 DIA 8
## 372 E169 DIA 8
## 373 E220 DIA 8
## 374 F412 DIA 8
## 375 G255 DIA 8
## 376 G309 DIA 8
## 377 G401 DIA 8
## 378 G990 DIA 8
## 379 I129 DIA 8
## 380 I158 DIA 8
## 381 I250 DIA 8
## 382 I803 DIA 8
## 383 J100 DIA 8
## 384 J151 DIA 8
## 385 J156 DIA 8
## 386 J219 DIA 8
## 387 J42X DIA 8
## 388 J46X DIA 8
## 389 J850 DIA 8
## 390 K046 DIA 8
## 391 K260 DIA 8
## 392 K760 DIA 8
## 393 K928 DIA 8
## 394 N159 DIA 8
## 395 N411 DIA 8
## 396 N433 DIA 8
## 397 R001 DIA 8
## 398 R578 DIA 8
## 399 R64X DIA 8
## 400 R770 DIA 8
## 401 S223 DIA 8
## 402 S619 DIA 8
## 403 S781 DIA 8
## 404 S920 DIA 8
## 405 T131 DIA 8
## 406 T252 DIA 8
## 407 T600 DIA 8
## 408 B009 DIA 7
## 409 F100 DIA 7
## 410 G060 DIA 7
## 411 G569 DIA 7
## 412 G939 DIA 7
## 413 H82X DIA 7
## 414 I130 DIA 7
## 415 I213 DIA 7
## 416 I350 DIA 7
## 417 I481 DIA 7
## 418 I498 DIA 7
## 419 I749 DIA 7
## 420 I800 DIA 7
## 421 I99X DIA 7
## 422 J448 DIA 7
## 423 J848 DIA 7
## 424 J948 DIA 7
## 425 J980 DIA 7
## 426 K219 DIA 7
## 427 K270 DIA 7
## 428 K353 DIA 7
## 429 K469 DIA 7
## 430 K579 DIA 7
## 431 K612 DIA 7
## 432 K717 DIA 7
## 433 K719 DIA 7
## 434 K839 DIA 7
## 435 K858 DIA 7
## 436 L309 DIA 7
## 437 L892 DIA 7
## 438 L893 DIA 7
## 439 M868 DIA 7
## 440 N281 DIA 7
## 441 R53X DIA 7
## 442 R55X DIA 7
## 443 R560 DIA 7
## 444 S328 DIA 7
## 445 B353 DIA 6
## 446 F011 DIA 6
## 447 F410 DIA 6
## 448 G039 DIA 6
## 449 G410 DIA 6
## 450 G589 DIA 6
## 451 H050 DIA 6
## 452 H350 DIA 6
## 453 H669 DIA 6
## 454 H811 DIA 6
## 455 I059 DIA 6
## 456 I152 DIA 6
## 457 I211 DIA 6
## 458 I440 DIA 6
## 459 I471 DIA 6
## 460 I479 DIA 6
## 461 I611 DIA 6
## 462 I801 DIA 6
## 463 J631 DIA 6
## 464 J91X DIA 6
## 465 J930 DIA 6
## 466 K20X DIA 6
## 467 K296 DIA 6
## 468 K351 DIA 6
## 469 K352 DIA 6
## 470 K420 DIA 6
## 471 K565 DIA 6
## 472 K605 DIA 6
## 473 K630 DIA 6
## 474 K632 DIA 6
## 475 K721 DIA 6
## 476 K740 DIA 6
## 477 K759 DIA 6
## 478 M199 DIA 6
## 479 M329 DIA 6
## 480 M609 DIA 6
## 481 M729 DIA 6
## 482 M861 DIA 6
## 483 M866 DIA 6
## 484 N209 DIA 6
## 485 N220 DIA 6
## 486 N309 DIA 6
## 487 N359 DIA 6
## 488 R42X DIA 6
## 489 S062 DIA 6
## 490 S817 DIA 6
## 491 S820 DIA 6
## 492 T059 DIA 6
## 493 T140 DIA 6
## 494 T633 DIA 6
## 495 T888 DIA 6
## 496 43552 DIA 5
## 497 43556 DIA 5
## 498 43565 DIA 5
## 499 C329 DIA 5
## 500 D414 DIA 5
## 501 F019 DIA 5
## 502 F103 DIA 5
## 503 F109 DIA 5
## 504 F432 DIA 5
## 505 G009 DIA 5
## 506 G400 DIA 5
## 507 G402 DIA 5
## 508 G619 DIA 5
## 509 G919 DIA 5
## 510 G936 DIA 5
## 511 H659 DIA 5
## 512 H919 DIA 5
## 513 I060 DIA 5
## 514 I229 DIA 5
## 515 I330 DIA 5
## 516 I441 DIA 5
## 517 I48 DIA 5
## 518 I516 DIA 5
## 519 I613 DIA 5
## 520 I688 DIA 5
## 521 I744 DIA 5
## 522 I831 DIA 5
## 523 I891 DIA 5
## 524 J019 DIA 5
## 525 J123 DIA 5
## 526 J450 DIA 5
## 527 J942 DIA 5
## 528 K102 DIA 5
## 529 K273 DIA 5
## 530 K318 DIA 5
## 531 K648 DIA 5
## 532 K720 DIA 5
## 533 K804 DIA 5
## 534 K828 DIA 5
## 535 M146 DIA 5
## 536 M490 DIA 5
## 537 M544 DIA 5
## 538 M600 DIA 5
## 539 M705 DIA 5
## 540 M793 DIA 5
## 541 M809 DIA 5
## 542 N009 DIA 5
## 543 N310 DIA 5
## 544 N512 DIA 5
## 545 Q667 DIA 5
## 546 R000 DIA 5
## 547 R074 DIA 5
## 548 R410 DIA 5
## 549 R798 DIA 5
## 550 S723 DIA 5
## 551 S810 DIA 5
## 552 S828 DIA 5
## 553 T242 DIA 5
## 554 T243 DIA 5
## 555 T253 DIA 5
## 556 T824 DIA 5
## 557 0 DIA 4
## 558 43540 DIA 4
## 559 43577 DIA 4
## 560 A480 DIA 4
## 561 D589 DIA 4
## 562 E038 DIA 4
## 563 E050 DIA 4
## 564 E230 DIA 4
## 565 E240 DIA 4
## 566 E272 DIA 4
## 567 F067 DIA 4
## 568 F130 DIA 4
## 569 F192 DIA 4
## 570 F411 DIA 4
## 571 F449 DIA 4
## 572 G041 DIA 4
## 573 G08X DIA 4
## 574 G442 DIA 4
## 575 G628 DIA 4
## 576 G92X DIA 4
## 577 G931 DIA 4
## 578 H259 DIA 4
## 579 H46X DIA 4
## 580 I248 DIA 4
## 581 I270 DIA 4
## 582 I601 DIA 4
## 583 I631 DIA 4
## 584 I660 DIA 4
## 585 I674 DIA 4
## 586 I770 DIA 4
## 587 I775 DIA 4
## 588 I778 DIA 4
## 589 J14X DIA 4
## 590 J441 DIA 4
## 591 J680 DIA 4
## 592 J938 DIA 4
## 593 J949 DIA 4
## 594 J988 DIA 4
## 595 K319 DIA 4
## 596 K350 DIA 4
## 597 K359 DIA 4
## 598 K562 DIA 4
## 599 K739 DIA 4
## 600 K803 DIA 4
## 601 K929 DIA 4
## 602 L100 DIA 4
## 603 L405 DIA 4
## 604 L500 DIA 4
## 605 L539 DIA 4
## 606 L891 DIA 4
## 607 M131 DIA 4
## 608 M169 DIA 4
## 609 M430 DIA 4
## 610 M541 DIA 4
## 611 M860 DIA 4
## 612 M879 DIA 4
## 613 M939 DIA 4
## 614 N029 DIA 4
## 615 N312 DIA 4
## 616 R160 DIA 4
## 617 R529 DIA 4
## 618 R54X DIA 4
## 619 R601 DIA 4
## 620 R69X DIA 4
## 621 S010 DIA 4
## 622 S024 DIA 4
## 623 S099 DIA 4
## 624 S399 DIA 4
## 625 S411 DIA 4
## 626 S618 DIA 4
## 627 S701 DIA 4
## 628 S718 DIA 4
## 629 S824 DIA 4
## 630 S910 DIA 4
## 631 T019 DIA 4
## 632 T054 DIA 4
## 633 T149 DIA 4
## 634 T813 DIA 4
## 635 T827 DIA 4
## 636 T856 DIA 4
## 637 T857 DIA 4
## 638 Y919 DIA 4
## 639 Z896 DIA 4
## 640 43561 DIA 3
## 641 E02X DIA 3
## 642 E512 DIA 3
## 643 E538 DIA 3
## 644 E756 DIA 3
## 645 E784 DIA 3
## 646 E831 DIA 3
## 647 E888 DIA 3
## 648 F058 DIA 3
## 649 F069 DIA 3
## 650 F09X DIA 3
## 651 F142 DIA 3
## 652 F172 DIA 3
## 653 F238 DIA 3
## 654 F328 DIA 3
## 655 F609 DIA 3
## 656 G01X DIA 3
## 657 G020 DIA 3
## 658 G040 DIA 3
## 659 G062 DIA 3
## 660 G09X DIA 3
## 661 G219 DIA 3
## 662 G373 DIA 3
## 663 G458 DIA 3
## 664 G531 DIA 3
## 665 G578 DIA 3
## 666 G822 DIA 3
## 667 G900 DIA 3
## 668 G938 DIA 3
## 669 G952 DIA 3
## 670 H059 DIA 3
## 671 H400 DIA 3
## 672 H609 DIA 3
## 673 H653 DIA 3
## 674 H660 DIA 3
## 675 I051 DIA 3
## 676 I212 DIA 3
## 677 I241 DIA 3
## 678 I451 DIA 3
## 679 I459 DIA 3
## 680 I495 DIA 3
## 681 I600 DIA 3
## 682 I670 DIA 3
## 683 I671 DIA 3
## 684 I729 DIA 3
## 685 I742 DIA 3
## 686 I748 DIA 3
## 687 I809 DIA 3
## 688 I821 DIA 3
## 689 I879 DIA 3
## 690 J160 DIA 3
## 691 J178 DIA 3
## 692 J342 DIA 3
## 693 K029 DIA 3
## 694 K221 DIA 3
## 695 K257 DIA 3
## 696 K421 DIA 3
## 697 K560 DIA 3
## 698 K602 DIA 3
## 699 K639 DIA 3
## 700 K658 DIA 3
## 701 K700 DIA 3
## 702 K704 DIA 3
## 703 K743 DIA 3
## 704 K768 DIA 3
## 705 K829 DIA 3
## 706 K831 DIA 3
## 707 K838 DIA 3
## 708 K868 DIA 3
## 709 L219 DIA 3
## 710 L401 DIA 3
## 711 L408 DIA 3
## 712 L509 DIA 3
## 713 L719 DIA 3
## 714 M029 DIA 3
## 715 M059 DIA 3
## 716 M073 DIA 3
## 717 M480 DIA 3
## 718 M511 DIA 3
## 719 M512 DIA 3
## 720 M549 DIA 3
## 721 M720 DIA 3
## 722 M728 DIA 3
## 723 M819 DIA 3
## 724 M878 DIA 3
## 725 N048 DIA 3
## 726 N059 DIA 3
## 727 N130 DIA 3
## 728 N202 DIA 3
## 729 N210 DIA 3
## 730 N219 DIA 3
## 731 N320 DIA 3
## 732 N44X DIA 3
## 733 N482 DIA 3
## 734 Q610 DIA 3
## 735 R040 DIA 3
## 736 R092 DIA 3
## 737 R162 DIA 3
## 738 R190 DIA 3
## 739 R392 DIA 3
## 740 R58X DIA 3
## 741 R600 DIA 3
## 742 R960 DIA 3
## 743 S008 DIA 3
## 744 S009 DIA 3
## 745 S064 DIA 3
## 746 S066 DIA 3
## 747 S109 DIA 3
## 748 S224 DIA 3
## 749 S271 DIA 3
## 750 S311 DIA 3
## 751 S313 DIA 3
## 752 S324 DIA 3
## 753 S369 DIA 3
## 754 S519 DIA 3
## 755 S611 DIA 3
## 756 S683 DIA 3
## 757 S711 DIA 3
## 758 S780 DIA 3
## 759 S821 DIA 3
## 760 S925 DIA 3
## 761 S929 DIA 3
## 762 T302 DIA 3
## 763 T303 DIA 3
## 764 T519 DIA 3
## 765 T659 DIA 3
## 766 W179 DIA 3
## 767 W199 DIA 3
## 768 Z488 DIA 3
## 769 Z519 DIA 3
## 770 Z608 DIA 3
## 771 Z950 DIA 3
## 772 43525 S889 2
## 773 43530 I10X 2
## 774 43547 DIA 2
## 775 43549 J159 2
## 776 43550 DIA 2
## 777 43554 S881 2
## 778 43558 DIA 2
## 779 43559 DIA 2
## 780 43564 DIA 2
## 781 43568 E86X 2
## 782 A083 DIA 2
## 783 A852 DIA 2
## 784 B449 J47X 2
## 785 B878 DIA 2
## 786 C258 DIA 2
## 787 C348 DIA 2
## 788 C840 DIA 2
## 789 D042 DIA 2
## 790 D151 DIA 2
## 791 D590 DIA 2
## 792 D591 DIA 2
## 793 D642 DIA 2
## 794 E041 DIA 2
## 795 E212 DIA 2
## 796 E713 DIA 2
## 797 E722 DIA 2
## 798 E739 DIA 2
## 799 E832 DIA 2
## 800 E873 A419 2
## 801 E874 DIA 2
## 802 F009 DIA 2
## 803 F018 DIA 2
## 804 F050 DIA 2
## 805 F059 DIA 2
## 806 F101 DIA 2
## 807 F104 DIA 2
## 808 F106 DIA 2
## 809 F199 DIA 2
## 810 F250 DIA 2
## 811 F29X DIA 2
## 812 F322 DIA 2
## 813 F339 DIA 2
## 814 F413 DIA 2
## 815 F429 DIA 2
## 816 F445 DIA 2
## 817 F721 DIA 2
## 818 G032 DIA 2
## 819 G048 DIA 2
## 820 G050 DIA 2
## 821 G058 DIA 2
## 822 G10X DIA 2
## 823 G131 DIA 2
## 824 G310 DIA 2
## 825 G311 DIA 2
## 826 G328 DIA 2
## 827 G35X DIA 2
## 828 G405 DIA 2
## 829 G419 DIA 2
## 830 G450 DIA 2
## 831 G464 DIA 2
## 832 G509 DIA 2
## 833 G579 DIA 2
## 834 G609 DIA 2
## 835 G611 DIA 2
## 836 G700 DIA 2
## 837 G808 DIA 2
## 838 G820 DIA 2
## 839 G825 DIA 2
## 840 G911 DIA 2
## 841 G959 DIA 2
## 842 G98X DIA 2
## 843 H109 DIA 2
## 844 H358 DIA 2
## 845 H544 DIA 2
## 846 H588 DIA 2
## 847 H602 DIA 2
## 848 H650 DIA 2
## 849 H708 DIA 2
## 850 H730 DIA 2
## 851 H900 DIA 2
## 852 H902 DIA 2
## 853 I050 DIA 2
## 854 I061 DIA 2
## 855 I089 DIA 2
## 856 I131 DIA 2
## 857 I139 DIA 2
## 858 I201 DIA 2
## 859 I240 DIA 2
## 860 I278 DIA 2
## 861 I279 DIA 2
## 862 I289 DIA 2
## 863 I313 DIA 2
## 864 I352 DIA 2
## 865 I358 DIA 2
## 866 I379 DIA 2
## 867 I400 DIA 2
## 868 I460 DIA 2
## 869 I461 DIA 2
## 870 I472 DIA 2
## 871 I482 DIA 2
## 872 I517 DIA 2
## 873 I615 DIA 2
## 874 I616 DIA 2
## 875 I659 R568 2
## 876 I663 DIA 2
## 877 I691 DIA 2
## 878 I700 DIA 2
## 879 I709 DIA 2
## 880 I738 DIA 2
## 881 I741 DIA 2
## 882 I776 DIA 2
## 883 I842 DIA 2
## 884 I864 DIA 2
## 885 I868 DIA 2
## 886 I871 DIA 2
## 887 I983 DIA 2
## 888 J014 DIA 2
## 889 J039 DIA 2
## 890 J040 DIA 2
## 891 J101 DIA 2
## 892 J15 E11 2
## 893 J154 DIA 2
## 894 J340 DIA 2
## 895 J36X DIA 2
## 896 J390 DIA 2
## 897 J398 DIA 2
## 898 J41 DIA 2
## 899 J438 DIA 2
## 900 J61X DIA 2
## 901 J64X DIA 2
## 902 J679 DIA 2
## 903 J840 DIA 2
## 904 J853 DIA 2
## 905 J931 DIA 2
## 906 K044 DIA 2
## 907 K050 DIA 2
## 908 K052 DIA 2
## 909 K113 DIA 2
## 910 K121 DIA 2
## 911 K222 DIA 2
## 912 K253 DIA 2
## 913 K254 DIA 2
## 914 K294 DIA 2
## 915 K298 DIA 2
## 916 K36X DIA 2
## 917 K419 DIA 2
## 918 K449 DIA 2
## 919 K500 DIA 2
## 920 K522 DIA 2
## 921 K550 DIA 2
## 922 K567 DIA 2
## 923 K570 DIA 2
## 924 K593 DIA 2
## 925 K626 DIA 2
## 926 K638 DIA 2
## 927 K649 DIA 2
## 928 K701 DIA 2
## 929 K752 DIA 2
## 930 K758 DIA 2
## 931 K766 DIA 2
## 932 K818 DIA 2
## 933 K822 DIA 2
## 934 K834 DIA 2
## 935 K85 DIA 2
## 936 K860 DIA 2
## 937 K861 DIA 2
## 938 K862 DIA 2
## 939 K869 DIA 2
## 940 K900 DIA 2
## 941 K914 DIA 2
## 942 L043 DIA 2
## 943 L109 DIA 2
## 944 L209 DIA 2
## 945 L259 DIA 2
## 946 L281 DIA 2
## 947 L729 DIA 2
## 948 L988 DIA 2
## 949 M013 DIA 2
## 950 M104 DIA 2
## 951 M130 DIA 2
## 952 M148 DIA 2
## 953 M150 DIA 2
## 954 M311 DIA 2
## 955 M313 DIA 2
## 956 M353 DIA 2
## 957 M360 DIA 2
## 958 M431 DIA 2
## 959 M45X DIA 2
## 960 M479 DIA 2
## 961 M489 DIA 2
## 962 M494 DIA 2
## 963 M543 DIA 2
## 964 M626 DIA 2
## 965 M704 DIA 2
## 966 M715 DIA 2
## 967 M723 DIA 2
## 968 M724 DIA 2
## 969 M779 DIA 2
## 970 M863 DIA 2
## 971 M864 DIA 2
## 972 M899 DIA 2
## 973 M932 DIA 2
## 974 M952 DIA 2
## 975 N020 DIA 2
## 976 N042 DIA 2
## 977 N071 DIA 2
## 978 N118 DIA 2
## 979 N131 DIA 2
## 980 N132 DIA 2
## 981 N138 DIA 2
## 982 N144 DIA 2
## 983 N158 DIA 2
## 984 N251 DIA 2
## 985 N258 DIA 2
## 986 N302 DIA 2
## 987 N321 DIA 2
## 988 N322 DIA 2
## 989 N328 DIA 2
## 990 N340 DIA 2
## 991 N341 DIA 2
## 992 N398 DIA 2
## 993 N40 DIA 2
## 994 N434 DIA 2
## 995 N485 DIA 2
## 996 N508 DIA 2
## 997 N990 DIA 2
## 998 Q249 DIA 2
## 999 Q283 DIA 2
## 1000 Q909 DIA 2
## 1001 R05X DIA 2
## 1002 R060 DIA 2
## 1003 R066 DIA 2
## 1004 R073 DIA 2
## 1005 R091 DIA 2
## 1006 R13X DIA 2
## 1007 R221 DIA 2
## 1008 R229 DIA 2
## 1009 R32X DIA 2
## 1010 R36X DIA 2
## 1011 R402 DIA 2
## 1012 R451 DIA 2
## 1013 R488 DIA 2
## 1014 R490 DIA 2
## 1015 R650 DIA 2
## 1016 S019 DIA 2
## 1017 S029 DIA 2
## 1018 S051 DIA 2
## 1019 S059 DIA 2
## 1020 S089 DIA 2
## 1021 S202 DIA 2
## 1022 S272 DIA 2
## 1023 S279 DIA 2
## 1024 S299 DIA 2
## 1025 S310 DIA 2
## 1026 S320 DIA 2
## 1027 S323 DIA 2
## 1028 S430 DIA 2
## 1029 S480 DIA 2
## 1030 S518 DIA 2
## 1031 S522 DIA 2
## 1032 S525 DIA 2
## 1033 S626 DIA 2
## 1034 S699 DIA 2
## 1035 S724 DIA 2
## 1036 S728 DIA 2
## 1037 S825 DIA 2
## 1038 S826 DIA 2
## 1039 S827 DIA 2
## 1040 S829 DIA 2
## 1041 S842 DIA 2
## 1042 S921 DIA 2
## 1043 T07X DIA 2
## 1044 T10X DIA 2
## 1045 T130 DIA 2
## 1046 T143 DIA 2
## 1047 T240 DIA 2
## 1048 T246 DIA 2
## 1049 T630 DIA 2
## 1050 T68X DIA 2
## 1051 T702 DIA 2
## 1052 T829 DIA 2
## 1053 T871 DIA 2
## 1054 T875 DIA 2
## 1055 T886 DIA 2
## 1056 T889 DIA 2
## 1057 T905 DIA 2
## 1058 T930 DIA 2
## 1059 U069 DIA 2
## 1060 W019 DIA 2
## 1061 Y579 DIA 2
## 1062 Y841 DIA 2
## 1063 Y912 DIA 2
## 1064 Z540 DIA 2
## 1065 A513 A162 1
## 1066 A540 A150 1
## 1067 A812 A419 1
## 1068 B207 A090 1
## 1069 B24 A154 1
## 1070 B358 B24X 1
## 1071 B427 A419 1
## 1072 B451 B009 1
## 1073 B779 A071 1
## 1074 C091 A419 1
## 1075 C449 A419 1
## 1076 C787 C629 1
## 1077 D136 B24X 1
## 1078 D199 B370 1
## 1079 D373 A162 1
## 1080 D382 A165 1
## 1081 D630 B222 1
## 1082 D65X A150 1
## 1083 D688 A419 1
## 1084 D695 A278 1
## 1085 D699 D649 1
## 1086 D70X B370 1
## 1087 D729 D500 1
## 1088 E031 DIA 1
## 1089 E063 DIA 1
## 1090 E069 DIA 1
## 1091 E15X DIA 1
## 1092 E168 DIA 1
## 1093 E210 DIA 1
## 1094 E236 DIA 1
## 1095 E248 D694 1
## 1096 E270 DIA 1
## 1097 E273 DIA 1
## 1098 E274 DIA 1
## 1099 E348 DIA 1
## 1100 E40X DIA 1
## 1101 E449 DIA 1
## 1102 E539 DIA 1
## 1103 E559 DIA 1
## 1104 E60X DIA 1
## 1105 E639 DIA 1
## 1106 E709 DIA 1
## 1107 E728 DIA 1
## 1108 E738 D509 1
## 1109 E750 DIA 1
## 1110 E768 DIA 1
## 1111 E786 DIA 1
## 1112 E788 DIA 1
## 1113 E790 DIA 1
## 1114 E804 DIA 1
## 1115 E849 DIA 1
## 1116 E882 DIA 1
## 1117 F002 DIA 1
## 1118 F012 DIA 1
## 1119 F013 DIA 1
## 1120 F020 DIA 1
## 1121 F023 DIA 1
## 1122 F062 DIA 1
## 1123 F064 DIA 1
## 1124 F078 DIA 1
## 1125 F079 DIA 1
## 1126 F116 DIA 1
## 1127 F132 DIA 1
## 1128 F135 D509 1
## 1129 F194 DIA 1
## 1130 F201 D649 1
## 1131 F230 DIA 1
## 1132 F232 DIA 1
## 1133 F239 DIA 1
## 1134 F31 DIA 1
## 1135 F310 DIA 1
## 1136 F319 DIA 1
## 1137 F320 DIA 1
## 1138 F323 DIA 1
## 1139 F440 DIA 1
## 1140 F444 DIA 1
## 1141 F481 DIA 1
## 1142 F500 DIA 1
## 1143 F514 DIA 1
## 1144 F602 DIA 1
## 1145 F608 DIA 1
## 1146 F61X DIA 1
## 1147 F639 DIA 1
## 1148 F669 DIA 1
## 1149 F709 DIA 1
## 1150 F719 DIA 1
## 1151 F729 DIA 1
## 1152 F790 DIA 1
## 1153 F799 DIA 1
## 1154 F808 DIA 1
## 1155 F82X DIA 1
## 1156 F844 DIA 1
## 1157 F88X DIA 1
## 1158 F900 DIA 1
## 1159 F909 DIA 1
## 1160 F919 DIA 1
## 1161 G030 DIA 1
## 1162 G038 DIA 1
## 1163 G051 DIA 1
## 1164 G061 DIA 1
## 1165 G129 DIA 1
## 1166 G218 DIA 1
## 1167 G22X DIA 1
## 1168 G249 DIA 1
## 1169 G250 DIA 1
## 1170 G258 DIA 1
## 1171 G300 C169 1
## 1172 G368 DIA 1
## 1173 G418 DIA 1
## 1174 G430 DIA 1
## 1175 G432 DIA 1
## 1176 G439 DIA 1
## 1177 G440 DIA 1
## 1178 G444 DIA 1
## 1179 G448 DIA 1
## 1180 G45 DIA 1
## 1181 G452 DIA 1
## 1182 G462 DIA 1
## 1183 G465 D180 1
## 1184 G468 DIA 1
## 1185 G500 DIA 1
## 1186 G528 DIA 1
## 1187 G529 DIA 1
## 1188 G530 DIA 1
## 1189 G540 DIA 1
## 1190 G544 DIA 1
## 1191 G560 DIA 1
## 1192 G561 DIA 1
## 1193 G588 DIA 1
## 1194 G638 DIA 1
## 1195 G64X DIA 1
## 1196 G729 DIA 1
## 1197 G902 DIA 1
## 1198 G912 DIA 1
## 1199 G930 DIA 1
## 1200 G948 DIA 1
## 1201 G951 DIA 1
## 1202 G958 DIA 1
## 1203 G961 DIA 1
## 1204 G970 DIA 1
## 1205 H031 B023 1
## 1206 H041 DIA 1
## 1207 H043 DIA 1
## 1208 H100 DIA 1
## 1209 H105 DIA 1
## 1210 H110 DIA 1
## 1211 H150 A539 1
## 1212 H151 DIA 1
## 1213 H160 DIA 1
## 1214 H162 DIA 1
## 1215 H193 DIA 1
## 1216 H209 DIA 1
## 1217 H218 DIA 1
## 1218 H220 A514 1
## 1219 H250 DIA 1
## 1220 H262 DIA 1
## 1221 H268 DIA 1
## 1222 H309 DIA 1
## 1223 H359 DIA 1
## 1224 H368 DIA 1
## 1225 H438 D649 1
## 1226 H449 DIA 1
## 1227 H490 DIA 1
## 1228 H521 DIA 1
## 1229 H523 DIA 1
## 1230 H530 DIA 1
## 1231 H533 DIA 1
## 1232 H542 DIA 1
## 1233 H549 DIA 1
## 1234 H600 DIA 1
## 1235 H601 DIA 1
## 1236 H603 DIA 1
## 1237 H60X DIA 1
## 1238 H622 DIA 1
## 1239 H651 DIA 1
## 1240 H652 DIA 1
## 1241 H664 DIA 1
## 1242 H678 DIA 1
## 1243 H810 DIA 1
## 1244 H814 D500 1
## 1245 H903 DIA 1
## 1246 H905 DIA 1
## 1247 H911 DIA 1
## 1248 I069 DIA 1
## 1249 I070 DIA 1
## 1250 I079 DIA 1
## 1251 I080 DIA 1
## 1252 I081 DIA 1
## 1253 I091 DIA 1
## 1254 I12 DIA 1
## 1255 I132 D649 1
## 1256 I208 DIA 1
## 1257 I21 D50 1
## 1258 I253 DIA 1
## 1259 I254 DIA 1
## 1260 I260 DIA 1
## 1261 I28 E11 1
## 1262 I300 C300 1
## 1263 I301 DIA 1
## 1264 I309 DIA 1
## 1265 I311 DIA 1
## 1266 I319 D649 1
## 1267 I348 DIA 1
## 1268 I369 DIA 1
## 1269 I378 DIA 1
## 1270 I38X DIA 1
## 1271 I409 DIA 1
## 1272 I421 DIA 1
## 1273 I429 DIA 1
## 1274 I438 DIA 1
## 1275 I447 DIA 1
## 1276 I452 A418 1
## 1277 I453 DIA 1
## 1278 I454 DIA 1
## 1279 I456 DIA 1
## 1280 I501 DIA 1
## 1281 I510 DIA 1
## 1282 I614 DIA 1
## 1283 I636 DIA 1
## 1284 I650 DIA 1
## 1285 I651 DIA 1
## 1286 I652 DIA 1
## 1287 I664 DIA 1
## 1288 I690 DIA 1
## 1289 I692 DIA 1
## 1290 I708 DIA 1
## 1291 I718 DIA 1
## 1292 I721 DIA 1
## 1293 I724 DIA 1
## 1294 I728 DIA 1
## 1295 I772 DIA 1
## 1296 I774 DIA 1
## 1297 I790 DIA 1
## 1298 I798 DIA 1
## 1299 I808 DIA 1
## 1300 I81X DIA 1
## 1301 I822 DIA 1
## 1302 I861 DIA 1
## 1303 I870 DIA 1
## 1304 I898 DIA 1
## 1305 I899 DIA 1
## 1306 I950 DIA 1
## 1307 I951 DIA 1
## 1308 I958 B972 1
## 1309 I982 DIA 1
## 1310 I988 DIA 1
## 1311 J020 DIA 1
## 1312 J028 DIA 1
## 1313 J030 DIA 1
## 1314 J041 DIA 1
## 1315 J042 0 1
## 1316 J06 DIA 1
## 1317 J09X DIA 1
## 1318 J111 DIA 1
## 1319 J118 DIA 1
## 1320 J155 D509 1
## 1321 J172 DIA 1
## 1322 J182 DIA 1
## 1323 J20 DIA 1
## 1324 J211 DIA 1
## 1325 J321 DIA 1
## 1326 J329 DIA 1
## 1327 J330 DIA 1
## 1328 J339 DIA 1
## 1329 J359 DIA 1
## 1330 J382 DIA 1
## 1331 J391 DIA 1
## 1332 J410 DIA 1
## 1333 J42 D500 1
## 1334 J458 DIA 1
## 1335 J620 DIA 1
## 1336 J633 DIA 1
## 1337 J638 DIA 1
## 1338 J691 DIA 1
## 1339 J940 DIA 1
## 1340 J951 DIA 1
## 1341 J953 DIA 1
## 1342 J955 B972 1
## 1343 J958 A415 1
## 1344 J982 DIA 1
## 1345 J986 DIA 1
## 1346 J98O DIA 1
## 1347 J998 DIA 1
## 1348 K021 DIA 1
## 1349 K039 B374 1
## 1350 K040 DIA 1
## 1351 K041 DIA 1
## 1352 K051 DIA 1
## 1353 K053 DIA 1
## 1354 K092 DIA 1
## 1355 K109 DIA 1
## 1356 K112 DIA 1
## 1357 K123 A150 1
## 1358 K20 DIA 1
## 1359 K210 DIA 1
## 1360 K220 A150 1
## 1361 K225 DIA 1
## 1362 K226 DIA 1
## 1363 K228 DIA 1
## 1364 K25 D53 1
## 1365 K256 DIA 1
## 1366 k259 DIA 1
## 1367 K261 A418 1
## 1368 K264 DIA 1
## 1369 K292 A419 1
## 1370 K293 DIA 1
## 1371 K299 DIA 1
## 1372 K310 DIA 1
## 1373 K311 D649 1
## 1374 K317 DIA 1
## 1375 K381 DIA 1
## 1376 K383 DIA 1
## 1377 K388 DIA 1
## 1378 K40 C61 1
## 1379 K403 DIA 1
## 1380 K404 DIA 1
## 1381 K440 DIA 1
## 1382 K450 DIA 1
## 1383 K458 A419 1
## 1384 K460 DIA 1
## 1385 K510 DIA 1
## 1386 K519 DIA 1
## 1387 K552 DIA 1
## 1388 K559 DIA 1
## 1389 K563 DIA 1
## 1390 K572 DIA 1
## 1391 K580 DIA 1
## 1392 K599 DIA 1
## 1393 K604 DIA 1
## 1394 K611 DIA 1
## 1395 K620 DIA 1
## 1396 K623 DIA 1
## 1397 K624 DIA 1
## 1398 K628 DIA 1
## 1399 K631 DIA 1
## 1400 K640 DIA 1
## 1401 K641 DIA 1
## 1402 K660 DIA 1
## 1403 K661 DIA 1
## 1404 K70 E11 1
## 1405 K702 DIA 1
## 1406 K710 A150 1
## 1407 K712 DIA 1
## 1408 K718 DIA 1
## 1409 K732 D134 1
## 1410 K753 DIA 1
## 1411 K76 E14 1
## 1412 K761 DIA 1
## 1413 K767 DIA 1
## 1414 K770 D509 1
## 1415 K80 DIA 1
## 1416 K81 A53 1
## 1417 K823 DIA 1
## 1418 K832 DIA 1
## 1419 K833 DIA 1
## 1420 K86 DIA 1
## 1421 K863 DIA 1
## 1422 K913 DIA 1
## 1423 K915 DIA 1
## 1424 K921 DIA 1
## 1425 L00X DIA 1
## 1426 L010 D638 1
## 1427 L03X E11X 1
## 1428 L040 DIA 1
## 1429 L048 DIA 1
## 1430 L08 DIA 1
## 1431 L081 D508 1
## 1432 L102 DIA 1
## 1433 L108 DIA 1
## 1434 L120 DIA 1
## 1435 L129 DIA 1
## 1436 L139 DIA 1
## 1437 L14X DIA 1
## 1438 L208 DIA 1
## 1439 L210 DIA 1
## 1440 L239 A162 1
## 1441 L249 DIA 1
## 1442 L26 A499 1
## 1443 L270 DIA 1
## 1444 L271 DIA 1
## 1445 L300 DIA 1
## 1446 L308 DIA 1
## 1447 L402 DIA 1
## 1448 L414 DIA 1
## 1449 L480 DIA 1
## 1450 L501 DIA 1
## 1451 L512 DIA 1
## 1452 L519 D649 1
## 1453 L538 D649 1
## 1454 L551 DIA 1
## 1455 L602 DIA 1
## 1456 L603 DIA 1
## 1457 L640 DIA 1
## 1458 L702 DIA 1
## 1459 L739 DIA 1
## 1460 L89 DIA 1
## 1461 L903 DIA 1
## 1462 L905 DIA 1
## 1463 L921 DIA 1
## 1464 L929 DIA 1
## 1465 L958 D649 1
## 1466 L959 DIA 1
## 1467 L97 DIA 1
## 1468 L982 DIA 1
## 1469 L989 DIA 1
## 1470 M000 DIA 1
## 1471 M008 DIA 1
## 1472 M018 DIA 1
## 1473 M030 DIA 1
## 1474 M050 DIA 1
## 1475 M053 DIA 1
## 1476 M064 DIA 1
## 1477 M103 DIA 1
## 1478 M112 DIA 1
## 1479 M120 DIA 1
## 1480 M125 DIA 1
## 1481 M138 DIA 1
## 1482 M142 DIA 1
## 1483 M145 DIA 1
## 1484 M160 DIA 1
## 1485 M170 DIA 1
## 1486 M171 DIA 1
## 1487 M189 D509 1
## 1488 M190 DIA 1
## 1489 M191 DIA 1
## 1490 M204 DIA 1
## 1491 M211 DIA 1
## 1492 M215 DIA 1
## 1493 M231 DIA 1
## 1494 M243 DIA 1
## 1495 M245 DIA 1
## 1496 M250 DIA 1
## 1497 M254 DIA 1
## 1498 M255 DIA 1
## 1499 M316 DIA 1
## 1500 M319 DIA 1
## 1501 M321 A419 1
## 1502 M328 DIA 1
## 1503 M331 DIA 1
## 1504 M348 DIA 1
## 1505 M350 DIA 1
## 1506 M354 DIA 1
## 1507 M359 DIA 1
## 1508 M361 DIA 1
## 1509 M429 DIA 1
## 1510 M462 DIA 1
## 1511 M464 DIA 1
## 1512 M465 DIA 1
## 1513 M47 E14 1
## 1514 M470 DIA 1
## 1515 M471 DIA 1
## 1516 M478 DIA 1
## 1517 M488 DIA 1
## 1518 M493 DIA 1
## 1519 M540 D530 1
## 1520 M542 DIA 1
## 1521 M546 DIA 1
## 1522 M620 DIA 1
## 1523 M621 DIA 1
## 1524 M623 DIA 1
## 1525 M624 DIA 1
## 1526 M650 DIA 1
## 1527 M658 DIA 1
## 1528 M659 DIA 1
## 1529 M701 DIA 1
## 1530 M703 DIA 1
## 1531 M706 DIA 1
## 1532 M711 DIA 1
## 1533 M712 DIA 1
## 1534 M719 DIA 1
## 1535 M722 DIA 1
## 1536 M753 DIA 1
## 1537 M754 DIA 1
## 1538 M755 D689 1
## 1539 M762 DIA 1
## 1540 M763 DIA 1
## 1541 M773 DIA 1
## 1542 M795 DIA 1
## 1543 M841 DIA 1
## 1544 M842 DIA 1
## 1545 M844 DIA 1
## 1546 M850 DIA 1
## 1547 M865 DIA 1
## 1548 M870 DIA 1
## 1549 M898 DIA 1
## 1550 M902 DIA 1
## 1551 M922 D500 1
## 1552 M928 DIA 1
## 1553 M960 DIA 1
## 1554 M995 DIA 1
## 1555 N000 DIA 1
## 1556 N002 DIA 1
## 1557 N025 DIA 1
## 1558 N030 DIA 1
## 1559 N038 DIA 1
## 1560 N063 A09X 1
## 1561 N078 DIA 1
## 1562 N10 B972 1
## 1563 N13 E11 1
## 1564 N134 DIA 1
## 1565 N135 DIA 1
## 1566 N136 DIA 1
## 1567 N19 D599 1
## 1568 N201 DIA 1
## 1569 N211 DIA 1
## 1570 N26X D649 1
## 1571 N288 DIA 1
## 1572 N311 DIA 1
## 1573 N342 DIA 1
## 1574 N350 DIA 1
## 1575 N358 DIA 1
## 1576 N360 DIA 1
## 1577 N391 DIA 1
## 1578 N418 DIA 1
## 1579 N46 DIA 1
## 1580 N48 DIA 1
## 1581 N489 DIA 1
## 1582 N509 DIA 1
## 1583 N62X DIA 1
## 1584 N81 B972 1
## 1585 N841 DIA 1
## 1586 N998 DIA 1
## 1587 Q019 DIA 1
## 1588 Q444 DIA 1
## 1589 Q612 C609 1
## 1590 Q785 DIA 1
## 1591 Q810 DIA 1
## 1592 Q811 DIA 1
## 1593 Q850 DIA 1
## 1594 Q871 DIA 1
## 1595 Q874 DIA 1
## 1596 R02 D509 1
## 1597 R031 DIA 1
## 1598 R049 DIA 1
## 1599 R063 DIA 1
## 1600 R068 DIA 1
## 1601 R070 DIA 1
## 1602 R071 DIA 1
## 1603 R098 DIA 1
## 1604 R102 DIA 1
## 1605 R103 DIA 1
## 1606 R17 C249 1
## 1607 R270 DIA 1
## 1608 R278 D649 1
## 1609 R398 DIA 1
## 1610 R400 DIA 1
## 1611 R458 A429 1
## 1612 R470 DIA 1
## 1613 R478 DIA 1
## 1614 R520 DIA 1
## 1615 R54 A415 1
## 1616 R590 DIA 1
## 1617 R634 DIA 1
## 1618 R730 DIA 1
## 1619 R740 DIA 1
## 1620 R799 DIA 1
## 1621 R829 B972 1
## 1622 R931 DIA 1
## 1623 R99X DIA 1
## 1624 S001 DIA 1
## 1625 S014 DIA 1
## 1626 S015 DIA 1
## 1627 S018 DIA 1
## 1628 S026 DIA 1
## 1629 S027 DIA 1
## 1630 S031 DIA 1
## 1631 S045 DIA 1
## 1632 S055 B370 1
## 1633 S056 DIA 1
## 1634 S061 DIA 1
## 1635 S063 DIA 1
## 1636 S068 DIA 1
## 1637 S098 DIA 1
## 1638 S100 DIA 1
## 1639 S119 DIA 1
## 1640 S143 DIA 1
## 1641 S144 DIA 1
## 1642 S159 DIA 1
## 1643 S203 DIA 1
## 1644 S222 B001 1
## 1645 S270 DIA 1
## 1646 S298 DIA 1
## 1647 S300 DIA 1
## 1648 S301 DIA 1
## 1649 S302 DIA 1
## 1650 S308 DIA 1
## 1651 S309 DIA 1
## 1652 S312 DIA 1
## 1653 S315 DIA 1
## 1654 S318 DIA 1
## 1655 S321 DIA 1
## 1656 S325 D648 1
## 1657 S340 DIA 1
## 1658 S341 DIA 1
## 1659 S344 DIA 1
## 1660 S359 D62X 1
## 1661 S370 D649 1
## 1662 S372 DIA 1
## 1663 S373 A419 1
## 1664 S390 DIA 1
## 1665 S398 D62X 1
## 1666 S407 DIA 1
## 1667 S420 DIA 1
## 1668 S421 DIA 1
## 1669 S422 DIA 1
## 1670 S431 DIA 1
## 1671 S489 DIA 1
## 1672 S507 DIA 1
## 1673 S510 D649 1
## 1674 S520 DIA 1
## 1675 S580 DIA 1
## 1676 S589 DIA 1
## 1677 S607 DIA 1
## 1678 S609 DIA 1
## 1679 S623 DIA 1
## 1680 S627 DIA 1
## 1681 S644 DIA 1
## 1682 S668 DIA 1
## 1683 S670 DIA 1
## 1684 S678 DIA 1
## 1685 S684 DIA 1
## 1686 S689 DIA 1
## 1687 S700 DIA 1
## 1688 S708 DIA 1
## 1689 S717 DIA 1
## 1690 S722 DIA 1
## 1691 S730 DIA 1
## 1692 S749 DIA 1
## 1693 S800 DIA 1
## 1694 S801 DIA 1
## 1695 S809 DIA 1
## 1696 S823 DIA 1
## 1697 S832 DIA 1
## 1698 S860 DIA 1
## 1699 S899 DIA 1
## 1700 S900 DIA 1
## 1701 S902 DIA 1
## 1702 S907 DIA 1
## 1703 S908 DIA 1
## 1704 S923 DIA 1
## 1705 S930 DIA 1
## 1706 S934 DIA 1
## 1707 S948 DIA 1
## 1708 S971 DIA 1
## 1709 S978 DIA 1
## 1710 S998 DIA 1
## 1711 T000 DIA 1
## 1712 T013 DIA 1
## 1713 T018 DIA 1
## 1714 T050 DIA 1
## 1715 T058 DIA 1
## 1716 T068 DIA 1
## 1717 T08X B972 1
## 1718 T093 A630 1
## 1719 T099 DIA 1
## 1720 T116 A418 1
## 1721 T135 DIA 1
## 1722 T139 DIA 1
## 1723 T145 DIA 1
## 1724 T148 DIA 1
## 1725 T202 DIA 1
## 1726 T212 DIA 1
## 1727 T222 DIA 1
## 1728 T230 DIA 1
## 1729 T241 DIA 1
## 1730 T250 D649 1
## 1731 T291 DIA 1
## 1732 T292 DIA 1
## 1733 T300 DIA 1
## 1734 T310 DIA 1
## 1735 T348 DIA 1
## 1736 T365 DIA 1
## 1737 T368 DIA 1
## 1738 T409 DIA 1
## 1739 T424 DIA 1
## 1740 T443 DIA 1
## 1741 T451 DIA 1
## 1742 T477 DIA 1
## 1743 T509 DIA 1
## 1744 T528 DIA 1
## 1745 T541 DIA 1
## 1746 T542 DIA 1
## 1747 T543 DIA 1
## 1748 T58X DIA 1
## 1749 T601 DIA 1
## 1750 T604 DIA 1
## 1751 T634 DIA 1
## 1752 T635 DIA 1
## 1753 T639 DIA 1
## 1754 T652 DIA 1
## 1755 T670 DIA 1
## 1756 T674 DIA 1
## 1757 T740 D649 1
## 1758 T741 DIA 1
## 1759 T782 DIA 1
## 1760 T783 DIA 1
## 1761 T784 DIA 1
## 1762 T803 D689 1
## 1763 T808 DIA 1
## 1764 T818 DIA 1
## 1765 T825 DIA 1
## 1766 T848 DIA 1
## 1767 T861 DIA 1
## 1768 T868 DIA 1
## 1769 T869 DIA 1
## 1770 T935 DIA 1
## 1771 T936 DIA 1
## 1772 T983 DIA 1
## 1773 U202 A150 1
## 1774 U205 DIA 1
## 1775 V099 DIA 1
## 1776 V385 DIA 1
## 1777 W010 DIA 1
## 1778 W079 DIA 1
## 1779 W170 DIA 1
## 1780 W204 DIA 1
## 1781 W229 DIA 1
## 1782 X259 DIA 1
## 1783 X689 A059 1
## 1784 X699 DIA 1
## 1785 Y279 DIA 1
## 1786 Y411 A153 1
## 1787 Y832 A419 1
## 1788 Y913 DIA 1
## 1789 Z038 DIA 1
## 1790 Z433 DIA 1
## 1791 Z450 DIA 1
## 1792 Z549 DIA 1
## 1793 Z721 DIA 1
## 1794 Z930 DIA 1
## 1795 Z992 DIA 1

### Male, 18 - 39 years old

## Diag1 Diag2 Frequency
## 1 N390 DIA 178
## 2 U071 DIA 154
## 3 E669 DIA 112
## 4 I10X DIA 108
## 5 J189 DIA 83
## 6 J960 DIA 52
## 7 A150 DIA 49
## 8 L031 DIA 45
## 9 N189 DIA 45
## 10 D649 DIA 39
## 11 B972 DIA 30
## 12 B24X DIA 29
## 13 E86X DIA 26
## 14 A090 DIA 25
## 15 J969 DIA 23
## 16 K859 DIA 21
## 17 N185 DIA 21
## 18 J159 DIA 20
## 19 L039 DIA 20
## 20 E162 DIA 19
## 21 E668 DIA 19
## 22 R739 DIA 17
## 23 J129 DIA 16
## 24 N179 DIA 16
## 25 G409 DIA 14
## 26 J128 DIA 14
## 27 R104 DIA 14
## 28 E872 DIA 13
## 29 L024 DIA 13
## 30 N10X DIA 13
## 31 R568 DIA 13
## 32 F209 DIA 12
## 33 D509 DIA 11
## 34 E660 DIA 10
## 35 E785 DIA 10
## 36 J459 DIA 10
## 37 K295 DIA 10
## 38 M869 DIA 10
## 39 D539 DIA 9
## 40 K358 DIA 9
## 41 N319 DIA 9
## 42 E780 DIA 8
## 43 G934 DIA 8
## 44 I678 DIA 8
## 45 J209 DIA 8
## 46 K291 DIA 8
## 47 K610 DIA 8
## 48 L029 DIA 8
## 49 L038 DIA 8
## 50 N039 DIA 8
## 51 N47X DIA 8
## 52 L030 DIA 7
## 53 N19X DIA 7
## 54 E249 DIA 6
## 55 E46X DIA 6
## 56 E782 DIA 6
## 57 E876 DIA 6
## 58 F102 DIA 6
## 59 J869 DIA 6
## 60 K529 DIA 6
## 61 N481 DIA 6
## 62 S913 DIA 6
## 63 S984 DIA 6
## 64 U072 DIA 6
## 65 A400 DIA 5
## 66 E220 DIA 5
## 67 E889 DIA 5
## 68 H269 DIA 5
## 69 H280 DIA 5
## 70 I64X DIA 5
## 71 J029 DIA 5
## 72 J80X DIA 5
## 73 J852 DIA 5
## 74 J90X DIA 5
## 75 K047 DIA 5
## 76 K297 DIA 5
## 77 K746 DIA 5
## 78 K810 DIA 5
## 79 K811 DIA 5
## 80 L023 DIA 5
## 81 L028 DIA 5
## 82 L409 DIA 5
## 83 L984 DIA 5
## 84 N181 DIA 5
## 85 N200 DIA 5
## 86 R100 DIA 5
## 87 R509 DIA 5
## 88 E835 DIA 4
## 89 F200 DIA 4
## 90 F329 DIA 4
## 91 G590 DIA 4
## 92 G632 DIA 4
## 93 H360 DIA 4
## 94 I209 DIA 4
## 95 I219 DIA 4
## 96 J180 DIA 4
## 97 J188 DIA 4
## 98 J22X DIA 4
## 99 J47X DIA 4
## 100 K703 DIA 4
## 101 K802 DIA 4
## 102 K851 DIA 4
## 103 L020 DIA 4
## 104 L400 DIA 4
## 105 M861 DIA 4
## 106 N049 DIA 4
## 107 N151 DIA 4
## 108 N399 DIA 4
## 109 R042 DIA 4
## 110 T136 DIA 4
## 111 T814 DIA 4
## 112 0 DIA 3
## 113 A480 DIA 3
## 114 B86X DIA 3
## 115 C329 DIA 3
## 116 E059 DIA 3
## 117 E43X DIA 3
## 118 E440 DIA 3
## 119 E871 DIA 3
## 120 E878 DIA 3
## 121 F142 DIA 3
## 122 G410 DIA 3
## 123 G909 DIA 3
## 124 I159 DIA 3
## 125 I500 DIA 3
## 126 I509 DIA 3
## 127 I639 DIA 3
## 128 I829 DIA 3
## 129 J13X DIA 3
## 130 K318 DIA 3
## 131 K591 DIA 3
## 132 K750 DIA 3
## 133 K819 DIA 3
## 134 L021 DIA 3
## 135 L022 DIA 3
## 136 L089 DIA 3
## 137 L401 DIA 3
## 138 M329 DIA 3
## 139 M545 DIA 3
## 140 M725 DIA 3
## 141 N492 DIA 3
## 142 N498 DIA 3
## 143 N512 DIA 3
## 144 R02X DIA 3
## 145 R51X DIA 3
## 146 R571 DIA 3
## 147 R572 DIA 3
## 148 S069 DIA 3
## 149 S822 DIA 3
## 150 T009 DIA 3
## 151 T888 A150 3
## 152 43558 DIA 2
## 153 43577 DIA 2
## 154 A852 DIA 2
## 155 B353 DIA 2
## 156 E050 DIA 2
## 157 E789 DIA 2
## 158 E832 DIA 2
## 159 F411 DIA 2
## 160 F419 DIA 2
## 161 F429 DIA 2
## 162 F432 DIA 2
## 163 F721 DIA 2
## 164 G408 DIA 2
## 165 G589 DIA 2
## 166 G629 DIA 2
## 167 H650 DIA 2
## 168 I10 DIA 2
## 169 I120 DIA 2
## 170 I200 DIA 2
## 171 I241 A150 2
## 172 I269 DIA 2
## 173 I610 DIA 2
## 174 I802 DIA 2
## 175 I872 DIA 2
## 176 J120 DIA 2
## 177 J150 DIA 2
## 178 J168 DIA 2
## 179 J181 DIA 2
## 180 J342 DIA 2
## 181 J40X DIA 2
## 182 J841 DIA 2
## 183 J851 DIA 2
## 184 J860 DIA 2
## 185 J931 DIA 2
## 186 J938 DIA 2
## 187 J948 DIA 2
## 188 J961 DIA 2
## 189 K30X DIA 2
## 190 K352 DIA 2
## 191 K402 DIA 2
## 192 K409 DIA 2
## 193 K469 DIA 2
## 194 K564 DIA 2
## 195 K565 DIA 2
## 196 K566 DIA 2
## 197 K603 DIA 2
## 198 K709 DIA 2
## 199 K719 DIA 2
## 200 K829 DIA 2
## 201 K830 DIA 2
## 202 K85 DIA 2
## 203 K858 DIA 2
## 204 K869 K850 2
## 205 K929 DIA 2
## 206 L033 DIA 2
## 207 L405 DIA 2
## 208 L500 DIA 2
## 209 L891 DIA 2
## 210 M073 DIA 2
## 211 M109 DIA 2
## 212 M139 DIA 2
## 213 M179 DIA 2
## 214 M490 DIA 2
## 215 M720 DIA 2
## 216 M729 DIA 2
## 217 N110 DIA 2
## 218 N111 DIA 2
## 219 N12X DIA 2
## 220 N182 DIA 2
## 221 N40X DIA 2
## 222 N411 DIA 2
## 223 N44X DIA 2
## 224 Q909 DIA 2
## 225 R101 DIA 2
## 226 R11X DIA 2
## 227 R18X DIA 2
## 228 R33X DIA 2
## 229 R36X DIA 2
## 230 R578 DIA 2
## 231 R609 DIA 2
## 232 R770 DIA 2
## 233 S051 DIA 2
## 234 S062 DIA 2
## 235 S311 DIA 2
## 236 S328 DIA 2
## 237 S399 DIA 2
## 238 S681 DIA 2
## 239 S820 DIA 2
## 240 S982 DIA 2
## 241 T600 DIA 2
## 242 T874 DIA 2
## 243 Y919 DIA 2
## 244 A190 A159 1
## 245 A540 A150 1
## 246 A90X A150 1
## 247 B200 A150 1
## 248 B207 A090 1
## 249 B24 A154 1
## 250 B450 B24X 1
## 251 B451 B009 1
## 252 B829 A090 1
## 253 D489 D352 1
## 254 D538 A153 1
## 255 D686 D510 1
## 256 D688 A419 1
## 257 D693 C920 1
## 258 E031 DIA 1
## 259 E10X E039 1
## 260 E160 DIA 1
## 261 E161 DIA 1
## 262 E169 A090 1
## 263 E230 D441 1
## 264 E270 DIA 1
## 265 E271 DIA 1
## 266 E273 DIA 1
## 267 E40X DIA 1
## 268 E441 DIA 1
## 269 E538 D509 1
## 270 E709 DIA 1
## 271 E750 DIA 1
## 272 E784 DIA 1
## 273 E788 DIA 1
## 274 E804 DIA 1
## 275 E870 B465 1
## 276 E874 DIA 1
## 277 E880 DIA 1
## 278 F062 DIA 1
## 279 F069 DIA 1
## 280 F106 DIA 1
## 281 F172 DIA 1
## 282 F192 DIA 1
## 283 F201 D649 1
## 284 F321 DIA 1
## 285 F412 DIA 1
## 286 F602 DIA 1
## 287 F609 DIA 1
## 288 F669 DIA 1
## 289 F709 DIA 1
## 290 F719 DIA 1
## 291 F729 DIA 1
## 292 F790 DIA 1
## 293 F900 DIA 1
## 294 G042 DIA 1
## 295 G129 DIA 1
## 296 G255 DIA 1
## 297 G401 DIA 1
## 298 G403 DIA 1
## 299 G419 DIA 1
## 300 G510 DIA 1
## 301 G611 DIA 1
## 302 G619 DIA 1
## 303 G825 A419 1
## 304 G911 DIA 1
## 305 G932 C793 1
## 306 G948 DIA 1
## 307 H050 DIA 1
## 308 H193 DIA 1
## 309 H262 DIA 1
## 310 H268 DIA 1
## 311 H409 DIA 1
## 312 H521 DIA 1
## 313 H523 DIA 1
## 314 H530 DIA 1
## 315 H542 DIA 1
## 316 H609 DIA 1
## 317 H651 DIA 1
## 318 H669 DIA 1
## 319 H678 DIA 1
## 320 H811 DIA 1
## 321 H813 DIA 1
## 322 H919 DIA 1
## 323 I110 DIA 1
## 324 I151 DIA 1
## 325 I255 DIA 1
## 326 I259 DIA 1
## 327 I278 A160 1
## 328 I460 A419 1
## 329 I469 DIA 1
## 330 I471 DIA 1
## 331 I479 DIA 1
## 332 I48X DIA 1
## 333 I609 DIA 1
## 334 I611 DIA 1
## 335 I620 DIA 1
## 336 I694 DIA 1
## 337 I698 DIA 1
## 338 I739 DIA 1
## 339 I742 DIA 1
## 340 I748 B671 1
## 341 I770 DIA 1
## 342 I771 DIA 1
## 343 I792 DIA 1
## 344 I803 DIA 1
## 345 I828 DIA 1
## 346 I830 DIA 1
## 347 I832 DIA 1
## 348 I842 DIA 1
## 349 I959 DIA 1
## 350 J014 DIA 1
## 351 J028 DIA 1
## 352 J039 DIA 1
## 353 J06 DIA 1
## 354 J068 DIA 1
## 355 J151 DIA 1
## 356 J156 DIA 1
## 357 J157 DIA 1
## 358 J340 DIA 1
## 359 J448 DIA 1
## 360 J449 DIA 1
## 361 J81X DIA 1
## 362 J849 DIA 1
## 363 J850 DIA 1
## 364 J930 A150 1
## 365 J980 DIA 1
## 366 J981 DIA 1
## 367 J984 DIA 1
## 368 K041 DIA 1
## 369 K051 DIA 1
## 370 K092 DIA 1
## 371 K102 DIA 1
## 372 K113 DIA 1
## 373 K122 DIA 1
## 374 K222 DIA 1
## 375 K270 DIA 1
## 376 K273 DIA 1
## 377 K290 DIA 1
## 378 K293 DIA 1
## 379 K296 DIA 1
## 380 K299 DIA 1
## 381 K37X DIA 1
## 382 K381 DIA 1
## 383 K429 DIA 1
## 384 K560 DIA 1
## 385 K590 DIA 1
## 386 K599 DIA 1
## 387 K612 DIA 1
## 388 K626 DIA 1
## 389 K632 DIA 1
## 390 K648 DIA 1
## 391 K658 DIA 1
## 392 K700 DIA 1
## 393 K704 DIA 1
## 394 K729 DIA 1
## 395 K740 DIA 1
## 396 K752 A090 1
## 397 K759 B018 1
## 398 K760 DIA 1
## 399 K769 DIA 1
## 400 K800 DIA 1
## 401 K801 DIA 1
## 402 K805 DIA 1
## 403 K839 DIA 1
## 404 K85X DIA 1
## 405 K861 DIA 1
## 406 K862 DIA 1
## 407 K863 DIA 1
## 408 L032 DIA 1
## 409 L043 DIA 1
## 410 L088 DIA 1
## 411 L210 DIA 1
## 412 L239 A162 1
## 413 L300 DIA 1
## 414 L551 DIA 1
## 415 L603 DIA 1
## 416 L719 DIA 1
## 417 L892 DIA 1
## 418 L899 DIA 1
## 419 L989 DIA 1
## 420 M000 DIA 1
## 421 M009 DIA 1
## 422 M069 DIA 1
## 423 M100 DIA 1
## 424 M145 DIA 1
## 425 M169 DIA 1
## 426 M199 DIA 1
## 427 M215 DIA 1
## 428 M321 A419 1
## 429 M350 DIA 1
## 430 M489 DIA 1
## 431 M511 DIA 1
## 432 M541 DIA 1
## 433 M609 DIA 1
## 434 M659 DIA 1
## 435 M726 DIA 1
## 436 M763 DIA 1
## 437 M809 DIA 1
## 438 M841 DIA 1
## 439 M866 DIA 1
## 440 M868 DIA 1
## 441 M932 DIA 1
## 442 M939 DIA 1
## 443 N009 DIA 1
## 444 N025 DIA 1
## 445 N029 DIA 1
## 446 N059 DIA 1
## 447 N083 DIA 1
## 448 N119 DIA 1
## 449 N133 C64X 1
## 450 N134 DIA 1
## 451 N136 DIA 1
## 452 N159 A419 1
## 453 N170 D539 1
## 454 N172 DIA 1
## 455 N178 DIA 1
## 456 N180 DIA 1
## 457 N18X E039 1
## 458 N220 DIA 1
## 459 N23X A099 1
## 460 N251 DIA 1
## 461 N258 A099 1
## 462 N310 DIA 1
## 463 N341 DIA 1
## 464 N398 DIA 1
## 465 N434 DIA 1
## 466 N459 DIA 1
## 467 N499 DIA 1
## 468 Q610 A400 1
## 469 Q785 DIA 1
## 470 Q811 DIA 1
## 471 Q871 DIA 1
## 472 R072 DIA 1
## 473 R098 DIA 1
## 474 R160 A150 1
## 475 R17X DIA 1
## 476 R392 DIA 1
## 477 R458 A429 1
## 478 R54X DIA 1
## 479 R560 DIA 1
## 480 R570 DIA 1
## 481 R64X DIA 1
## 482 R798 DIA 1
## 483 R799 DIA 1
## 484 R960 DIA 1
## 485 S010 DIA 1
## 486 S045 DIA 1
## 487 S063 DIA 1
## 488 S099 DIA 1
## 489 S109 DIA 1
## 490 S271 DIA 1
## 491 S272 DIA 1
## 492 S344 DIA 1
## 493 S359 D62X 1
## 494 S369 DIA 1
## 495 S398 D62X 1
## 496 S431 DIA 1
## 497 S520 DIA 1
## 498 S610 DIA 1
## 499 S717 DIA 1
## 500 S720 DIA 1
## 501 S729 DIA 1
## 502 S817 DIA 1
## 503 S819 DIA 1
## 504 S821 DIA 1
## 505 S824 DIA 1
## 506 S917 DIA 1
## 507 S930 DIA 1
## 508 S981 DIA 1
## 509 T000 DIA 1
## 510 T019 DIA 1
## 511 T054 DIA 1
## 512 T059 DIA 1
## 513 T093 A630 1
## 514 T141 DIA 1
## 515 T477 DIA 1
## 516 T604 DIA 1
## 517 T633 DIA 1
## 518 T782 DIA 1
## 519 T783 DIA 1
## 520 T793 DIA 1
## 521 T808 DIA 1
## 522 T856 A499 1
## 523 T869 DIA 1
## 524 T889 DIA 1
## 525 T930 DIA 1
## 526 U202 A150 1
## 527 X689 A059 1

### Male, 40 - 59 years old

## Diag1 Diag2 Frequency
## 1 I10X DIA 1469
## 2 U071 DIA 754
## 3 N390 DIA 700
## 4 N189 DIA 587
## 5 J189 DIA 519
## 6 J960 DIA 365
## 7 L031 DIA 346
## 8 D649 DIA 263
## 9 A419 DIA 228
## 10 N185 DIA 167
## 11 J969 DIA 159
## 12 E669 DIA 158
## 13 D509 DIA 110
## 14 I500 DIA 107
## 15 A150 DIA 100
## 16 J129 DIA 97
## 17 L039 DIA 96
## 18 N40X DIA 94
## 19 K922 DIA 89
## 20 R739 DIA 81
## 21 E162 DIA 76
## 22 I639 DIA 75
## 23 S913 DIA 74
## 24 A090 DIA 68
## 25 J128 DIA 67
## 26 K746 DIA 67
## 27 N039 DIA 66
## 28 J159 DIA 63
## 29 M869 DIA 61
## 30 L030 DIA 58
## 31 L97X DIA 53
## 32 I509 DIA 52
## 33 I678 DIA 52
## 34 E86X DIA 49
## 35 K859 DIA 48
## 36 L029 DIA 47
## 37 N10X DIA 47
## 38 K610 DIA 45
## 39 U072 DIA 45
## 40 K703 DIA 43
## 41 L024 DIA 43
## 42 N179 DIA 42
## 43 R02X DIA 42
## 44 I739 DIA 41
## 45 S984 DIA 39
## 46 J90X DIA 38
## 47 L023 DIA 38
## 48 G409 DIA 37
## 49 I679 DIA 37
## 50 A169 DIA 35
## 51 N19X DIA 35
## 52 R104 DIA 35
## 53 T136 DIA 35
## 54 J47X DIA 34
## 55 H360 DIA 33
## 56 L038 DIA 33
## 57 K810 DIA 32
## 58 L032 DIA 32
## 59 R568 DIA 32
## 60 G934 DIA 31
## 61 I619 DIA 31
## 62 J80X DIA 30
## 63 N498 DIA 30
## 64 A418 DIA 28
## 65 E039 DIA 28
## 66 F200 DIA 28
## 67 K358 DIA 28
## 68 N180 DIA 28
## 69 R509 DIA 28
## 70 S981 DIA 27
## 71 I159 DIA 26
## 72 J459 DIA 26
## 73 I119 DIA 25
## 74 K850 DIA 25
## 75 R042 DIA 25
## 76 E872 DIA 24
## 77 I259 DIA 24
## 78 K802 DIA 24
## 79 L089 DIA 24
## 80 N200 DIA 24
## 81 D500 DIA 23
## 82 E660 DIA 23
## 83 J852 DIA 23
## 84 R11X DIA 23
## 85 E668 DIA 22
## 86 K85X DIA 22
## 87 L409 DIA 22
## 88 J869 DIA 21
## 89 D539 DIA 20
## 90 I200 DIA 20
## 91 J841 DIA 20
## 92 N049 DIA 20
## 93 L984 DIA 19
## 94 E782 DIA 18
## 95 L022 DIA 18
## 96 S819 DIA 18
## 97 E46X DIA 17
## 98 E780 DIA 17
## 99 I872 DIA 17
## 100 K429 DIA 17
## 101 K529 DIA 17
## 102 K566 DIA 17
## 103 K811 DIA 17
## 104 N110 DIA 17
## 105 N181 DIA 17
## 106 R572 DIA 17
## 107 G459 DIA 16
## 108 I489 DIA 16
## 109 I802 DIA 16
## 110 I829 DIA 16
## 111 J690 DIA 16
## 112 K750 DIA 16
## 113 L021 DIA 16
## 114 L899 DIA 16
## 115 M109 DIA 16
## 116 N12X DIA 16
## 117 N183 DIA 16
## 118 A153 DIA 15
## 119 F102 DIA 15
## 120 I694 DIA 15
## 121 I859 DIA 15
## 122 D648 DIA 14
## 123 E785 DIA 14
## 124 J22X DIA 14
## 125 J81X DIA 14
## 126 J961 DIA 14
## 127 K801 DIA 14
## 128 M725 DIA 14
## 129 N151 DIA 14
## 130 F209 DIA 13
## 131 I633 DIA 13
## 132 I792 DIA 13
## 133 J157 DIA 13
## 134 J849 DIA 13
## 135 L020 DIA 13
## 136 N492 DIA 13
## 137 G590 DIA 12
## 138 G629 DIA 12
## 139 I255 DIA 12
## 140 I480 DIA 12
## 141 I610 DIA 12
## 142 J120 DIA 12
## 143 K805 DIA 12
## 144 N184 DIA 12
## 145 N459 DIA 12
## 146 R51X DIA 12
## 147 S069 DIA 12
## 148 G632 DIA 11
## 149 J029 DIA 11
## 150 K122 DIA 11
## 151 K409 DIA 11
## 152 K590 DIA 11
## 153 R33X DIA 11
## 154 S911 DIA 11
## 155 A09X DIA 10
## 156 C169 DIA 10
## 157 E789 DIA 10
## 158 I110 DIA 10
## 159 I209 DIA 10
## 160 J180 DIA 10
## 161 J449 DIA 10
## 162 K259 DIA 10
## 163 K800 DIA 10
## 164 L028 DIA 10
## 165 M009 DIA 10
## 166 S982 DIA 10
## 167 T009 DIA 10
## 168 A409 DIA 9
## 169 E43X DIA 9
## 170 E889 DIA 9
## 171 F329 DIA 9
## 172 F419 DIA 9
## 173 I120 DIA 9
## 174 I340 DIA 9
## 175 I609 DIA 9
## 176 I832 DIA 9
## 177 J150 DIA 9
## 178 J188 DIA 9
## 179 K603 DIA 9
## 180 L80X DIA 9
## 181 L890 DIA 9
## 182 M726 DIA 9
## 183 N289 DIA 9
## 184 N450 DIA 9
## 185 N499 DIA 9
## 186 S681 DIA 9
## 187 S912 DIA 9
## 188 T141 DIA 9
## 189 E441 DIA 8
## 190 E871 DIA 8
## 191 G610 DIA 8
## 192 H269 DIA 8
## 193 I210 DIA 8
## 194 I48X DIA 8
## 195 I499 DIA 8
## 196 J068 DIA 8
## 197 J984 DIA 8
## 198 K047 DIA 8
## 199 K291 DIA 8
## 200 K295 DIA 8
## 201 K769 DIA 8
## 202 N399 DIA 8
## 203 R571 DIA 8
## 204 S065 DIA 8
## 205 S822 DIA 8
## 206 S881 DIA 8
## 207 T252 DIA 8
## 208 T793 DIA 8
## 209 B378 DIA 7
## 210 D609 DIA 7
## 211 E059 DIA 7
## 212 E160 DIA 7
## 213 E878 DIA 7
## 214 E880 DIA 7
## 215 F100 DIA 7
## 216 G569 DIA 7
## 217 H819 DIA 7
## 218 I151 DIA 7
## 219 I251 DIA 7
## 220 I469 DIA 7
## 221 I612 DIA 7
## 222 I630 DIA 7
## 223 I702 DIA 7
## 224 J168 DIA 7
## 225 K37X DIA 7
## 226 K591 DIA 7
## 227 K830 DIA 7
## 228 M069 DIA 7
## 229 N111 DIA 7
## 230 N139 DIA 7
## 231 N182 DIA 7
## 232 N319 DIA 7
## 233 R001 DIA 7
## 234 R18X DIA 7
## 235 S223 DIA 7
## 236 S980 DIA 7
## 237 T876 DIA 7
## 238 A971 DIA 6
## 239 C220 DIA 6
## 240 C259 DIA 6
## 241 E161 DIA 6
## 242 E271 DIA 6
## 243 E781 DIA 6
## 244 E870 DIA 6
## 245 E876 DIA 6
## 246 G049 DIA 6
## 247 H540 DIA 6
## 248 I249 DIA 6
## 249 I258 DIA 6
## 250 I519 DIA 6
## 251 I618 DIA 6
## 252 I629 DIA 6
## 253 I693 DIA 6
## 254 I743 DIA 6
## 255 I771 DIA 6
## 256 I959 DIA 6
## 257 J181 DIA 6
## 258 J851 DIA 6
## 259 J860 DIA 6
## 260 K046 DIA 6
## 261 K279 DIA 6
## 262 K30X DIA 6
## 263 K353 DIA 6
## 264 K564 DIA 6
## 265 K650 DIA 6
## 266 L080 DIA 6
## 267 L088 DIA 6
## 268 L400 DIA 6
## 269 N119 DIA 6
## 270 R17X DIA 6
## 271 R609 DIA 6
## 272 T131 DIA 6
## 273 T814 DIA 6
## 274 T874 DIA 6
## 275 B370 DIA 5
## 276 B829 DIA 5
## 277 E249 DIA 5
## 278 E440 DIA 5
## 279 F412 DIA 5
## 280 G042 DIA 5
## 281 G510 DIA 5
## 282 G819 DIA 5
## 283 H280 DIA 5
## 284 H659 DIA 5
## 285 I213 DIA 5
## 286 I269 DIA 5
## 287 I490 DIA 5
## 288 I632 DIA 5
## 289 I850 DIA 5
## 290 J13X DIA 5
## 291 J448 DIA 5
## 292 J850 DIA 5
## 293 J939 DIA 5
## 294 J981 DIA 5
## 295 K439 DIA 5
## 296 K605 DIA 5
## 297 K612 DIA 5
## 298 K709 DIA 5
## 299 K721 DIA 5
## 300 K808 DIA 5
## 301 K819 DIA 5
## 302 M609 DIA 5
## 303 N083 DIA 5
## 304 N23X DIA 5
## 305 N309 DIA 5
## 306 N433 DIA 5
## 307 R101 DIA 5
## 308 S619 DIA 5
## 309 S682 DIA 5
## 310 S810 DIA 5
## 311 S818 DIA 5
## 312 S920 DIA 5
## 313 S983 DIA 5
## 314 T887 DIA 5
## 315 B353 DIA 4
## 316 E272 DIA 4
## 317 E835 DIA 4
## 318 E875 DIA 4
## 319 G039 DIA 4
## 320 G060 DIA 4
## 321 G20X DIA 4
## 322 G589 DIA 4
## 323 G628 DIA 4
## 324 G919 DIA 4
## 325 G932 DIA 4
## 326 H350 DIA 4
## 327 I211 DIA 4
## 328 I620 DIA 4
## 329 I634 DIA 4
## 330 I688 DIA 4
## 331 I778 DIA 4
## 332 J100 DIA 4
## 333 J152 DIA 4
## 334 J158 DIA 4
## 335 J698 DIA 4
## 336 J91X DIA 4
## 337 J980 DIA 4
## 338 K102 DIA 4
## 339 K273 DIA 4
## 340 K290 DIA 4
## 341 K351 DIA 4
## 342 K630 DIA 4
## 343 K632 DIA 4
## 344 K759 DIA 4
## 345 K760 DIA 4
## 346 K839 DIA 4
## 347 L033 DIA 4
## 348 L309 DIA 4
## 349 L89X DIA 4
## 350 M100 DIA 4
## 351 M139 DIA 4
## 352 M544 DIA 4
## 353 N040 DIA 4
## 354 N170 DIA 4
## 355 N209 DIA 4
## 356 N411 DIA 4
## 357 N47X DIA 4
## 358 R072 DIA 4
## 359 R418 DIA 4
## 360 R53X DIA 4
## 361 R570 DIA 4
## 362 S328 DIA 4
## 363 S610 DIA 4
## 364 S720 DIA 4
## 365 S723 DIA 4
## 366 S781 DIA 4
## 367 T059 DIA 4
## 368 T140 DIA 4
## 369 T600 DIA 4
## 370 43556 DIA 3
## 371 B351 DIA 3
## 372 E169 DIA 3
## 373 E220 DIA 3
## 374 E756 DIA 3
## 375 F103 DIA 3
## 376 F321 DIA 3
## 377 F449 DIA 3
## 378 G08X DIA 3
## 379 G09X DIA 3
## 380 G400 DIA 3
## 381 G401 DIA 3
## 382 G402 DIA 3
## 383 G403 DIA 3
## 384 G442 DIA 3
## 385 G619 DIA 3
## 386 G822 DIA 3
## 387 G939 DIA 3
## 388 H050 DIA 3
## 389 H46X DIA 3
## 390 H660 DIA 3
## 391 H82X DIA 3
## 392 I150 DIA 3
## 393 I229 DIA 3
## 394 I250 DIA 3
## 395 I420 DIA 3
## 396 I442 DIA 3
## 397 I613 DIA 3
## 398 I635 DIA 3
## 399 I674 DIA 3
## 400 I698 DIA 3
## 401 I801 DIA 3
## 402 I828 DIA 3
## 403 I891 DIA 3
## 404 J069 DIA 3
## 405 J156 DIA 3
## 406 J450 DIA 3
## 407 J46X DIA 3
## 408 K029 DIA 3
## 409 K219 DIA 3
## 410 K250 DIA 3
## 411 K257 DIA 3
## 412 K296 DIA 3
## 413 K352 DIA 3
## 414 K579 DIA 3
## 415 K602 DIA 3
## 416 K745 DIA 3
## 417 K828 DIA 3
## 418 K868 DIA 3
## 419 K928 DIA 3
## 420 L100 DIA 3
## 421 L408 DIA 3
## 422 L509 DIA 3
## 423 L88X DIA 3
## 424 M199 DIA 3
## 425 M705 DIA 3
## 426 M793 DIA 3
## 427 M809 DIA 3
## 428 M868 DIA 3
## 429 M879 DIA 3
## 430 M939 DIA 3
## 431 N133 DIA 3
## 432 N172 DIA 3
## 433 N188 DIA 3
## 434 N220 DIA 3
## 435 N310 DIA 3
## 436 N481 DIA 3
## 437 R000 DIA 3
## 438 R31X DIA 3
## 439 R560 DIA 3
## 440 R579 DIA 3
## 441 R600 DIA 3
## 442 R601 DIA 3
## 443 R64X DIA 3
## 444 R770 DIA 3
## 445 S008 DIA 3
## 446 S010 DIA 3
## 447 S062 DIA 3
## 448 S683 DIA 3
## 449 S718 DIA 3
## 450 S789 DIA 3
## 451 S917 DIA 3
## 452 S925 DIA 3
## 453 S929 DIA 3
## 454 T019 DIA 3
## 455 T242 DIA 3
## 456 T253 DIA 3
## 457 T303 DIA 3
## 458 T519 DIA 3
## 459 T633 DIA 3
## 460 T888 DIA 3
## 461 Z608 DIA 3
## 462 43525 S889 2
## 463 43530 I10X 2
## 464 43547 DIA 2
## 465 43552 DIA 2
## 466 43565 I10X 2
## 467 B009 DIA 2
## 468 C840 DIA 2
## 469 E041 DIA 2
## 470 E050 DIA 2
## 471 E10 DIA 2
## 472 E230 DIA 2
## 473 E240 DIA 2
## 474 E713 DIA 2
## 475 E722 DIA 2
## 476 E739 DIA 2
## 477 E888 DIA 2
## 478 F104 DIA 2
## 479 F172 DIA 2
## 480 F192 DIA 2
## 481 F238 DIA 2
## 482 F250 DIA 2
## 483 F29X DIA 2
## 484 F328 DIA 2
## 485 F339 DIA 2
## 486 F410 DIA 2
## 487 F411 DIA 2
## 488 F413 DIA 2
## 489 F445 DIA 2
## 490 G01X DIA 2
## 491 G040 DIA 2
## 492 G041 DIA 2
## 493 G048 DIA 2
## 494 G050 DIA 2
## 495 G062 DIA 2
## 496 G10X DIA 2
## 497 G131 DIA 2
## 498 G373 DIA 2
## 499 G408 DIA 2
## 500 G458 DIA 2
## 501 G509 DIA 2
## 502 G531 DIA 2
## 503 G579 DIA 2
## 504 G808 DIA 2
## 505 G909 DIA 2
## 506 G969 DIA 2
## 507 G990 DIA 2
## 508 H358 DIA 2
## 509 H409 DIA 2
## 510 H653 DIA 2
## 511 H669 DIA 2
## 512 H730 DIA 2
## 513 I051 DIA 2
## 514 I060 DIA 2
## 515 I158 DIA 2
## 516 I212 DIA 2
## 517 I214 DIA 2
## 518 I248 DIA 2
## 519 I252 DIA 2
## 520 I48 DIA 2
## 521 I495 DIA 2
## 522 I516 DIA 2
## 523 I616 DIA 2
## 524 I631 DIA 2
## 525 I64 DIA 2
## 526 I700 DIA 2
## 527 I729 DIA 2
## 528 I741 DIA 2
## 529 I770 DIA 2
## 530 I775 DIA 2
## 531 I800 DIA 2
## 532 I809 DIA 2
## 533 J00X DIA 2
## 534 J019 DIA 2
## 535 J040 DIA 2
## 536 J123 DIA 2
## 537 J151 DIA 2
## 538 J219 DIA 2
## 539 J36X DIA 2
## 540 J40X DIA 2
## 541 J440 DIA 2
## 542 J441 DIA 2
## 543 J680 DIA 2
## 544 J930 DIA 2
## 545 J942 DIA 2
## 546 K044 DIA 2
## 547 K052 DIA 2
## 548 K20X DIA 2
## 549 K221 DIA 2
## 550 K269 DIA 2
## 551 K319 DIA 2
## 552 K36X DIA 2
## 553 K420 DIA 2
## 554 K522 DIA 2
## 555 K648 DIA 2
## 556 K658 DIA 2
## 557 K717 DIA 2
## 558 K719 DIA 2
## 559 K743 DIA 2
## 560 K758 DIA 2
## 561 K831 DIA 2
## 562 K834 DIA 2
## 563 K838 DIA 2
## 564 K851 DIA 2
## 565 K858 DIA 2
## 566 K860 DIA 2
## 567 K900 DIA 2
## 568 K920 DIA 2
## 569 K929 DIA 2
## 570 L219 DIA 2
## 571 L259 DIA 2
## 572 L405 DIA 2
## 573 L500 DIA 2
## 574 L729 DIA 2
## 575 L892 DIA 2
## 576 L893 DIA 2
## 577 L988 DIA 2
## 578 M029 DIA 2
## 579 M130 DIA 2
## 580 M131 DIA 2
## 581 M148 DIA 2
## 582 M169 DIA 2
## 583 M329 DIA 2
## 584 M360 DIA 2
## 585 M45X DIA 2
## 586 M480 DIA 2
## 587 M494 DIA 2
## 588 M512 DIA 2
## 589 M549 DIA 2
## 590 M600 DIA 2
## 591 M715 DIA 2
## 592 M723 DIA 2
## 593 M728 DIA 2
## 594 M729 DIA 2
## 595 M860 DIA 2
## 596 M861 DIA 2
## 597 M866 DIA 2
## 598 M899 DIA 2
## 599 M952 DIA 2
## 600 N009 DIA 2
## 601 N042 DIA 2
## 602 N071 DIA 2
## 603 N130 DIA 2
## 604 N138 DIA 2
## 605 N178 DIA 2
## 606 N202 DIA 2
## 607 N281 DIA 2
## 608 N312 I872 2
## 609 N328 DIA 2
## 610 N340 DIA 2
## 611 N512 DIA 2
## 612 Q283 DIA 2
## 613 Q667 DIA 2
## 614 R074 DIA 2
## 615 R13X DIA 2
## 616 R190 DIA 2
## 617 R55X DIA 2
## 618 R578 DIA 2
## 619 R58X DIA 2
## 620 R688 DIA 2
## 621 R69X DIA 2
## 622 S019 DIA 2
## 623 S024 DIA 2
## 624 S066 DIA 2
## 625 S279 DIA 2
## 626 S320 DIA 2
## 627 S369 DIA 2
## 628 S399 DIA 2
## 629 S522 DIA 2
## 630 S525 DIA 2
## 631 S611 DIA 2
## 632 S626 DIA 2
## 633 S699 DIA 2
## 634 S701 DIA 2
## 635 S711 DIA 2
## 636 S721 DIA 2
## 637 S824 DIA 2
## 638 S825 DIA 2
## 639 S829 DIA 2
## 640 S910 DIA 2
## 641 S921 DIA 2
## 642 T07X DIA 2
## 643 T130 DIA 2
## 644 T149 DIA 2
## 645 T246 DIA 2
## 646 T630 DIA 2
## 647 T659 DIA 2
## 648 T702 DIA 2
## 649 T813 DIA 2
## 650 T824 DIA 2
## 651 T829 DIA 2
## 652 T857 DIA 2
## 653 T875 DIA 2
## 654 T886 DIA 2
## 655 W179 DIA 2
## 656 Y579 DIA 2
## 657 Y912 DIA 2
## 658 Z519 DIA 2
## 659 A181 A150 1
## 660 A513 A162 1
## 661 B358 B24X 1
## 662 B779 A071 1
## 663 C770 C341 1
## 664 C786 C250 1
## 665 C787 C629 1
## 666 C819 C349 1
## 667 D136 B24X 1
## 668 D382 A165 1
## 669 D65X A150 1
## 670 D695 A278 1
## 671 D728 C602 1
## 672 E038 DIA 1
## 673 E063 DIA 1
## 674 E10X D509 1
## 675 E210 DIA 1
## 676 E236 DIA 1
## 677 E248 D694 1
## 678 E274 DIA 1
## 679 E449 DIA 1
## 680 E538 DIA 1
## 681 E539 DIA 1
## 682 E559 DIA 1
## 683 E60X DIA 1
## 684 E639 DIA 1
## 685 E738 D509 1
## 686 E784 DIA 1
## 687 E831 DIA 1
## 688 E873 A419 1
## 689 E882 DIA 1
## 690 F050 DIA 1
## 691 F058 DIA 1
## 692 F059 DIA 1
## 693 F067 DIA 1
## 694 F079 DIA 1
## 695 F09X B24X 1
## 696 F101 DIA 1
## 697 F106 DIA 1
## 698 F109 DIA 1
## 699 F116 DIA 1
## 700 F130 A162 1
## 701 F135 D509 1
## 702 F230 DIA 1
## 703 F232 DIA 1
## 704 F319 DIA 1
## 705 F320 DIA 1
## 706 F322 D649 1
## 707 F323 DIA 1
## 708 F432 B972 1
## 709 F440 DIA 1
## 710 F444 DIA 1
## 711 F609 D649 1
## 712 F61X DIA 1
## 713 F799 DIA 1
## 714 F808 DIA 1
## 715 F82X DIA 1
## 716 F844 DIA 1
## 717 F909 DIA 1
## 718 F919 DIA 1
## 719 G009 DIA 1
## 720 G020 B24X 1
## 721 G038 DIA 1
## 722 G051 DIA 1
## 723 G061 DIA 1
## 724 G22X DIA 1
## 725 G249 DIA 1
## 726 G250 DIA 1
## 727 G255 DIA 1
## 728 G35X DIA 1
## 729 G368 DIA 1
## 730 G404 DIA 1
## 731 G410 DIA 1
## 732 G419 DIA 1
## 733 G439 DIA 1
## 734 G440 DIA 1
## 735 G444 DIA 1
## 736 G448 DIA 1
## 737 G45 DIA 1
## 738 G452 DIA 1
## 739 G462 DIA 1
## 740 G464 DIA 1
## 741 G465 D180 1
## 742 G530 DIA 1
## 743 G578 DIA 1
## 744 G609 DIA 1
## 745 G611 DIA 1
## 746 G700 DIA 1
## 747 G820 DIA 1
## 748 G930 DIA 1
## 749 G931 DIA 1
## 750 G936 DIA 1
## 751 G938 DIA 1
## 752 G951 DIA 1
## 753 G959 A514 1
## 754 G98X DIA 1
## 755 H031 B023 1
## 756 H043 DIA 1
## 757 H059 DIA 1
## 758 H105 DIA 1
## 759 H110 DIA 1
## 760 H151 DIA 1
## 761 H209 DIA 1
## 762 H220 A514 1
## 763 H250 DIA 1
## 764 H309 DIA 1
## 765 H359 DIA 1
## 766 H368 DIA 1
## 767 H449 DIA 1
## 768 H490 DIA 1
## 769 H533 DIA 1
## 770 H588 B580 1
## 771 H603 DIA 1
## 772 H60X DIA 1
## 773 H708 DIA 1
## 774 H810 DIA 1
## 775 H811 DIA 1
## 776 H813 DIA 1
## 777 H814 D500 1
## 778 I061 D509 1
## 779 I070 DIA 1
## 780 I091 DIA 1
## 781 I129 DIA 1
## 782 I130 DIA 1
## 783 I131 DIA 1
## 784 I132 D649 1
## 785 I201 DIA 1
## 786 I208 DIA 1
## 787 I21 D50 1
## 788 I241 B022 1
## 789 I253 DIA 1
## 790 I254 DIA 1
## 791 I270 DIA 1
## 792 I278 DIA 1
## 793 I289 DIA 1
## 794 I300 C300 1
## 795 I301 DIA 1
## 796 I309 DIA 1
## 797 I313 A419 1
## 798 I319 D649 1
## 799 I330 DIA 1
## 800 I348 DIA 1
## 801 I350 DIA 1
## 802 I358 DIA 1
## 803 I378 DIA 1
## 804 I38X DIA 1
## 805 I400 DIA 1
## 806 I409 DIA 1
## 807 I421 DIA 1
## 808 I429 DIA 1
## 809 I441 DIA 1
## 810 I447 DIA 1
## 811 I453 DIA 1
## 812 I456 DIA 1
## 813 I459 DIA 1
## 814 I461 A419 1
## 815 I472 DIA 1
## 816 I479 DIA 1
## 817 I510 DIA 1
## 818 I601 DIA 1
## 819 I611 DIA 1
## 820 I615 DIA 1
## 821 I636 DIA 1
## 822 I638 DIA 1
## 823 I652 DIA 1
## 824 I663 DIA 1
## 825 I664 DIA 1
## 826 I670 DIA 1
## 827 I671 DIA 1
## 828 I709 A419 1
## 829 I721 DIA 1
## 830 I738 DIA 1
## 831 I742 DIA 1
## 832 I744 DIA 1
## 833 I749 DIA 1
## 834 I772 DIA 1
## 835 I774 DIA 1
## 836 I790 DIA 1
## 837 I803 DIA 1
## 838 I81X DIA 1
## 839 I831 DIA 1
## 840 I839 DIA 1
## 841 I842 DIA 1
## 842 I861 DIA 1
## 843 I864 DIA 1
## 844 I868 B972 1
## 845 I879 DIA 1
## 846 I899 DIA 1
## 847 I950 DIA 1
## 848 I982 DIA 1
## 849 J020 DIA 1
## 850 J030 DIA 1
## 851 J039 DIA 1
## 852 J041 DIA 1
## 853 J118 DIA 1
## 854 J14X B374 1
## 855 J154 DIA 1
## 856 J160 DIA 1
## 857 J172 DIA 1
## 858 J20 DIA 1
## 859 J211 DIA 1
## 860 J22 E11 1
## 861 J321 DIA 1
## 862 J339 DIA 1
## 863 J342 DIA 1
## 864 J382 DIA 1
## 865 J390 DIA 1
## 866 J391 DIA 1
## 867 J398 DIA 1
## 868 J42 D500 1
## 869 J438 DIA 1
## 870 J458 DIA 1
## 871 J61X DIA 1
## 872 J631 DIA 1
## 873 J638 DIA 1
## 874 J691 DIA 1
## 875 J848 DIA 1
## 876 J853 DIA 1
## 877 J948 A190 1
## 878 J949 DIA 1
## 879 J953 DIA 1
## 880 J955 B972 1
## 881 J958 A415 1
## 882 J986 DIA 1
## 883 J989 DIA 1
## 884 J998 DIA 1
## 885 K021 DIA 1
## 886 K039 B374 1
## 887 K040 DIA 1
## 888 K050 B379 1
## 889 K053 DIA 1
## 890 K113 DIA 1
## 891 K121 DIA 1
## 892 K123 A150 1
## 893 K210 DIA 1
## 894 K226 DIA 1
## 895 K25 D53 1
## 896 K254 DIA 1
## 897 K256 DIA 1
## 898 K260 DIA 1
## 899 K261 A418 1
## 900 K270 DIA 1
## 901 K292 A419 1
## 902 K318 DIA 1
## 903 K350 DIA 1
## 904 K359 DIA 1
## 905 K383 DIA 1
## 906 K388 DIA 1
## 907 K402 DIA 1
## 908 K403 DIA 1
## 909 K421 DIA 1
## 910 K440 DIA 1
## 911 K450 DIA 1
## 912 K458 A419 1
## 913 K500 DIA 1
## 914 K565 DIA 1
## 915 K567 DIA 1
## 916 K572 DIA 1
## 917 K580 DIA 1
## 918 K604 DIA 1
## 919 K611 DIA 1
## 920 K620 DIA 1
## 921 K623 DIA 1
## 922 K624 DIA 1
## 923 K626 DIA 1
## 924 K628 DIA 1
## 925 K641 DIA 1
## 926 K649 DIA 1
## 927 K70 E11 1
## 928 K700 B370 1
## 929 K702 DIA 1
## 930 K710 A150 1
## 931 K712 DIA 1
## 932 K720 DIA 1
## 933 K732 D134 1
## 934 K739 DIA 1
## 935 K76 E14 1
## 936 K761 DIA 1
## 937 K768 DIA 1
## 938 K770 D509 1
## 939 K803 DIA 1
## 940 K81 A53 1
## 941 K818 DIA 1
## 942 K86 DIA 1
## 943 K861 DIA 1
## 944 K862 DIA 1
## 945 K914 DIA 1
## 946 K915 DIA 1
## 947 K921 DIA 1
## 948 L00X DIA 1
## 949 L010 D638 1
## 950 L03 E11 1
## 951 L03X E11X 1
## 952 L040 DIA 1
## 953 L043 DIA 1
## 954 L08 DIA 1
## 955 L109 DIA 1
## 956 L139 DIA 1
## 957 L209 DIA 1
## 958 L249 DIA 1
## 959 L26 A499 1
## 960 L270 DIA 1
## 961 L281 DIA 1
## 962 L402 DIA 1
## 963 L414 DIA 1
## 964 L519 D649 1
## 965 L538 D649 1
## 966 L539 DIA 1
## 967 L602 DIA 1
## 968 L702 DIA 1
## 969 L719 DIA 1
## 970 L739 DIA 1
## 971 M053 DIA 1
## 972 M059 DIA 1
## 973 M064 DIA 1
## 974 M073 DIA 1
## 975 M103 DIA 1
## 976 M104 DIA 1
## 977 M120 DIA 1
## 978 M138 DIA 1
## 979 M142 DIA 1
## 980 M146 DIA 1
## 981 M150 DIA 1
## 982 M170 DIA 1
## 983 M171 DIA 1
## 984 M179 D649 1
## 985 M189 D509 1
## 986 M191 DIA 1
## 987 M204 DIA 1
## 988 M231 DIA 1
## 989 M245 DIA 1
## 990 M250 DIA 1
## 991 M313 DIA 1
## 992 M319 DIA 1
## 993 M328 DIA 1
## 994 M331 DIA 1
## 995 M354 DIA 1
## 996 M429 DIA 1
## 997 M430 DIA 1
## 998 M431 DIA 1
## 999 M465 DIA 1
## 1000 M470 DIA 1
## 1001 M471 DIA 1
## 1002 M478 DIA 1
## 1003 M479 DIA 1
## 1004 M488 DIA 1
## 1005 M490 DIA 1
## 1006 M493 DIA 1
## 1007 M511 DIA 1
## 1008 M540 D530 1
## 1009 M542 DIA 1
## 1010 M543 DIA 1
## 1011 M546 DIA 1
## 1012 M621 DIA 1
## 1013 M624 DIA 1
## 1014 M626 DIA 1
## 1015 M650 DIA 1
## 1016 M703 DIA 1
## 1017 M706 DIA 1
## 1018 M711 DIA 1
## 1019 M712 DIA 1
## 1020 M719 DIA 1
## 1021 M762 DIA 1
## 1022 M779 DIA 1
## 1023 M795 DIA 1
## 1024 M819 DIA 1
## 1025 M842 DIA 1
## 1026 M844 DIA 1
## 1027 M878 D649 1
## 1028 M928 DIA 1
## 1029 M932 DIA 1
## 1030 M995 DIA 1
## 1031 N020 DIA 1
## 1032 N029 D649 1
## 1033 N030 DIA 1
## 1034 N038 DIA 1
## 1035 N048 DIA 1
## 1036 N059 DIA 1
## 1037 N118 DIA 1
## 1038 N131 DIA 1
## 1039 N132 DIA 1
## 1040 N144 DIA 1
## 1041 N158 D303 1
## 1042 N18 DIA 1
## 1043 N18X DIA 1
## 1044 N201 DIA 1
## 1045 N210 DIA 1
## 1046 N211 DIA 1
## 1047 N251 DIA 1
## 1048 N258 DIA 1
## 1049 N311 DIA 1
## 1050 N320 DIA 1
## 1051 N321 DIA 1
## 1052 N341 A010 1
## 1053 N342 DIA 1
## 1054 N350 DIA 1
## 1055 N358 DIA 1
## 1056 N391 DIA 1
## 1057 N40 B91 1
## 1058 N419 DIA 1
## 1059 N434 DIA 1
## 1060 N48 DIA 1
## 1061 N489 DIA 1
## 1062 N508 DIA 1
## 1063 N62X DIA 1
## 1064 N998 DIA 1
## 1065 Q444 DIA 1
## 1066 Q850 DIA 1
## 1067 Q874 DIA 1
## 1068 R031 DIA 1
## 1069 R040 DIA 1
## 1070 R049 DIA 1
## 1071 R063 DIA 1
## 1072 R068 DIA 1
## 1073 R070 DIA 1
## 1074 R071 DIA 1
## 1075 R073 DIA 1
## 1076 R092 DIA 1
## 1077 R162 DIA 1
## 1078 R229 DIA 1
## 1079 R270 DIA 1
## 1080 R278 D649 1
## 1081 R400 DIA 1
## 1082 R42X DIA 1
## 1083 R470 DIA 1
## 1084 R488 DIA 1
## 1085 R520 DIA 1
## 1086 R590 DIA 1
## 1087 R634 DIA 1
## 1088 R650 B448 1
## 1089 R730 DIA 1
## 1090 R960 DIA 1
## 1091 S001 DIA 1
## 1092 S009 DIA 1
## 1093 S015 DIA 1
## 1094 S018 DIA 1
## 1095 S026 DIA 1
## 1096 S029 DIA 1
## 1097 S055 B370 1
## 1098 S059 DIA 1
## 1099 S064 DIA 1
## 1100 S089 DIA 1
## 1101 S119 DIA 1
## 1102 S222 B001 1
## 1103 S224 DIA 1
## 1104 S270 DIA 1
## 1105 S272 DIA 1
## 1106 S299 DIA 1
## 1107 S300 DIA 1
## 1108 S302 DIA 1
## 1109 S308 DIA 1
## 1110 S309 DIA 1
## 1111 S310 DIA 1
## 1112 S312 DIA 1
## 1113 S318 DIA 1
## 1114 S321 DIA 1
## 1115 S324 DIA 1
## 1116 S340 DIA 1
## 1117 S370 D649 1
## 1118 S390 DIA 1
## 1119 S407 DIA 1
## 1120 S411 DIA 1
## 1121 S420 DIA 1
## 1122 S421 DIA 1
## 1123 S430 DIA 1
## 1124 S489 DIA 1
## 1125 S507 DIA 1
## 1126 S510 D649 1
## 1127 S518 DIA 1
## 1128 S519 DIA 1
## 1129 S580 DIA 1
## 1130 S589 DIA 1
## 1131 S607 DIA 1
## 1132 S609 DIA 1
## 1133 S618 DIA 1
## 1134 S623 DIA 1
## 1135 S644 DIA 1
## 1136 S668 DIA 1
## 1137 S670 DIA 1
## 1138 S678 DIA 1
## 1139 S689 DIA 1
## 1140 S722 DIA 1
## 1141 S724 DIA 1
## 1142 S728 DIA 1
## 1143 S729 D649 1
## 1144 S809 DIA 1
## 1145 S817 DIA 1
## 1146 S821 DIA 1
## 1147 S823 DIA 1
## 1148 S826 DIA 1
## 1149 S827 DIA 1
## 1150 S828 DIA 1
## 1151 S832 DIA 1
## 1152 S899 DIA 1
## 1153 S908 DIA 1
## 1154 S923 DIA 1
## 1155 S934 DIA 1
## 1156 S948 DIA 1
## 1157 S971 DIA 1
## 1158 S998 DIA 1
## 1159 T054 DIA 1
## 1160 T058 DIA 1
## 1161 T08X B972 1
## 1162 T099 DIA 1
## 1163 T10X DIA 1
## 1164 T139 DIA 1
## 1165 T145 DIA 1
## 1166 T148 DIA 1
## 1167 T202 DIA 1
## 1168 T222 DIA 1
## 1169 T230 DIA 1
## 1170 T243 DIA 1
## 1171 T250 D649 1
## 1172 T302 DIA 1
## 1173 T310 DIA 1
## 1174 T409 DIA 1
## 1175 T424 DIA 1
## 1176 T443 DIA 1
## 1177 T528 DIA 1
## 1178 T541 DIA 1
## 1179 T542 DIA 1
## 1180 T543 DIA 1
## 1181 T634 DIA 1
## 1182 T639 DIA 1
## 1183 T652 DIA 1
## 1184 T740 D649 1
## 1185 T741 DIA 1
## 1186 T818 DIA 1
## 1187 T827 DIA 1
## 1188 T856 B201 1
## 1189 T868 DIA 1
## 1190 T871 DIA 1
## 1191 T889 DIA 1
## 1192 T930 DIA 1
## 1193 U069 DIA 1
## 1194 U205 DIA 1
## 1195 V385 DIA 1
## 1196 W010 DIA 1
## 1197 W079 DIA 1
## 1198 W170 DIA 1
## 1199 W199 DIA 1
## 1200 W204 DIA 1
## 1201 W229 DIA 1
## 1202 X259 DIA 1
## 1203 X699 DIA 1
## 1204 Y279 DIA 1
## 1205 Y411 A153 1
## 1206 Y832 A419 1
## 1207 Y919 DIA 1
## 1208 Z433 DIA 1
## 1209 Z450 DIA 1
## 1210 Z549 DIA 1
## 1211 Z721 DIA 1
## 1212 Z950 DIA 1
## 1213 Z992 DIA 1

### Male, 60 o more years old

## Diag1 Diag2 Frequency
## 1 I10X DIA 2934
## 2 N390 DIA 1160
## 3 N189 DIA 803
## 4 J189 DIA 792
## 5 U071 DIA 766
## 6 J960 DIA 502
## 7 N40X DIA 428
## 8 A419 DIA 369
## 9 D649 DIA 301
## 10 L031 DIA 286
## 11 I500 DIA 245
## 12 I64X DIA 239
## 13 N185 DIA 197
## 14 I219 DIA 173
## 15 E162 DIA 164
## 16 K922 DIA 162
## 17 I509 DIA 161
## 18 J159 DIA 160
## 19 I678 DIA 145
## 20 I639 DIA 143
## 21 K746 DIA 113
## 22 D509 DIA 111
## 23 S913 DIA 94
## 24 I739 DIA 89
## 25 I679 DIA 87
## 26 J128 DIA 85
## 27 J129 DIA 84
## 28 E669 DIA 79
## 29 J841 DIA 71
## 30 L039 DIA 71
## 31 N179 DIA 71
## 32 K703 DIA 70
## 33 N039 DIA 70
## 34 A099 DIA 69
## 35 J90X DIA 66
## 36 I489 DIA 64
## 37 R739 DIA 64
## 38 E039 DIA 63
## 39 A150 DIA 59
## 40 I619 DIA 59
## 41 J849 DIA 59
## 42 I694 DIA 56
## 43 N19X DIA 54
## 44 J690 DIA 53
## 45 A090 DIA 52
## 46 E86X DIA 52
## 47 A418 DIA 50
## 48 J961 DIA 50
## 49 N10X DIA 50
## 50 I119 DIA 48
## 51 G934 DIA 45
## 52 J80X DIA 45
## 53 L97X DIA 45
## 54 G409 DIA 44
## 55 S984 DIA 43
## 56 I480 DIA 41
## 57 L030 DIA 41
## 58 K805 DIA 40
## 59 C61X DIA 39
## 60 I10 DIA 39
## 61 S889 DIA 39
## 62 S981 DIA 39
## 63 I872 DIA 38
## 64 R568 DIA 38
## 65 I159 DIA 37
## 66 I200 DIA 37
## 67 I259 DIA 37
## 68 D539 DIA 36
## 69 G459 DIA 36
## 70 M869 DIA 36
## 71 J180 DIA 35
## 72 L038 DIA 35
## 73 R02X DIA 34
## 74 N139 DIA 33
## 75 T136 DIA 33
## 76 K409 DIA 32
## 77 K811 DIA 32
## 78 K859 DIA 32
## 79 L024 DIA 32
## 80 U072 DIA 32
## 81 I120 DIA 31
## 82 J47X DIA 31
## 83 L899 DIA 31
## 84 N180 DIA 31
## 85 R509 DIA 31
## 86 J459 DIA 30
## 87 K802 DIA 29
## 88 B24X DIA 28
## 89 J188 DIA 28
## 90 K810 DIA 28
## 91 N183 DIA 27
## 92 N184 DIA 27
## 93 R104 DIA 27
## 94 I110 DIA 26
## 95 E160 DIA 25
## 96 I499 DIA 25
## 97 N181 DIA 25
## 98 K729 DIA 24
## 99 S982 DIA 24
## 100 E46X DIA 23
## 101 N110 DIA 23
## 102 N12X DIA 23
## 103 I633 DIA 22
## 104 I634 DIA 22
## 105 J209 DIA 22
## 106 J22X DIA 22
## 107 L089 DIA 22
## 108 I698 DIA 21
## 109 J13X DIA 21
## 110 L890 DIA 21
## 111 E660 DIA 20
## 112 I830 DIA 20
## 113 I610 DIA 19
## 114 J158 DIA 19
## 115 N289 DIA 19
## 116 A169 DIA 18
## 117 I442 DIA 18
## 118 J120 DIA 18
## 119 K259 DIA 18
## 120 S069 DIA 18
## 121 A409 DIA 17
## 122 E871 DIA 17
## 123 I802 DIA 17
## 124 K590 DIA 17
## 125 L023 DIA 17
## 126 N200 DIA 17
## 127 T874 DIA 17
## 128 D648 DIA 16
## 129 E872 DIA 16
## 130 G20X DIA 16
## 131 I209 DIA 16
## 132 I693 DIA 16
## 133 J157 DIA 16
## 134 K769 DIA 16
## 135 N498 DIA 16
## 136 R579 DIA 16
## 137 E785 DIA 15
## 138 I630 DIA 15
## 139 K801 DIA 15
## 140 R18X DIA 15
## 141 R31X DIA 15
## 142 S880 DIA 15
## 143 A09X DIA 14
## 144 E780 DIA 14
## 145 E870 DIA 14
## 146 H360 DIA 14
## 147 I620 DIA 14
## 148 J168 DIA 14
## 149 K297 DIA 14
## 150 K358 DIA 14
## 151 L032 DIA 14
## 152 M109 DIA 14
## 153 N459 DIA 14
## 154 D696 DIA 13
## 155 F03X DIA 13
## 156 I609 DIA 13
## 157 I635 DIA 13
## 158 J152 DIA 13
## 159 J181 DIA 13
## 160 K610 DIA 13
## 161 K800 DIA 13
## 162 L984 DIA 13
## 163 R572 DIA 13
## 164 S065 DIA 13
## 165 S819 DIA 13
## 166 E161 DIA 12
## 167 E668 DIA 12
## 168 G819 DIA 12
## 169 I255 DIA 12
## 170 I832 DIA 12
## 171 K429 DIA 12
## 172 K819 DIA 12
## 173 K830 DIA 12
## 174 R17X DIA 12
## 175 R570 DIA 12
## 176 R609 DIA 12
## 177 S681 DIA 12
## 178 H813 DIA 11
## 179 I210 DIA 11
## 180 I469 DIA 11
## 181 I638 DIA 11
## 182 K30X DIA 11
## 183 K566 DIA 11
## 184 K750 DIA 11
## 185 K85X DIA 11
## 186 L029 DIA 11
## 187 R11X DIA 11
## 188 S721 DIA 11
## 189 S789 DIA 11
## 190 A499 DIA 10
## 191 F102 DIA 10
## 192 G969 DIA 10
## 193 I443 DIA 10
## 194 I490 DIA 10
## 195 J150 DIA 10
## 196 J40X DIA 10
## 197 K439 DIA 10
## 198 K920 DIA 10
## 199 M545 DIA 10
## 200 M725 DIA 10
## 201 S911 DIA 10
## 202 T009 DIA 10
## 203 B86X DIA 9
## 204 D638 DIA 9
## 205 E880 DIA 9
## 206 G510 DIA 9
## 207 H269 DIA 9
## 208 H540 DIA 9
## 209 I252 DIA 9
## 210 I340 DIA 9
## 211 I519 DIA 9
## 212 I743 DIA 9
## 213 I829 DIA 9
## 214 I839 DIA 9
## 215 J069 DIA 9
## 216 K291 DIA 9
## 217 K295 DIA 9
## 218 L022 DIA 9
## 219 L409 DIA 9
## 220 N049 DIA 9
## 221 N492 DIA 9
## 222 N499 DIA 9
## 223 R042 DIA 9
## 224 R33X DIA 9
## 225 R418 DIA 9
## 226 R571 DIA 9
## 227 T814 DIA 9
## 228 D410 DIA 8
## 229 E876 DIA 8
## 230 F209 DIA 8
## 231 F321 DIA 8
## 232 G309 DIA 8
## 233 G610 DIA 8
## 234 I251 DIA 8
## 235 I420 DIA 8
## 236 I629 DIA 8
## 237 I792 DIA 8
## 238 I850 DIA 8
## 239 J42X DIA 8
## 240 J440 DIA 8
## 241 J698 DIA 8
## 242 J852 DIA 8
## 243 J984 DIA 8
## 244 K250 DIA 8
## 245 M009 DIA 8
## 246 M726 DIA 8
## 247 N111 DIA 8
## 248 N172 DIA 8
## 249 N182 DIA 8
## 250 N188 DIA 8
## 251 R688 DIA 8
## 252 S822 DIA 8
## 253 S881 DIA 8
## 254 S912 DIA 8
## 255 A153 DIA 7
## 256 C229 DIA 7
## 257 E059 DIA 7
## 258 E782 DIA 7
## 259 G403 DIA 7
## 260 H409 DIA 7
## 261 I129 DIA 7
## 262 I150 DIA 7
## 263 I269 DIA 7
## 264 I481 DIA 7
## 265 I498 DIA 7
## 266 I702 DIA 7
## 267 I959 DIA 7
## 268 I99X DIA 7
## 269 J029 DIA 7
## 270 J939 DIA 7
## 271 K260 DIA 7
## 272 K269 DIA 7
## 273 K37X DIA 7
## 274 K400 DIA 7
## 275 K402 DIA 7
## 276 L028 DIA 7
## 277 L033 DIA 7
## 278 L89X DIA 7
## 279 M139 DIA 7
## 280 N159 DIA 7
## 281 S729 DIA 7
## 282 S818 DIA 7
## 283 T147 DIA 7
## 284 Y835 DIA 7
## 285 E440 DIA 6
## 286 E875 DIA 6
## 287 F011 DIA 6
## 288 G049 DIA 6
## 289 G255 DIA 6
## 290 G590 DIA 6
## 291 G629 DIA 6
## 292 G909 DIA 6
## 293 G990 DIA 6
## 294 I059 DIA 6
## 295 I130 DIA 6
## 296 I151 DIA 6
## 297 I158 DIA 6
## 298 I249 DIA 6
## 299 I350 DIA 6
## 300 I440 DIA 6
## 301 I612 DIA 6
## 302 I618 DIA 6
## 303 I749 DIA 6
## 304 I771 DIA 6
## 305 I803 DIA 6
## 306 I828 DIA 6
## 307 I859 DIA 6
## 308 J219 DIA 6
## 309 J848 DIA 6
## 310 K745 DIA 6
## 311 L80X DIA 6
## 312 L88X DIA 6
## 313 N133 DIA 6
## 314 N178 DIA 6
## 315 N359 DIA 6
## 316 S917 DIA 6
## 317 T887 DIA 6
## 318 B354 DIA 5
## 319 B370 DIA 5
## 320 C900 DIA 5
## 321 E789 DIA 5
## 322 E878 DIA 5
## 323 F019 DIA 5
## 324 G042 DIA 5
## 325 G408 DIA 5
## 326 H819 DIA 5
## 327 I152 DIA 5
## 328 I250 DIA 5
## 329 I471 DIA 5
## 330 I632 DIA 5
## 331 I800 DIA 5
## 332 J151 DIA 5
## 333 J46X DIA 5
## 334 J631 DIA 5
## 335 J851 DIA 5
## 336 K270 DIA 5
## 337 K279 DIA 5
## 338 K469 DIA 5
## 339 K591 DIA 5
## 340 K717 DIA 5
## 341 K740 DIA 5
## 342 K804 DIA 5
## 343 K851 DIA 5
## 344 K928 DIA 5
## 345 L020 DIA 5
## 346 L021 DIA 5
## 347 L893 DIA 5
## 348 M100 DIA 5
## 349 N040 DIA 5
## 350 N083 DIA 5
## 351 N119 DIA 5
## 352 N170 DIA 5
## 353 N281 DIA 5
## 354 R100 DIA 5
## 355 R410 DIA 5
## 356 R42X DIA 5
## 357 R55X DIA 5
## 358 S682 DIA 5
## 359 T141 DIA 5
## 360 T793 DIA 5
## 361 T876 DIA 5
## 362 43540 DIA 4
## 363 43565 DIA 4
## 364 C910 DIA 4
## 365 D414 DIA 4
## 366 E169 DIA 4
## 367 E43X DIA 4
## 368 E781 DIA 4
## 369 E835 DIA 4
## 370 E889 DIA 4
## 371 F109 DIA 4
## 372 F200 DIA 4
## 373 F410 DIA 4
## 374 G009 DIA 4
## 375 G401 DIA 4
## 376 G632 DIA 4
## 377 G92X DIA 4
## 378 G932 DIA 4
## 379 G936 DIA 4
## 380 G939 DIA 4
## 381 H259 DIA 4
## 382 H811 DIA 4
## 383 H82X DIA 4
## 384 H919 DIA 4
## 385 I258 DIA 4
## 386 I330 DIA 4
## 387 I441 DIA 4
## 388 I479 DIA 4
## 389 I611 DIA 4
## 390 I660 DIA 4
## 391 I744 DIA 4
## 392 I831 DIA 4
## 393 J100 DIA 4
## 394 J156 DIA 4
## 395 J869 DIA 4
## 396 J948 DIA 4
## 397 J988 DIA 4
## 398 K122 DIA 4
## 399 K20X DIA 4
## 400 K219 DIA 4
## 401 K420 DIA 4
## 402 K529 DIA 4
## 403 K562 DIA 4
## 404 K564 DIA 4
## 405 K579 DIA 4
## 406 K659 DIA 4
## 407 K709 DIA 4
## 408 K720 DIA 4
## 409 L088 DIA 4
## 410 L892 DIA 4
## 411 M069 DIA 4
## 412 M146 DIA 4
## 413 N151 DIA 4
## 414 N319 DIA 4
## 415 N450 DIA 4
## 416 R072 DIA 4
## 417 R529 DIA 4
## 418 R578 DIA 4
## 419 R64X DIA 4
## 420 R798 DIA 4
## 421 S610 DIA 4
## 422 S817 DIA 4
## 423 S820 DIA 4
## 424 S828 DIA 4
## 425 S983 DIA 4
## 426 T243 DIA 4
## 427 Z896 DIA 4
## 428 43552 DIA 3
## 429 43561 DIA 3
## 430 E02X DIA 3
## 431 E038 DIA 3
## 432 E249 DIA 3
## 433 E271 DIA 3
## 434 E512 DIA 3
## 435 F067 DIA 3
## 436 F130 DIA 3
## 437 F329 DIA 3
## 438 G060 DIA 3
## 439 G219 DIA 3
## 440 G900 DIA 3
## 441 G931 DIA 3
## 442 G952 DIA 3
## 443 H400 DIA 3
## 444 H669 DIA 3
## 445 I060 DIA 3
## 446 I270 DIA 3
## 447 I451 DIA 3
## 448 I48 DIA 3
## 449 I516 DIA 3
## 450 I600 DIA 3
## 451 I601 DIA 3
## 452 I801 DIA 3
## 453 I821 DIA 3
## 454 J019 DIA 3
## 455 J123 DIA 3
## 456 J14X DIA 3
## 457 J178 DIA 3
## 458 J930 DIA 3
## 459 J942 DIA 3
## 460 J949 DIA 3
## 461 K047 DIA 3
## 462 K350 DIA 3
## 463 K359 DIA 3
## 464 K565 DIA 3
## 465 K603 DIA 3
## 466 K639 DIA 3
## 467 K650 DIA 3
## 468 K719 DIA 3
## 469 K739 DIA 3
## 470 K760 DIA 3
## 471 K803 DIA 3
## 472 K858 DIA 3
## 473 L080 DIA 3
## 474 L309 DIA 3
## 475 L400 DIA 3
## 476 L539 DIA 3
## 477 M430 DIA 3
## 478 M541 DIA 3
## 479 M600 DIA 3
## 480 M866 DIA 3
## 481 M868 DIA 3
## 482 N18X DIA 3
## 483 N219 DIA 3
## 484 N23X DIA 3
## 485 N312 DIA 3
## 486 N433 DIA 3
## 487 N482 DIA 3
## 488 Q667 DIA 3
## 489 R074 DIA 3
## 490 R160 DIA 3
## 491 R53X DIA 3
## 492 R54X DIA 3
## 493 R560 DIA 3
## 494 R770 DIA 3
## 495 S099 DIA 3
## 496 S313 DIA 3
## 497 S411 DIA 3
## 498 S618 DIA 3
## 499 S619 DIA 3
## 500 S780 DIA 3
## 501 S920 DIA 3
## 502 T824 DIA 3
## 503 T827 DIA 3
## 504 Z488 DIA 3
## 505 43549 J159 2
## 506 43550 DIA 2
## 507 43554 S881 2
## 508 43556 DIA 2
## 509 43559 DIA 2
## 510 43564 DIA 2
## 511 43568 E86X 2
## 512 43577 DIA 2
## 513 A083 DIA 2
## 514 B449 J47X 2
## 515 B878 DIA 2
## 516 C258 DIA 2
## 517 C348 DIA 2
## 518 D042 DIA 2
## 519 D151 DIA 2
## 520 D589 DIA 2
## 521 D590 DIA 2
## 522 D591 DIA 2
## 523 D642 DIA 2
## 524 E212 DIA 2
## 525 E240 DIA 2
## 526 E831 DIA 2
## 527 F009 DIA 2
## 528 F018 DIA 2
## 529 F058 DIA 2
## 530 F069 DIA 2
## 531 F09X DIA 2
## 532 F103 DIA 2
## 533 F199 DIA 2
## 534 F412 DIA 2
## 535 F419 DIA 2
## 536 F432 DIA 2
## 537 G020 DIA 2
## 538 G032 DIA 2
## 539 G039 DIA 2
## 540 G041 DIA 2
## 541 G058 DIA 2
## 542 G310 DIA 2
## 543 G311 DIA 2
## 544 G328 DIA 2
## 545 G400 DIA 2
## 546 G402 DIA 2
## 547 G405 DIA 2
## 548 G410 DIA 2
## 549 G450 DIA 2
## 550 G578 DIA 2
## 551 G938 DIA 2
## 552 H050 DIA 2
## 553 H059 DIA 2
## 554 H109 DIA 2
## 555 H350 DIA 2
## 556 H544 DIA 2
## 557 H602 DIA 2
## 558 H609 DIA 2
## 559 H900 DIA 2
## 560 H902 DIA 2
## 561 I050 DIA 2
## 562 I089 DIA 2
## 563 I139 DIA 2
## 564 I211 DIA 2
## 565 I213 DIA 2
## 566 I229 DIA 2
## 567 I240 DIA 2
## 568 I248 DIA 2
## 569 I279 DIA 2
## 570 I352 DIA 2
## 571 I379 DIA 2
## 572 I459 DIA 2
## 573 I482 DIA 2
## 574 I517 DIA 2
## 575 I613 DIA 2
## 576 I631 DIA 2
## 577 I659 R568 2
## 578 I670 DIA 2
## 579 I671 DIA 2
## 580 I691 DIA 2
## 581 I748 DIA 2
## 582 I775 DIA 2
## 583 I776 DIA 2
## 584 I871 DIA 2
## 585 I879 DIA 2
## 586 I891 DIA 2
## 587 I983 DIA 2
## 588 J101 DIA 2
## 589 J15 E11 2
## 590 J160 DIA 2
## 591 J41 DIA 2
## 592 J441 DIA 2
## 593 J450 DIA 2
## 594 J64X DIA 2
## 595 J679 DIA 2
## 596 J680 DIA 2
## 597 J840 DIA 2
## 598 J850 DIA 2
## 599 J91X DIA 2
## 600 J938 DIA 2
## 601 J980 DIA 2
## 602 K046 DIA 2
## 603 K253 DIA 2
## 604 K294 DIA 2
## 605 K296 DIA 2
## 606 K298 DIA 2
## 607 K319 DIA 2
## 608 K351 DIA 2
## 609 K419 DIA 2
## 610 K421 DIA 2
## 611 K449 DIA 2
## 612 K550 DIA 2
## 613 K560 DIA 2
## 614 K570 DIA 2
## 615 K593 DIA 2
## 616 K630 DIA 2
## 617 K638 DIA 2
## 618 K648 DIA 2
## 619 K701 DIA 2
## 620 K704 DIA 2
## 621 K766 DIA 2
## 622 K768 DIA 2
## 623 K822 DIA 2
## 624 K828 DIA 2
## 625 K839 DIA 2
## 626 L03 DIA 2
## 627 L891 DIA 2
## 628 M013 DIA 2
## 629 M059 DIA 2
## 630 M131 DIA 2
## 631 M199 DIA 2
## 632 M311 DIA 2
## 633 M353 DIA 2
## 634 M490 DIA 2
## 635 M704 DIA 2
## 636 M705 DIA 2
## 637 M724 DIA 2
## 638 M729 DIA 2
## 639 M793 DIA 2
## 640 M819 DIA 2
## 641 M860 DIA 2
## 642 M863 DIA 2
## 643 M864 DIA 2
## 644 M878 DIA 2
## 645 N009 DIA 2
## 646 N029 DIA 2
## 647 N048 DIA 2
## 648 N209 DIA 2
## 649 N210 DIA 2
## 650 N220 DIA 2
## 651 N302 DIA 2
## 652 N320 DIA 2
## 653 N322 DIA 2
## 654 N410 DIA 2
## 655 N411 DIA 2
## 656 N485 DIA 2
## 657 N990 DIA 2
## 658 Q249 DIA 2
## 659 Q610 DIA 2
## 660 R000 DIA 2
## 661 R040 DIA 2
## 662 R05X DIA 2
## 663 R060 DIA 2
## 664 R066 DIA 2
## 665 R091 DIA 2
## 666 R092 DIA 2
## 667 R101 DIA 2
## 668 R162 DIA 2
## 669 R221 DIA 2
## 670 R32X DIA 2
## 671 R392 DIA 2
## 672 R402 DIA 2
## 673 R451 DIA 2
## 674 R490 DIA 2
## 675 R51X DIA 2
## 676 R69X DIA 2
## 677 S009 DIA 2
## 678 S024 DIA 2
## 679 S064 DIA 2
## 680 S109 DIA 2
## 681 S202 DIA 2
## 682 S224 DIA 2
## 683 S271 DIA 2
## 684 S323 DIA 2
## 685 S324 DIA 2
## 686 S480 DIA 2
## 687 S519 DIA 2
## 688 S701 DIA 2
## 689 S842 DIA 2
## 690 S910 DIA 2
## 691 T054 DIA 2
## 692 T131 DIA 2
## 693 T140 DIA 2
## 694 T143 DIA 2
## 695 T149 DIA 2
## 696 T240 DIA 2
## 697 T242 DIA 2
## 698 T253 DIA 2
## 699 T302 DIA 2
## 700 T600 DIA 2
## 701 T633 DIA 2
## 702 T68X DIA 2
## 703 T813 DIA 2
## 704 T856 DIA 2
## 705 T857 DIA 2
## 706 T905 DIA 2
## 707 W019 DIA 2
## 708 W199 DIA 2
## 709 Y841 DIA 2
## 710 Z540 DIA 2
## 711 Z950 DIA 2
## 712 A812 A419 1
## 713 B269 A090 1
## 714 B427 A419 1
## 715 B479 A162 1
## 716 B59X B210 1
## 717 C091 A419 1
## 718 C449 A419 1
## 719 C772 C252 1
## 720 D199 B370 1
## 721 D373 A162 1
## 722 D439 C719 1
## 723 D630 B222 1
## 724 D686 C780 1
## 725 D699 D649 1
## 726 D70X B370 1
## 727 D729 D500 1
## 728 E069 DIA 1
## 729 E12 DIA 1
## 730 E15X DIA 1
## 731 E168 DIA 1
## 732 E230 DIA 1
## 733 E348 DIA 1
## 734 E441 DIA 1
## 735 E538 DIA 1
## 736 E728 DIA 1
## 737 E768 DIA 1
## 738 E784 DIA 1
## 739 E786 DIA 1
## 740 E790 DIA 1
## 741 E849 DIA 1
## 742 E873 A419 1
## 743 E874 DIA 1
## 744 E888 DIA 1
## 745 F002 DIA 1
## 746 F012 DIA 1
## 747 F013 DIA 1
## 748 F020 DIA 1
## 749 F023 DIA 1
## 750 F050 DIA 1
## 751 F059 DIA 1
## 752 F064 DIA 1
## 753 F078 DIA 1
## 754 F101 DIA 1
## 755 F132 DIA 1
## 756 F192 DIA 1
## 757 F194 DIA 1
## 758 F238 DIA 1
## 759 F239 DIA 1
## 760 F31 DIA 1
## 761 F310 DIA 1
## 762 F322 DIA 1
## 763 F328 DIA 1
## 764 F449 DIA 1
## 765 F481 DIA 1
## 766 F500 DIA 1
## 767 F514 DIA 1
## 768 F608 DIA 1
## 769 F609 B379 1
## 770 F639 DIA 1
## 771 F88X DIA 1
## 772 G01X DIA 1
## 773 G030 DIA 1
## 774 G040 DIA 1
## 775 G062 DIA 1
## 776 G08X D890 1
## 777 G218 DIA 1
## 778 G258 DIA 1
## 779 G300 C169 1
## 780 G35X DIA 1
## 781 G373 DIA 1
## 782 G418 DIA 1
## 783 G430 DIA 1
## 784 G432 DIA 1
## 785 G442 DIA 1
## 786 G458 DIA 1
## 787 G464 DIA 1
## 788 G468 DIA 1
## 789 G500 DIA 1
## 790 G528 DIA 1
## 791 G529 DIA 1
## 792 G531 DIA 1
## 793 G540 DIA 1
## 794 G544 DIA 1
## 795 G560 DIA 1
## 796 G561 DIA 1
## 797 G588 DIA 1
## 798 G609 DIA 1
## 799 G619 DIA 1
## 800 G638 DIA 1
## 801 G64X DIA 1
## 802 G700 DIA 1
## 803 G729 DIA 1
## 804 G820 DIA 1
## 805 G825 DIA 1
## 806 G902 DIA 1
## 807 G911 DIA 1
## 808 G912 DIA 1
## 809 G919 DIA 1
## 810 G958 DIA 1
## 811 G959 DIA 1
## 812 G961 DIA 1
## 813 G970 DIA 1
## 814 G98X DIA 1
## 815 H041 DIA 1
## 816 H100 DIA 1
## 817 H150 A539 1
## 818 H160 DIA 1
## 819 H162 DIA 1
## 820 H218 DIA 1
## 821 H280 DIA 1
## 822 H438 D649 1
## 823 H46X DIA 1
## 824 H549 DIA 1
## 825 H588 A527 1
## 826 H600 DIA 1
## 827 H601 DIA 1
## 828 H622 DIA 1
## 829 H652 DIA 1
## 830 H653 DIA 1
## 831 H664 DIA 1
## 832 H708 DIA 1
## 833 H903 DIA 1
## 834 H905 DIA 1
## 835 H911 DIA 1
## 836 I061 DIA 1
## 837 I069 DIA 1
## 838 I079 DIA 1
## 839 I080 DIA 1
## 840 I081 DIA 1
## 841 I12 DIA 1
## 842 I131 DIA 1
## 843 I201 DIA 1
## 844 I212 DIA 1
## 845 I260 DIA 1
## 846 I28 E11 1
## 847 I289 DIA 1
## 848 I311 DIA 1
## 849 I313 A159 1
## 850 I358 DIA 1
## 851 I369 DIA 1
## 852 I400 DIA 1
## 853 I438 DIA 1
## 854 I452 A418 1
## 855 I454 DIA 1
## 856 I460 D649 1
## 857 I461 DIA 1
## 858 I472 DIA 1
## 859 I495 DIA 1
## 860 I501 DIA 1
## 861 I608 DIA 1
## 862 I614 DIA 1
## 863 I615 DIA 1
## 864 I650 DIA 1
## 865 I651 DIA 1
## 866 I663 DIA 1
## 867 I674 DIA 1
## 868 I688 DIA 1
## 869 I690 DIA 1
## 870 I692 DIA 1
## 871 I708 DIA 1
## 872 I709 DIA 1
## 873 I718 DIA 1
## 874 I724 DIA 1
## 875 I728 DIA 1
## 876 I729 DIA 1
## 877 I738 DIA 1
## 878 I742 DIA 1
## 879 I770 DIA 1
## 880 I798 DIA 1
## 881 I808 DIA 1
## 882 I809 DIA 1
## 883 I822 DIA 1
## 884 I864 DIA 1
## 885 I868 A090 1
## 886 I870 DIA 1
## 887 I889 DIA 1
## 888 I898 DIA 1
## 889 I951 DIA 1
## 890 I958 B972 1
## 891 I988 DIA 1
## 892 J00X DIA 1
## 893 J014 DIA 1
## 894 J042 0 1
## 895 J068 B342 1
## 896 J09X DIA 1
## 897 J111 DIA 1
## 898 J154 DIA 1
## 899 J155 D509 1
## 900 J182 DIA 1
## 901 J22 B972 1
## 902 J329 DIA 1
## 903 J330 DIA 1
## 904 J340 B49X 1
## 905 J359 DIA 1
## 906 J390 DIA 1
## 907 J398 DIA 1
## 908 J410 DIA 1
## 909 J438 DIA 1
## 910 J448 DIA 1
## 911 J61X DIA 1
## 912 J620 DIA 1
## 913 J633 DIA 1
## 914 J853 DIA 1
## 915 J940 DIA 1
## 916 J951 DIA 1
## 917 J982 DIA 1
## 918 J98O DIA 1
## 919 K050 DIA 1
## 920 K109 DIA 1
## 921 K112 DIA 1
## 922 K121 DIA 1
## 923 K20 DIA 1
## 924 K220 A150 1
## 925 K221 DIA 1
## 926 K222 DIA 1
## 927 K225 DIA 1
## 928 K228 DIA 1
## 929 K254 D509 1
## 930 k259 DIA 1
## 931 K264 DIA 1
## 932 K310 DIA 1
## 933 K311 D649 1
## 934 K317 DIA 1
## 935 K318 D649 1
## 936 K352 DIA 1
## 937 K353 DIA 1
## 938 K40 C61 1
## 939 K404 DIA 1
## 940 K460 DIA 1
## 941 K500 DIA 1
## 942 K510 DIA 1
## 943 K519 DIA 1
## 944 K552 DIA 1
## 945 K559 DIA 1
## 946 K563 DIA 1
## 947 K567 DIA 1
## 948 K605 D539 1
## 949 K612 DIA 1
## 950 K631 DIA 1
## 951 K632 A419 1
## 952 K640 DIA 1
## 953 K649 DIA 1
## 954 K660 DIA 1
## 955 K661 DIA 1
## 956 K700 DIA 1
## 957 K718 DIA 1
## 958 K721 DIA 1
## 959 K743 DIA 1
## 960 K752 DIA 1
## 961 K753 DIA 1
## 962 K759 DIA 1
## 963 K767 DIA 1
## 964 K80 DIA 1
## 965 K818 DIA 1
## 966 K823 DIA 1
## 967 K829 DIA 1
## 968 K831 DIA 1
## 969 K832 DIA 1
## 970 K833 DIA 1
## 971 K838 D649 1
## 972 K869 DIA 1
## 973 K913 DIA 1
## 974 K914 DIA 1
## 975 L048 DIA 1
## 976 L081 D508 1
## 977 L100 DIA 1
## 978 L102 DIA 1
## 979 L108 DIA 1
## 980 L109 A409 1
## 981 L120 DIA 1
## 982 L129 DIA 1
## 983 L14X DIA 1
## 984 L208 DIA 1
## 985 L209 DIA 1
## 986 L219 DIA 1
## 987 L271 DIA 1
## 988 L281 DIA 1
## 989 L308 DIA 1
## 990 L480 DIA 1
## 991 L501 DIA 1
## 992 L512 DIA 1
## 993 L640 DIA 1
## 994 L719 DIA 1
## 995 L89 DIA 1
## 996 L903 DIA 1
## 997 L905 DIA 1
## 998 L921 DIA 1
## 999 L929 DIA 1
## 1000 L958 D649 1
## 1001 L959 DIA 1
## 1002 L97 DIA 1
## 1003 L982 DIA 1
## 1004 M008 DIA 1
## 1005 M018 DIA 1
## 1006 M029 DIA 1
## 1007 M030 DIA 1
## 1008 M050 DIA 1
## 1009 M104 DIA 1
## 1010 M112 DIA 1
## 1011 M125 DIA 1
## 1012 M150 DIA 1
## 1013 M160 DIA 1
## 1014 M169 DIA 1
## 1015 M190 DIA 1
## 1016 M211 DIA 1
## 1017 M243 DIA 1
## 1018 M254 DIA 1
## 1019 M255 DIA 1
## 1020 M313 DIA 1
## 1021 M316 DIA 1
## 1022 M329 DIA 1
## 1023 M348 DIA 1
## 1024 M359 DIA 1
## 1025 M361 DIA 1
## 1026 M431 DIA 1
## 1027 M462 DIA 1
## 1028 M464 DIA 1
## 1029 M47 E14 1
## 1030 M479 DIA 1
## 1031 M480 DIA 1
## 1032 M489 DIA 1
## 1033 M511 DIA 1
## 1034 M512 DIA 1
## 1035 M543 DIA 1
## 1036 M544 DIA 1
## 1037 M549 DIA 1
## 1038 M620 DIA 1
## 1039 M623 DIA 1
## 1040 M626 DIA 1
## 1041 M658 DIA 1
## 1042 M701 DIA 1
## 1043 M720 DIA 1
## 1044 M722 DIA 1
## 1045 M728 DIA 1
## 1046 M753 DIA 1
## 1047 M754 DIA 1
## 1048 M755 D689 1
## 1049 M773 DIA 1
## 1050 M779 DIA 1
## 1051 M809 DIA 1
## 1052 M850 DIA 1
## 1053 M865 DIA 1
## 1054 M870 DIA 1
## 1055 M879 DIA 1
## 1056 M898 DIA 1
## 1057 M902 DIA 1
## 1058 M922 D500 1
## 1059 M960 DIA 1
## 1060 N000 DIA 1
## 1061 N002 DIA 1
## 1062 N020 DIA 1
## 1063 N059 DIA 1
## 1064 N063 A09X 1
## 1065 N078 DIA 1
## 1066 N10 B972 1
## 1067 N118 D500 1
## 1068 N13 E11 1
## 1069 N130 DIA 1
## 1070 N131 DIA 1
## 1071 N132 A419 1
## 1072 N135 DIA 1
## 1073 N144 D649 1
## 1074 N158 DIA 1
## 1075 N19 D599 1
## 1076 N202 D509 1
## 1077 N26X D649 1
## 1078 N288 DIA 1
## 1079 N309 DIA 1
## 1080 N310 DIA 1
## 1081 N321 DIA 1
## 1082 N360 DIA 1
## 1083 N398 DIA 1
## 1084 N40 DIA 1
## 1085 N418 DIA 1
## 1086 N44X DIA 1
## 1087 N46 DIA 1
## 1088 N47X DIA 1
## 1089 N481 DIA 1
## 1090 N508 DIA 1
## 1091 N509 DIA 1
## 1092 N81 B972 1
## 1093 N841 DIA 1
## 1094 Q019 DIA 1
## 1095 Q612 C609 1
## 1096 Q810 DIA 1
## 1097 R001 DIA 1
## 1098 R02 D509 1
## 1099 R073 DIA 1
## 1100 R102 DIA 1
## 1101 R103 DIA 1
## 1102 R17 C249 1
## 1103 R190 DIA 1
## 1104 R229 DIA 1
## 1105 R398 DIA 1
## 1106 R478 DIA 1
## 1107 R488 DIA 1
## 1108 R54 A415 1
## 1109 R58X DIA 1
## 1110 R601 DIA 1
## 1111 R650 DIA 1
## 1112 R740 DIA 1
## 1113 R829 B972 1
## 1114 R931 DIA 1
## 1115 R960 DIA 1
## 1116 R99X DIA 1
## 1117 S014 DIA 1
## 1118 S027 DIA 1
## 1119 S029 DIA 1
## 1120 S031 DIA 1
## 1121 S056 DIA 1
## 1122 S059 DIA 1
## 1123 S061 DIA 1
## 1124 S062 DIA 1
## 1125 S066 DIA 1
## 1126 S068 DIA 1
## 1127 S089 DIA 1
## 1128 S098 DIA 1
## 1129 S100 DIA 1
## 1130 S143 DIA 1
## 1131 S144 DIA 1
## 1132 S159 DIA 1
## 1133 S203 DIA 1
## 1134 S223 DIA 1
## 1135 S298 DIA 1
## 1136 S299 DIA 1
## 1137 S301 DIA 1
## 1138 S310 DIA 1
## 1139 S311 DIA 1
## 1140 S315 DIA 1
## 1141 S325 D648 1
## 1142 S328 DIA 1
## 1143 S341 DIA 1
## 1144 S372 DIA 1
## 1145 S373 A419 1
## 1146 S422 DIA 1
## 1147 S423 DIA 1
## 1148 S430 DIA 1
## 1149 S518 DIA 1
## 1150 S611 DIA 1
## 1151 S627 DIA 1
## 1152 S684 DIA 1
## 1153 S700 DIA 1
## 1154 S708 DIA 1
## 1155 S711 DIA 1
## 1156 S718 DIA 1
## 1157 S723 DIA 1
## 1158 S724 DIA 1
## 1159 S728 DIA 1
## 1160 S730 DIA 1
## 1161 S749 DIA 1
## 1162 S800 DIA 1
## 1163 S801 DIA 1
## 1164 S821 DIA 1
## 1165 S824 DIA 1
## 1166 S826 DIA 1
## 1167 S827 DIA 1
## 1168 S860 DIA 1
## 1169 S900 DIA 1
## 1170 S902 DIA 1
## 1171 S907 DIA 1
## 1172 S978 DIA 1
## 1173 T013 DIA 1
## 1174 T018 DIA 1
## 1175 T050 DIA 1
## 1176 T056 DIA 1
## 1177 T059 DIA 1
## 1178 T068 DIA 1
## 1179 T10X DIA 1
## 1180 T116 A418 1
## 1181 T135 DIA 1
## 1182 T212 DIA 1
## 1183 T241 DIA 1
## 1184 T291 DIA 1
## 1185 T292 DIA 1
## 1186 T300 DIA 1
## 1187 T348 DIA 1
## 1188 T365 DIA 1
## 1189 T368 DIA 1
## 1190 T451 DIA 1
## 1191 T509 DIA 1
## 1192 T58X DIA 1
## 1193 T601 DIA 1
## 1194 T635 DIA 1
## 1195 T659 DIA 1
## 1196 T670 DIA 1
## 1197 T674 DIA 1
## 1198 T784 DIA 1
## 1199 T803 D689 1
## 1200 T825 DIA 1
## 1201 T848 DIA 1
## 1202 T861 DIA 1
## 1203 T871 DIA 1
## 1204 T935 DIA 1
## 1205 T936 DIA 1
## 1206 T983 DIA 1
## 1207 U069 DIA 1
## 1208 V099 DIA 1
## 1209 W179 DIA 1
## 1210 Y913 DIA 1
## 1211 Y919 DIA 1
## 1212 Z038 DIA 1
## 1213 Z519 DIA 1
## 1214 Z930 DIA 1

## Female

## Diag1 Diag2 Frequency
## 1 N390 DIA 7896
## 2 I10X DIA 7066
## 3 N189 DIA 1857
## 4 J189 DIA 1544
## 5 U071 DIA 1184
## 6 D649 DIA 1027
## 7 A419 DIA 894
## 8 J960 DIA 865
## 9 N10X DIA 719
## 10 E669 DIA 566
## 11 L031 DIA 551
## 12 I500 DIA 514
## 13 E162 DIA 470
## 14 N185 DIA 457
## 15 E039 DIA 446
## 16 J969 DIA 409
## 17 D509 DIA 383
## 18 I64X DIA 354
## 19 N110 DIA 338
## 20 K746 DIA 300
## 21 N12X DIA 285
## 22 I509 DIA 284
## 23 K922 DIA 274
## 24 R739 DIA 255
## 25 R104 DIA 254
## 26 A560 DIA 244
## 27 J159 DIA 238
## 28 A090 DIA 237
## 29 A099 DIA 228
## 30 L039 DIA 226
## 31 B972 DIA 219
## 32 I639 DIA 216
## 33 I678 DIA 215
## 34 E86X DIA 203
## 35 K811 DIA 187
## 36 K802 DIA 186
## 37 N200 DIA 185
## 38 N179 DIA 183
## 39 I219 DIA 168
## 40 J849 DIA 168
## 41 A150 DIA 152
## 42 I679 DIA 152
## 43 N039 DIA 148
## 44 K859 DIA 146
## 45 J841 DIA 144
## 46 J90X DIA 143
## 47 N19X DIA 128
## 48 N819 DIA 128
## 49 J128 DIA 121
## 50 J459 DIA 121
## 51 G934 DIA 116
## 52 K297 DIA 116
## 53 I119 DIA 115
## 54 M069 DIA 114
## 55 S913 DIA 112
## 56 K703 DIA 101
## 57 D539 DIA 100
## 58 K801 DIA 99
## 59 K810 DIA 97
## 60 D500 DIA 96
## 61 L97X DIA 94
## 62 A418 DIA 93
## 63 I489 DIA 93
## 64 E660 DIA 92
## 65 I159 DIA 92
## 66 J449 DIA 92
## 67 G409 DIA 89
## 68 J209 DIA 87
## 69 J80X DIA 87
## 70 R509 DIA 87
## 71 I619 DIA 84
## 72 L030 DIA 84
## 73 I694 DIA 82
## 74 R11X DIA 82
## 75 J961 DIA 81
## 76 K800 DIA 78
## 77 E160 DIA 77
## 78 I739 DIA 77
## 79 K805 DIA 77
## 80 K850 DIA 77
## 81 L023 DIA 77
## 82 N399 DIA 76
## 83 S720 DIA 75
## 84 J180 DIA 74
## 85 N832 DIA 72
## 86 L038 DIA 71
## 87 N151 DIA 71
## 88 N813 DIA 71
## 89 U072 DIA 70
## 90 I480 DIA 69
## 91 N111 DIA 66
## 92 N184 DIA 66
## 93 K295 DIA 65
## 94 I110 DIA 64
## 95 E668 DIA 63
## 96 L024 DIA 61
## 97 N181 DIA 61
## 98 E46X DIA 60
## 99 J13X DIA 59
## 100 K429 DIA 58
## 101 L899 DIA 58
## 102 R568 DIA 58
## 103 E872 DIA 57
## 104 I872 DIA 56
## 105 K291 DIA 56
## 106 N180 DIA 56
## 107 G459 DIA 55
## 108 N760 DIA 55
## 109 K729 DIA 54
## 110 K85X DIA 54
## 111 L032 DIA 54
## 112 O829 DIA 54
## 113 K819 DIA 53
## 114 R572 DIA 53
## 115 E782 DIA 52
## 116 K439 DIA 52
## 117 T136 DIA 52
## 118 J690 DIA 51
## 119 R18X DIA 51
## 120 G20X DIA 50
## 121 I120 DIA 50
## 122 J120 DIA 50
## 123 K590 DIA 49
## 124 N119 DIA 49
## 125 J188 DIA 48
## 126 J22X DIA 48
## 127 C539 DIA 47
## 128 R51X DIA 47
## 129 F412 DIA 46
## 130 I150 DIA 45
## 131 R02X DIA 45
## 132 A09X DIA 44
## 133 F329 DIA 43
## 134 K808 DIA 43
## 135 J81X DIA 42
## 136 I633 DIA 41
## 137 S889 DIA 41
## 138 A409 DIA 40
## 139 O821 DIA 40
## 140 R042 DIA 40
## 141 T814 DIA 40
## 142 I48X DIA 39
## 143 I693 DIA 39
## 144 I802 DIA 39
## 145 N183 DIA 39
## 146 A162 DIA 38
## 147 E059 DIA 38
## 148 I469 DIA 38
## 149 M545 DIA 38
## 150 N209 DIA 38
## 151 N61X DIA 38
## 152 S984 DIA 38
## 153 A010 DIA 37
## 154 A169 DIA 37
## 155 I259 DIA 37
## 156 I499 DIA 37
## 157 I610 DIA 37
## 158 H360 DIA 36
## 159 I200 DIA 36
## 160 J029 DIA 36
## 161 K566 DIA 36
## 162 L022 DIA 36
## 163 L890 DIA 36
## 164 E871 DIA 35
## 165 F419 DIA 35
## 166 S721 DIA 35
## 167 F03X DIA 34
## 168 I830 DIA 34
## 169 L089 DIA 34
## 170 N814 DIA 34
## 171 R100 DIA 34
## 172 R101 DIA 34
## 173 A159 DIA 33
## 174 E038 DIA 33
## 175 J158 DIA 33
## 176 L984 DIA 33
## 177 N049 DIA 33
## 178 N133 DIA 33
## 179 K30X DIA 32
## 180 K830 DIA 32
## 181 L028 DIA 32
## 182 D693 DIA 31
## 183 K610 DIA 31
## 184 L021 DIA 31
## 185 N289 DIA 31
## 186 O234 DIA 31
## 187 I829 DIA 30
## 188 N771 DIA 30
## 189 O990 DIA 30
## 190 S981 DIA 30
## 191 I609 DIA 29
## 192 I859 DIA 29
## 193 K750 DIA 27
## 194 L020 DIA 27
## 195 M726 DIA 27
## 196 M869 DIA 27
## 197 R609 DIA 27
## 198 S069 DIA 27
## 199 S729 DIA 27
## 200 C189 DIA 26
## 201 C56X DIA 26
## 202 G590 DIA 26
## 203 I442 DIA 26
## 204 I634 DIA 26
## 205 L89X DIA 26
## 206 N812 DIA 26
## 207 F200 DIA 25
## 208 K851 DIA 25
## 209 L033 DIA 25
## 210 O034 DIA 25
## 211 S822 DIA 25
## 212 E43X DIA 24
## 213 E878 DIA 24
## 214 J100 DIA 24
## 215 K409 DIA 24
## 216 M725 DIA 24
## 217 O342 DIA 24
## 218 A020 DIA 23
## 219 A90X DIA 23
## 220 G309 DIA 23
## 221 G610 DIA 23
## 222 J984 DIA 23
## 223 K290 DIA 23
## 224 O809 DIA 23
## 225 E161 DIA 22
## 226 E440 DIA 22
## 227 G632 DIA 22
## 228 G819 DIA 22
## 229 K047 DIA 22
## 230 N23X DIA 22
## 231 N300 DIA 22
## 232 E876 DIA 21
## 233 G442 DIA 21
## 234 I490 DIA 21
## 235 I630 DIA 21
## 236 I698 DIA 21
## 237 I850 DIA 21
## 238 J448 DIA 21
## 239 K37X DIA 21
## 240 O064 DIA 21
## 241 O149 DIA 21
## 242 R32X DIA 21
## 243 R571 DIA 21
## 244 H050 DIA 20
## 245 N182 DIA 20
## 246 N938 DIA 20
## 247 S819 DIA 20
## 248 S982 DIA 20
## 249 A410 DIA 19
## 250 E880 DIA 19
## 251 I269 DIA 19
## 252 I809 DIA 19
## 253 K296 DIA 19
## 254 N083 DIA 19
## 255 N312 DIA 19
## 256 R31X DIA 19
## 257 S828 DIA 19
## 258 J150 DIA 18
## 259 J157 DIA 18
## 260 J181 DIA 18
## 261 M059 DIA 18
## 262 M179 DIA 18
## 263 O820 DIA 18
## 264 E870 DIA 17
## 265 I828 DIA 17
## 266 I832 DIA 17
## 267 J91X DIA 17
## 268 J980 DIA 17
## 269 L509 DIA 17
## 270 M139 DIA 17
## 271 T887 DIA 17
## 272 D487 DIA 16
## 273 G909 DIA 16
## 274 H269 DIA 16
## 275 I158 DIA 16
## 276 I255 DIA 16
## 277 I743 DIA 16
## 278 J852 DIA 16
## 279 K122 DIA 16
## 280 K469 DIA 16
## 281 N310 DIA 16
## 282 O141 DIA 16
## 283 R570 DIA 16
## 284 S880 DIA 16
## 285 T009 DIA 16
## 286 C220 DIA 15
## 287 E781 DIA 15
## 288 I519 DIA 15
## 289 J152 DIA 15
## 290 J156 DIA 15
## 291 K250 DIA 15
## 292 K359 DIA 15
## 293 K769 DIA 15
## 294 N178 DIA 15
## 295 R601 DIA 15
## 296 G629 DIA 14
## 297 G969 DIA 14
## 298 I249 DIA 14
## 299 I612 DIA 14
## 300 I635 DIA 14
## 301 J068 DIA 14
## 302 J440 DIA 14
## 303 K259 DIA 14
## 304 K279 DIA 14
## 305 K650 DIA 14
## 306 K920 DIA 14
## 307 N818 DIA 14
## 308 O200 DIA 14
## 309 B379 DIA 13
## 310 D391 DIA 13
## 311 H46X DIA 13
## 312 I771 DIA 13
## 313 I959 DIA 13
## 314 K660 DIA 13
## 315 K745 DIA 13
## 316 M544 DIA 13
## 317 N130 DIA 13
## 318 O364 DIA 13
## 319 O800 DIA 13
## 320 R33X DIA 13
## 321 R418 DIA 13
## 322 S911 DIA 13
## 323 T874 DIA 13
## 324 D410 DIA 12
## 325 D531 DIA 12
## 326 E249 DIA 12
## 327 E441 DIA 12
## 328 F019 DIA 12
## 329 H669 DIA 12
## 330 I340 DIA 12
## 331 I471 DIA 12
## 332 I516 DIA 12
## 333 J123 DIA 12
## 334 J450 DIA 12
## 335 K564 DIA 12
## 336 K659 DIA 12
## 337 K929 DIA 12
## 338 N220 DIA 12
## 339 N872 DIA 12
## 340 T813 DIA 12
## 341 A400 DIA 11
## 342 C250 DIA 11
## 343 D376 DIA 11
## 344 E835 DIA 11
## 345 F011 DIA 11
## 346 F321 DIA 11
## 347 G408 DIA 11
## 348 G510 DIA 11
## 349 H811 DIA 11
## 350 I151 DIA 11
## 351 I210 DIA 11
## 352 I611 DIA 11
## 353 I620 DIA 11
## 354 I629 DIA 11
## 355 J069 DIA 11
## 356 J42X DIA 11
## 357 J698 DIA 11
## 358 K420 DIA 11
## 359 K760 DIA 11
## 360 L88X DIA 11
## 361 M321 DIA 11
## 362 N172 DIA 11
## 363 N219 DIA 11
## 364 N709 DIA 11
## 365 N840 DIA 11
## 366 R001 DIA 11
## 367 T054 DIA 11
## 368 T141 DIA 11
## 369 T147 DIA 11
## 370 T793 DIA 11
## 371 D069 DIA 10
## 372 G049 DIA 10
## 373 H280 DIA 10
## 374 H813 DIA 10
## 375 H82X DIA 10
## 376 I749 DIA 10
## 377 I839 DIA 10
## 378 J039 DIA 10
## 379 K20X DIA 10
## 380 K591 DIA 10
## 381 K803 DIA 10
## 382 L309 DIA 10
## 383 L893 DIA 10
## 384 M009 DIA 10
## 385 N309 DIA 10
## 386 N328 DIA 10
## 387 N394 DIA 10
## 388 N398 DIA 10
## 389 N830 DIA 10
## 390 R688 DIA 10
## 391 R770 DIA 10
## 392 S723 DIA 10
## 393 T252 DIA 10
## 394 T302 DIA 10
## 395 T633 DIA 10
## 396 Z488 DIA 10
## 397 C221 DIA 9
## 398 E041 DIA 9
## 399 F009 DIA 9
## 400 G255 DIA 9
## 401 G400 DIA 9
## 402 G919 DIA 9
## 403 H259 DIA 9
## 404 H409 DIA 9
## 405 I152 DIA 9
## 406 I674 DIA 9
## 407 J219 DIA 9
## 408 J410 DIA 9
## 409 J631 DIA 9
## 410 K269 DIA 9
## 411 K294 DIA 9
## 412 K318 DIA 9
## 413 K625 DIA 9
## 414 N040 DIA 9
## 415 N170 DIA 9
## 416 N210 DIA 9
## 417 N971 DIA 9
## 418 O210 DIA 9
## 419 R579 DIA 9
## 420 S422 DIA 9
## 421 S619 DIA 9
## 422 S724 DIA 9
## 423 T131 DIA 9
## 424 A170 DIA 8
## 425 B028 DIA 8
## 426 B465 DIA 8
## 427 C910 DIA 8
## 428 E169 DIA 8
## 429 E230 DIA 8
## 430 E242 DIA 8
## 431 G360 DIA 8
## 432 G990 DIA 8
## 433 I350 DIA 8
## 434 I443 DIA 8
## 435 I600 DIA 8
## 436 I613 DIA 8
## 437 J36X DIA 8
## 438 J981 DIA 8
## 439 K351 DIA 8
## 440 K352 DIA 8
## 441 K768 DIA 8
## 442 M255 DIA 8
## 443 M431 DIA 8
## 444 N000 DIA 8
## 445 N159 DIA 8
## 446 N202 DIA 8
## 447 N751 DIA 8
## 448 N810 DIA 8
## 449 N870 DIA 8
## 450 N879 DIA 8
## 451 N911 DIA 8
## 452 N952 DIA 8
## 453 O140 DIA 8
## 454 O230 DIA 8
## 455 O249 DIA 8
## 456 R42X DIA 8
## 457 S065 DIA 8
## 458 S818 DIA 8
## 459 S820 DIA 8
## 460 S917 DIA 8
## 461 S980 DIA 8
## 462 W199 DIA 8
## 463 43566 DIA 7
## 464 A156 DIA 7
## 465 A279 DIA 7
## 466 B461 DIA 7
## 467 E02X DIA 7
## 468 E049 DIA 7
## 469 E874 DIA 7
## 470 E875 DIA 7
## 471 E889 DIA 7
## 472 F050 DIA 7
## 473 F059 DIA 7
## 474 F320 DIA 7
## 475 F341 DIA 7
## 476 F449 DIA 7
## 477 G589 DIA 7
## 478 H650 DIA 7
## 479 H919 DIA 7
## 480 I129 DIA 7
## 481 I429 DIA 7
## 482 I460 DIA 7
## 483 I618 DIA 7
## 484 I632 DIA 7
## 485 I702 DIA 7
## 486 I776 DIA 7
## 487 J151 DIA 7
## 488 J848 DIA 7
## 489 J869 DIA 7
## 490 K210 DIA 7
## 491 K221 DIA 7
## 492 K632 DIA 7
## 493 K839 DIA 7
## 494 L088 DIA 7
## 495 L409 DIA 7
## 496 L959 DIA 7
## 497 M331 DIA 7
## 498 M512 DIA 7
## 499 M600 DIA 7
## 500 M866 DIA 7
## 501 N809 DIA 7
## 502 N816 DIA 7
## 503 O020 DIA 7
## 504 O689 DIA 7
## 505 R030 DIA 7
## 506 R072 DIA 7
## 507 R53X DIA 7
## 508 R55X DIA 7
## 509 S202 DIA 7
## 510 S525 DIA 7
## 511 S912 DIA 7
## 512 43553 DIA 6
## 513 E220 DIA 6
## 514 E240 DIA 6
## 515 F064 DIA 6
## 516 F410 DIA 6
## 517 F448 DIA 6
## 518 G959 DIA 6
## 519 I250 DIA 6
## 520 I252 DIA 6
## 521 I447 DIA 6
## 522 I48 DIA 6
## 523 I775 DIA 6
## 524 I803 DIA 6
## 525 J040 DIA 6
## 526 J851 DIA 6
## 527 J939 DIA 6
## 528 J948 DIA 6
## 529 J949 DIA 6
## 530 K299 DIA 6
## 531 K658 DIA 6
## 532 K721 DIA 6
## 533 K759 DIA 6
## 534 K804 DIA 6
## 535 K861 DIA 6
## 536 L891 DIA 6
## 537 L989 DIA 6
## 538 M053 DIA 6
## 539 M169 DIA 6
## 540 M549 DIA 6
## 541 M819 DIA 6
## 542 N009 DIA 6
## 543 N059 DIA 6
## 544 N139 DIA 6
## 545 N158 DIA 6
## 546 N188 DIA 6
## 547 N288 DIA 6
## 548 N738 DIA 6
## 549 N820 DIA 6
## 550 O021 DIA 6
## 551 O429 DIA 6
## 552 O730 DIA 6
## 553 O758 DIA 6
## 554 R000 DIA 6
## 555 R392 DIA 6
## 556 S411 DIA 6
## 557 S789 DIA 6
## 558 T888 A150 6
## 559 Y835 DIA 6
## 560 Z519 DIA 6
## 561 43549 DIA 5
## 562 43552 DIA 5
## 563 43564 DIA 5
## 564 E222 DIA 5
## 565 E784 DIA 5
## 566 E873 DIA 5
## 567 F323 DIA 5
## 568 F328 DIA 5
## 569 G439 DIA 5
## 570 G619 DIA 5
## 571 H540 DIA 5
## 572 H601 DIA 5
## 573 H660 DIA 5
## 574 H663 DIA 5
## 575 I051 DIA 5
## 576 I059 DIA 5
## 577 I130 DIA 5
## 578 I270 DIA 5
## 579 I420 DIA 5
## 580 I459 DIA 5
## 581 I631 DIA 5
## 582 I709 DIA 5
## 583 I779 DIA 5
## 584 J850 DIA 5
## 585 K260 DIA 5
## 586 K319 DIA 5
## 587 K350 DIA 5
## 588 K353 DIA 5
## 589 K400 DIA 5
## 590 K449 DIA 5
## 591 K567 DIA 5
## 592 K661 DIA 5
## 593 K719 DIA 5
## 594 K766 DIA 5
## 595 K863 DIA 5
## 596 K928 DIA 5
## 597 L304 DIA 5
## 598 L400 DIA 5
## 599 M150 DIA 5
## 600 M300 DIA 5
## 601 M479 DIA 5
## 602 M490 DIA 5
## 603 M729 DIA 5
## 604 M793 DIA 5
## 605 N050 DIA 5
## 606 N132 DIA 5
## 607 N201 DIA 5
## 608 N281 DIA 5
## 609 N391 DIA 5
## 610 N736 DIA 5
## 611 N800 DIA 5
## 612 O069 DIA 5
## 613 O600 DIA 5
## 614 R074 DIA 5
## 615 R092 DIA 5
## 616 R402 DIA 5
## 617 R520 DIA 5
## 618 R54X DIA 5
## 619 R578 DIA 5
## 620 R69X DIA 5
## 621 S423 DIA 5
## 622 S681 DIA 5
## 623 S810 DIA 5
## 624 S983 DIA 5
## 625 T140 DIA 5
## 626 T824 DIA 5
## 627 43571 DIA 4
## 628 43580 DIA 4
## 629 A064 DIA 4
## 630 A521 DIA 4
## 631 D329 DIA 4
## 632 D609 DIA 4
## 633 E271 DIA 4
## 634 E538 DIA 4
## 635 E700 DIA 4
## 636 E755 DIA 4
## 637 F09X DIA 4
## 638 F130 DIA 4
## 639 F339 DIA 4
## 640 F411 DIA 4
## 641 G009 DIA 4
## 642 G373 DIA 4
## 643 G402 DIA 4
## 644 G530 DIA 4
## 645 G618 DIA 4
## 646 G92X DIA 4
## 647 G931 DIA 4
## 648 G932 DIA 4
## 649 G936 DIA 4
## 650 H024 DIA 4
## 651 H043 DIA 4
## 652 H654 DIA 4
## 653 H701 DIA 4
## 654 H810 DIA 4
## 655 H903 DIA 4
## 656 I060 DIA 4
## 657 I071 DIA 4
## 658 I211 DIA 4
## 659 I251 DIA 4
## 660 I260 DIA 4
## 661 I309 DIA 4
## 662 I479 DIA 4
## 663 I481 DIA 4
## 664 I501 DIA 4
## 665 I517 DIA 4
## 666 I614 DIA 4
## 667 I64 DIA 4
## 668 I671 DIA 4
## 669 I691 DIA 4
## 670 I770 DIA 4
## 671 I822 DIA 4
## 672 J00X DIA 4
## 673 J019 DIA 4
## 674 J139 DIA 4
## 675 J14X DIA 4
## 676 J170 DIA 4
## 677 J386 DIA 4
## 678 J398 DIA 4
## 679 J938 DIA 4
## 680 J988 DIA 4
## 681 K046 DIA 4
## 682 K113 DIA 4
## 683 K219 DIA 4
## 684 K293 DIA 4
## 685 K402 DIA 4
## 686 K623 DIA 4
## 687 K710 DIA 4
## 688 K717 DIA 4
## 689 K720 DIA 4
## 690 K740 DIA 4
## 691 K744 DIA 4
## 692 K829 DIA 4
## 693 K85 DIA 4
## 694 L080 DIA 4
## 695 L500 DIA 4
## 696 L511 DIA 4
## 697 L719 DIA 4
## 698 L80X DIA 4
## 699 L89 DIA 4
## 700 L892 DIA 4
## 701 M541 DIA 4
## 702 M842 DIA 4
## 703 M868 DIA 4
## 704 N029 DIA 4
## 705 N131 DIA 4
## 706 N320 DIA 4
## 707 N322 DIA 4
## 708 N63X DIA 4
## 709 N72X DIA 4
## 710 N871 DIA 4
## 711 O335 DIA 4
## 712 O40X DIA 4
## 713 O479 DIA 4
## 714 O828 DIA 4
## 715 O860 DIA 4
## 716 R190 DIA 4
## 717 R410 DIA 4
## 718 R58X DIA 4
## 719 R600 DIA 4
## 720 R64X DIA 4
## 721 R99X DIA 4
## 722 S010 DIA 4
## 723 S099 DIA 4
## 724 S328 DIA 4
## 725 S424 DIA 4
## 726 S520 DIA 4
## 727 S628 DIA 4
## 728 S700 DIA 4
## 729 S722 DIA 4
## 730 S781 DIA 4
## 731 S823 DIA 4
## 732 S826 DIA 4
## 733 S829 DIA 4
## 734 S910 DIA 4
## 735 T07X DIA 4
## 736 T243 DIA 4
## 737 T600 DIA 4
## 738 T630 DIA 4
## 739 T634 DIA 4
## 740 T827 DIA 4
## 741 T857 DIA 4
## 742 Z359 DIA 4
## 743 43519 DIA 3
## 744 43554 DIA 3
## 745 43558 DIA 3
## 746 43560 DIA 3
## 747 43563 DIA 3
## 748 43570 DIA 3
## 749 B022 DIA 3
## 750 D50 DIA 3
## 751 E030 DIA 3
## 752 E031 DIA 3
## 753 E034 DIA 3
## 754 E042 DIA 3
## 755 E052 DIA 3
## 756 E209 DIA 3
## 757 E229 DIA 3
## 758 E232 DIA 3
## 759 E559 DIA 3
## 760 F010 DIA 3
## 761 F067 DIA 3
## 762 F069 DIA 3
## 763 F101 DIA 3
## 764 F319 DIA 3
## 765 F432 DIA 3
## 766 F445 DIA 3
## 767 F450 DIA 3
## 768 G041 DIA 3
## 769 G042 DIA 3
## 770 G048 DIA 3
## 771 G060 DIA 3
## 772 G122 DIA 3
## 773 G219 DIA 3
## 774 G311 DIA 3
## 775 G401 DIA 3
## 776 G403 DIA 3
## 777 G500 DIA 3
## 778 G588 DIA 3
## 779 G628 DIA 3
## 780 G633 DIA 3
## 781 G700 DIA 3
## 782 G98X DIA 3
## 783 H010 DIA 3
## 784 H103 DIA 3
## 785 H109 DIA 3
## 786 H268 DIA 3
## 787 H352 DIA 3
## 788 H368 DIA 3
## 789 H440 DIA 3
## 790 H490 DIA 3
## 791 H527 DIA 3
## 792 H588 DIA 3
## 793 H609 DIA 3
## 794 H664 DIA 3
## 795 H900 DIA 3
## 796 I050 DIA 3
## 797 I131 DIA 3
## 798 I139 DIA 3
## 799 I258 DIA 3
## 800 I339 DIA 3
## 801 I390 DIA 3
## 802 I446 DIA 3
## 803 I461 DIA 3
## 804 I482 DIA 3
## 805 I528 DIA 3
## 806 I602 DIA 3
## 807 I606 DIA 3
## 808 I690 DIA 3
## 809 I700 DIA 3
## 810 I729 DIA 3
## 811 I738 DIA 3
## 812 I800 DIA 3
## 813 I864 DIA 3
## 814 I890 DIA 3
## 815 I99X DIA 3
## 816 J200 DIA 3
## 817 J320 DIA 3
## 818 J380 DIA 3
## 819 J393 DIA 3
## 820 J441 DIA 3
## 821 J451 DIA 3
## 822 J679 DIA 3
## 823 J680 DIA 3
## 824 J840 DIA 3
## 825 J853 DIA 3
## 826 J941 DIA 3
## 827 K102 DIA 3
## 828 K253 DIA 3
## 829 K257 DIA 3
## 830 k259 DIA 3
## 831 K270 DIA 3
## 832 K298 DIA 3
## 833 K316 DIA 3
## 834 K36X DIA 3
## 835 K461 DIA 3
## 836 K565 DIA 3
## 837 K579 DIA 3
## 838 K630 DIA 3
## 839 K631 DIA 3
## 840 K648 DIA 3
## 841 K649 DIA 3
## 842 K700 DIA 3
## 843 K818 DIA 3
## 844 K820 DIA 3
## 845 K823 DIA 3
## 846 K904 DIA 3
## 847 L00X DIA 3
## 848 L100 DIA 3
## 849 L102 DIA 3
## 850 L259 DIA 3
## 851 L600 DIA 3
## 852 L930 DIA 3
## 853 M068 DIA 3
## 854 M131 DIA 3
## 855 M154 DIA 3
## 856 M480 DIA 3
## 857 M542 DIA 3
## 858 M790 DIA 3
## 859 M809 DIA 3
## 860 M861 DIA 3
## 861 M870 DIA 3
## 862 M900 DIA 3
## 863 N048 DIA 3
## 864 N079 DIA 3
## 865 N118 DIA 3
## 866 N137 DIA 3
## 867 N211 DIA 3
## 868 N311 DIA 3
## 869 N340 DIA 3
## 870 N700 DIA 3
## 871 N701 DIA 3
## 872 N710 DIA 3
## 873 N750 DIA 3
## 874 N758 DIA 3
## 875 N761 DIA 3
## 876 N778 DIA 3
## 877 N835 DIA 3
## 878 N851 DIA 3
## 879 N859 DIA 3
## 880 N898 DIA 3
## 881 N930 DIA 3
## 882 N994 DIA 3
## 883 O009 DIA 3
## 884 O100 DIA 3
## 885 O13X DIA 3
## 886 O16X DIA 3
## 887 O268 DIA 3
## 888 O367 DIA 3
## 889 O410 DIA 3
## 890 O441 DIA 3
## 891 O649 DIA 3
## 892 O669 DIA 3
## 893 O839 DIA 3
## 894 O912 DIA 3
## 895 O992 DIA 3
## 896 Q250 DIA 3
## 897 Q501 DIA 3
## 898 R160 DIA 3
## 899 R221 DIA 3
## 900 R222 DIA 3
## 901 R522 DIA 3
## 902 R529 DIA 3
## 903 R560 DIA 3
## 904 R651 DIA 3
## 905 R828 DIA 3
## 906 S009 DIA 3
## 907 S062 DIA 3
## 908 S210 DIA 3
## 909 S223 DIA 3
## 910 S310 DIA 3
## 911 S311 DIA 3
## 912 S320 DIA 3
## 913 S610 DIA 3
## 914 S682 DIA 3
## 915 S711 DIA 3
## 916 S817 DIA 3
## 917 S821 DIA 3
## 918 T10X DIA 3
## 919 T242 DIA 3
## 920 T301 DIA 3
## 921 T424 DIA 3
## 922 T477 DIA 3
## 923 T702 DIA 3
## 924 T825 DIA 3
## 925 Z100 DIA 3
## 926 43498 DIA 2
## 927 43501 I10X 2
## 928 43509 DIA 2
## 929 43526 I509 2
## 930 43541 I10X 2
## 931 43547 DIA 2
## 932 43551 N390 2
## 933 43555 DIA 2
## 934 43559 DIA 2
## 935 43562 I10X 2
## 936 43567 DIA 2
## 937 43569 N390 2
## 938 43572 DIA 2
## 939 43574 DIA 2
## 940 43579 DIA 2
## 941 A58X DIA 2
## 942 A599 DIA 2
## 943 C166 DIA 2
## 944 C310 DIA 2
## 945 C540 DIA 2
## 946 C959 DIA 2
## 947 D061 DIA 2
## 948 D521 DIA 2
## 949 E000 DIA 2
## 950 E010 DIA 2
## 951 E012 DIA 2
## 952 E032 DIA 2
## 953 E033 DIA 2
## 954 E055 DIA 2
## 955 E069 DIA 2
## 956 E079 DIA 2
## 957 E10 DIA 2
## 958 E15X DIA 2
## 959 E200 DIA 2
## 960 E210 DIA 2
## 961 E236 DIA 2
## 962 E270 DIA 2
## 963 E274 DIA 2
## 964 E283 DIA 2
## 965 E308 DIA 2
## 966 E45X DIA 2
## 967 E662 DIA 2
## 968 E739 DIA 2
## 969 E748 DIA 2
## 970 E756 DIA 2
## 971 E788 DIA 2
## 972 E839 DIA 2
## 973 E840 DIA 2
## 974 E859 DIA 2
## 975 E890 DIA 2
## 976 F028 DIA 2
## 977 F051 DIA 2
## 978 F058 DIA 2
## 979 F062 DIA 2
## 980 F072 DIA 2
## 981 F078 DIA 2
## 982 F102 DIA 2
## 983 F105 DIA 2
## 984 F190 DIA 2
## 985 F203 DIA 2
## 986 F206 DIA 2
## 987 F250 DIA 2
## 988 F29X DIA 2
## 989 F332 DIA 2
## 990 F409 DIA 2
## 991 F418 DIA 2
## 992 F459 DIA 2
## 993 F606 DIA 2
## 994 F799 DIA 2
## 995 F811 DIA 2
## 996 F99X DIA 2
## 997 G008 DIA 2
## 998 G050 DIA 2
## 999 G253 DIA 2
## 1000 G35X DIA 2
## 1001 G404 DIA 2
## 1002 G410 DIA 2
## 1003 G430 DIA 2
## 1004 G448 DIA 2
## 1005 G464 DIA 2
## 1006 G519 DIA 2
## 1007 G579 DIA 2
## 1008 G709 DIA 2
## 1009 G710 DIA 2
## 1010 G729 DIA 2
## 1011 G802 DIA 2
## 1012 G903 DIA 2
## 1013 G911 DIA 2
## 1014 G938 DIA 2
## 1015 G952 DIA 2
## 1016 G998 B690 2
## 1017 H059 DIA 2
## 1018 H060 DIA 2
## 1019 H062 DIA 2
## 1020 H110 DIA 2
## 1021 H400 DIA 2
## 1022 H431 DIA 2
## 1023 H544 DIA 2
## 1024 H549 DIA 2
## 1025 H620 DIA 2
## 1026 H651 DIA 2
## 1027 H700 DIA 2
## 1028 H709 DIA 2
## 1029 H812 DIA 2
## 1030 I052 DIA 2
## 1031 I069 DIA 2
## 1032 I132 DIA 2
## 1033 I213 DIA 2
## 1034 I229 DIA 2
## 1035 I278 DIA 2
## 1036 I288 DIA 2
## 1037 I351 DIA 2
## 1038 I378 DIA 2
## 1039 I38X DIA 2
## 1040 I421 DIA 2
## 1041 I440 DIA 2
## 1042 I441 DIA 2
## 1043 I510 DIA 2
## 1044 I601 DIA 2
## 1045 I604 DIA 2
## 1046 I607 DIA 2
## 1047 I608 DIA 2
## 1048 I615 DIA 2
## 1049 I688 DIA 2
## 1050 I719 DIA 2
## 1051 I728 DIA 2
## 1052 I742 DIA 2
## 1053 I801 DIA 2
## 1054 I821 DIA 2
## 1055 I842 DIA 2
## 1056 I848 DIA 2
## 1057 I849 DIA 2
## 1058 I868 DIA 2
## 1059 I889 DIA 2
## 1060 I899 DIA 2
## 1061 I982 DIA 2
## 1062 J010 DIA 2
## 1063 J014 R040 2
## 1064 J101 DIA 2
## 1065 J153 DIA 2
## 1066 J155 DIA 2
## 1067 J160 DIA 2
## 1068 J208 DIA 2
## 1069 J210 DIA 2
## 1070 J304 DIA 2
## 1071 J342 DIA 2
## 1072 J371 DIA 2
## 1073 J390 DIA 2
## 1074 J61X DIA 2
## 1075 J942 DIA 2
## 1076 J950 DIA 2
## 1077 J990 DIA 2
## 1078 J998 DIA 2
## 1079 K041 DIA 2
## 1080 K228 DIA 2
## 1081 K251 DIA 2
## 1082 K263 DIA 2
## 1083 K317 DIA 2
## 1084 K388 DIA 2
## 1085 K389 DIA 2
## 1086 K421 DIA 2
## 1087 K430 DIA 2
## 1088 K440 DIA 2
## 1089 K458 DIA 2
## 1090 K519 DIA 2
## 1091 K559 DIA 2
## 1092 K573 DIA 2
## 1093 K574 DIA 2
## 1094 K589 DIA 2
## 1095 K612 DIA 2
## 1096 K627 DIA 2
## 1097 K628 DIA 2
## 1098 K635 DIA 2
## 1099 K702 DIA 2
## 1100 K711 DIA 2
## 1101 K712 DIA 2
## 1102 K743 DIA 2
## 1103 K828 DIA 2
## 1104 K833 DIA 2
## 1105 K858 DIA 2
## 1106 K860 DIA 2
## 1107 K868 DIA 2
## 1108 K915 DIA 2
## 1109 K918 DIA 2
## 1110 K921 DIA 2
## 1111 L010 DIA 2
## 1112 L043 DIA 2
## 1113 L048 DIA 2
## 1114 L10 DIA 2
## 1115 L209 DIA 2
## 1116 L239 DIA 2
## 1117 L270 A150 2
## 1118 L281 DIA 2
## 1119 L301 DIA 2
## 1120 L402 DIA 2
## 1121 L510 DIA 2
## 1122 L729 DIA 2
## 1123 L932 DIA 2
## 1124 L983 DIA 2
## 1125 M050 DIA 2
## 1126 M058 DIA 2
## 1127 M060 DIA 2
## 1128 M125 DIA 2
## 1129 M130 DIA 2
## 1130 M171 DIA 2
## 1131 M189 DIA 2
## 1132 M254 DIA 2
## 1133 M318 DIA 2
## 1134 M320 DIA 2
## 1135 M330 DIA 2
## 1136 M340 DIA 2
## 1137 M349 DIA 2
## 1138 M411 DIA 2
## 1139 M465 DIA 2
## 1140 M478 DIA 2
## 1141 M531 DIA 2
## 1142 M620 DIA 2
## 1143 M623 DIA 2
## 1144 M624 DIA 2
## 1145 M625 DIA 2
## 1146 M659 DIA 2
## 1147 M712 DIA 2
## 1148 M752 DIA 2
## 1149 M779 DIA 2
## 1150 M795 DIA 2
## 1151 M844 DIA 2
## 1152 M860 DIA 2
## 1153 M879 DIA 2
## 1154 M930 DIA 2
## 1155 M932 DIA 2
## 1156 M939 DIA 2
## 1157 M940 DIA 2
## 1158 N038 DIA 2
## 1159 N069 DIA 2
## 1160 N080 DIA 2
## 1161 N10 DIA 2
## 1162 N150 DIA 2
## 1163 N18 DIA 2
## 1164 N20 DIA 2
## 1165 N228 DIA 2
## 1166 N290 DIA 2
## 1167 N301 DIA 2
## 1168 N318 DIA 2
## 1169 N321 DIA 2
## 1170 N350 DIA 2
## 1171 N368 DIA 2
## 1172 N392 DIA 2
## 1173 N649 DIA 2
## 1174 N711 DIA 2
## 1175 N719 DIA 2
## 1176 N748 DIA 2
## 1177 N763 DIA 2
## 1178 N766 DIA 2
## 1179 N770 DIA 2
## 1180 N829 DIA 2
## 1181 N841 DIA 2
## 1182 N842 DIA 2
## 1183 N857 DIA 2
## 1184 N888 DIA 2
## 1185 N900 DIA 2
## 1186 N910 DIA 2
## 1187 N912 DIA 2
## 1188 N949 DIA 2
## 1189 N979 DIA 2
## 1190 N993 DIA 2
## 1191 O039 DIA 2
## 1192 O211 DIA 2
## 1193 O235 DIA 2
## 1194 O269 DIA 2
## 1195 O321 DIA 2
## 1196 O366 DIA 2
## 1197 O601 DIA 2
## 1198 O60X DIA 2
## 1199 O639 DIA 2
## 1200 O731 DIA 2
## 1201 O911 DIA 2
## 1202 Q181 DIA 2
## 1203 Q211 DIA 2
## 1204 Q249 DIA 2
## 1205 Q282 DIA 2
## 1206 Q505 DIA 2
## 1207 Q603 DIA 2
## 1208 Q667 DIA 2
## 1209 Q803 DIA 2
## 1210 Q909 DIA 2
## 1211 R049 DIA 2
## 1212 R060 DIA 2
## 1213 R070 DIA 2
## 1214 R098 DIA 2
## 1215 R13X DIA 2
## 1216 R14X DIA 2
## 1217 R229 DIA 2
## 1218 R34X DIA 2
## 1219 R35X DIA 2
## 1220 R450 DIA 2
## 1221 R590 DIA 2
## 1222 R634 DIA 2
## 1223 R730 DIA 2
## 1224 R820 DIA 2
## 1225 R827 DIA 2
## 1226 S019 DIA 2
## 1227 S064 DIA 2
## 1228 S066 DIA 2
## 1229 S219 DIA 2
## 1230 S220 DIA 2
## 1231 S271 DIA 2
## 1232 S300 DIA 2
## 1233 S301 DIA 2
## 1234 S321 DIA 2
## 1235 S322 DIA 2
## 1236 S324 DIA 2
## 1237 S364 DIA 2
## 1238 S370 DIA 2
## 1239 S399 DIA 2
## 1240 S400 DIA 2
## 1241 S420 DIA 2
## 1242 S430 DIA 2
## 1243 S526 DIA 2
## 1244 S611 DIA 2
## 1245 S626 DIA 2
## 1246 S701 DIA 2
## 1247 S728 DIA 2
## 1248 S730 DIA 2
## 1249 S770 DIA 2
## 1250 S808 DIA 2
## 1251 S903 DIA 2
## 1252 S922 DIA 2
## 1253 T008 DIA 2
## 1254 T055 DIA 2
## 1255 T056 DIA 2
## 1256 T111 DIA 2
## 1257 T116 DIA 2
## 1258 T245 DIA 2
## 1259 T250 DIA 2
## 1260 T253 DIA 2
## 1261 T310 DIA 2
## 1262 T509 DIA 2
## 1263 T543 DIA 2
## 1264 T659 DIA 2
## 1265 T671 DIA 2
## 1266 T709 DIA 2
## 1267 T740 DIA 2
## 1268 T784 DIA 2
## 1269 T802 DIA 2
## 1270 T809 DIA 2
## 1271 T856 DIA 2
## 1272 T905 DIA 2
## 1273 T983 DIA 2
## 1274 W019 DIA 2
## 1275 W180 DIA 2
## 1276 Z302 DIA 2
## 1277 B178 B029 1
## 1278 B200 A150 1
## 1279 B780 A060 1
## 1280 B971 A90X 1
## 1281 C269 B180 1
## 1282 C412 B24X 1
## 1283 C448 A414 1
## 1284 C460 B24X 1
## 1285 C787 C189 1
## 1286 C793 C349 1
## 1287 C919 C910 1
## 1288 D070 D060 1
## 1289 D169 B24X 1
## 1290 D25 C64X 1
## 1291 D339 C719 1
## 1292 D379 C786 1
## 1293 D823 A419 1
## 1294 E009 DIA 1
## 1295 E011 DIA 1
## 1296 E040 DIA 1
## 1297 E060 DIA 1
## 1298 E063 DIA 1
## 1299 E11.6 DIA 1
## 1300 E111D D250 1
## 1301 E168 DIA 1
## 1302 E208 DIA 1
## 1303 E211 DIA 1
## 1304 E233 DIA 1
## 1305 E237 DIA 1
## 1306 E248 DIA 1
## 1307 E260 D509 1
## 1308 E272 DIA 1
## 1309 E273 A083 1
## 1310 E279 DIA 1
## 1311 E282 D432 1
## 1312 E340 A150 1
## 1313 E344 DIA 1
## 1314 E350 DIA 1
## 1315 E40X DIA 1
## 1316 E43 C169 1
## 1317 E509 DIA 1
## 1318 E60X DIA 1
## 1319 E639 DIA 1
## 1320 E649 A419 1
## 1321 E6691 B972 1
## 1322 E66X D259 1
## 1323 E673 DIA 1
## 1324 E720 DIA 1
## 1325 E722 DIA 1
## 1326 E729 DIA 1
## 1327 E742 DIA 1
## 1328 E752 DIA 1
## 1329 E778 DIA 1
## 1330 E779 DIA 1
## 1331 E786 DIA 1
## 1332 E790 DIA 1
## 1333 E800 DIA 1
## 1334 E806 A090 1
## 1335 E807 DIA 1
## 1336 E831 DIA 1
## 1337 E849 DIA 1
## 1338 E850 DIA 1
## 1339 E893 DIA 1
## 1340 E90X DIA 1
## 1341 F000 DIA 1
## 1342 F002 DIA 1
## 1343 F012 DIA 1
## 1344 F013 DIA 1
## 1345 F018 DIA 1
## 1346 F068 DIA 1
## 1347 F070 DIA 1
## 1348 F129 DIA 1
## 1349 F132 DIA 1
## 1350 F141 DIA 1
## 1351 F171 DIA 1
## 1352 F172 DIA 1
## 1353 F189 DIA 1
## 1354 F201 DIA 1
## 1355 F20X DIA 1
## 1356 F21X D500 1
## 1357 F229 DIA 1
## 1358 F230 D060 1
## 1359 F232 DIA 1
## 1360 F239 DIA 1
## 1361 F259 DIA 1
## 1362 F311 DIA 1
## 1363 F312 DIA 1
## 1364 F313 DIA 1
## 1365 F316 DIA 1
## 1366 F330 DIA 1
## 1367 F41 DIA 1
## 1368 F413 DIA 1
## 1369 F481 DIA 1
## 1370 F504 DIA 1
## 1371 F509 DIA 1
## 1372 F510 DIA 1
## 1373 F519 DIA 1
## 1374 F603 DIA 1
## 1375 F604 DIA 1
## 1376 F609 DIA 1
## 1377 F639 DIA 1
## 1378 F708 DIA 1
## 1379 F709 DIA 1
## 1380 F719 DIA 1
## 1381 F780 DIA 1
## 1382 F800 DIA 1
## 1383 F82X DIA 1
## 1384 F980 DIA 1
## 1385 G000 DIA 1
## 1386 G01X A170 1
## 1387 G030 DIA 1
## 1388 G038 B461 1
## 1389 G039 DIA 1
## 1390 G051 DIA 1
## 1391 G061 DIA 1
## 1392 G10X DIA 1
## 1393 G112 DIA 1
## 1394 G114 DIA 1
## 1395 G20 DIA 1
## 1396 G211 DIA 1
## 1397 G231 DIA 1
## 1398 G249 DIA 1
## 1399 G300 DIA 1
## 1400 G301 DIA 1
## 1401 G310 DIA 1
## 1402 G369 DIA 1
## 1403 G370 DIA 1
## 1404 G371 DIA 1
## 1405 G372 DIA 1
## 1406 G379 DIA 1
## 1407 G412 DIA 1
## 1408 G419 DIA 1
## 1409 G432 DIA 1
## 1410 G440 DIA 1
## 1411 G441 DIA 1
## 1412 G443 DIA 1
## 1413 G450 DIA 1
## 1414 G458 DIA 1
## 1415 G460 DIA 1
## 1416 G473 DIA 1
## 1417 G529 B690 1
## 1418 G538 DIA 1
## 1419 G540 DIA 1
## 1420 G569 DIA 1
## 1421 G580 A010 1
## 1422 G600 DIA 1
## 1423 G603 DIA 1
## 1424 G609 D509 1
## 1425 G638 DIA 1
## 1426 G713 DIA 1
## 1427 G732 A418 1
## 1428 G735 DIA 1
## 1429 G800 DIA 1
## 1430 G822 DIA 1
## 1431 G825 DIA 1
## 1432 G908 DIA 1
## 1433 G912 DIA 1
## 1434 G918 DIA 1
## 1435 G930 DIA 1
## 1436 G939 DIA 1
## 1437 G941 D432 1
## 1438 G948 DIA 1
## 1439 G968 DIA 1
## 1440 H000 DIA 1
## 1441 H027 DIA 1
## 1442 H045 DIA 1
## 1443 H113 DIA 1
## 1444 H160 DIA 1
## 1445 H162 DIA 1
## 1446 H169 DIA 1
## 1447 H189 C539 1
## 1448 H193 DIA 1
## 1449 H208 DIA 1
## 1450 H282 DIA 1
## 1451 H335 A090 1
## 1452 H350 DIA 1
## 1453 H358 DIA 1
## 1454 H359 DIA 1
## 1455 H428 DIA 1
## 1456 H441 DIA 1
## 1457 H448 DIA 1
## 1458 H451 DIA 1
## 1459 H472 DIA 1
## 1460 H492 DIA 1
## 1461 H493 DIA 1
## 1462 H494 DIA 1
## 1463 H524 DIA 1
## 1464 H578 DIA 1
## 1465 H598 DIA 1
## 1466 H600 DIA 1
## 1467 H602 DIA 1
## 1468 H603 DIA 1
## 1469 H604 DIA 1
## 1470 H605 DIA 1
## 1471 H611 DIA 1
## 1472 H622 DIA 1
## 1473 H652 DIA 1
## 1474 H659 DIA 1
## 1475 H818 DIA 1
## 1476 H830 DIA 1
## 1477 H901 A161 1
## 1478 H913 DIA 1
## 1479 H920 DIA 1
## 1480 H933 DIA 1
## 1481 I010 DIA 1
## 1482 I018 DIA 1
## 1483 I061 DIA 1
## 1484 I080 DIA 1
## 1485 I089 DIA 1
## 1486 I208 DIA 1
## 1487 I212 DIA 1
## 1488 I214 DIA 1
## 1489 I233 DIA 1
## 1490 I236 DIA 1
## 1491 I238 DIA 1
## 1492 I256 B342 1
## 1493 I272 DIA 1
## 1494 I280 DIA 1
## 1495 I311 A156 1
## 1496 I319 DIA 1
## 1497 I321 DIA 1
## 1498 I358 DIA 1
## 1499 I361 DIA 1
## 1500 I372 DIA 1
## 1501 I392 B342 1
## 1502 I411 DIA 1
## 1503 I422 DIA 1
## 1504 I424 DIA 1
## 1505 I432 DIA 1
## 1506 I438 DIA 1
## 1507 I451 DIA 1
## 1508 I452 DIA 1
## 1509 I454 DIA 1
## 1510 I470 DIA 1
## 1511 I493 DIA 1
## 1512 I495 DIA 1
## 1513 I498 DIA 1
## 1514 I513 DIA 1
## 1515 I603 DIA 1
## 1516 I61 E11 1
## 1517 I652 DIA 1
## 1518 I660 DIA 1
## 1519 I661 DIA 1
## 1520 I669 DIA 1
## 1521 I670 DIA 1
## 1522 I676 DIA 1
## 1523 I69 DIA 1
## 1524 I692 DIA 1
## 1525 I720 DIA 1
## 1526 I724 DIA 1
## 1527 I730 DIA 1
## 1528 I745 DIA 1
## 1529 I808 DIA 1
## 1530 I831 DIA 1
## 1531 I840 DIA 1
## 1532 I863 DIA 1
## 1533 I870 DIA 1
## 1534 I871 C349 1
## 1535 I891 DIA 1
## 1536 I951 D649 1
## 1537 I958 A418 1
## 1538 I988 DIA 1
## 1539 J038 DIA 1
## 1540 J042 DIA 1
## 1541 J060 DIA 1
## 1542 J09X DIA 1
## 1543 J110 D509 1
## 1544 J118 DIA 1
## 1545 J122 DIA 1
## 1546 J15 A90 1
## 1547 J154 DIA 1
## 1548 J173 B59X 1
## 1549 J178 DIA 1
## 1550 J182 DIA 1
## 1551 J20 DIA 1
## 1552 J20X DIA 1
## 1553 J312 DIA 1
## 1554 J329 DIA 1
## 1555 J340 DIA 1
## 1556 J370 DIA 1
## 1557 J392 DIA 1
## 1558 J40 E11 1
## 1559 J41 E14 1
## 1560 J439 D649 1
## 1561 J45 E11 1
## 1562 J458 DIA 1
## 1563 J45X DIA 1
## 1564 J47 DIA 1
## 1565 J60X DIA 1
## 1566 J628 DIA 1
## 1567 J633 DIA 1
## 1568 J634 D649 1
## 1569 J64X DIA 1
## 1570 J65X DIA 1
## 1571 J671 DIA 1
## 1572 J677 DIA 1
## 1573 J684 DIA 1
## 1574 J691 DIA 1
## 1575 J90 C500 1
## 1576 J929 DIA 1
## 1577 J930 DIA 1
## 1578 J951 DIA 1
## 1579 J958 DIA 1
## 1580 J96 A90 1
## 1581 J982 DIA 1
## 1582 J986 DIA 1
## 1583 K029 DIA 1
## 1584 K040 DIA 1
## 1585 K044 DIA 1
## 1586 K052 DIA 1
## 1587 K068 DIA 1
## 1588 K088 DIA 1
## 1589 K099 DIA 1
## 1590 K103 DIA 1
## 1591 K109 DIA 1
## 1592 K112 DIA 1
## 1593 K115 DIA 1
## 1594 K118 DIA 1
## 1595 K119 A09X 1
## 1596 K120 C859 1
## 1597 K225 DIA 1
## 1598 K226 DIA 1
## 1599 k250 DIA 1
## 1600 K252 DIA 1
## 1601 K254 D539 1
## 1602 K255 DIA 1
## 1603 K264 DIA 1
## 1604 K267 DIA 1
## 1605 K274 DIA 1
## 1606 K275 A419 1
## 1607 K292 DIA 1
## 1608 K310 DIA 1
## 1609 K314 DIA 1
## 1610 K315 DIA 1
## 1611 K381 DIA 1
## 1612 K403 DIA 1
## 1613 K404 DIA 1
## 1614 K410 DIA 1
## 1615 K431 DIA 1
## 1616 K450 DIA 1
## 1617 K460 A419 1
## 1618 K509 DIA 1
## 1619 K512 D500 1
## 1620 K515 DIA 1
## 1621 K520 DIA 1
## 1622 K521 DIA 1
## 1623 K528 DIA 1
## 1624 K550 DIA 1
## 1625 K563 DIA 1
## 1626 K570 DIA 1
## 1627 K572 DIA 1
## 1628 K578 DIA 1
## 1629 K59 DIA 1
## 1630 K592 DIA 1
## 1631 K593 DIA 1
## 1632 K601 DIA 1
## 1633 K602 DIA 1
## 1634 K611 DIA 1
## 1635 K614 DIA 1
## 1636 K626 D539 1
## 1637 K633 D500 1
## 1638 K66 E14 1
## 1639 K70 DIA 1
## 1640 K701 D649 1
## 1641 K704 D509 1
## 1642 K713 A099 1
## 1643 K714 DIA 1
## 1644 K716 DIA 1
## 1645 K718 DIA 1
## 1646 K730 DIA 1
## 1647 K738 DIA 1
## 1648 K739 DIA 1
## 1649 K754 DIA 1
## 1650 K758 DIA 1
## 1651 K767 DIA 1
## 1652 K778 DIA 1
## 1653 K80 DIA 1
## 1654 K80X DIA 1
## 1655 K81 B24 1
## 1656 K81X B24X 1
## 1657 K821 DIA 1
## 1658 K824 DIA 1
## 1659 K831 C259 1
## 1660 K862 DIA 1
## 1661 K869 DIA 1
## 1662 K90 E14 1
## 1663 K913 DIA 1
## 1664 K914 DIA 1
## 1665 K938 DIA 1
## 1666 L03 E11 1
## 1667 L040 DIA 1
## 1668 L059 DIA 1
## 1669 L121 D649 1
## 1670 L200 DIA 1
## 1671 L219 DIA 1
## 1672 L22X DIA 1
## 1673 L231 D509 1
## 1674 L238 DIA 1
## 1675 L280 DIA 1
## 1676 L282 DIA 1
## 1677 L292 DIA 1
## 1678 L298 DIA 1
## 1679 L308 DIA 1
## 1680 L401 DIA 1
## 1681 L408 DIA 1
## 1682 L502 DIA 1
## 1683 L508 DIA 1
## 1684 L512 DIA 1
## 1685 L539 DIA 1
## 1686 L570 DIA 1
## 1687 L680 DIA 1
## 1688 L739 DIA 1
## 1689 L817 DIA 1
## 1690 L819 A099 1
## 1691 L82X DIA 1
## 1692 L84X DIA 1
## 1693 L853 DIA 1
## 1694 L871 DIA 1
## 1695 L921 DIA 1
## 1696 L931 DIA 1
## 1697 L940 DIA 1
## 1698 L958 DIA 1
## 1699 L980 B180 1
## 1700 L982 DIA 1
## 1701 L988 DIA 1
## 1702 L998 DIA 1
## 1703 M000 DIA 1
## 1704 M029 DIA 1
## 1705 M052 DIA 1
## 1706 M0699 DIA 1
## 1707 M082 DIA 1
## 1708 M109 DIA 1
## 1709 M142 DIA 1
## 1710 M153 DIA 1
## 1711 M159 A090 1
## 1712 M160 DIA 1
## 1713 M161 DIA 1
## 1714 M172 DIA 1
## 1715 M175 DIA 1
## 1716 M185 DIA 1
## 1717 M190 DIA 1
## 1718 M198 DIA 1
## 1719 M224 DIA 1
## 1720 M232 DIA 1
## 1721 M235 DIA 1
## 1722 M259 DIA 1
## 1723 M311 C64X 1
## 1724 M313 DIA 1
## 1725 M317 DIA 1
## 1726 M319 DIA 1
## 1727 M328 DIA 1
## 1728 M348 DIA 1
## 1729 M354 DIA 1
## 1730 M359 DIA 1
## 1731 M413 DIA 1
## 1732 M418 DIA 1
## 1733 M421 A498 1
## 1734 M430 DIA 1
## 1735 M489 DIA 1
## 1736 M502 DIA 1
## 1737 M510 DIA 1
## 1738 M518 DIA 1
## 1739 M530 DIA 1
## 1740 M543 DIA 1
## 1741 M548 DIA 1
## 1742 M609 DIA 1
## 1743 M651 DIA 1
## 1744 M653 DIA 1
## 1745 M662 DIA 1
## 1746 M688 DIA 1
## 1747 M719 DIA 1
## 1748 M722 DIA 1
## 1749 M728 DIA 1
## 1750 M751 D693 1
## 1751 M755 DIA 1
## 1752 M758 DIA 1
## 1753 M760 D500 1
## 1754 M761 DIA 1
## 1755 M766 DIA 1
## 1756 M770 DIA 1
## 1757 M792 DIA 1
## 1758 M796 DIA 1
## 1759 M797 DIA 1
## 1760 M800 DIA 1
## 1761 M839 DIA 1
## 1762 M841 DIA 1
## 1763 M843 DIA 1
## 1764 M848 DIA 1
## 1765 M854 C050 1
## 1766 M862 A419 1
## 1767 M863 DIA 1
## 1768 M864 DIA 1
## 1769 M878 DIA 1
## 1770 M901 DIA 1
## 1771 M903 DIA 1
## 1772 M906 DIA 1
## 1773 M948 DIA 1
## 1774 M966 DIA 1
## 1775 M990 DIA 1
## 1776 N020 DIA 1
## 1777 N021 DIA 1
## 1778 N028 DIA 1
## 1779 N030 DIA 1
## 1780 N058 DIA 1
## 1781 N070 DIA 1
## 1782 N078 DIA 1
## 1783 N088 DIA 1
## 1784 N12 DIA 1
## 1785 N13 E14 1
## 1786 N136 DIA 1
## 1787 N138 DIA 1
## 1788 N140 DIA 1
## 1789 N144 DIA 1
## 1790 N164 DIA 1
## 1791 N165 DIA 1
## 1792 N171 DIA 1
## 1793 N21 E13 1
## 1794 N218 DIA 1
## 1795 N222 DIA 1
## 1796 N258 DIA 1
## 1797 N298 DIA 1
## 1798 N302 DIA 1
## 1799 N303 DIA 1
## 1800 N304 D501 1
## 1801 N308 DIA 1
## 1802 N329 A049 1
## 1803 N359 DIA 1
## 1804 N362 DIA 1
## 1805 N369 A419 1
## 1806 N370 DIA 1
## 1807 N498 DIA 1
## 1808 N500 DIA 1
## 1809 N600 DIA 1
## 1810 N601 DIA 1
## 1811 N61 E14 1
## 1812 N62X DIA 1
## 1813 N641 A419 1
## 1814 N645 DIA 1
## 1815 N730 DIA 1
## 1816 N731 D589 1
## 1817 N732 D539 1
## 1818 N734 DIA 1
## 1819 N76 DIA 1
## 1820 N762 DIA 1
## 1821 N765 D049 1
## 1822 N808 DIA 1
## 1823 N828 DIA 1
## 1824 N831 DIA 1
## 1825 N8324 DIA 1
## 1826 N838 DIA 1
## 1827 N839 DIA 1
## 1828 N849 DIA 1
## 1829 N855 DIA 1
## 1830 N890 DIA 1
## 1831 N891 DIA 1
## 1832 N908 DIA 1
## 1833 N909 DIA 1
## 1834 N950 D649 1
## 1835 N951 E10X 1
## 1836 N970 DIA 1
## 1837 N990 DIA 1
## 1838 N992 DIA 1
## 1839 O000 DIA 1
## 1840 O010 DIA 1
## 1841 O019 DIA 1
## 1842 O030 DIA 1
## 1843 O033 DIA 1
## 1844 O054 DIA 1
## 1845 O088 DIA 1
## 1846 O219 DIA 1
## 1847 O220 DIA 1
## 1848 O231 DIA 1
## 1849 O239 DIA 1
## 1850 O243 DIA 1
## 1851 O244 DIA 1
## 1852 O25X DIA 1
## 1853 O260 DIA 1
## 1854 O300 DIA 1
## 1855 O312 DIA 1
## 1856 O323 DIA 1
## 1857 O324 DIA 1
## 1858 O326 DIA 1
## 1859 O330 DIA 1
## 1860 O334 DIA 1
## 1861 O338 DIA 1
## 1862 O339 DIA 1
## 1863 O360 DIA 1
## 1864 O365 DIA 1
## 1865 O368 DIA 1
## 1866 O420 DIA 1
## 1867 O421 DIA 1
## 1868 O439 DIA 1
## 1869 O471 DIA 1
## 1870 O610 DIA 1
## 1871 O624 DIA 1
## 1872 O645 DIA 1
## 1873 O648 DIA 1
## 1874 O662 DIA 1
## 1875 O679 DIA 1
## 1876 O700 DIA 1
## 1877 O710 DIA 1
## 1878 O722 DIA 1
## 1879 O759 DIA 1
## 1880 O8001 DIA 1
## 1881 O808 DIA 1
## 1882 O8284 DIA 1
## 1883 O838 DIA 1
## 1884 O840 B972 1
## 1885 O85X DIA 1
## 1886 O862 DIA 1
## 1887 O863 DIA 1
## 1888 O868 DIA 1
## 1889 O900 DIA 1
## 1890 O908 DIA 1
## 1891 O994 DIA 1
## 1892 P369 DIA 1
## 1893 P95X DIA 1
## 1894 Q046 DIA 1
## 1895 Q049 DIA 1
## 1896 Q219 DIA 1
## 1897 Q253 DIA 1
## 1898 Q259 DIA 1
## 1899 Q273 DIA 1
## 1900 Q279 DIA 1
## 1901 Q400 DIA 1
## 1902 Q444 D649 1
## 1903 Q602 DIA 1
## 1904 Q605 DIA 1
## 1905 Q610 DIA 1
## 1906 Q620 DIA 1
## 1907 Q631 D410 1
## 1908 Q649 DIA 1
## 1909 Q660 DIA 1
## 1910 Q720 C300 1
## 1911 Q738 DIA 1
## 1912 Q780 DIA 1
## 1913 Q828 DIA 1
## 1914 Q831 DIA 1
## 1915 Q96 DIA 1
## 1916 R011 DIA 1
## 1917 R05X A419 1
## 1918 R063 DIA 1
## 1919 R071 DIA 1
## 1920 R090 DIA 1
## 1921 R12X DIA 1
## 1922 R162 DIA 1
## 1923 R198 DIA 1
## 1924 R224 DIA 1
## 1925 R301 D509 1
## 1926 R390 DIA 1
## 1927 R398 DIA 1
## 1928 R458 DIA 1
## 1929 R488 DIA 1
## 1930 R51 DIA 1
## 1931 R599 DIA 1
## 1932 R632 DIA 1
## 1933 R650 DIA 1
## 1934 R652 DIA 1
## 1935 R680 A403 1
## 1936 R740 A090 1
## 1937 R749 DIA 1
## 1938 R785 DIA 1
## 1939 R826 D508 1
## 1940 R831 A152 1
## 1941 R900 DIA 1
## 1942 R960 DIA 1
## 1943 R98X DIA 1
## 1944 S001 DIA 1
## 1945 S007 DIA 1
## 1946 S008 DIA 1
## 1947 S017 DIA 1
## 1948 S025 DIA 1
## 1949 S027 DIA 1
## 1950 S060 DIA 1
## 1951 S06X E10X 1
## 1952 S071 DIA 1
## 1953 S127 DIA 1
## 1954 S129 DIA 1
## 1955 S151 DIA 1
## 1956 S208 DIA 1
## 1957 S218 DIA 1
## 1958 S228 DIA 1
## 1959 S230 DIA 1
## 1960 S270 DIA 1
## 1961 S299 DIA 1
## 1962 S309 DIA 1
## 1963 S318 DIA 1
## 1964 S327 DIA 1
## 1965 S361 D649 1
## 1966 S408 DIA 1
## 1967 S410 DIA 1
## 1968 S418 DIA 1
## 1969 S421 DIA 1
## 1970 S429 DIA 1
## 1971 S459 DIA 1
## 1972 S460 DIA 1
## 1973 S481 DIA 1
## 1974 S489 DIA 1
## 1975 S498 DIA 1
## 1976 S518 DIA 1
## 1977 S521 DIA 1
## 1978 S522 DIA 1
## 1979 S523 DIA 1
## 1980 S531 D649 1
## 1981 S550 DIA 1
## 1982 S589 DIA 1
## 1983 S609 B360 1
## 1984 S627 DIA 1
## 1985 S635 DIA 1
## 1986 S668 DIA 1
## 1987 S670 DIA 1
## 1988 S683 DIA 1
## 1989 S718 DIA 1
## 1990 S748 DIA 1
## 1991 S749 DIA 1
## 1992 S799 DIA 1
## 1993 S801 DIA 1
## 1994 S849 DIA 1
## 1995 S878 DIA 1
## 1996 S909 DIA 1
## 1997 S920 DIA 1
## 1998 S925 D649 1
## 1999 S927 DIA 1
## 2000 S929 DIA 1
## 2001 S930 DIA 1
## 2002 S934 DIA 1
## 2003 S936 A480 1
## 2004 S960 DIA 1
## 2005 S971 DIA 1
## 2006 S998 DIA 1
## 2007 S999 DIA 1
## 2008 T013 DIA 1
## 2009 T019 DIA 1
## 2010 T093 DIA 1
## 2011 T138 DIA 1
## 2012 T142 DIA 1
## 2013 T143 DIA 1
## 2014 T149 DIA 1
## 2015 T150 DIA 1
## 2016 T180 DIA 1
## 2017 T189 DIA 1
## 2018 T191 DIA 1
## 2019 T202 DIA 1
## 2020 T210 DIA 1
## 2021 T212 DIA 1
## 2022 T214 A419 1
## 2023 T222 DIA 1
## 2024 T231 DIA 1
## 2025 T240 DIA 1
## 2026 T292 DIA 1
## 2027 T293 DIA 1
## 2028 T303 DIA 1
## 2029 T304 DIA 1
## 2030 T315 DIA 1
## 2031 T348 DIA 1
## 2032 T441 DIA 1
## 2033 T451 C509 1
## 2034 T479 DIA 1
## 2035 T519 DIA 1
## 2036 T639 DIA 1
## 2037 T698 DIA 1
## 2038 T751 DIA 1
## 2039 T78 DIA 1
## 2040 T782 DIA 1
## 2041 T789 DIA 1
## 2042 T794 DIA 1
## 2043 T801 DIA 1
## 2044 T815 DIA 1
## 2045 T818 DIA 1
## 2046 T819 DIA 1
## 2047 T833 DIA 1
## 2048 T835 D649 1
## 2049 T859 DIA 1
## 2050 T860 DIA 1
## 2051 T861 D649 1
## 2052 T868 DIA 1
## 2053 U069 DIA 1
## 2054 U202 DIA 1
## 2055 W010 DIA 1
## 2056 W018 DIA 1
## 2057 W060 DIA 1
## 2058 W089 DIA 1
## 2059 W159 DIA 1
## 2060 W170 DIA 1
## 2061 W184 DIA 1
## 2062 X100 DIA 1
## 2063 X109 DIA 1
## 2064 X259 DIA 1
## 2065 X690 DIA 1
## 2066 X954 DIA 1
## 2067 Y423 DIA 1
## 2068 Y822 DIA 1
## 2069 Y834 DIA 1
## 2070 Y846 DIA 1
## 2071 Z208 DIA 1
## 2072 Z226 B86X 1
## 2073 Z3593 DIA 1
## 2074 Z391 DIA 1
## 2075 Z392 DIA 1
## 2076 Z433 DIA 1
## 2077 Z540 DIA 1
## 2078 Z639 DIA 1
## 2079 Z730 DIA 1
## 2080 Z749 DIA 1
## 2081 Z896 DIA 1
## 2082 Z932 DIA 1
## 2083 Z958 DIA 1
## 2084 Z988 DIA 1
## 2085 Z992 DIA 1

### Female, 18 - 39 years old

## Diag1 Diag2 Frequency
## 1 N390 DIA 676
## 2 I10X DIA 139
## 3 D649 DIA 123
## 4 U071 DIA 86
## 5 E669 DIA 84
## 6 N189 DIA 76
## 7 J189 DIA 75
## 8 N10X DIA 68
## 9 A419 DIA 64
## 10 J960 DIA 47
## 11 E039 DIA 46
## 12 D509 DIA 44
## 13 O829 DIA 44
## 14 L031 DIA 41
## 15 K859 DIA 37
## 16 O821 DIA 34
## 17 N110 DIA 33
## 18 E162 DIA 32
## 19 R104 DIA 32
## 20 A090 DIA 28
## 21 N12X DIA 26
## 22 N200 DIA 26
## 23 A560 DIA 25
## 24 O990 DIA 25
## 25 O234 DIA 23
## 26 K802 DIA 22
## 27 B972 DIA 21
## 28 K297 DIA 21
## 29 E86X DIA 19
## 30 J969 DIA 19
## 31 K850 DIA 18
## 32 O149 DIA 18
## 33 O809 DIA 18
## 34 R739 DIA 18
## 35 O342 DIA 17
## 36 O820 DIA 17
## 37 K295 DIA 16
## 38 N185 DIA 16
## 39 N760 DIA 16
## 40 O034 DIA 16
## 41 O064 DIA 16
## 42 N61X DIA 15
## 43 E660 DIA 14
## 44 O141 DIA 14
## 45 J459 DIA 13
## 46 K811 DIA 13
## 47 R11X DIA 13
## 48 K358 DIA 12
## 49 N179 DIA 12
## 50 N832 DIA 12
## 51 N939 DIA 12
## 52 O200 DIA 12
## 53 E059 DIA 11
## 54 L032 DIA 11
## 55 L038 DIA 11
## 56 N151 DIA 11
## 57 O364 DIA 11
## 58 O800 DIA 11
## 59 R509 DIA 11
## 60 E46X DIA 10
## 61 F329 DIA 10
## 62 F412 DIA 10
## 63 G409 DIA 10
## 64 J209 DIA 10
## 65 K291 DIA 10
## 66 K85X DIA 10
## 67 N111 DIA 10
## 68 N399 DIA 10
## 69 E43X DIA 9
## 70 E668 DIA 9
## 71 E785 DIA 9
## 72 J128 DIA 9
## 73 K800 DIA 9
## 74 L023 DIA 9
## 75 O210 DIA 9
## 76 R572 DIA 9
## 77 D539 DIA 8
## 78 K801 DIA 8
## 79 K922 DIA 8
## 80 N19X DIA 8
## 81 E038 DIA 7
## 82 E872 DIA 7
## 83 I500 DIA 7
## 84 J029 DIA 7
## 85 J90X DIA 7
## 86 K37X DIA 7
## 87 K805 DIA 7
## 88 K808 DIA 7
## 89 L020 DIA 7
## 90 M069 DIA 7
## 91 M329 DIA 7
## 92 N133 DIA 7
## 93 N771 DIA 7
## 94 N911 DIA 7
## 95 O020 DIA 7
## 96 O140 DIA 7
## 97 A162 DIA 6
## 98 F200 DIA 6
## 99 G442 DIA 6
## 100 I64X DIA 6
## 101 J129 DIA 6
## 102 J159 DIA 6
## 103 K746 DIA 6
## 104 L024 DIA 6
## 105 L97X DIA 6
## 106 N23X DIA 6
## 107 O230 DIA 6
## 108 R042 DIA 6
## 109 R101 DIA 6
## 110 E249 DIA 5
## 111 G610 DIA 5
## 112 G934 DIA 5
## 113 I119 DIA 5
## 114 J188 DIA 5
## 115 K122 DIA 5
## 116 K290 DIA 5
## 117 K810 DIA 5
## 118 K819 DIA 5
## 119 K851 DIA 5
## 120 N209 DIA 5
## 121 N971 DIA 5
## 122 O069 DIA 5
## 123 O249 DIA 5
## 124 O429 DIA 5
## 125 R100 DIA 5
## 126 T633 DIA 5
## 127 A09X DIA 4
## 128 C56X DIA 4
## 129 E440 DIA 4
## 130 E781 DIA 4
## 131 E876 DIA 4
## 132 E878 DIA 4
## 133 F209 DIA 4
## 134 F341 DIA 4
## 135 F419 DIA 4
## 136 G629 DIA 4
## 137 G632 DIA 4
## 138 H660 DIA 4
## 139 I159 DIA 4
## 140 I509 DIA 4
## 141 I739 DIA 4
## 142 J81X DIA 4
## 143 K318 DIA 4
## 144 K351 DIA 4
## 145 K439 DIA 4
## 146 K610 DIA 4
## 147 L022 DIA 4
## 148 L028 DIA 4
## 149 L030 DIA 4
## 150 L089 DIA 4
## 151 L509 DIA 4
## 152 M321 DIA 4
## 153 M545 DIA 4
## 154 N049 DIA 4
## 155 N178 DIA 4
## 156 N319 DIA 4
## 157 N709 DIA 4
## 158 N738 DIA 4
## 159 O021 DIA 4
## 160 O335 DIA 4
## 161 O40X DIA 4
## 162 O479 DIA 4
## 163 O689 DIA 4
## 164 O730 DIA 4
## 165 O828 DIA 4
## 166 R568 DIA 4
## 167 S822 DIA 4
## 168 S913 DIA 4
## 169 Z359 DIA 4
## 170 43563 DIA 3
## 171 A020 DIA 3
## 172 A400 DIA 3
## 173 A409 DIA 3
## 174 F328 DIA 3
## 175 G590 DIA 3
## 176 G990 DIA 3
## 177 H269 DIA 3
## 178 H280 DIA 3
## 179 H360 DIA 3
## 180 H46X DIA 3
## 181 H654 DIA 3
## 182 H664 DIA 3
## 183 H669 DIA 3
## 184 I460 DIA 3
## 185 I469 DIA 3
## 186 I489 DIA 3
## 187 I633 DIA 3
## 188 I743 DIA 3
## 189 J068 DIA 3
## 190 J80X DIA 3
## 191 J852 DIA 3
## 192 K210 DIA 3
## 193 K30X DIA 3
## 194 K359 DIA 3
## 195 K659 DIA 3
## 196 K750 DIA 3
## 197 K863 DIA 3
## 198 L259 DIA 3
## 199 M869 DIA 3
## 200 N180 DIA 3
## 201 N219 DIA 3
## 202 N328 DIA 3
## 203 N751 DIA 3
## 204 N764 DIA 3
## 205 N850 DIA 3
## 206 O268 DIA 3
## 207 O367 DIA 3
## 208 O600 DIA 3
## 209 O758 DIA 3
## 210 O860 DIA 3
## 211 R103 DIA 3
## 212 R18X DIA 3
## 213 R51X DIA 3
## 214 T814 DIA 3
## 215 T888 A150 3
## 216 U072 DIA 3
## 217 Z519 DIA 3
## 218 43553 DIA 2
## 219 43564 DIA 2
## 220 43571 DIA 2
## 221 A410 DIA 2
## 222 A521 DIA 2
## 223 D376 DIA 2
## 224 E209 DIA 2
## 225 E220 DIA 2
## 226 E230 DIA 2
## 227 E236 DIA 2
## 228 E240 DIA 2
## 229 E283 DIA 2
## 230 E441 DIA 2
## 231 E756 DIA 2
## 232 E870 DIA 2
## 233 E871 DIA 2
## 234 F321 DIA 2
## 235 G400 DIA 2
## 236 G633 DIA 2
## 237 G909 DIA 2
## 238 H268 DIA 2
## 239 H409 DIA 2
## 240 H919 DIA 2
## 241 I219 DIA 2
## 242 I471 DIA 2
## 243 I609 DIA 2
## 244 I639 DIA 2
## 245 I671 DIA 2
## 246 I678 DIA 2
## 247 I809 DIA 2
## 248 I830 DIA 2
## 249 I959 DIA 2
## 250 J13X DIA 2
## 251 J150 DIA 2
## 252 J151 DIA 2
## 253 J36X DIA 2
## 254 J40X DIA 2
## 255 J690 DIA 2
## 256 J91X DIA 2
## 257 J984 DIA 2
## 258 K102 DIA 2
## 259 K20X DIA 2
## 260 K296 DIA 2
## 261 K319 DIA 2
## 262 K350 DIA 2
## 263 K353 DIA 2
## 264 K409 DIA 2
## 265 K429 DIA 2
## 266 K529 DIA 2
## 267 K591 DIA 2
## 268 K660 DIA 2
## 269 K661 DIA 2
## 270 K729 DIA 2
## 271 K745 DIA 2
## 272 K760 DIA 2
## 273 K904 DIA 2
## 274 L402 DIA 2
## 275 L890 DIA 2
## 276 L899 DIA 2
## 277 L930 DIA 2
## 278 M320 DIA 2
## 279 N000 DIA 2
## 280 N039 DIA 2
## 281 N040 DIA 2
## 282 N119 DIA 2
## 283 N289 DIA 2
## 284 N312 DIA 2
## 285 N393 DIA 2
## 286 N63X DIA 2
## 287 N700 DIA 2
## 288 N758 DIA 2
## 289 N809 DIA 2
## 290 N813 DIA 2
## 291 N830 DIA 2
## 292 N859 DIA 2
## 293 N872 DIA 2
## 294 N879 DIA 2
## 295 N910 DIA 2
## 296 O009 DIA 2
## 297 O039 DIA 2
## 298 O13X DIA 2
## 299 O269 DIA 2
## 300 O366 DIA 2
## 301 O410 DIA 2
## 302 O441 DIA 2
## 303 O649 DIA 2
## 304 O731 DIA 2
## 305 O839 DIA 2
## 306 O912 DIA 2
## 307 O992 DIA 2
## 308 Q803 DIA 2
## 309 R000 DIA 2
## 310 R092 DIA 2
## 311 R402 DIA 2
## 312 R571 DIA 2
## 313 R634 DIA 2
## 314 R64X DIA 2
## 315 S069 DIA 2
## 316 S210 DIA 2
## 317 S611 DIA 2
## 318 S789 DIA 2
## 319 S911 DIA 2
## 320 S981 DIA 2
## 321 S984 DIA 2
## 322 T600 DIA 2
## 323 T809 DIA 2
## 324 T887 DIA 2
## 325 Z302 DIA 2
## 326 A403 A150 1
## 327 A539 A199 1
## 328 B829 A090 1
## 329 C189 A099 1
## 330 C229 A419 1
## 331 C412 B24X 1
## 332 C460 B24X 1
## 333 E000 DIA 1
## 334 E031 DIA 1
## 335 E034 DIA 1
## 336 E041 DIA 1
## 337 E160 DIA 1
## 338 E169 DIA 1
## 339 E229 DIA 1
## 340 E242 DIA 1
## 341 E248 DIA 1
## 342 E270 DIA 1
## 343 E279 DIA 1
## 344 E282 D432 1
## 345 E344 DIA 1
## 346 E649 A419 1
## 347 E662 DIA 1
## 348 E673 DIA 1
## 349 E784 DIA 1
## 350 E786 DIA 1
## 351 E788 DIA 1
## 352 E789 DIA 1
## 353 E835 DIA 1
## 354 E873 A400 1
## 355 E880 DIA 1
## 356 E889 DIA 1
## 357 F064 DIA 1
## 358 F070 DIA 1
## 359 F100 DIA 1
## 360 F101 DIA 1
## 361 F129 DIA 1
## 362 F201 DIA 1
## 363 F250 DIA 1
## 364 F29X DIA 1
## 365 F313 DIA 1
## 366 F319 DIA 1
## 367 F320 DIA 1
## 368 F322 DIA 1
## 369 F410 DIA 1
## 370 F432 DIA 1
## 371 F450 A239 1
## 372 F609 DIA 1
## 373 F709 DIA 1
## 374 G009 DIA 1
## 375 G049 DIA 1
## 376 G060 DIA 1
## 377 G122 DIA 1
## 378 G403 DIA 1
## 379 G432 DIA 1
## 380 G440 DIA 1
## 381 G443 DIA 1
## 382 G459 D649 1
## 383 G510 DIA 1
## 384 G519 DIA 1
## 385 G603 DIA 1
## 386 G713 DIA 1
## 387 G729 DIA 1
## 388 G819 DIA 1
## 389 G900 DIA 1
## 390 G936 DIA 1
## 391 H043 D539 1
## 392 H050 DIA 1
## 393 H527 DIA 1
## 394 H650 DIA 1
## 395 H659 DIA 1
## 396 H818 DIA 1
## 397 H819 DIA 1
## 398 H82X DIA 1
## 399 H913 DIA 1
## 400 H920 DIA 1
## 401 I120 DIA 1
## 402 I150 DIA 1
## 403 I158 B960 1
## 404 I200 DIA 1
## 405 I208 DIA 1
## 406 I259 DIA 1
## 407 I339 DIA 1
## 408 I424 DIA 1
## 409 I459 A419 1
## 410 I510 DIA 1
## 411 I600 DIA 1
## 412 I606 DIA 1
## 413 I618 DIA 1
## 414 I635 DIA 1
## 415 I679 DIA 1
## 416 I694 B972 1
## 417 I832 DIA 1
## 418 I849 DIA 1
## 419 I889 DIA 1
## 420 J040 DIA 1
## 421 J100 DIA 1
## 422 J120 DIA 1
## 423 J122 DIA 1
## 424 J157 DIA 1
## 425 J180 DIA 1
## 426 J208 DIA 1
## 427 J219 DIA 1
## 428 J22X DIA 1
## 429 J340 DIA 1
## 430 J380 DIA 1
## 431 J398 DIA 1
## 432 J448 DIA 1
## 433 J449 A047 1
## 434 J60X DIA 1
## 435 J631 A169 1
## 436 J849 DIA 1
## 437 J850 DIA 1
## 438 J853 A418 1
## 439 J930 DIA 1
## 440 J938 A158 1
## 441 J950 DIA 1
## 442 J961 DIA 1
## 443 J981 DIA 1
## 444 J982 DIA 1
## 445 J988 DIA 1
## 446 K029 DIA 1
## 447 K041 DIA 1
## 448 K046 DIA 1
## 449 K047 D649 1
## 450 K113 DIA 1
## 451 K226 DIA 1
## 452 K250 DIA 1
## 453 K253 DIA 1
## 454 K279 DIA 1
## 455 K299 D649 1
## 456 K352 DIA 1
## 457 K388 D509 1
## 458 K400 DIA 1
## 459 K420 DIA 1
## 460 K430 DIA 1
## 461 K449 DIA 1
## 462 K515 DIA 1
## 463 K565 DIA 1
## 464 K566 DIA 1
## 465 K590 D509 1
## 466 K592 DIA 1
## 467 K602 DIA 1
## 468 K603 DIA 1
## 469 K625 DIA 1
## 470 K650 D414 1
## 471 K658 DIA 1
## 472 K709 DIA 1
## 473 K738 DIA 1
## 474 K739 DIA 1
## 475 K743 DIA 1
## 476 K818 DIA 1
## 477 K833 DIA 1
## 478 K85 E11 1
## 479 K861 DIA 1
## 480 L021 DIA 1
## 481 L048 DIA 1
## 482 L231 D509 1
## 483 L281 B353 1
## 484 L680 DIA 1
## 485 L932 DIA 1
## 486 L989 DIA 1
## 487 M139 DIA 1
## 488 M512 DIA 1
## 489 M544 DIA 1
## 490 M620 DIA 1
## 491 M623 D649 1
## 492 M725 DIA 1
## 493 M726 DIA 1
## 494 M793 DIA 1
## 495 M839 DIA 1
## 496 M861 DIA 1
## 497 M866 DIA 1
## 498 N009 D649 1
## 499 N021 DIA 1
## 500 N048 DIA 1
## 501 N078 DIA 1
## 502 N080 A409 1
## 503 N083 B373 1
## 504 N12 DIA 1
## 505 N130 DIA 1
## 506 N131 DIA 1
## 507 N164 DIA 1
## 508 N171 DIA 1
## 509 N182 DIA 1
## 510 N183 DIA 1
## 511 N184 DIA 1
## 512 N201 DIA 1
## 513 N210 DIA 1
## 514 N220 DIA 1
## 515 N298 DIA 1
## 516 N308 DIA 1
## 517 N310 DIA 1
## 518 N322 DIA 1
## 519 N368 DIA 1
## 520 N369 A419 1
## 521 N398 DIA 1
## 522 N600 DIA 1
## 523 N62X DIA 1
## 524 N649 DIA 1
## 525 N730 DIA 1
## 526 N736 DIA 1
## 527 N761 DIA 1
## 528 N766 DIA 1
## 529 N778 DIA 1
## 530 N811 DIA 1
## 531 N819 DIA 1
## 532 N829 DIA 1
## 533 N855 DIA 1
## 534 N871 DIA 1
## 535 N908 DIA 1
## 536 N912 DIA 1
## 537 N949 DIA 1
## 538 N979 DIA 1
## 539 N990 DIA 1
## 540 N994 DIA 1
## 541 O000 DIA 1
## 542 O010 DIA 1
## 543 O030 DIA 1
## 544 O054 DIA 1
## 545 O088 DIA 1
## 546 O100 DIA 1
## 547 O16X DIA 1
## 548 O211 DIA 1
## 549 O219 DIA 1
## 550 O231 DIA 1
## 551 O239 DIA 1
## 552 O243 DIA 1
## 553 O25X DIA 1
## 554 O260 DIA 1
## 555 O300 DIA 1
## 556 O312 DIA 1
## 557 O321 DIA 1
## 558 O324 DIA 1
## 559 O334 DIA 1
## 560 O338 DIA 1
## 561 O339 DIA 1
## 562 O360 DIA 1
## 563 O365 DIA 1
## 564 O368 DIA 1
## 565 O420 DIA 1
## 566 O421 DIA 1
## 567 O439 DIA 1
## 568 O471 DIA 1
## 569 O601 DIA 1
## 570 O60X DIA 1
## 571 O610 DIA 1
## 572 O639 DIA 1
## 573 O645 DIA 1
## 574 O648 DIA 1
## 575 O662 DIA 1
## 576 O669 DIA 1
## 577 O700 DIA 1
## 578 O710 DIA 1
## 579 O759 DIA 1
## 580 O8001 DIA 1
## 581 O808 DIA 1
## 582 O8284 DIA 1
## 583 O838 DIA 1
## 584 O840 B972 1
## 585 O862 DIA 1
## 586 O863 DIA 1
## 587 O868 DIA 1
## 588 O908 DIA 1
## 589 O911 DIA 1
## 590 O994 DIA 1
## 591 P95X DIA 1
## 592 Q049 DIA 1
## 593 Q249 DIA 1
## 594 Q279 DIA 1
## 595 Q501 DIA 1
## 596 Q505 DIA 1
## 597 Q602 DIA 1
## 598 Q603 DIA 1
## 599 Q96 DIA 1
## 600 R001 D649 1
## 601 R02X DIA 1
## 602 R040 DIA 1
## 603 R17X D649 1
## 604 R32X DIA 1
## 605 R33X A099 1
## 606 R458 DIA 1
## 607 R529 DIA 1
## 608 R560 DIA 1
## 609 R570 DIA 1
## 610 R578 A419 1
## 611 R609 DIA 1
## 612 R632 DIA 1
## 613 R688 DIA 1
## 614 R770 A183 1
## 615 R785 DIA 1
## 616 R826 D508 1
## 617 R960 DIA 1
## 618 S017 DIA 1
## 619 S027 DIA 1
## 620 S310 DIA 1
## 621 S364 DIA 1
## 622 S498 DIA 1
## 623 S610 DIA 1
## 624 S619 DIA 1
## 625 S628 DIA 1
## 626 S681 DIA 1
## 627 S720 DIA 1
## 628 S729 DIA 1
## 629 S810 DIA 1
## 630 S819 B353 1
## 631 S826 DIA 1
## 632 S828 DIA 1
## 633 S881 DIA 1
## 634 T013 DIA 1
## 635 T111 DIA 1
## 636 T136 DIA 1
## 637 T147 DIA 1
## 638 T210 DIA 1
## 639 T231 DIA 1
## 640 T243 D649 1
## 641 T252 DIA 1
## 642 T302 DIA 1
## 643 T304 DIA 1
## 644 T509 DIA 1
## 645 T519 DIA 1
## 646 T630 DIA 1
## 647 T659 DIA 1
## 648 T698 DIA 1
## 649 T782 DIA 1
## 650 T789 DIA 1
## 651 T793 DIA 1
## 652 T819 DIA 1
## 653 T860 DIA 1
## 654 X109 DIA 1
## 655 X690 DIA 1
## 656 Y835 DIA 1
## 657 Z3593 DIA 1
## 658 Z391 DIA 1
## 659 Z433 DIA 1
## 660 Z749 DIA 1

### Female, 40 - 59 years old

## Diag1 Diag2 Frequency
## 1 N390 DIA 3091
## 2 I10X DIA 1984
## 3 N189 DIA 679
## 4 U071 DIA 492
## 5 J189 DIA 421
## 6 D649 DIA 399
## 7 N10X DIA 362
## 8 E669 DIA 300
## 9 J960 DIA 275
## 10 A419 DIA 246
## 11 L031 DIA 246
## 12 N185 DIA 183
## 13 D509 DIA 156
## 14 E039 DIA 155
## 15 N110 DIA 152
## 16 N12X DIA 141
## 17 R739 DIA 127
## 18 I500 DIA 122
## 19 R104 DIA 120
## 20 A560 DIA 114
## 21 J969 DIA 110
## 22 E162 DIA 108
## 23 L039 DIA 108
## 24 N200 DIA 104
## 25 K802 DIA 83
## 26 K811 DIA 81
## 27 A099 DIA 78
## 28 A150 DIA 75
## 29 E86X DIA 71
## 30 J159 DIA 71
## 31 J129 DIA 68
## 32 K922 DIA 68
## 33 I509 DIA 64
## 34 N179 DIA 60
## 35 E660 DIA 58
## 36 K859 DIA 58
## 37 I64X DIA 57
## 38 J90X DIA 56
## 39 M069 DIA 56
## 40 N039 DIA 56
## 41 K297 DIA 55
## 42 S913 DIA 52
## 43 J459 DIA 50
## 44 K810 DIA 49
## 45 J128 DIA 48
## 46 L030 DIA 46
## 47 N151 DIA 46
## 48 N819 DIA 45
## 49 K801 DIA 44
## 50 I219 DIA 43
## 51 G409 DIA 39
## 52 I639 DIA 39
## 53 I678 DIA 39
## 54 N19X DIA 39
## 55 K800 DIA 38
## 56 R11X DIA 38
## 57 E668 DIA 37
## 58 L023 DIA 37
## 59 N181 DIA 37
## 60 N832 DIA 36
## 61 U072 DIA 35
## 62 J47X DIA 34
## 63 L024 DIA 34
## 64 L038 DIA 34
## 65 N739 DIA 34
## 66 N111 DIA 32
## 67 N399 DIA 32
## 68 N850 DIA 32
## 69 E872 DIA 31
## 70 K295 DIA 31
## 71 R509 DIA 31
## 72 I119 DIA 30
## 73 J80X DIA 29
## 74 J849 DIA 29
## 75 L032 DIA 29
## 76 F412 DIA 28
## 77 I679 DIA 28
## 78 K819 DIA 28
## 79 G934 DIA 27
## 80 L029 DIA 27
## 81 N184 DIA 27
## 82 R042 DIA 27
## 83 J209 DIA 26
## 84 E780 DIA 25
## 85 K291 DIA 25
## 86 K805 DIA 25
## 87 J841 DIA 24
## 88 K703 DIA 24
## 89 N760 DIA 24
## 90 A418 DIA 23
## 91 E782 DIA 23
## 92 K850 DIA 23
## 93 K85X DIA 23
## 94 N209 DIA 23
## 95 R51X DIA 23
## 96 F419 DIA 22
## 97 J449 DIA 22
## 98 N133 DIA 22
## 99 R568 DIA 22
## 100 E160 DIA 21
## 101 I619 DIA 21
## 102 I802 DIA 21
## 103 K439 DIA 21
## 104 K590 DIA 21
## 105 N119 DIA 21
## 106 I120 DIA 20
## 107 K429 DIA 20
## 108 K529 DIA 20
## 109 L028 DIA 20
## 110 A159 DIA 19
## 111 N049 DIA 19
## 112 N61X DIA 19
## 113 H360 DIA 18
## 114 J180 DIA 18
## 115 R572 DIA 18
## 116 J961 DIA 17
## 117 K610 DIA 17
## 118 L021 DIA 17
## 119 L033 DIA 17
## 120 M545 DIA 17
## 121 N180 DIA 17
## 122 R02X DIA 17
## 123 S889 DIA 17
## 124 I159 DIA 16
## 125 J029 DIA 16
## 126 K047 DIA 16
## 127 K729 DIA 16
## 128 K808 DIA 16
## 129 R18X DIA 16
## 130 S984 DIA 16
## 131 A162 DIA 15
## 132 L020 DIA 15
## 133 N814 DIA 15
## 134 N938 DIA 15
## 135 R100 DIA 15
## 136 I694 DIA 14
## 137 I739 DIA 14
## 138 I792 DIA 14
## 139 M726 DIA 14
## 140 M869 DIA 14
## 141 G590 DIA 13
## 142 I480 DIA 13
## 143 I859 DIA 13
## 144 J22X DIA 13
## 145 K566 DIA 13
## 146 L984 DIA 13
## 147 N183 DIA 13
## 148 R609 DIA 13
## 149 A409 DIA 12
## 150 E46X DIA 12
## 151 F200 DIA 12
## 152 I872 DIA 12
## 153 J81X DIA 12
## 154 L089 DIA 12
## 155 M725 DIA 12
## 156 N771 DIA 12
## 157 N813 DIA 12
## 158 R101 DIA 12
## 159 T136 DIA 12
## 160 E038 DIA 11
## 161 E059 DIA 11
## 162 E878 DIA 11
## 163 G632 DIA 11
## 164 I150 DIA 11
## 165 I829 DIA 11
## 166 K30X DIA 11
## 167 K830 DIA 11
## 168 G442 DIA 10
## 169 I259 DIA 10
## 170 I609 DIA 10
## 171 J13X DIA 10
## 172 J157 DIA 10
## 173 J188 DIA 10
## 174 J40X DIA 10
## 175 K37X DIA 10
## 176 K851 DIA 10
## 177 L890 DIA 10
## 178 M059 DIA 10
## 179 O829 DIA 10
## 180 S822 DIA 10
## 181 S828 DIA 10
## 182 T814 DIA 10
## 183 A09X DIA 9
## 184 G610 DIA 9
## 185 I200 DIA 9
## 186 I469 DIA 9
## 187 I850 DIA 9
## 188 J158 DIA 9
## 189 J46X DIA 9
## 190 K290 DIA 9
## 191 K469 DIA 9
## 192 K650 DIA 9
## 193 K750 DIA 9
## 194 M544 DIA 9
## 195 N182 DIA 9
## 196 N840 DIA 9
## 197 O034 DIA 9
## 198 R32X DIA 9
## 199 S819 DIA 9
## 200 S981 DIA 9
## 201 G819 DIA 8
## 202 H050 DIA 8
## 203 H819 DIA 8
## 204 I830 DIA 8
## 205 J120 DIA 8
## 206 J984 DIA 8
## 207 L509 DIA 8
## 208 L899 DIA 8
## 209 M139 DIA 8
## 210 N083 DIA 8
## 211 N312 DIA 8
## 212 N764 DIA 8
## 213 N830 DIA 8
## 214 O234 DIA 8
## 215 R571 DIA 8
## 216 S982 DIA 8
## 217 T252 DIA 8
## 218 T887 DIA 8
## 219 D069 DIA 7
## 220 D487 DIA 7
## 221 E041 DIA 7
## 222 E249 DIA 7
## 223 E880 DIA 7
## 224 G459 DIA 7
## 225 I693 DIA 7
## 226 I809 DIA 7
## 227 J039 DIA 7
## 228 J068 DIA 7
## 229 J100 DIA 7
## 230 J852 DIA 7
## 231 K279 DIA 7
## 232 K296 DIA 7
## 233 K760 DIA 7
## 234 L959 DIA 7
## 235 N130 DIA 7
## 236 N202 DIA 7
## 237 N23X DIA 7
## 238 N300 DIA 7
## 239 N709 DIA 7
## 240 N812 DIA 7
## 241 O342 DIA 7
## 242 S069 DIA 7
## 243 S720 DIA 7
## 244 A060 DIA 6
## 245 B461 DIA 6
## 246 B465 DIA 6
## 247 E161 DIA 6
## 248 E441 DIA 6
## 249 E781 DIA 6
## 250 F321 DIA 6
## 251 G510 DIA 6
## 252 H813 DIA 6
## 253 I499 DIA 6
## 254 I610 DIA 6
## 255 I633 DIA 6
## 256 J181 DIA 6
## 257 J91X DIA 6
## 258 K352 DIA 6
## 259 K709 DIA 6
## 260 M179 DIA 6
## 261 M199 DIA 6
## 262 N159 DIA 6
## 263 N289 DIA 6
## 264 N310 DIA 6
## 265 N870 DIA 6
## 266 N872 DIA 6
## 267 O821 DIA 6
## 268 S619 DIA 6
## 269 S911 DIA 6
## 270 T302 DIA 6
## 271 T793 DIA 6
## 272 A156 DIA 5
## 273 A279 DIA 5
## 274 C910 DIA 5
## 275 E050 DIA 5
## 276 E242 DIA 5
## 277 E871 DIA 5
## 278 E876 DIA 5
## 279 F320 DIA 5
## 280 G360 DIA 5
## 281 G619 DIA 5
## 282 H280 DIA 5
## 283 H46X DIA 5
## 284 I249 DIA 5
## 285 I255 DIA 5
## 286 I698 DIA 5
## 287 I828 DIA 5
## 288 I832 DIA 5
## 289 J069 DIA 5
## 290 J150 DIA 5
## 291 J36X DIA 5
## 292 J690 DIA 5
## 293 J869 DIA 5
## 294 K20X DIA 5
## 295 K359 DIA 5
## 296 K409 DIA 5
## 297 K420 DIA 5
## 298 K660 DIA 5
## 299 K803 DIA 5
## 300 L409 DIA 5
## 301 M321 DIA 5
## 302 N009 DIA 5
## 303 N040 DIA 5
## 304 N170 DIA 5
## 305 N220 DIA 5
## 306 N328 DIA 5
## 307 N398 DIA 5
## 308 N800 DIA 5
## 309 N818 DIA 5
## 310 N820 DIA 5
## 311 O064 DIA 5
## 312 O809 DIA 5
## 313 O990 DIA 5
## 314 R601 DIA 5
## 315 S411 DIA 5
## 316 S917 DIA 5
## 317 S980 DIA 5
## 318 T009 DIA 5
## 319 T054 DIA 5
## 320 T141 DIA 5
## 321 Z488 DIA 5
## 322 43549 DIA 4
## 323 E02X DIA 4
## 324 E049 DIA 4
## 325 E43X DIA 4
## 326 E440 DIA 4
## 327 E835 DIA 4
## 328 F323 DIA 4
## 329 F339 DIA 4
## 330 G049 DIA 4
## 331 G439 DIA 4
## 332 G618 DIA 4
## 333 G909 DIA 4
## 334 G919 DIA 4
## 335 H650 DIA 4
## 336 H811 DIA 4
## 337 H82X DIA 4
## 338 I151 DIA 4
## 339 I152 DIA 4
## 340 I158 DIA 4
## 341 I340 DIA 4
## 342 I471 DIA 4
## 343 I489 DIA 4
## 344 I48X DIA 4
## 345 I612 DIA 4
## 346 I613 DIA 4
## 347 I634 DIA 4
## 348 I743 DIA 4
## 349 I776 DIA 4
## 350 I839 DIA 4
## 351 J123 DIA 4
## 352 J440 DIA 4
## 353 J851 DIA 4
## 354 J980 DIA 4
## 355 K351 DIA 4
## 356 K564 DIA 4
## 357 K567 DIA 4
## 358 K591 DIA 4
## 359 K744 DIA 4
## 360 K759 DIA 4
## 361 K769 DIA 4
## 362 K861 DIA 4
## 363 L088 DIA 4
## 364 L309 DIA 4
## 365 L400 DIA 4
## 366 L88X DIA 4
## 367 L89X DIA 4
## 368 M009 DIA 4
## 369 M169 DIA 4
## 370 M255 DIA 4
## 371 M549 DIA 4
## 372 N000 DIA 4
## 373 N139 DIA 4
## 374 N309 DIA 4
## 375 N768 DIA 4
## 376 N971 DIA 4
## 377 R190 DIA 4
## 378 R31X DIA 4
## 379 R69X DIA 4
## 380 S681 DIA 4
## 381 S721 DIA 4
## 382 S820 DIA 4
## 383 S880 DIA 4
## 384 S912 DIA 4
## 385 T131 DIA 4
## 386 T140 DIA 4
## 387 T634 DIA 4
## 388 T813 DIA 4
## 389 E220 DIA 3
## 390 E230 DIA 3
## 391 E875 DIA 3
## 392 F059 DIA 3
## 393 F448 DIA 3
## 394 F449 DIA 3
## 395 G048 DIA 3
## 396 G408 DIA 3
## 397 G530 DIA 3
## 398 G959 DIA 3
## 399 H601 DIA 3
## 400 H669 DIA 3
## 401 I260 DIA 3
## 402 I479 DIA 3
## 403 I516 DIA 3
## 404 I519 DIA 3
## 405 I611 DIA 3
## 406 I630 DIA 3
## 407 I638 DIA 3
## 408 I770 DIA 3
## 409 I959 DIA 3
## 410 J139 DIA 3
## 411 J152 DIA 3
## 412 J156 DIA 3
## 413 J168 DIA 3
## 414 J219 DIA 3
## 415 J398 DIA 3
## 416 J42X DIA 3
## 417 J448 DIA 3
## 418 J450 DIA 3
## 419 J680 DIA 3
## 420 J948 DIA 3
## 421 K221 DIA 3
## 422 K250 DIA 3
## 423 K293 DIA 3
## 424 K294 DIA 3
## 425 K318 DIA 3
## 426 K350 DIA 3
## 427 K353 DIA 3
## 428 K603 DIA 3
## 429 K661 DIA 3
## 430 K766 DIA 3
## 431 K804 DIA 3
## 432 L102 DIA 3
## 433 L511 DIA 3
## 434 M053 DIA 3
## 435 M068 DIA 3
## 436 M331 DIA 3
## 437 M479 DIA 3
## 438 M512 DIA 3
## 439 M729 DIA 3
## 440 M866 DIA 3
## 441 N059 DIA 3
## 442 N132 DIA 3
## 443 N137 DIA 3
## 444 N158 DIA 3
## 445 N172 DIA 3
## 446 N201 DIA 3
## 447 N210 DIA 3
## 448 N288 DIA 3
## 449 N340 DIA 3
## 450 N394 DIA 3
## 451 N701 DIA 3
## 452 N751 DIA 3
## 453 N809 DIA 3
## 454 N851 DIA 3
## 455 N952 DIA 3
## 456 O149 DIA 3
## 457 O249 DIA 3
## 458 O470 DIA 3
## 459 O689 DIA 3
## 460 O758 DIA 3
## 461 R040 DIA 3
## 462 R102 DIA 3
## 463 R160 DIA 3
## 464 R33X DIA 3
## 465 R392 DIA 3
## 466 R522 DIA 3
## 467 R55X DIA 3
## 468 R570 DIA 3
## 469 R600 DIA 3
## 470 R770 DIA 3
## 471 S525 DIA 3
## 472 S723 DIA 3
## 473 S729 DIA 3
## 474 S823 DIA 3
## 475 T242 DIA 3
## 476 T633 DIA 3
## 477 T702 DIA 3
## 478 T874 DIA 3
## 479 T888 A150 3
## 480 43509 DIA 2
## 481 43553 DIA 2
## 482 43555 DIA 2
## 483 43559 DIA 2
## 484 43560 N189 2
## 485 43564 I10X 2
## 486 43566 DIA 2
## 487 43571 DIA 2
## 488 A064 DIA 2
## 489 A58X DIA 2
## 490 A599 DIA 2
## 491 E010 DIA 2
## 492 E032 DIA 2
## 493 E169 DIA 2
## 494 E232 DIA 2
## 495 E240 DIA 2
## 496 E271 DIA 2
## 497 E274 DIA 2
## 498 E308 DIA 2
## 499 E748 DIA 2
## 500 E870 DIA 2
## 501 E889 DIA 2
## 502 F130 DIA 2
## 503 F341 DIA 2
## 504 F411 DIA 2
## 505 F450 DIA 2
## 506 F459 DIA 2
## 507 F606 DIA 2
## 508 G041 DIA 2
## 509 G060 DIA 2
## 510 G20X DIA 2
## 511 G255 DIA 2
## 512 G373 DIA 2
## 513 G400 DIA 2
## 514 G588 DIA 2
## 515 G589 DIA 2
## 516 G629 DIA 2
## 517 G700 DIA 2
## 518 G802 DIA 2
## 519 G92X DIA 2
## 520 G931 DIA 2
## 521 G932 DIA 2
## 522 G969 A419 2
## 523 G990 DIA 2
## 524 H103 DIA 2
## 525 H352 DIA 2
## 526 H409 DIA 2
## 527 H490 DIA 2
## 528 H544 DIA 2
## 529 H549 DIA 2
## 530 H588 DIA 2
## 531 H620 DIA 2
## 532 H663 DIA 2
## 533 H810 DIA 2
## 534 H812 DIA 2
## 535 H903 DIA 2
## 536 I129 DIA 2
## 537 I229 DIA 2
## 538 I250 DIA 2
## 539 I251 DIA 2
## 540 I309 DIA 2
## 541 I38X DIA 2
## 542 I390 DIA 2
## 543 I420 DIA 2
## 544 I429 DIA 2
## 545 I442 DIA 2
## 546 I601 DIA 2
## 547 I629 DIA 2
## 548 I635 DIA 2
## 549 I738 DIA 2
## 550 I742 DIA 2
## 551 I771 DIA 2
## 552 I779 DIA 2
## 553 I803 DIA 2
## 554 I868 DIA 2
## 555 I890 DIA 2
## 556 J00X DIA 2
## 557 J14X DIA 2
## 558 J320 DIA 2
## 559 J386 DIA 2
## 560 J451 DIA 2
## 561 J848 DIA 2
## 562 J939 DIA 2
## 563 J941 DIA 2
## 564 J981 DIA 2
## 565 K046 DIA 2
## 566 K113 DIA 2
## 567 K122 DIA 2
## 568 K210 DIA 2
## 569 K259 DIA 2
## 570 K269 DIA 2
## 571 K298 DIA 2
## 572 K299 DIA 2
## 573 K36X DIA 2
## 574 K461 DIA 2
## 575 K625 DIA 2
## 576 K630 DIA 2
## 577 K632 DIA 2
## 578 K648 DIA 2
## 579 K659 DIA 2
## 580 K717 DIA 2
## 581 K818 DIA 2
## 582 K823 DIA 2
## 583 K829 DIA 2
## 584 K858 DIA 2
## 585 K915 DIA 2
## 586 K929 DIA 2
## 587 L209 DIA 2
## 588 L301 DIA 2
## 589 L304 DIA 2
## 590 L510 DIA 2
## 591 L600 DIA 2
## 592 L719 DIA 2
## 593 M058 DIA 2
## 594 M130 DIA 2
## 595 M131 DIA 2
## 596 M154 DIA 2
## 597 M254 DIA 2
## 598 M300 DIA 2
## 599 M318 DIA 2
## 600 M330 DIA 2
## 601 M465 DIA 2
## 602 M624 DIA 2
## 603 M625 DIA 2
## 604 M752 DIA 2
## 605 M779 DIA 2
## 606 M790 DIA 2
## 607 M860 DIA 2
## 608 M868 DIA 2
## 609 M870 DIA 2
## 610 N048 DIA 2
## 611 N050 DIA 2
## 612 N069 DIA 2
## 613 N118 DIA 2
## 614 N131 DIA 2
## 615 N188 DIA 2
## 616 N20 DIA 2
## 617 N211 DIA 2
## 618 N219 DIA 2
## 619 N301 DIA 2
## 620 N391 DIA 2
## 621 N63X DIA 2
## 622 N710 DIA 2
## 623 N72X DIA 2
## 624 N736 DIA 2
## 625 N748 DIA 2
## 626 N778 DIA 2
## 627 N810 DIA 2
## 628 N816 DIA 2
## 629 N835 DIA 2
## 630 N841 DIA 2
## 631 N871 DIA 2
## 632 N879 DIA 2
## 633 N898 DIA 2
## 634 O021 DIA 2
## 635 O100 DIA 2
## 636 O141 DIA 2
## 637 O16X DIA 2
## 638 O200 DIA 2
## 639 O230 DIA 2
## 640 O235 DIA 2
## 641 O364 DIA 2
## 642 O600 DIA 2
## 643 O669 DIA 2
## 644 O730 DIA 2
## 645 O800 DIA 2
## 646 Q501 DIA 2
## 647 Q909 DIA 2
## 648 R001 DIA 2
## 649 R070 DIA 2
## 650 R072 DIA 2
## 651 R074 DIA 2
## 652 R34X DIA 2
## 653 R402 DIA 2
## 654 R578 DIA 2
## 655 R579 DIA 2
## 656 R590 DIA 2
## 657 R820 DIA 2
## 658 R828 DIA 2
## 659 S099 DIA 2
## 660 S202 DIA 2
## 661 S311 DIA 2
## 662 S370 DIA 2
## 663 S399 DIA 2
## 664 S420 DIA 2
## 665 S422 DIA 2
## 666 S423 DIA 2
## 667 S610 DIA 2
## 668 S628 DIA 2
## 669 S682 DIA 2
## 670 S701 DIA 2
## 671 S724 DIA 2
## 672 S789 DIA 2
## 673 S808 DIA 2
## 674 S810 DIA 2
## 675 S818 DIA 2
## 676 S821 DIA 2
## 677 S826 DIA 2
## 678 S881 DIA 2
## 679 T147 DIA 2
## 680 T243 DIA 2
## 681 T253 DIA 2
## 682 T424 DIA 2
## 683 T477 DIA 2
## 684 T630 DIA 2
## 685 T740 DIA 2
## 686 T802 DIA 2
## 687 T824 DIA 2
## 688 T825 DIA 2
## 689 T857 DIA 2
## 690 Z519 DIA 2
## 691 A183 A169 1
## 692 A239 A090 1
## 693 A270 A071 1
## 694 A46X A418 1
## 695 B178 B029 1
## 696 B200 A150 1
## 697 B870 B559 1
## 698 B971 A90X 1
## 699 C269 B180 1
## 700 C765 C341 1
## 701 C919 C910 1
## 702 D070 D060 1
## 703 D169 B24X 1
## 704 D25 C64X 1
## 705 D692 A090 1
## 706 D728 D649 1
## 707 E000 DIA 1
## 708 E031 D500 1
## 709 E033 C220 1
## 710 E042 D352 1
## 711 E052 DIA 1
## 712 E055 DIA 1
## 713 E060 DIA 1
## 714 E063 DIA 1
## 715 E079 DIA 1
## 716 E11.6 DIA 1
## 717 E15X DIA 1
## 718 E208 DIA 1
## 719 E222 D352 1
## 720 E229 D352 1
## 721 E260 D509 1
## 722 E272 DIA 1
## 723 E273 A083 1
## 724 E340 A150 1
## 725 E45X DIA 1
## 726 E538 DIA 1
## 727 E559 DIA 1
## 728 E60X DIA 1
## 729 E639 DIA 1
## 730 E6691 B972 1
## 731 E66X D259 1
## 732 E720 DIA 1
## 733 E742 DIA 1
## 734 E755 DIA 1
## 735 E778 DIA 1
## 736 E784 DIA 1
## 737 E788 DIA 1
## 738 E800 DIA 1
## 739 E831 DIA 1
## 740 E859 D509 1
## 741 E873 DIA 1
## 742 E893 DIA 1
## 743 E90X DIA 1
## 744 F019 DIA 1
## 745 F050 DIA 1
## 746 F064 DIA 1
## 747 F072 DIA 1
## 748 F100 DIA 1
## 749 F102 DIA 1
## 750 F105 DIA 1
## 751 F141 DIA 1
## 752 F171 DIA 1
## 753 F172 DIA 1
## 754 F203 DIA 1
## 755 F206 DIA 1
## 756 F20X DIA 1
## 757 F21X D500 1
## 758 F229 DIA 1
## 759 F230 D060 1
## 760 F232 DIA 1
## 761 F250 DIA 1
## 762 F259 DIA 1
## 763 F311 DIA 1
## 764 F312 DIA 1
## 765 F328 DIA 1
## 766 F332 DIA 1
## 767 F409 DIA 1
## 768 F410 DIA 1
## 769 F413 DIA 1
## 770 F418 DIA 1
## 771 F432 DIA 1
## 772 F445 DIA 1
## 773 F603 DIA 1
## 774 F708 DIA 1
## 775 F719 DIA 1
## 776 F780 DIA 1
## 777 F799 D649 1
## 778 F800 DIA 1
## 779 F980 DIA 1
## 780 G000 DIA 1
## 781 G008 DIA 1
## 782 G009 DIA 1
## 783 G01X A170 1
## 784 G030 DIA 1
## 785 G038 B461 1
## 786 G042 B369 1
## 787 G050 A178 1
## 788 G10X DIA 1
## 789 G122 DIA 1
## 790 G219 DIA 1
## 791 G253 DIA 1
## 792 G309 DIA 1
## 793 G372 DIA 1
## 794 G402 DIA 1
## 795 G410 DIA 1
## 796 G419 DIA 1
## 797 G430 DIA 1
## 798 G441 DIA 1
## 799 G448 DIA 1
## 800 G458 DIA 1
## 801 G464 DIA 1
## 802 G473 DIA 1
## 803 G529 B690 1
## 804 G538 DIA 1
## 805 G540 DIA 1
## 806 G579 DIA 1
## 807 G580 A010 1
## 808 G600 DIA 1
## 809 G609 D509 1
## 810 G633 DIA 1
## 811 G638 DIA 1
## 812 G709 DIA 1
## 813 G710 DIA 1
## 814 G735 DIA 1
## 815 G822 DIA 1
## 816 G908 DIA 1
## 817 G911 DIA 1
## 818 G918 DIA 1
## 819 G930 DIA 1
## 820 G936 D71X 1
## 821 G939 DIA 1
## 822 G952 DIA 1
## 823 G998 B690 1
## 824 H000 DIA 1
## 825 H010 DIA 1
## 826 H024 DIA 1
## 827 H027 DIA 1
## 828 H043 DIA 1
## 829 H045 DIA 1
## 830 H059 DIA 1
## 831 H060 DIA 1
## 832 H062 DIA 1
## 833 H109 D649 1
## 834 H160 DIA 1
## 835 H169 DIA 1
## 836 H193 DIA 1
## 837 H208 DIA 1
## 838 H259 DIA 1
## 839 H268 DIA 1
## 840 H335 A090 1
## 841 H358 DIA 1
## 842 H428 DIA 1
## 843 H440 DIA 1
## 844 H441 DIA 1
## 845 H448 DIA 1
## 846 H472 DIA 1
## 847 H493 DIA 1
## 848 H494 DIA 1
## 849 H524 DIA 1
## 850 H540 DIA 1
## 851 H578 DIA 1
## 852 H598 DIA 1
## 853 H603 DIA 1
## 854 H609 DIA 1
## 855 H622 DIA 1
## 856 H651 D509 1
## 857 H652 DIA 1
## 858 H654 DIA 1
## 859 H900 DIA 1
## 860 H919 DIA 1
## 861 H933 DIA 1
## 862 I018 DIA 1
## 863 I050 DIA 1
## 864 I059 DIA 1
## 865 I071 DIA 1
## 866 I130 D649 1
## 867 I210 D539 1
## 868 I211 DIA 1
## 869 I212 DIA 1
## 870 I236 DIA 1
## 871 I269 DIA 1
## 872 I270 D649 1
## 873 I278 DIA 1
## 874 I280 DIA 1
## 875 I319 DIA 1
## 876 I339 DIA 1
## 877 I372 DIA 1
## 878 I392 B342 1
## 879 I432 DIA 1
## 880 I447 DIA 1
## 881 I460 DIA 1
## 882 I470 DIA 1
## 883 I490 DIA 1
## 884 I513 DIA 1
## 885 I517 DIA 1
## 886 I528 DIA 1
## 887 I600 D649 1
## 888 I602 DIA 1
## 889 I604 B373 1
## 890 I606 DIA 1
## 891 I608 B373 1
## 892 I632 DIA 1
## 893 I64 DIA 1
## 894 I660 DIA 1
## 895 I669 DIA 1
## 896 I674 DIA 1
## 897 I700 DIA 1
## 898 I719 DIA 1
## 899 I720 DIA 1
## 900 I724 DIA 1
## 901 I728 DIA 1
## 902 I801 D590 1
## 903 I808 DIA 1
## 904 I821 DIA 1
## 905 I822 DIA 1
## 906 I842 DIA 1
## 907 I848 D649 1
## 908 I849 DIA 1
## 909 I863 DIA 1
## 910 I891 DIA 1
## 911 I958 A418 1
## 912 I982 DIA 1
## 913 J010 DIA 1
## 914 J019 DIA 1
## 915 J038 DIA 1
## 916 J040 DIA 1
## 917 J060 DIA 1
## 918 J09X DIA 1
## 919 J151 DIA 1
## 920 J154 DIA 1
## 921 J200 DIA 1
## 922 J210 DIA 1
## 923 J312 DIA 1
## 924 J329 DIA 1
## 925 J342 DIA 1
## 926 J370 DIA 1
## 927 J380 D538 1
## 928 J393 DIA 1
## 929 J410 DIA 1
## 930 J45X DIA 1
## 931 J47 DIA 1
## 932 J628 DIA 1
## 933 J631 DIA 1
## 934 J64X DIA 1
## 935 J684 DIA 1
## 936 J698 DIA 1
## 937 J840 A150 1
## 938 J850 DIA 1
## 939 J853 DIA 1
## 940 J938 DIA 1
## 941 J942 C189 1
## 942 J949 DIA 1
## 943 J951 DIA 1
## 944 J958 DIA 1
## 945 J989 DIA 1
## 946 K041 DIA 1
## 947 K088 DIA 1
## 948 K099 DIA 1
## 949 K102 DIA 1
## 950 K103 DIA 1
## 951 K112 DIA 1
## 952 K115 DIA 1
## 953 K119 A09X 1
## 954 K120 C859 1
## 955 K219 DIA 1
## 956 k250 DIA 1
## 957 K251 DIA 1
## 958 k259 DIA 1
## 959 K260 D649 1
## 960 K270 DIA 1
## 961 K315 DIA 1
## 962 K316 DIA 1
## 963 K317 DIA 1
## 964 K381 DIA 1
## 965 K388 DIA 1
## 966 K400 DIA 1
## 967 K402 DIA 1
## 968 K404 DIA 1
## 969 K421 DIA 1
## 970 K430 DIA 1
## 971 K431 DIA 1
## 972 K440 C159 1
## 973 K449 DIA 1
## 974 K458 DIA 1
## 975 K509 DIA 1
## 976 K519 A419 1
## 977 K520 DIA 1
## 978 K521 DIA 1
## 979 K528 DIA 1
## 980 K559 DIA 1
## 981 K563 DIA 1
## 982 K565 DIA 1
## 983 K578 DIA 1
## 984 K579 DIA 1
## 985 K612 DIA 1
## 986 K614 DIA 1
## 987 K626 D539 1
## 988 K631 DIA 1
## 989 K635 DIA 1
## 990 K658 DIA 1
## 991 K70 DIA 1
## 992 K704 D509 1
## 993 K716 DIA 1
## 994 K719 DIA 1
## 995 K721 B373 1
## 996 K743 DIA 1
## 997 K745 DIA 1
## 998 K768 DIA 1
## 999 K81 B24 1
## 1000 K81X B24X 1
## 1001 K820 DIA 1
## 1002 K824 DIA 1
## 1003 K828 DIA 1
## 1004 K839 DIA 1
## 1005 K85 DIA 1
## 1006 K860 DIA 1
## 1007 K868 DIA 1
## 1008 K869 DIA 1
## 1009 K904 DIA 1
## 1010 K920 DIA 1
## 1011 K921 D62X 1
## 1012 L00X DIA 1
## 1013 L010 B028 1
## 1014 L03 E11 1
## 1015 L040 DIA 1
## 1016 L043 DIA 1
## 1017 L048 DIA 1
## 1018 L080 DIA 1
## 1019 L100 DIA 1
## 1020 L200 DIA 1
## 1021 L219 DIA 1
## 1022 L239 DIA 1
## 1023 L280 DIA 1
## 1024 L281 D509 1
## 1025 L292 DIA 1
## 1026 L308 DIA 1
## 1027 L401 DIA 1
## 1028 L408 DIA 1
## 1029 L508 DIA 1
## 1030 L512 DIA 1
## 1031 L739 DIA 1
## 1032 L80X DIA 1
## 1033 L817 DIA 1
## 1034 L819 A099 1
## 1035 L84X DIA 1
## 1036 L891 A099 1
## 1037 L892 DIA 1
## 1038 L893 DIA 1
## 1039 L932 DIA 1
## 1040 L958 DIA 1
## 1041 L980 B180 1
## 1042 L989 DIA 1
## 1043 M029 DIA 1
## 1044 M050 DIA 1
## 1045 M052 DIA 1
## 1046 M060 D509 1
## 1047 M0699 DIA 1
## 1048 M109 DIA 1
## 1049 M142 DIA 1
## 1050 M170 DIA 1
## 1051 M175 DIA 1
## 1052 M185 DIA 1
## 1053 M224 DIA 1
## 1054 M232 DIA 1
## 1055 M235 DIA 1
## 1056 M311 C64X 1
## 1057 M313 DIA 1
## 1058 M319 DIA 1
## 1059 M328 DIA 1
## 1060 M349 D649 1
## 1061 M354 DIA 1
## 1062 M359 DIA 1
## 1063 M430 DIA 1
## 1064 M480 DIA 1
## 1065 M490 DIA 1
## 1066 M518 DIA 1
## 1067 M530 DIA 1
## 1068 M541 DIA 1
## 1069 M543 DIA 1
## 1070 M600 DIA 1
## 1071 M609 DIA 1
## 1072 M653 DIA 1
## 1073 M659 DIA 1
## 1074 M662 DIA 1
## 1075 M688 DIA 1
## 1076 M719 DIA 1
## 1077 M728 DIA 1
## 1078 M761 DIA 1
## 1079 M766 DIA 1
## 1080 M793 DIA 1
## 1081 M795 DIA 1
## 1082 M797 DIA 1
## 1083 M809 DIA 1
## 1084 M819 DIA 1
## 1085 M842 DIA 1
## 1086 M843 DIA 1
## 1087 M844 DIA 1
## 1088 M861 DIA 1
## 1089 M863 DIA 1
## 1090 M864 DIA 1
## 1091 M878 DIA 1
## 1092 M900 DIA 1
## 1093 M932 DIA 1
## 1094 M939 D259 1
## 1095 N020 DIA 1
## 1096 N030 DIA 1
## 1097 N038 DIA 1
## 1098 N079 DIA 1
## 1099 N080 DIA 1
## 1100 N088 DIA 1
## 1101 N10 DIA 1
## 1102 N13 E14 1
## 1103 N136 DIA 1
## 1104 N138 DIA 1
## 1105 N150 D509 1
## 1106 N178 DIA 1
## 1107 N18 DIA 1
## 1108 N222 DIA 1
## 1109 N228 DIA 1
## 1110 N281 DIA 1
## 1111 N290 DIA 1
## 1112 N320 DIA 1
## 1113 N322 DIA 1
## 1114 N329 A049 1
## 1115 N350 D649 1
## 1116 N368 D500 1
## 1117 N498 DIA 1
## 1118 N500 DIA 1
## 1119 N601 DIA 1
## 1120 N61 E14 1
## 1121 N641 A419 1
## 1122 N645 DIA 1
## 1123 N649 DIA 1
## 1124 N719 DIA 1
## 1125 N731 D589 1
## 1126 N732 D539 1
## 1127 N734 DIA 1
## 1128 N750 DIA 1
## 1129 N758 DIA 1
## 1130 N761 DIA 1
## 1131 N762 DIA 1
## 1132 N763 DIA 1
## 1133 N770 DIA 1
## 1134 N808 DIA 1
## 1135 N828 DIA 1
## 1136 N829 D259 1
## 1137 N831 DIA 1
## 1138 N838 DIA 1
## 1139 N839 DIA 1
## 1140 N849 DIA 1
## 1141 N888 D27X 1
## 1142 N900 DIA 1
## 1143 N911 DIA 1
## 1144 N912 DIA 1
## 1145 N930 DIA 1
## 1146 N949 DIA 1
## 1147 N950 D649 1
## 1148 N970 DIA 1
## 1149 N979 DIA 1
## 1150 N992 DIA 1
## 1151 N993 D500 1
## 1152 O009 DIA 1
## 1153 O019 DIA 1
## 1154 O033 DIA 1
## 1155 O13X DIA 1
## 1156 O140 DIA 1
## 1157 O211 DIA 1
## 1158 O220 DIA 1
## 1159 O244 DIA 1
## 1160 O321 DIA 1
## 1161 O323 DIA 1
## 1162 O326 DIA 1
## 1163 O330 DIA 1
## 1164 O410 DIA 1
## 1165 O429 DIA 1
## 1166 O441 DIA 1
## 1167 O601 DIA 1
## 1168 O60X DIA 1
## 1169 O624 DIA 1
## 1170 O639 DIA 1
## 1171 O649 DIA 1
## 1172 O679 DIA 1
## 1173 O722 DIA 1
## 1174 O820 DIA 1
## 1175 O839 DIA 1
## 1176 O85X DIA 1
## 1177 O860 DIA 1
## 1178 O900 DIA 1
## 1179 O911 DIA 1
## 1180 O912 DIA 1
## 1181 O992 DIA 1
## 1182 P369 DIA 1
## 1183 Q219 DIA 1
## 1184 Q249 DIA 1
## 1185 Q259 DIA 1
## 1186 Q282 DIA 1
## 1187 Q505 D391 1
## 1188 Q603 DIA 1
## 1189 Q610 DIA 1
## 1190 Q631 D410 1
## 1191 Q667 DIA 1
## 1192 Q720 C300 1
## 1193 Q738 DIA 1
## 1194 Q780 DIA 1
## 1195 Q828 DIA 1
## 1196 Q831 DIA 1
## 1197 R000 C329 1
## 1198 R030 DIA 1
## 1199 R049 DIA 1
## 1200 R063 DIA 1
## 1201 R092 A049 1
## 1202 R12X DIA 1
## 1203 R13X DIA 1
## 1204 R162 DIA 1
## 1205 R198 DIA 1
## 1206 R221 DIA 1
## 1207 R224 DIA 1
## 1208 R301 D509 1
## 1209 R35X D259 1
## 1210 R390 DIA 1
## 1211 R410 DIA 1
## 1212 R418 DIA 1
## 1213 R488 DIA 1
## 1214 R51 DIA 1
## 1215 R520 DIA 1
## 1216 R529 DIA 1
## 1217 R560 A090 1
## 1218 R64X D538 1
## 1219 R650 DIA 1
## 1220 R730 DIA 1
## 1221 R740 A090 1
## 1222 R798 DIA 1
## 1223 R831 A152 1
## 1224 R900 DIA 1
## 1225 R98X DIA 1
## 1226 S008 DIA 1
## 1227 S019 DIA 1
## 1228 S025 DIA 1
## 1229 S062 DIA 1
## 1230 S064 DIA 1
## 1231 S065 DIA 1
## 1232 S06X E10X 1
## 1233 S071 DIA 1
## 1234 S151 DIA 1
## 1235 S208 DIA 1
## 1236 S210 A419 1
## 1237 S218 DIA 1
## 1238 S219 DIA 1
## 1239 S228 DIA 1
## 1240 S271 DIA 1
## 1241 S301 DIA 1
## 1242 S318 DIA 1
## 1243 S320 DIA 1
## 1244 S321 DIA 1
## 1245 S324 DIA 1
## 1246 S327 DIA 1
## 1247 S328 DIA 1
## 1248 S361 D649 1
## 1249 S364 DIA 1
## 1250 S400 DIA 1
## 1251 S410 DIA 1
## 1252 S418 DIA 1
## 1253 S421 DIA 1
## 1254 S424 DIA 1
## 1255 S489 DIA 1
## 1256 S519 DIA 1
## 1257 S531 D649 1
## 1258 S626 DIA 1
## 1259 S627 DIA 1
## 1260 S635 DIA 1
## 1261 S670 DIA 1
## 1262 S683 DIA 1
## 1263 S700 DIA 1
## 1264 S781 DIA 1
## 1265 S801 DIA 1
## 1266 S829 DIA 1
## 1267 S903 DIA 1
## 1268 S910 DIA 1
## 1269 S929 DIA 1
## 1270 S930 DIA 1
## 1271 S934 DIA 1
## 1272 S971 DIA 1
## 1273 S983 DIA 1
## 1274 S998 DIA 1
## 1275 T019 DIA 1
## 1276 T055 DIA 1
## 1277 T10X DIA 1
## 1278 T111 DIA 1
## 1279 T116 DIA 1
## 1280 T138 DIA 1
## 1281 T142 DIA 1
## 1282 T202 DIA 1
## 1283 T212 DIA 1
## 1284 T222 DIA 1
## 1285 T240 DIA 1
## 1286 T292 DIA 1
## 1287 T301 DIA 1
## 1288 T315 DIA 1
## 1289 T441 DIA 1
## 1290 T451 C509 1
## 1291 T479 DIA 1
## 1292 T543 DIA 1
## 1293 T600 DIA 1
## 1294 T639 DIA 1
## 1295 T659 DIA 1
## 1296 T709 DIA 1
## 1297 T751 DIA 1
## 1298 T78 DIA 1
## 1299 T784 DIA 1
## 1300 T801 DIA 1
## 1301 T815 DIA 1
## 1302 T818 DIA 1
## 1303 T833 DIA 1
## 1304 T835 D649 1
## 1305 T856 DIA 1
## 1306 T861 D649 1
## 1307 T868 DIA 1
## 1308 W010 DIA 1
## 1309 W019 DIA 1
## 1310 W159 DIA 1
## 1311 X259 DIA 1
## 1312 X954 DIA 1
## 1313 Z208 DIA 1
## 1314 Z392 DIA 1
## 1315 Z988 DIA 1
## 1316 Z992 DIA 1

### Female, 60 o more years old

## Diag1 Diag2 Frequency
## 1 I10X DIA 4943
## 2 N390 DIA 4129
## 3 N189 DIA 1102
## 4 J189 DIA 1048
## 5 U071 DIA 606
## 6 A419 DIA 584
## 7 J960 DIA 543
## 8 D649 DIA 505
## 9 I500 DIA 385
## 10 E162 DIA 330
## 11 I64X DIA 291
## 12 N10X DIA 289
## 13 J969 DIA 280
## 14 L031 DIA 264
## 15 N185 DIA 258
## 16 E039 DIA 245
## 17 K746 DIA 223
## 18 I509 DIA 216
## 19 K922 DIA 198
## 20 D509 DIA 183
## 21 E669 DIA 182
## 22 I639 DIA 175
## 23 I678 DIA 174
## 24 J159 DIA 161
## 25 N110 DIA 153
## 26 J849 DIA 138
## 27 A099 DIA 130
## 28 I219 DIA 123
## 29 I679 DIA 123
## 30 A090 DIA 122
## 31 J841 DIA 120
## 32 N12X DIA 118
## 33 E86X DIA 113
## 34 R739 DIA 110
## 35 A560 DIA 105
## 36 R104 DIA 102
## 37 L039 DIA 100
## 38 K811 DIA 93
## 39 N039 DIA 90
## 40 I489 DIA 86
## 41 G934 DIA 84
## 42 N819 DIA 82
## 43 K802 DIA 81
## 44 N19X DIA 81
## 45 I119 DIA 80
## 46 J90X DIA 80
## 47 K703 DIA 76
## 48 I159 DIA 72
## 49 J449 DIA 69
## 50 I694 DIA 67
## 51 A418 DIA 66
## 52 J128 DIA 64
## 53 I619 DIA 63
## 54 J961 DIA 63
## 55 D539 DIA 59
## 56 I739 DIA 59
## 57 J459 DIA 58
## 58 N813 DIA 57
## 59 I480 DIA 56
## 60 L97X DIA 56
## 61 S913 DIA 56
## 62 E160 DIA 55
## 63 J180 DIA 55
## 64 J80X DIA 55
## 65 N200 DIA 55
## 66 D500 DIA 52
## 67 J209 DIA 51
## 68 K859 DIA 51
## 69 M069 DIA 51
## 70 G20X DIA 48
## 71 L899 DIA 48
## 72 G459 DIA 47
## 73 J13X DIA 47
## 74 K801 DIA 47
## 75 K805 DIA 45
## 76 R509 DIA 45
## 77 I110 DIA 44
## 78 I872 DIA 44
## 79 J690 DIA 44
## 80 K810 DIA 43
## 81 J120 DIA 41
## 82 G409 DIA 40
## 83 K297 DIA 40
## 84 T136 DIA 39
## 85 E46X DIA 38
## 86 N184 DIA 38
## 87 K429 DIA 36
## 88 K729 DIA 36
## 89 K850 DIA 36
## 90 N180 DIA 36
## 91 I48X DIA 35
## 92 F03X DIA 34
## 93 J22X DIA 34
## 94 L030 DIA 34
## 95 N399 DIA 34
## 96 I150 DIA 33
## 97 J188 DIA 33
## 98 E785 DIA 32
## 99 I633 DIA 32
## 100 I693 DIA 32
## 101 R18X DIA 32
## 102 R568 DIA 32
## 103 U072 DIA 32
## 104 A09X DIA 31
## 105 I499 DIA 31
## 106 I610 DIA 31
## 107 K358 DIA 31
## 108 K800 DIA 31
## 109 L023 DIA 31
## 110 R11X DIA 31
## 111 S721 DIA 31
## 112 I120 DIA 29
## 113 E871 DIA 28
## 114 K439 DIA 27
## 115 K590 DIA 27
## 116 R02X DIA 27
## 117 T814 DIA 27
## 118 I200 DIA 26
## 119 I259 DIA 26
## 120 I469 DIA 26
## 121 J81X DIA 26
## 122 L038 DIA 26
## 123 N119 DIA 26
## 124 R572 DIA 26
## 125 A409 DIA 25
## 126 C539 DIA 25
## 127 N183 DIA 25
## 128 C189 DIA 24
## 129 I442 DIA 24
## 130 I830 DIA 24
## 131 J158 DIA 24
## 132 L890 DIA 24
## 133 N111 DIA 24
## 134 N181 DIA 24
## 135 N832 DIA 24
## 136 S889 DIA 24
## 137 E782 DIA 23
## 138 N289 DIA 23
## 139 N393 DIA 23
## 140 S729 DIA 23
## 141 G309 DIA 22
## 142 I209 DIA 22
## 143 I634 DIA 22
## 144 K566 DIA 22
## 145 L89X DIA 22
## 146 K291 DIA 21
## 147 K830 DIA 21
## 148 K85X DIA 21
## 149 L024 DIA 21
## 150 R51X DIA 21
## 151 D693 DIA 20
## 152 E660 DIA 20
## 153 E780 DIA 20
## 154 I490 DIA 20
## 155 K808 DIA 20
## 156 K819 DIA 20
## 157 L984 DIA 20
## 158 E872 DIA 19
## 159 I829 DIA 19
## 160 N812 DIA 19
## 161 N814 DIA 19
## 162 R17X DIA 19
## 163 S981 DIA 19
## 164 I269 DIA 18
## 165 I630 DIA 18
## 166 I802 DIA 18
## 167 K295 DIA 18
## 168 K30X DIA 18
## 169 L089 DIA 18
## 170 N319 DIA 18
## 171 S069 DIA 18
## 172 E668 DIA 17
## 173 I609 DIA 17
## 174 J448 DIA 17
## 175 K409 DIA 17
## 176 M545 DIA 17
## 177 A010 DIA 16
## 178 E059 DIA 16
## 179 E161 DIA 16
## 180 I698 DIA 16
## 181 I859 DIA 16
## 182 J100 DIA 16
## 183 R101 DIA 16
## 184 E038 DIA 15
## 185 H360 DIA 15
## 186 K750 DIA 15
## 187 N300 DIA 15
## 188 N760 DIA 15
## 189 R31X DIA 15
## 190 A169 DIA 14
## 191 E440 DIA 14
## 192 L032 DIA 14
## 193 N151 DIA 14
## 194 R100 DIA 14
## 195 C220 DIA 13
## 196 E870 DIA 13
## 197 F209 DIA 13
## 198 G819 DIA 13
## 199 J029 DIA 13
## 200 J980 DIA 13
## 201 J984 DIA 13
## 202 K920 DIA 13
## 203 L021 DIA 13
## 204 L029 DIA 13
## 205 R609 DIA 13
## 206 E876 DIA 12
## 207 G969 DIA 12
## 208 I519 DIA 12
## 209 I828 DIA 12
## 210 I850 DIA 12
## 211 J152 DIA 12
## 212 J156 DIA 12
## 213 J181 DIA 12
## 214 K259 DIA 12
## 215 M179 DIA 12
## 216 M726 DIA 12
## 217 R418 DIA 12
## 218 R570 DIA 12
## 219 S880 DIA 12
## 220 S982 DIA 12
## 221 E43X DIA 11
## 222 E880 DIA 11
## 223 F011 DIA 11
## 224 F019 DIA 11
## 225 F329 DIA 11
## 226 H050 DIA 11
## 227 I158 DIA 11
## 228 I255 DIA 11
## 229 I620 DIA 11
## 230 I635 DIA 11
## 231 I771 DIA 11
## 232 I832 DIA 11
## 233 J150 DIA 11
## 234 K250 DIA 11
## 235 K769 DIA 11
## 236 L022 DIA 11
## 237 M725 DIA 11
## 238 N771 DIA 11
## 239 N811 DIA 11
## 240 R32X DIA 11
## 241 R571 DIA 11
## 242 S822 DIA 11
## 243 T009 DIA 11
## 244 C541 DIA 10
## 245 G590 DIA 10
## 246 G909 DIA 10
## 247 I210 DIA 10
## 248 I612 DIA 10
## 249 I749 DIA 10
## 250 I809 DIA 10
## 251 J440 DIA 10
## 252 J698 DIA 10
## 253 K296 DIA 10
## 254 K610 DIA 10
## 255 K851 DIA 10
## 256 K929 DIA 10
## 257 M869 DIA 10
## 258 N049 DIA 10
## 259 N083 DIA 10
## 260 N178 DIA 10
## 261 N182 DIA 10
## 262 N209 DIA 10
## 263 R601 DIA 10
## 264 S819 DIA 10
## 265 T874 DIA 10
## 266 A90X DIA 9
## 267 E878 DIA 9
## 268 F009 DIA 9
## 269 F419 DIA 9
## 270 G610 DIA 9
## 271 I249 DIA 9
## 272 I516 DIA 9
## 273 I629 DIA 9
## 274 I743 DIA 9
## 275 J450 DIA 9
## 276 J91X DIA 9
## 277 K122 DIA 9
## 278 K290 DIA 9
## 279 L893 DIA 9
## 280 N23X DIA 9
## 281 N310 DIA 9
## 282 N312 DIA 9
## 283 N818 DIA 9
## 284 R33X DIA 9
## 285 R688 DIA 9
## 286 C221 DIA 8
## 287 C250 DIA 8
## 288 D531 DIA 8
## 289 E789 DIA 8
## 290 F412 DIA 8
## 291 G408 DIA 8
## 292 G629 DIA 8
## 293 H259 DIA 8
## 294 I340 DIA 8
## 295 I350 DIA 8
## 296 I443 DIA 8
## 297 I611 DIA 8
## 298 I674 DIA 8
## 299 I959 DIA 8
## 300 J123 DIA 8
## 301 J410 DIA 8
## 302 J42X DIA 8
## 303 K564 DIA 8
## 304 L028 DIA 8
## 305 L033 DIA 8
## 306 M059 DIA 8
## 307 M139 DIA 8
## 308 M431 DIA 8
## 309 N172 DIA 8
## 310 R001 DIA 8
## 311 R42X DIA 8
## 312 S828 DIA 8
## 313 T147 DIA 8
## 314 T813 DIA 8
## 315 W199 DIA 8
## 316 E874 DIA 7
## 317 F200 DIA 7
## 318 G255 DIA 7
## 319 H811 DIA 7
## 320 I151 DIA 7
## 321 I702 DIA 7
## 322 J157 DIA 7
## 323 J631 DIA 7
## 324 K269 DIA 7
## 325 K469 DIA 7
## 326 K659 DIA 7
## 327 K768 DIA 7
## 328 L88X DIA 7
## 329 M329 DIA 7
## 330 N394 DIA 7
## 331 R042 DIA 7
## 332 R53X DIA 7
## 333 R579 DIA 7
## 334 S065 DIA 7
## 335 S422 DIA 7
## 336 S723 DIA 7
## 337 S724 DIA 7
## 338 T887 DIA 7
## 339 A170 DIA 6
## 340 B028 DIA 6
## 341 B379 DIA 6
## 342 D391 DIA 6
## 343 D410 DIA 6
## 344 E835 DIA 6
## 345 F050 DIA 6
## 346 H269 DIA 6
## 347 H669 DIA 6
## 348 I252 DIA 6
## 349 I471 DIA 6
## 350 I48 DIA 6
## 351 I600 DIA 6
## 352 I618 DIA 6
## 353 I632 DIA 6
## 354 I775 DIA 6
## 355 I792 DIA 6
## 356 I839 DIA 6
## 357 J069 DIA 6
## 358 J46X DIA 6
## 359 J852 DIA 6
## 360 K279 DIA 6
## 361 K294 DIA 6
## 362 K625 DIA 6
## 363 K660 DIA 6
## 364 K839 DIA 6
## 365 L309 DIA 6
## 366 M009 DIA 6
## 367 M600 DIA 6
## 368 N219 DIA 6
## 369 N220 DIA 6
## 370 N309 DIA 6
## 371 N810 DIA 6
## 372 N939 DIA 6
## 373 R030 DIA 6
## 374 R770 DIA 6
## 375 S818 DIA 6
## 376 T054 DIA 6
## 377 T141 DIA 6
## 378 43566 DIA 5
## 379 E169 DIA 5
## 380 E781 DIA 5
## 381 G049 DIA 5
## 382 G400 DIA 5
## 383 G442 DIA 5
## 384 G589 DIA 5
## 385 G919 DIA 5
## 386 H409 DIA 5
## 387 H46X DIA 5
## 388 H82X DIA 5
## 389 I051 DIA 5
## 390 I129 DIA 5
## 391 I152 DIA 5
## 392 I429 DIA 5
## 393 I447 DIA 5
## 394 I631 DIA 5
## 395 I709 DIA 5
## 396 J219 DIA 5
## 397 J848 DIA 5
## 398 J949 DIA 5
## 399 J981 DIA 5
## 400 K047 DIA 5
## 401 K420 DIA 5
## 402 K632 DIA 5
## 403 K721 DIA 5
## 404 K803 DIA 5
## 405 K928 DIA 5
## 406 L020 DIA 5
## 407 L509 DIA 5
## 408 L891 DIA 5
## 409 M150 DIA 5
## 410 M819 DIA 5
## 411 N130 DIA 5
## 412 N210 DIA 5
## 413 N816 DIA 5
## 414 N938 DIA 5
## 415 N952 DIA 5
## 416 R072 DIA 5
## 417 R54X DIA 5
## 418 S202 DIA 5
## 419 S911 DIA 5
## 420 T131 DIA 5
## 421 Y835 DIA 5
## 422 Z488 DIA 5
## 423 43552 DIA 4
## 424 43580 DIA 4
## 425 D329 DIA 4
## 426 D609 DIA 4
## 427 E222 DIA 4
## 428 E441 DIA 4
## 429 E700 DIA 4
## 430 E875 DIA 4
## 431 E889 DIA 4
## 432 F059 DIA 4
## 433 F064 DIA 4
## 434 F09X DIA 4
## 435 F322 DIA 4
## 436 F410 DIA 4
## 437 F449 DIA 4
## 438 G510 DIA 4
## 439 H540 DIA 4
## 440 H701 DIA 4
## 441 H813 DIA 4
## 442 H919 DIA 4
## 443 I059 DIA 4
## 444 I060 DIA 4
## 445 I130 DIA 4
## 446 I250 DIA 4
## 447 I270 DIA 4
## 448 I459 DIA 4
## 449 I481 DIA 4
## 450 I501 DIA 4
## 451 I613 DIA 4
## 452 I614 DIA 4
## 453 I691 DIA 4
## 454 I803 DIA 4
## 455 J040 DIA 4
## 456 J068 DIA 4
## 457 J151 DIA 4
## 458 J170 DIA 4
## 459 J939 DIA 4
## 460 K221 DIA 4
## 461 K260 DIA 4
## 462 K37X DIA 4
## 463 K591 DIA 4
## 464 K623 DIA 4
## 465 K650 DIA 4
## 466 K658 DIA 4
## 467 K710 DIA 4
## 468 K719 DIA 4
## 469 K720 DIA 4
## 470 K740 DIA 4
## 471 L500 DIA 4
## 472 L89 DIA 4
## 473 L989 DIA 4
## 474 M255 DIA 4
## 475 M331 DIA 4
## 476 M490 DIA 4
## 477 N029 DIA 4
## 478 N133 DIA 4
## 479 N170 DIA 4
## 480 N188 DIA 4
## 481 N281 DIA 4
## 482 N398 DIA 4
## 483 N61X DIA 4
## 484 N872 DIA 4
## 485 N879 DIA 4
## 486 R520 DIA 4
## 487 R55X DIA 4
## 488 R58X DIA 4
## 489 R99X DIA 4
## 490 S010 DIA 4
## 491 S520 DIA 4
## 492 S525 DIA 4
## 493 S722 DIA 4
## 494 S820 DIA 4
## 495 S983 DIA 4
## 496 T07X DIA 4
## 497 T793 DIA 4
## 498 T827 DIA 4
## 499 T876 DIA 4
## 500 43519 DIA 3
## 501 43554 DIA 3
## 502 43558 DIA 3
## 503 D50 DIA 3
## 504 E02X DIA 3
## 505 E030 DIA 3
## 506 E049 DIA 3
## 507 E230 DIA 3
## 508 E538 DIA 3
## 509 E755 DIA 3
## 510 E784 DIA 3
## 511 E873 DIA 3
## 512 F010 DIA 3
## 513 F067 DIA 3
## 514 F069 DIA 3
## 515 F321 DIA 3
## 516 F448 DIA 3
## 517 G311 DIA 3
## 518 G360 DIA 3
## 519 G401 DIA 3
## 520 G402 DIA 3
## 521 G500 DIA 3
## 522 G628 DIA 3
## 523 G959 DIA 3
## 524 G98X DIA 3
## 525 G990 DIA 3
## 526 H024 DIA 3
## 527 H368 DIA 3
## 528 H663 DIA 3
## 529 I071 DIA 3
## 530 I131 DIA 3
## 531 I139 DIA 3
## 532 I211 DIA 3
## 533 I258 DIA 3
## 534 I420 DIA 3
## 535 I446 DIA 3
## 536 I460 DIA 3
## 537 I461 DIA 3
## 538 I482 DIA 3
## 539 I517 DIA 3
## 540 I64 DIA 3
## 541 I690 DIA 3
## 542 I729 DIA 3
## 543 I776 DIA 3
## 544 I779 DIA 3
## 545 I800 DIA 3
## 546 I822 DIA 3
## 547 I864 DIA 3
## 548 I99X DIA 3
## 549 J019 DIA 3
## 550 J039 DIA 3
## 551 J441 DIA 3
## 552 J679 DIA 3
## 553 J850 DIA 3
## 554 J948 DIA 3
## 555 J988 DIA 3
## 556 K20X DIA 3
## 557 K219 DIA 3
## 558 K257 DIA 3
## 559 K299 DIA 3
## 560 K319 DIA 3
## 561 K400 DIA 3
## 562 K402 DIA 3
## 563 K449 DIA 3
## 564 K562 DIA 3
## 565 K649 DIA 3
## 566 K700 DIA 3
## 567 K804 DIA 3
## 568 K85 DIA 3
## 569 L080 DIA 3
## 570 L088 DIA 3
## 571 L304 DIA 3
## 572 L80X DIA 3
## 573 L892 DIA 3
## 574 M053 DIA 3
## 575 M300 DIA 3
## 576 M512 DIA 3
## 577 M541 DIA 3
## 578 M542 DIA 3
## 579 M544 DIA 3
## 580 M793 DIA 3
## 581 M842 DIA 3
## 582 M866 DIA 3
## 583 N050 DIA 3
## 584 N059 DIA 3
## 585 N158 DIA 3
## 586 N288 DIA 3
## 587 N311 DIA 3
## 588 N320 DIA 3
## 589 N391 DIA 3
## 590 N768 DIA 3
## 591 Q250 DIA 3
## 592 R000 DIA 3
## 593 R074 DIA 3
## 594 R222 DIA 3
## 595 R392 DIA 3
## 596 R410 DIA 3
## 597 R651 DIA 3
## 598 S009 DIA 3
## 599 S223 DIA 3
## 600 S328 DIA 3
## 601 S423 DIA 3
## 602 S424 DIA 3
## 603 S700 DIA 3
## 604 S711 DIA 3
## 605 S781 DIA 3
## 606 S817 DIA 3
## 607 S829 DIA 3
## 608 S910 DIA 3
## 609 S912 DIA 3
## 610 S917 DIA 3
## 611 S980 DIA 3
## 612 T302 DIA 3
## 613 T824 DIA 3
## 614 Z100 DIA 3
## 615 43498 DIA 2
## 616 43501 I10X 2
## 617 43526 I509 2
## 618 43541 I10X 2
## 619 43547 DIA 2
## 620 43549 N390 2
## 621 43551 N390 2
## 622 43553 DIA 2
## 623 43560 DIA 2
## 624 43562 I10X 2
## 625 43564 DIA 2
## 626 43567 DIA 2
## 627 43569 N390 2
## 628 43570 DIA 2
## 629 43572 DIA 2
## 630 43574 DIA 2
## 631 43579 DIA 2
## 632 B022 DIA 2
## 633 C166 DIA 2
## 634 C310 DIA 2
## 635 C540 DIA 2
## 636 C959 DIA 2
## 637 D061 DIA 2
## 638 D521 DIA 2
## 639 D589 E46X 2
## 640 E012 DIA 2
## 641 E034 DIA 2
## 642 E042 DIA 2
## 643 E052 DIA 2
## 644 E069 DIA 2
## 645 E10 DIA 2
## 646 E200 DIA 2
## 647 E210 DIA 2
## 648 E240 DIA 2
## 649 E242 DIA 2
## 650 E271 DIA 2
## 651 E559 DIA 2
## 652 E739 DIA 2
## 653 E839 DIA 2
## 654 E840 DIA 2
## 655 E890 DIA 2
## 656 F028 DIA 2
## 657 F051 DIA 2
## 658 F058 DIA 2
## 659 F062 DIA 2
## 660 F078 DIA 2
## 661 F101 DIA 2
## 662 F130 DIA 2
## 663 F190 DIA 2
## 664 F319 DIA 2
## 665 F411 DIA 2
## 666 F445 DIA 2
## 667 F811 DIA 2
## 668 F99X DIA 2
## 669 G009 DIA 2
## 670 G042 DIA 2
## 671 G219 DIA 2
## 672 G35X DIA 2
## 673 G373 DIA 2
## 674 G403 DIA 2
## 675 G404 DIA 2
## 676 G903 DIA 2
## 677 G92X DIA 2
## 678 G931 DIA 2
## 679 G932 DIA 2
## 680 G936 DIA 2
## 681 G938 DIA 2
## 682 H010 DIA 2
## 683 H043 DIA 2
## 684 H109 DIA 2
## 685 H110 DIA 2
## 686 H280 DIA 2
## 687 H400 DIA 2
## 688 H431 DIA 2
## 689 H440 DIA 2
## 690 H527 DIA 2
## 691 H601 DIA 2
## 692 H609 DIA 2
## 693 H650 DIA 2
## 694 H700 DIA 2
## 695 H709 DIA 2
## 696 H810 DIA 2
## 697 H900 DIA 2
## 698 H903 DIA 2
## 699 I050 DIA 2
## 700 I052 DIA 2
## 701 I069 DIA 2
## 702 I132 DIA 2
## 703 I213 DIA 2
## 704 I251 DIA 2
## 705 I288 DIA 2
## 706 I309 DIA 2
## 707 I351 DIA 2
## 708 I378 DIA 2
## 709 I421 DIA 2
## 710 I440 DIA 2
## 711 I441 DIA 2
## 712 I528 DIA 2
## 713 I602 DIA 2
## 714 I607 DIA 2
## 715 I615 DIA 2
## 716 I671 DIA 2
## 717 I688 DIA 2
## 718 I700 DIA 2
## 719 I899 DIA 2
## 720 J00X DIA 2
## 721 J014 R040 2
## 722 J101 DIA 2
## 723 J14X DIA 2
## 724 J153 DIA 2
## 725 J155 DIA 2
## 726 J160 DIA 2
## 727 J200 DIA 2
## 728 J304 DIA 2
## 729 J371 DIA 2
## 730 J386 DIA 2
## 731 J390 DIA 2
## 732 J393 DIA 2
## 733 J61X DIA 2
## 734 J840 DIA 2
## 735 J851 DIA 2
## 736 J869 DIA 2
## 737 J938 DIA 2
## 738 J990 DIA 2
## 739 J998 DIA 2
## 740 K210 DIA 2
## 741 K228 DIA 2
## 742 K253 DIA 2
## 743 k259 DIA 2
## 744 K263 DIA 2
## 745 K270 DIA 2
## 746 K316 DIA 2
## 747 K318 DIA 2
## 748 K389 DIA 2
## 749 K560 DIA 2
## 750 K573 DIA 2
## 751 K574 DIA 2
## 752 K579 DIA 2
## 753 K589 DIA 2
## 754 K627 DIA 2
## 755 K628 DIA 2
## 756 K631 DIA 2
## 757 K702 DIA 2
## 758 K711 DIA 2
## 759 K712 DIA 2
## 760 K717 DIA 2
## 761 K759 DIA 2
## 762 K760 DIA 2
## 763 K766 DIA 2
## 764 K820 DIA 2
## 765 K829 DIA 2
## 766 K863 DIA 2
## 767 K918 DIA 2
## 768 L00X DIA 2
## 769 L10 DIA 2
## 770 L100 DIA 2
## 771 L270 A150 2
## 772 L409 DIA 2
## 773 L719 DIA 2
## 774 L729 DIA 2
## 775 L983 DIA 2
## 776 M125 DIA 2
## 777 M169 DIA 2
## 778 M171 DIA 2
## 779 M189 DIA 2
## 780 M321 DIA 2
## 781 M340 DIA 2
## 782 M411 DIA 2
## 783 M478 DIA 2
## 784 M479 DIA 2
## 785 M480 DIA 2
## 786 M531 DIA 2
## 787 M549 DIA 2
## 788 M712 DIA 2
## 789 M729 DIA 2
## 790 M809 DIA 2
## 791 M868 DIA 2
## 792 M879 DIA 2
## 793 M900 DIA 2
## 794 M930 DIA 2
## 795 M940 DIA 2
## 796 N000 DIA 2
## 797 N040 DIA 2
## 798 N079 DIA 2
## 799 N132 DIA 2
## 800 N139 DIA 2
## 801 N159 DIA 2
## 802 N318 DIA 2
## 803 N321 DIA 2
## 804 N322 DIA 2
## 805 N328 DIA 2
## 806 N392 DIA 2
## 807 N711 DIA 2
## 808 N72X DIA 2
## 809 N736 DIA 2
## 810 N738 DIA 2
## 811 N739 DIA 2
## 812 N750 DIA 2
## 813 N751 DIA 2
## 814 N809 DIA 2
## 815 N840 DIA 2
## 816 N842 DIA 2
## 817 N857 DIA 2
## 818 N870 DIA 2
## 819 N930 DIA 2
## 820 N994 DIA 2
## 821 Q181 DIA 2
## 822 Q211 DIA 2
## 823 R060 DIA 2
## 824 R092 DIA 2
## 825 R098 DIA 2
## 826 R103 DIA 2
## 827 R14X DIA 2
## 828 R221 DIA 2
## 829 R229 DIA 2
## 830 R450 DIA 2
## 831 R578 DIA 2
## 832 R827 DIA 2
## 833 S062 DIA 2
## 834 S066 DIA 2
## 835 S099 DIA 2
## 836 S220 DIA 2
## 837 S300 DIA 2
## 838 S310 DIA 2
## 839 S320 DIA 2
## 840 S322 DIA 2
## 841 S430 DIA 2
## 842 S526 DIA 2
## 843 S619 DIA 2
## 844 S728 DIA 2
## 845 S730 DIA 2
## 846 S770 DIA 2
## 847 S789 DIA 2
## 848 S810 DIA 2
## 849 S922 DIA 2
## 850 T008 DIA 2
## 851 T056 DIA 2
## 852 T10X DIA 2
## 853 T245 DIA 2
## 854 T250 DIA 2
## 855 T301 DIA 2
## 856 T310 DIA 2
## 857 T633 DIA 2
## 858 T671 DIA 2
## 859 T857 DIA 2
## 860 T905 DIA 2
## 861 T983 DIA 2
## 862 W180 DIA 2
## 863 A179 A159 1
## 864 A972 A270 1
## 865 B780 A060 1
## 866 B91X A180 1
## 867 C448 A414 1
## 868 C499 C241 1
## 869 C787 C189 1
## 870 C793 C349 1
## 871 D339 C719 1
## 872 D379 C786 1
## 873 D464 C679 1
## 874 D70X C833 1
## 875 D823 A419 1
## 876 E009 DIA 1
## 877 E011 DIA 1
## 878 E031 DIA 1
## 879 E033 DIA 1
## 880 E040 DIA 1
## 881 E041 DIA 1
## 882 E055 DIA 1
## 883 E079 DIA 1
## 884 E111D D250 1
## 885 E15X DIA 1
## 886 E168 DIA 1
## 887 E209 DIA 1
## 888 E211 DIA 1
## 889 E220 DIA 1
## 890 E229 DIA 1
## 891 E232 DIA 1
## 892 E233 DIA 1
## 893 E237 DIA 1
## 894 E270 DIA 1
## 895 E350 DIA 1
## 896 E40X DIA 1
## 897 E43 C169 1
## 898 E45X DIA 1
## 899 E509 DIA 1
## 900 E662 DIA 1
## 901 E722 DIA 1
## 902 E729 DIA 1
## 903 E752 DIA 1
## 904 E779 DIA 1
## 905 E790 DIA 1
## 906 E806 A090 1
## 907 E807 DIA 1
## 908 E849 DIA 1
## 909 E850 DIA 1
## 910 E859 DIA 1
## 911 F000 DIA 1
## 912 F002 DIA 1
## 913 F012 DIA 1
## 914 F013 DIA 1
## 915 F018 DIA 1
## 916 F068 DIA 1
## 917 F072 DIA 1
## 918 F102 DIA 1
## 919 F105 DIA 1
## 920 F132 DIA 1
## 921 F189 DIA 1
## 922 F203 DIA 1
## 923 F206 DIA 1
## 924 F239 DIA 1
## 925 F29X D649 1
## 926 F316 DIA 1
## 927 F320 DIA 1
## 928 F323 DIA 1
## 929 F328 DIA 1
## 930 F330 DIA 1
## 931 F332 DIA 1
## 932 F341 DIA 1
## 933 F409 DIA 1
## 934 F41 DIA 1
## 935 F418 DIA 1
## 936 F432 DIA 1
## 937 F481 DIA 1
## 938 F504 DIA 1
## 939 F509 DIA 1
## 940 F510 DIA 1
## 941 F519 DIA 1
## 942 F604 DIA 1
## 943 F639 DIA 1
## 944 F799 DIA 1
## 945 F82X DIA 1
## 946 G008 DIA 1
## 947 G039 DIA 1
## 948 G041 DIA 1
## 949 G050 A169 1
## 950 G051 DIA 1
## 951 G061 DIA 1
## 952 G112 DIA 1
## 953 G114 DIA 1
## 954 G122 DIA 1
## 955 G20 DIA 1
## 956 G211 DIA 1
## 957 G231 DIA 1
## 958 G249 DIA 1
## 959 G253 DIA 1
## 960 G300 DIA 1
## 961 G301 DIA 1
## 962 G310 DIA 1
## 963 G369 DIA 1
## 964 G370 DIA 1
## 965 G371 DIA 1
## 966 G379 DIA 1
## 967 G410 DIA 1
## 968 G412 DIA 1
## 969 G430 DIA 1
## 970 G439 DIA 1
## 971 G448 DIA 1
## 972 G450 DIA 1
## 973 G460 DIA 1
## 974 G464 DIA 1
## 975 G519 DIA 1
## 976 G530 DIA 1
## 977 G569 DIA 1
## 978 G579 DIA 1
## 979 G588 D443 1
## 980 G700 DIA 1
## 981 G709 DIA 1
## 982 G710 DIA 1
## 983 G729 DIA 1
## 984 G732 A418 1
## 985 G800 DIA 1
## 986 G825 DIA 1
## 987 G900 DIA 1
## 988 G911 DIA 1
## 989 G912 DIA 1
## 990 G941 D432 1
## 991 G948 DIA 1
## 992 G952 DIA 1
## 993 G968 DIA 1
## 994 G998 B690 1
## 995 H059 DIA 1
## 996 H060 DIA 1
## 997 H062 DIA 1
## 998 H103 B029 1
## 999 H113 DIA 1
## 1000 H162 DIA 1
## 1001 H189 C539 1
## 1002 H282 DIA 1
## 1003 H350 DIA 1
## 1004 H352 DIA 1
## 1005 H359 DIA 1
## 1006 H451 DIA 1
## 1007 H490 DIA 1
## 1008 H492 DIA 1
## 1009 H588 B023 1
## 1010 H600 DIA 1
## 1011 H602 DIA 1
## 1012 H604 DIA 1
## 1013 H605 DIA 1
## 1014 H611 DIA 1
## 1015 H651 DIA 1
## 1016 H660 DIA 1
## 1017 H830 DIA 1
## 1018 H901 A161 1
## 1019 I010 DIA 1
## 1020 I061 DIA 1
## 1021 I080 DIA 1
## 1022 I089 DIA 1
## 1023 I214 DIA 1
## 1024 I233 DIA 1
## 1025 I238 DIA 1
## 1026 I256 B342 1
## 1027 I260 DIA 1
## 1028 I272 DIA 1
## 1029 I278 DIA 1
## 1030 I311 A156 1
## 1031 I321 DIA 1
## 1032 I339 DIA 1
## 1033 I358 DIA 1
## 1034 I361 DIA 1
## 1035 I390 DIA 1
## 1036 I411 DIA 1
## 1037 I422 DIA 1
## 1038 I438 DIA 1
## 1039 I451 DIA 1
## 1040 I452 DIA 1
## 1041 I454 DIA 1
## 1042 I479 DIA 1
## 1043 I493 DIA 1
## 1044 I495 DIA 1
## 1045 I498 DIA 1
## 1046 I510 DIA 1
## 1047 I603 DIA 1
## 1048 I604 DIA 1
## 1049 I606 DIA 1
## 1050 I608 DIA 1
## 1051 I61 E11 1
## 1052 I652 DIA 1
## 1053 I661 DIA 1
## 1054 I670 DIA 1
## 1055 I676 DIA 1
## 1056 I69 DIA 1
## 1057 I692 DIA 1
## 1058 I719 DIA 1
## 1059 I728 D649 1
## 1060 I730 DIA 1
## 1061 I738 DIA 1
## 1062 I745 DIA 1
## 1063 I770 DIA 1
## 1064 I801 DIA 1
## 1065 I821 DIA 1
## 1066 I831 DIA 1
## 1067 I840 DIA 1
## 1068 I842 DIA 1
## 1069 I848 DIA 1
## 1070 I870 DIA 1
## 1071 I871 C349 1
## 1072 I889 DIA 1
## 1073 I890 DIA 1
## 1074 I951 D649 1
## 1075 I982 DIA 1
## 1076 I988 DIA 1
## 1077 J010 DIA 1
## 1078 J042 DIA 1
## 1079 J110 D509 1
## 1080 J118 DIA 1
## 1081 J139 DIA 1
## 1082 J15 A90 1
## 1083 J173 B59X 1
## 1084 J178 DIA 1
## 1085 J182 DIA 1
## 1086 J20 DIA 1
## 1087 J208 D649 1
## 1088 J20X DIA 1
## 1089 J210 DIA 1
## 1090 J320 DIA 1
## 1091 J342 DIA 1
## 1092 J36X DIA 1
## 1093 J380 B909 1
## 1094 J392 DIA 1
## 1095 J40 E11 1
## 1096 J41 E14 1
## 1097 J439 D649 1
## 1098 J45 E11 1
## 1099 J451 DIA 1
## 1100 J458 DIA 1
## 1101 J633 DIA 1
## 1102 J634 D649 1
## 1103 J65X DIA 1
## 1104 J671 DIA 1
## 1105 J677 DIA 1
## 1106 J691 DIA 1
## 1107 J853 DIA 1
## 1108 J90 C500 1
## 1109 J929 DIA 1
## 1110 J941 DIA 1
## 1111 J942 DIA 1
## 1112 J950 C329 1
## 1113 J96 A90 1
## 1114 J986 DIA 1
## 1115 K040 DIA 1
## 1116 K044 DIA 1
## 1117 K046 D165 1
## 1118 K052 DIA 1
## 1119 K068 DIA 1
## 1120 K109 DIA 1
## 1121 K113 DIA 1
## 1122 K118 DIA 1
## 1123 K225 DIA 1
## 1124 K251 DIA 1
## 1125 K252 DIA 1
## 1126 K254 D539 1
## 1127 K255 DIA 1
## 1128 K264 DIA 1
## 1129 K267 DIA 1
## 1130 K274 DIA 1
## 1131 K275 A419 1
## 1132 K292 DIA 1
## 1133 K293 DIA 1
## 1134 K298 DIA 1
## 1135 K310 DIA 1
## 1136 K314 DIA 1
## 1137 K317 D649 1
## 1138 K352 DIA 1
## 1139 K36X DIA 1
## 1140 K403 DIA 1
## 1141 K410 DIA 1
## 1142 K421 DIA 1
## 1143 K440 DIA 1
## 1144 K450 DIA 1
## 1145 K458 DIA 1
## 1146 K460 A419 1
## 1147 K461 DIA 1
## 1148 K512 D500 1
## 1149 K519 DIA 1
## 1150 K550 DIA 1
## 1151 K559 A419 1
## 1152 K565 DIA 1
## 1153 K567 DIA 1
## 1154 K570 DIA 1
## 1155 K572 DIA 1
## 1156 K59 DIA 1
## 1157 K593 DIA 1
## 1158 K601 DIA 1
## 1159 K611 DIA 1
## 1160 K612 DIA 1
## 1161 K630 DIA 1
## 1162 K633 D500 1
## 1163 K635 DIA 1
## 1164 K648 DIA 1
## 1165 K66 E14 1
## 1166 K701 D649 1
## 1167 K713 A099 1
## 1168 K714 DIA 1
## 1169 K718 DIA 1
## 1170 K730 DIA 1
## 1171 K754 DIA 1
## 1172 K758 DIA 1
## 1173 K767 DIA 1
## 1174 K778 DIA 1
## 1175 K80 DIA 1
## 1176 K80X DIA 1
## 1177 K821 DIA 1
## 1178 K823 B670 1
## 1179 K828 DIA 1
## 1180 K831 C259 1
## 1181 K833 DIA 1
## 1182 K860 DIA 1
## 1183 K861 DIA 1
## 1184 K862 DIA 1
## 1185 K868 DIA 1
## 1186 K90 E14 1
## 1187 K913 DIA 1
## 1188 K914 DIA 1
## 1189 K921 DIA 1
## 1190 K938 DIA 1
## 1191 L010 DIA 1
## 1192 L043 DIA 1
## 1193 L059 DIA 1
## 1194 L121 D649 1
## 1195 L22X DIA 1
## 1196 L238 DIA 1
## 1197 L239 DIA 1
## 1198 L282 DIA 1
## 1199 L298 DIA 1
## 1200 L400 D649 1
## 1201 L502 DIA 1
## 1202 L511 DIA 1
## 1203 L539 DIA 1
## 1204 L570 DIA 1
## 1205 L600 DIA 1
## 1206 L82X DIA 1
## 1207 L853 DIA 1
## 1208 L871 DIA 1
## 1209 L921 DIA 1
## 1210 L930 DIA 1
## 1211 L931 DIA 1
## 1212 L940 DIA 1
## 1213 L982 DIA 1
## 1214 L988 DIA 1
## 1215 L998 DIA 1
## 1216 M000 DIA 1
## 1217 M050 DIA 1
## 1218 M060 DIA 1
## 1219 M082 DIA 1
## 1220 M131 DIA 1
## 1221 M153 DIA 1
## 1222 M154 B870 1
## 1223 M159 A090 1
## 1224 M160 DIA 1
## 1225 M161 DIA 1
## 1226 M172 DIA 1
## 1227 M190 DIA 1
## 1228 M198 DIA 1
## 1229 M259 DIA 1
## 1230 M317 DIA 1
## 1231 M348 DIA 1
## 1232 M349 DIA 1
## 1233 M413 DIA 1
## 1234 M418 DIA 1
## 1235 M421 A498 1
## 1236 M489 DIA 1
## 1237 M502 DIA 1
## 1238 M510 DIA 1
## 1239 M548 DIA 1
## 1240 M620 DIA 1
## 1241 M623 DIA 1
## 1242 M651 DIA 1
## 1243 M659 DIA 1
## 1244 M722 DIA 1
## 1245 M751 D693 1
## 1246 M755 DIA 1
## 1247 M758 DIA 1
## 1248 M760 D500 1
## 1249 M770 DIA 1
## 1250 M790 DIA 1
## 1251 M792 DIA 1
## 1252 M795 DIA 1
## 1253 M796 DIA 1
## 1254 M800 DIA 1
## 1255 M841 DIA 1
## 1256 M844 DIA 1
## 1257 M848 DIA 1
## 1258 M854 C050 1
## 1259 M861 DIA 1
## 1260 M862 A419 1
## 1261 M870 B972 1
## 1262 M901 DIA 1
## 1263 M903 DIA 1
## 1264 M906 DIA 1
## 1265 M932 DIA 1
## 1266 M939 DIA 1
## 1267 M948 DIA 1
## 1268 M966 DIA 1
## 1269 M990 DIA 1
## 1270 N028 DIA 1
## 1271 N038 D538 1
## 1272 N058 DIA 1
## 1273 N070 DIA 1
## 1274 N10 DIA 1
## 1275 N118 DIA 1
## 1276 N131 C531 1
## 1277 N140 DIA 1
## 1278 N144 DIA 1
## 1279 N150 DIA 1
## 1280 N165 DIA 1
## 1281 N18 DIA 1
## 1282 N201 DIA 1
## 1283 N202 D649 1
## 1284 N21 E13 1
## 1285 N211 DIA 1
## 1286 N218 DIA 1
## 1287 N228 D649 1
## 1288 N258 DIA 1
## 1289 N290 DIA 1
## 1290 N302 DIA 1
## 1291 N303 DIA 1
## 1292 N304 D501 1
## 1293 N350 DIA 1
## 1294 N359 DIA 1
## 1295 N362 DIA 1
## 1296 N370 DIA 1
## 1297 N700 DIA 1
## 1298 N710 D649 1
## 1299 N719 DIA 1
## 1300 N76 DIA 1
## 1301 N761 DIA 1
## 1302 N763 DIA 1
## 1303 N765 D049 1
## 1304 N766 DIA 1
## 1305 N770 DIA 1
## 1306 N820 DIA 1
## 1307 N8324 DIA 1
## 1308 N835 DIA 1
## 1309 N871 B909 1
## 1310 N888 D649 1
## 1311 N890 DIA 1
## 1312 N891 DIA 1
## 1313 N898 DIA 1
## 1314 N900 DIA 1
## 1315 N909 DIA 1
## 1316 N951 E10X 1
## 1317 N993 DIA 1
## 1318 Q046 DIA 1
## 1319 Q253 DIA 1
## 1320 Q273 DIA 1
## 1321 Q282 DIA 1
## 1322 Q400 DIA 1
## 1323 Q444 D649 1
## 1324 Q605 DIA 1
## 1325 Q620 DIA 1
## 1326 Q649 DIA 1
## 1327 Q660 DIA 1
## 1328 Q667 DIA 1
## 1329 R011 DIA 1
## 1330 R049 DIA 1
## 1331 R05X A419 1
## 1332 R071 DIA 1
## 1333 R090 DIA 1
## 1334 R102 DIA 1
## 1335 R13X C220 1
## 1336 R35X A419 1
## 1337 R398 DIA 1
## 1338 R402 D509 1
## 1339 R529 DIA 1
## 1340 R560 DIA 1
## 1341 R599 DIA 1
## 1342 R600 DIA 1
## 1343 R64X DIA 1
## 1344 R652 DIA 1
## 1345 R680 A403 1
## 1346 R69X A419 1
## 1347 R730 DIA 1
## 1348 R749 DIA 1
## 1349 R828 DIA 1
## 1350 S001 DIA 1
## 1351 S007 DIA 1
## 1352 S019 DIA 1
## 1353 S060 DIA 1
## 1354 S064 DIA 1
## 1355 S127 DIA 1
## 1356 S129 DIA 1
## 1357 S219 DIA 1
## 1358 S230 DIA 1
## 1359 S270 DIA 1
## 1360 S271 DIA 1
## 1361 S299 DIA 1
## 1362 S301 DIA 1
## 1363 S309 DIA 1
## 1364 S311 DIA 1
## 1365 S321 DIA 1
## 1366 S324 DIA 1
## 1367 S400 DIA 1
## 1368 S408 DIA 1
## 1369 S411 DIA 1
## 1370 S429 DIA 1
## 1371 S459 DIA 1
## 1372 S460 DIA 1
## 1373 S481 DIA 1
## 1374 S518 DIA 1
## 1375 S521 DIA 1
## 1376 S522 DIA 1
## 1377 S523 DIA 1
## 1378 S550 DIA 1
## 1379 S589 DIA 1
## 1380 S609 B360 1
## 1381 S626 DIA 1
## 1382 S628 DIA 1
## 1383 S668 DIA 1
## 1384 S682 DIA 1
## 1385 S718 DIA 1
## 1386 S748 DIA 1
## 1387 S749 DIA 1
## 1388 S799 DIA 1
## 1389 S800 DIA 1
## 1390 S821 DIA 1
## 1391 S823 DIA 1
## 1392 S826 DIA 1
## 1393 S849 DIA 1
## 1394 S878 DIA 1
## 1395 S903 DIA 1
## 1396 S909 DIA 1
## 1397 S920 DIA 1
## 1398 S925 D649 1
## 1399 S927 DIA 1
## 1400 S936 A480 1
## 1401 S960 DIA 1
## 1402 S999 DIA 1
## 1403 T055 DIA 1
## 1404 T093 DIA 1
## 1405 T116 DIA 1
## 1406 T140 DIA 1
## 1407 T143 DIA 1
## 1408 T149 DIA 1
## 1409 T150 DIA 1
## 1410 T180 DIA 1
## 1411 T189 DIA 1
## 1412 T191 DIA 1
## 1413 T214 A419 1
## 1414 T243 DIA 1
## 1415 T252 DIA 1
## 1416 T293 DIA 1
## 1417 T303 DIA 1
## 1418 T348 DIA 1
## 1419 T424 DIA 1
## 1420 T477 DIA 1
## 1421 T509 DIA 1
## 1422 T543 A050 1
## 1423 T600 DIA 1
## 1424 T630 DIA 1
## 1425 T709 DIA 1
## 1426 T784 A090 1
## 1427 T794 DIA 1
## 1428 T825 DIA 1
## 1429 T856 A419 1
## 1430 T859 DIA 1
## 1431 U069 DIA 1
## 1432 U202 DIA 1
## 1433 W018 DIA 1
## 1434 W019 DIA 1
## 1435 W060 DIA 1
## 1436 W089 DIA 1
## 1437 W170 DIA 1
## 1438 W184 DIA 1
## 1439 X100 DIA 1
## 1440 Y423 DIA 1
## 1441 Y822 DIA 1
## 1442 Y834 DIA 1
## 1443 Y846 DIA 1
## 1444 Z226 B86X 1
## 1445 Z519 DIA 1
## 1446 Z540 DIA 1
## 1447 Z639 DIA 1
## 1448 Z730 DIA 1
## 1449 Z896 DIA 1
## 1450 Z932 DIA 1
## 1451 Z958 DIA 1
